# Physical Adaptations to High‐Intensity Multimodal Training in Recreationally Active Adults: A Randomised Control Trial

**DOI:** 10.1002/ejsc.70199

**Published:** 2026-06-12

**Authors:** Tijana Sharp, Katie Slattery, Samuel Higham, Aaron J. Coutts, Nathan Mageropolous, Andrew R. Novak, Mark Brewer, Lee Wallace

**Affiliations:** ^1^ School of Human Performance, Rehabilitation and Population Health, Faculty of Health University of Technology Sydney Moore Park Sydney Australia; ^2^ Human Performance Research Centre, Faculty of Health University of Technology Sydney Moore Park Sydney Australia; ^3^ Performance Everyday Neutral Bay Sydney Australia

**Keywords:** aerobic fitness, body composition, concurrent training, high‐intensity multimodal training, muscular strength

## Abstract

High‐intensity multimodal training (HIMT), combines aerobic and resistance exercise within a single session, offering a time‐efficient training mode. However, the efficacy of HIMT for physical adaptations compared to other combined training models (e.g., inter‐session concurrent training [ISCT]) remains unclear. This two‐arm parallel randomised controlled trial examined the physical adaptations of 7 weeks of HIMT compared to a matched active control (ISCT) in recreationally active adults. The HIMT group completed 4 × 45‐min sessions/week integrating aerobic and resistance exercises. The ISCT group completed 2 × 45‐min resistance sessions/week and 2 × 45‐min aerobic sessions/week. Training intensity was prescribed at ≥ 7 RPE (CR‐10) with weekly volume and exercise selection matched across groups. Maximal strength, aerobic capacity, anthropometry and body composition were assessed pre and post‐intervention. Both HIMT (*n* = 38) and ISCT (*n* = 37) demonstrated small improvements in upper‐ and lower‐body strength, peak oxygen uptake and maximum work rate (*p* < 0.05). Trivial but significant improvements were also observed in fat mass, body fat percentage, lean mass, waist circumference and waist‐to‐hip ratio (*p* < 0.05). A modest group × time interaction favoured ISCT for reducing body fat percentage (*b* = −0.628, 95% CI [−1.21 to −0.046], *p* = 0.035 and *d* = −0.082). Seven weeks of HIMT and ISCT improved muscular strength, aerobic fitness and body composition. Separating high‐intensity aerobic and resistance sessions across days may offer a small advantage for reducing relative body fat. Longer interventions may be needed for greater adaptations.

**Trial Registration:**

ETH24‐9364, ACTRN12624000556549, Protocol: available on the Open Science Framework

## Highlights

- HIMT is an effective strategy for improving physical health and performance in recreationally active adults.
- Maximal strength, aerobic fitness and body composition improved similarly in both groups. Comparable findings suggest that physical outcomes are more associated with overall aerobic and resistance training exposure rather than differences in exercise order.
- Moderate external loading (≥ 7 RPE for 10 reps/set) is sufficient to promote strength adaptations in this population. This is likely more accessible for community gym settings compared to traditional very high loads and low repetition sets.

## 1 Introduction

Current physical activity guidelines recommend regular participation in aerobic (i.e., ≥ 30 min of moderate intensity on 5 days/week or ≥ 20 min of vigorous intensity on 2 days/week) and resistance‐based exercise (i.e., ≥ 2 days/week) for positive health outcomes (Warburton and Bredin 2017; Penedo and Dahn 2005; American College of Sports Medicine 2025). Despite this, adherence remains low due to common barriers such as lack of time, reduced enjoyment and/or motivation (Ekkekakis et al. 2008; Ryan and Deci 2000). High‐intensity multimodal training (HIMT) refers to all styles of exercise that combine aerobic, resistance and/or bodyweight training performed at a high or vigorous intensity (e.g., CrossFit and group fitness) (Sharp et al. 2022). HIMT has taken interest in the community as a proposed time efficient way for individuals to achieve the combined physical activity guidelines.

The positive effects of HIMT on health and physical performance when examined in isolation and compared to a sedentary or habitual daily activity control group are well demonstrated (Ballesta‐García et al. 2019; Batrakoulis et al. 2018; McRae et al. 2012; Sperlich et al. 2017). However, the magnitude of these effects remains unclear when compared to other methods of combined training (e.g., concurrent training involving separate blocks or days of aerobic and resistance exercise). In particular, the impact of exercise modality order in HIMT on the training stimulus and physical adaptations remains unclear. Previous findings have demonstrated the impact of aerobic and resistance exercise order on training stimuli and subsequent adaptations within traditional concurrent training (Eddens et al. 2018; Vechin et al. 2021). This phenomenon described as the interference effect refers to attenuated musculoskeletal adaptations (i.e., strength and hypertrophy) when aerobic and resistance exercise blocks occur subsequently within a single session or within separate sessions (Eddens et al. 2018). This effect appears to occur when molecular signalling pathways induced by aerobic exercise inhibit those pathways responsible for regulating protein synthesis and/or increasing protein anabolism (e.g., human growth hormone and testosterone) (Fyfe et al. 2014; Kraemer and Ratamess 2005; Kraemer et al. 2016). Other mechanisms of the interference effect may include the reduction of the anabolic response to resistance exercise or indirect compromise of the resistance training stimulus (due to residual fatigue or substrate depletion) (Fyfe et al. 2014). It is plausible that the interference effect may be relevant to HIMT given the continuous combination of aerobic and resistance exercises (i.e., strength‐endurance peripheral stimulus) that occurs within a single session (often with minimal rest periods) (Vechin et al. 2021; Schlegel 2020).

Moreover, the combination of exercise modes in HIMT may contribute to a negative perceptual response in training, wherein typical rapid transitions between exercises may contribute to increased perception of effort, reduced training quality or exercise enjoyment. Although this interference effect may be detrimental to athletes or populations with specific muscular fitness goals (e.g., maximal strength), it may be less relevant to the general population with more holistic training goals (e.g., health and wellbeing). Despite the potential attenuation of specific muscular gains, HIMT stands to provide a simultaneous aerobic and resistance exercise stimulus and proposed alternate method for individuals to meet combined physical activity guidelines. Furthermore, the sequenced combination of exercise modalities with reduced rest in HIMT may instead contribute to an ‘interaction’ effect, whereby greater physiological work and central or peripheral fatigue is accumulated during a session compared to concurrent training, contributing to favourable endurance adaptations (Carroll et al. 2016). Minimal rest periods may also promote a sustained elevation in heart rate (HR) eliciting a greater aerobic stimulus.

The presence and mechanisms of a potential interference or ‘interaction’ effect in HIMT remain unclear due to methodological limitations in the existing literature, including a lack of rigorous comparisons with matched active control groups (e.g., separate modes of aerobic and resistance training) and inconsistent standardisation and reporting of training dose and prescription (e.g., volume, intensity, exercise selection and order). These issues limit the synthesis and comparison of findings reducing the overall understanding of the efficacy of HIMT (Slade et al. 2016). Accordingly, this study examined the physical outcomes of 7‐weeks of HIMT (i.e., within‐session combined aerobic and resistance training) compared with a volume‐ and intensity‐matched active control condition in which the same aerobic and resistance exercises were separated across sessions (i.e., inter‐session concurrent training [ISCT]). The primary aim was to determine whether training structure (i.e., exercise mode order) influences adaptations in maximal strength, aerobic fitness and body composition when prescribed training dose is matched. Improved understanding of these outcomes may support informed decision‐making at both practitioner and participant levels with implications for HIMT service delivery and long‐term exercise engagement.

## 2 Methods

**FIGURE 1:**
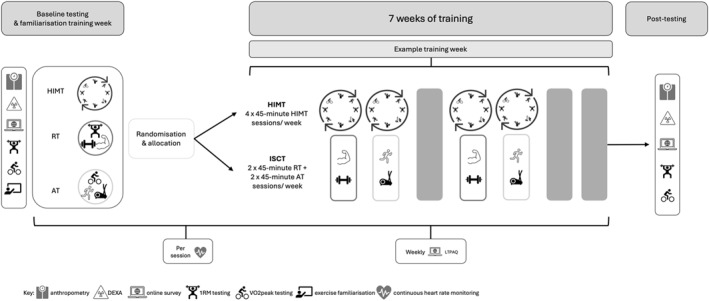
Outline of the study procedures. AT, aerobic training; HIMT, high‐intensity multimodal training; ISCT, inter‐session concurrent training; LTPAQ, leisure time physical activity scale; RT, resistance training *, optional make up sessions were provided.

This study implemented a nonblinded randomised (1:1 ratio) 2‐arm parallel design (Figure 1) to investigate the physical outcomes of 7‐weeks of HIMT compared to a matched separate aerobic and resistance training control group (ISCT). Complete methodological details are available on The Open Science Framework (OSF). Physical outcome data collection occurred at baseline and postintervention (Figure 1). The study ran from June 2024 to November 2024 across three locations. Recruitment involved an arm's length approach via email and social media. This study protocol was preregistered with the Australian New Zealand Clinical Trials Registry (ACTRN12624000556549) and prepared in accordance with the Consensus on Exercise Reporting Template (CERT) (Slade et al. 2016) and the Consolidated Standards of Reporting Trials (CONSORT) (Hopewell et al. 2025). This study received ethics approval from the University Human Research Ethics Committee (HREC ID: ETH24‐9364) with all participants providing written informed consent.

### 2.1 Participants

A priori sample size calculation, *n* = 47, *n* = 24 per group to detect small to medium effect size for the primary outcome measure (muscular strength) was performed using G*Power software (Version 3.1). Power calculations were based on the primary outcome of muscular strength (alpha level = 0.05, power = 0.80 and effect size 0.42) (Cohen's *f*) (Currier et al. 2023). The effect size (0.42) has been observed in previous research comparing the effects of resistance exercise protocols (low external load) for muscular strength (Currier et al. 2023). Assuming a drop out of ∼20%, 29 participants per group was estimated, total *n* = 58 (Collins et al. 2022). To be eligible, participants were aged 18–65 years, recreationally active and apparently healthy. Recreationally active was defined as at least 30 min of moderate or 20 min of vigorous activity on ≥ 3 days per week for the past 6 months with at least one weekly session involving resistance training (e.g., weights and bodyweight exercises). This ensured participants were accustomed to both aerobic and resistance exercise stimuli at levels consistent with physical activity guidelines reducing potential confounding from training status.

### 2.2 Data Collection

Testing at baseline and post‐testing took place in climate‐controlled conditions (i.e., resistance training and exercise physiology laboratory facilities). Participants arrived fasted at an agreed upon time between 6:00 a.m. to 9:00 a.m. and were required to avoid alcohol for 24 h, refrain from exercise (beyond activities of daily living) for 48 h, wear minimal clothing (exercise attire), void their bladder and remove jewellery and metal. These requirements were communicated verbally and via infographic during the virtual familiarisation session. After body composition assessment, participants were provided carbohydrate‐rich snacks and fluids to standardise fuelling before maximal strength testing. Participants completed a 24‐h diet recall before baseline testing (Qualtrics XM Platform 2024, Provo, Utah, USA) and were asked to replicate a similar intake before post‐testing, including consuming the same carbohydrate‐rich snack/meal and hydration fluid. All testing was administered by qualified exercise professionals, and assessors were familiarised with protocols as a group to minimise inter and intratester variability. Baseline testing procedures were repeated at post‐testing (Figure 1).

#### 2.2.1 Anthropometry

Standing height was recorded using a wall mounted stadiometre (Holtain Limited, Crymmych, Pembs, United Kingdom). Participants removed footwear and stood in the anatomical position with a standardised foot placement and the lower eyelids aligned with the auricular lobes. Body mass was measured using a calibrated electronic scale with participants in minimal clothing (Kunshan Lightlever, Kunshan City, China). The resulting measurements were used to calculate body mass index (BMI). Waist and hip circumference were measured as per previous recommendations (World Health Organization 2008; National Heart, Lung and Blood Institute, North American Association for the Study of Ovesity 2000).

#### 2.2.2 Body Composition

Provided no X‐ray or nuclear medicine scans were performed in the preceding 7 days, participants underwent a whole‐body gold standard (Dual Energy X‐ray Absorptiometry) DEXA scan to assess fat mass (FM), lean mass (LM) and bone mineral content (BMC) (Lunar Prodigy, GE Medical, Milwaukee, USA) (Shiel et al. 2017). The Lunar Prodigy has demonstrated valid and reliable FM, LM and BMC measurements compared with two‐ and three‐compartment models (Glickman et al. 2004; Norcross and Van Loan 2004). The machine was calibrated before each testing round per manufacturer guidelines. Participants provided additional informed consent and completed mandatory screening before each baseline and post‐testing scan. All scans were conducted by a licenced professional following the Australian High Performance Sport System DEXA Best Practice Guidelines (Australian Institute of Sport 2022). Participants presented in a euhydrated, fasted and rested state. Custom‐made positioning aids were utilised to ensure consistency in positioning. The foam blocks were made of Styrofoam, making them transparent under DEXA (Nana et al. 2012). DEXA protocols met compliance requirements for ionising radiation apparatus used in diagnostic imaging (Radiation Protection Series No. 8) (New South Wales Environment Protection Authority 2004).

#### 2.2.3 Maximal Strength

Following a 60‐min recovery period to allow rehydration and food intake, upper‐ and lower‐body maximal strength were assessed via one‐repetition maximum (1RM) testing in the barbell parallel back squat (1RM_squat_) and flat barbell bench press (1RM_bench_). These protocols are well‐established measures of maximal strength (Schoenfeld et al. 2019; Grgic et al. 2020). Testing was conducted by qualified exercise professionals in an environmentally controlled resistance‐training room, following ACSM guidelines (American College of Sports Medicine 2025). Participants completed a general warm‐up consisting of 5 min of cycling at 50W on a Wattbike Pro (Nottingham, UK) and two sets of 10–12 repetitions of dynamic exercises (bodyweight squat, glute bridge and push‐up). Participants could perform additional dynamic movements for up to 3 min. Assessors demonstrated the squat set‐up and technique, after which participants completed a bar‐only familiarisation set (8 kg or 20 kg depending on perceived strength) for 8–10 repetitions. Warm‐up sets included 8–10 repetitions at ∼50% perceived 1RM, followed by 2–3 repetitions at ∼60% 1RM and 2–3 repetitions at ∼80% 1RM. Participants then performed single repetitions with progressively increasing loads (∼5% increments or as judged appropriate by the assessor) to determine 1RM, with 3–5 min of seated rest between attempts. Failure on two consecutive attempts resulted in the previous successful load being recorded as the 1RM (American College of Sports Medicine 2025). After ≥ 5 min rest, the same procedure was repeated for the barbell bench press (excluding the aerobic warm‐up). All attempts and associated RPE (CR‐10) were recorded.

#### 2.2.4 Aerobic Fitness

Following 30 min rest after 1RM testing, aerobic fitness was assessed using a graded exercise test (GXT) on a cycle ergometer (SRM, Jülich, Germany). Participants began at 50W, and power output increased by 25W each minute until volitional exhaustion. Power output (W) and cadence were recorded throughout. Maximal aerobic power (Work_max_) was defined as the average W for the last completed stage of the test. The mean of the highest three consecutive periods (10 s) of oxygen consumption was used to determine $\dot{V}$O_2peak_. Heart rate (HR) was recorded throughout the test. The highest HR observed was taken as maximal HR (HR_max_) (FT7, Polar Electro, Kempele, Finland). RPE was recorded at test completion. Oxygen consumption was determined by measuring oxygen and carbon dioxide concentrations with a metabolic gas analyser (Medgraphics Ultima System, Saint Paul, USA). The metabolic cart was calibrated according to the manufacturer's instructions, involving a pneumotachometer calibration via a 3‐L syringe, analysis of ambient air and gas calibration with a gravimetric gas mixture of known concentrations [CO_2_ 4.1 (0.1) % and O_2_ 15.7 (0.2) %] (Allen et al. 2017).

#### 2.2.5 Exercise Familiarisation

After the aerobic fitness assessment, participants completed a brief familiarisation session (∼10–15 min) with an intervention instructor. Exercises included the Romanian deadlift, goblet squat, dumbbell chest press, barbell bent‐over row, cycle ergometer (Wattbike, Nottingham, UK; Concept2 BikeErg, Vermont, USA), rowing ergometer (Concept2 RowErg, Vermont, USA), aerobic step‐up and shuttle run. This session familiarised participants with exercise technique, the prescribed training intensity (≥ 7 RPE) and allowed estimation of appropriate external loads for training sessions. These loads were recorded and provided to participants at the subsequent three familiarisation sessions. After at least 24 h of recovery, participants completed three 45‐min familiarisation sessions (HIMT, aerobic training [AT] and resistance training [RT]) in a randomised order. Participants were instructed to arrive appropriately fuelled and hydrated for high‐intensity exercise (carbohydrate‐rich meal/snack 1–2 h prior and adequate hydration). These sessions familiarised participants with exercises and equipment for week 1 of training, training locations, instructors and data‐collection procedures. Full details of these sessions are available on the OSF.

#### 2.2.6 Training Load Completed

During each training session, participants recorded repetitions, weight lifted, distance and ergometer resistance in a paper training diary. These diaries allowed participants to track progress and apply progressive overload. Heart rate (HR) was continuously monitored during all familiarisation and training sessions (FT7, Polar Electro 2024, Kempele, Finland). Participants could view their HR on an iPad, although HR was not used to prescribe or regulate exercise intensity. To ensure no additional high‐intensity training occurred outside the intervention, participants reported any external physical activity each week using the Godin Leisure‐Time Physical Activity Questionnaire via an online survey (Godin 2011).

### 2.3 Training Intervention

Following baseline testing, participants were stratified by sex and randomly allocated into either the HIMT group or ISCT group at a 1:1 ratio. The HIMT group performed 4 × 45‐min HIMT sessions per week (Figure 1, Table 1) for 7‐weeks. The rationale for this frequency was to enable participants meet physical activity guidelines across both training modes and promote appropriate recovery between sessions. HIMT session intensity was prescribed as ≥ 7 RPE (Bishop et al. 2025). Exercise intensity was monitored during session delivery using RPE (i.e., RPE ≥ 7) or repetitions in reserve (RIR) (i.e., ≤ 3 RIR) for resistance exercises (Bishop et al. 2025; Zourdos et al. 2016). All resistance‐based exercise sets throughout the HIMT programme were prescribed as 10 reps to standardise and match prescribed volume‐load between groups (i.e., sets × reps). Exercise selection and work: relief ratios varied fortnightly to sustain variety and apply progressive overload. The ISCT group performed 2 × AT and 2 × RT sessions per week (Figure 1, Table 1). The ISCT programme was matched to the HIMT programme for prescribed weekly exercise selection, intensity (i.e., ≥ 7 RPE) and volume (i.e., interval duration and work: relief, sets and reps). Therefore, the only distinguishing prescriptive variable between the HIMT and ISCT groups was exercise mode format (i.e., combined vs. separate aerobic and resistance exercise within vs. between a session) (Table 1). All training sessions were conducted in small groups (3–8 participants: instructor). In the event more than 8 participants were scheduled into a session, an additional instructor was involved in session delivery. The complete details of the prescribed and delivered training intervention have been reported previously (Sharp et al. 2026).

**TABLE 1:** Example exercise order of training sessions performed in a training week.

Group	HIMT	ISCT	
Session type	HIMT	Resistance training	Aerobic training
Exercise 1	Romanian deadlift	Romanian deadlift	Bike ergometer
Exercise 2	Bike ergometer	Barbell bent over row	Aerobic stepper
Exercise 3	Barbell bent over row	Goblet squat	Rower ergometer
Exercise 4	Aerobic stepper	Flat chest press	Shuttle run
Exercise 5	Goblet squat	Romanian deadlift	Bike ergometer
Exercise 6	Rower ergometer	Barbell bent over row	Aerobic stepper
Exercise 7	Flat chest press	Goblet squat	Rower ergometer
Exercise 8	Shuttle run	Flat chest press	Shuttle run

Abbreviations: HIMT, high‐intensity multimodal training; ISCT, inter‐session concurrent training.

### 2.4 Statistical Analysis

An intention to treat analysis using a linear mixed effects regression (using maximum likelihood estimation) was conducted to examine change over time and group × time interactions (2 × 2) for maximal strength, aerobic fitness, body composition and anthropometry. For each outcome measure, a separate regression model was developed with age, sex, baseline, and location specified as fixed effects, the main timepoint:group interaction effect and random intercepts were specified for each participant ID. Sex and age were included as covariates within the model; however, as they were not the primary variables of interest, differences between sexes and ages were not interpreted. To address potential clustering effects of participant training environments, training location was initially included as a random effect, however, given that models did not converge likely due to relatively few locations and additional model complexity, the variable was retained as a fixed effect. Statistical analysis was conducted in R (v4.4.3, R Core Team, Vienna, Austria) in RStudio (v2024.9.1.394, Posit Team, Boston, MA) via the glmmTMB package (v1.1.11, Brooks et al. 2017). The residuals of each model were inspected via Q–Q plot and plots of the residuals versus fitted values. Statistical significance was set at *p* < 0.05; however, given that sixteen different outcome variables were analysed, findings have also been reported in light of a Bonferroni‐corrected value of *p* < 0.003. 95% confidence intervals were used to estimate the precision of effect estimates. Effect sizes for mixed‐effects model contrasts were calculated as standardised mean differences (Cohen's *d*) with thresholds interpreted as small (0.2), medium (0.5) and large (0.8) (Cohen 1988). The complete statistical analysis is available in Supporting Information S1. A sensitivity analysis was also conducted to compare those that adhered to the programme (i.e., ≥ 80% session attendance) with those that did not. This is available in Supporting Information S2.

## 3 Results

**FIGURE 2:**
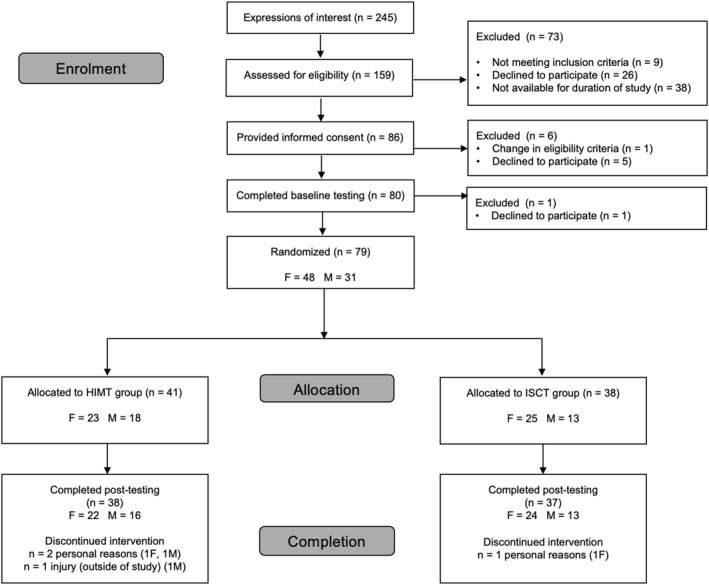
Study flow diagram. F, female; HIMT, high‐intensity multimodal training; ISCT, inter‐session concurrent training; M, male; *n*, number.

Figure 2 outlines the flow of participants through the study. Participants' baseline characteristics are presented descriptively in Table 2. As participants were randomised, no statistical tests were performed to assess baseline differences as per CONSORT and methodological recommendations (Bland and Altman 2011). Training session attendance and completion data are reported elsewhere (Sharp et al. 2026). No differences in training session intensity (i.e., S‐RPE) were observed between groups.

**TABLE 2:** Participant baseline characteristics.

Characteristics, mean ± SD	HIMT (*n* = 41)	ISCT (*n* = 38)	Total (*n* = 79)
Age (y)	35.8 ± 11.7	37.0 ± 12.5	36.3 ± 11.9
Female	23	25	48
Male	18	13	31
Body mass (kg)	71.4 ± 11.7	73.2 ± 13.9	72.1 ± 12.7
Height (cm)	169.0 ± 8.9	170.6 ± 8.2	169.7 ± 8.6
BMI (kg/m^2^)	24.9 ± 2.9	25.1 ± 4.4	25.0 ± 3.7
Training location			
1	23	18	41
2	7	10	17
3	11	10	21

Abbreviations: BMI, body mass index; cm, centimetres; HIMT, high‐intensity multimodal training; ISCT, inter‐session concurrent training; kg, kilogrammes; m^2^, metres squared; *n*, number; SD, standard deviation y, years.

**FIGURE 3:**
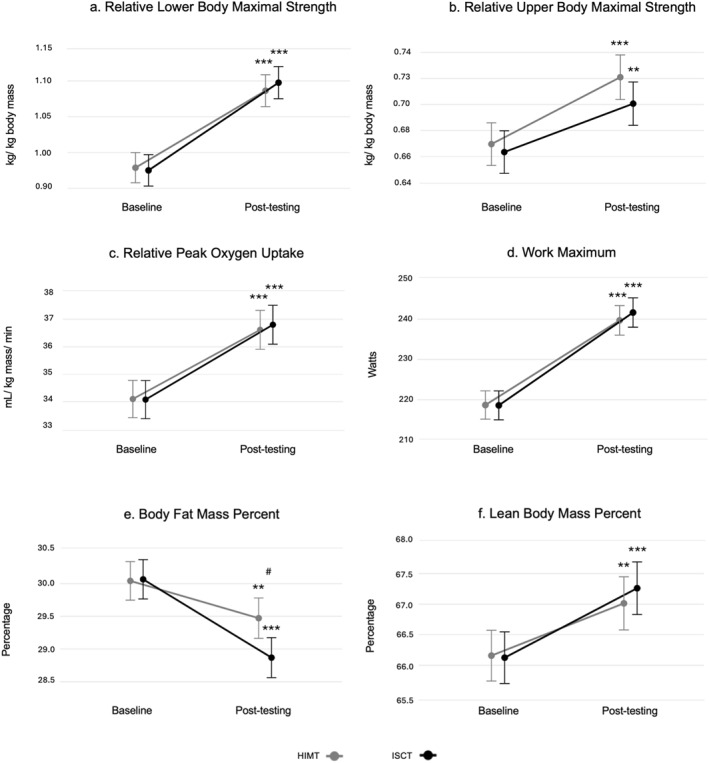
Physical outcome changes from baseline to post‐testing, (3a) relative lower body maximal strength, (3b) relative upper body maximal strength, (3c) relative peak oxygen uptake, (3d) work maximum, (3e) body fat mass percent, (3f) lean body mass percent. HIMT, high‐intensity multimodal training; ISCT, inter‐session concurrent training; kg, kilogramme; mL, millilitre; min, minute; *, significant within group effect; ** (*p* < 0.01), *** (*p* < 0.001); #, significant between group effect (*p* < 0.05).

Twelve participants (*n* = 5 HIMT and *n* = 7 ISCT), were excluded from the 1RM_squat_ analysis due to adaptation of the test (i.e., 3RM completion due to safety concerns from the interventionist) (*n* = 3 HIMT and *n* = 5 ISCT) or‐self termination of the test (*n* = 2 HIMT and *n* = 2 ISCT). The data of the remaining 67 participants are presented in the 1RM_squat_ measures. Both the HIMT and ISCT groups demonstrated small within‐group time effects for relative lower body maximal strength (HIMT: *b* = 0.107, 95% CI [0.079–0.136], *p* < 0.001 and *d* = 0.397 and ISCT: *b* = 0.122, 95% CI [0.092–0.153], *p* < 0.001 and *d* = 0.452) (Figure 3 and Table 3). Both groups demonstrated trivial within‐group time effects for upper body maximal strength (HIMT: *b* = 0.051, 95% CI [0.029–0.074], *p* < 0.001 and *d* = 0.195 and ISCT: *b* = 0.037, 95% CI [0.015–0.060], *p* < 0.001 and *d* = 0.141) with no group × time interactions observed (*p* > 0.05) (Figure 3 and Table 2). Similarly, both groups demonstrated small within‐group time effects for $\dot{V}$O_2peak_ (HIMT: *b* = 2.517, 95% CI [1.577–3.457], *p* < 0.001 and *d* = 0.303 and ISCT: *b* = 2.724, 95% CI [1.760–3.689], *p* < 0.001 and *d* = 0.328) and Work_max_ (HIMT: *b* = 21.016, 95% CI [16.174–25.858], *p* < 0.001 and *d* = 0.362 and ISCT: *b* = 23.049, 95% CI [18.085–28.014] *p* < 0.001 and *d* = 0.397) with no group × time interactions (*p* > 0.05) (Figure 3, Table 3). No within or between‐group differences were observed for HR_max_ (*p* > 0.05) (Table 3).

**TABLE 3:** Maximal strength and aerobic fitness outcomes.

Group	HIMT							ISCT							Group × time point comparison				
Outcome	Baseline (mean ± SD)	Post‐testing (mean ± SD)	Estimate	Lower CL	Upper CL	*p*‐value	Effect size (*d*)	Baseline (mean ± SD)	Post‐testing (mean ± SD)	Estimate	Lower CL	Upper CL	*p*‐value	Effect size (*d*)	Estimate	Lower CL	Upper CL	*p*‐value	Effect size (*d*)
1RM squat (kg/kg body mass)	0.99 ± 0.29	1.11 ± 0.28***	0.107	0.079	0.136	< 0.001	0.397	0.94 ± 0.25	1.07 ± 0.23***	0.122	0.092	0.153	< 0.001	0.452	0.015	−0.027	0.056	0.478	0.055
1RM bench press (kg/kg body mass)	0.72 ± 0.29	0.78 ± 0.29***	0.051	0.029	0.074	< 0.001	0.195	0.61 ± 0.22	0.64 ± 0.21**	0.037	0.015	0.060	0.002	0.141	−0.014	−0.046	0.017	0.375	−0.054
$\dot{V}$O_2peak_ (mL/kg/min)	34.13 ± 7.8	36.77 ± 7.85***	2.517	1.577	3.457	< 0.001	0.303	33.84 ± 8.7	36.48 ± 8.74***	2.724	1.760	3.689	< 0.001	0.328	0.207	−1.139	1.554	0.761	0.025
Work_max_ (watts)	218.84 ± 56.94	242.78 ± 58.32**	21.016	16.174	25.858	< 0.001	0.362	214.67 ± 55.87	237.09 ± 58.76**	23.049	18.085	28.014	< 0.001	0.397	2.033	−4.901	8.996	0.563	0.035
HR_max_ (achieved in GXT)	174.6 ± 12.6	172.4 ± 25.6	−2.299	−7.431	2.832	0.377	−0.133	175.5 ± 14.1	174.1 ± 14.8	−1.570	−6.832	3.692	0.556	−0.091	0.73	−6.619	8.078	0.845	0.042

Abbreviations: 1RM, one repetition maximum; GXT, graded exercise test; HIMT, high‐intensity multimodal training; HR_max_, heart rate maximum; ISCT, inter‐session concurrent training; kg, kilogrammes; L, litres; min, minutes; ml, millilitre; $\dot{V}$O_2peak_, peak oxygen uptake; Work_max_, maximum power output achieved in final stage of graded exercise test.

^a^ Within‐group × time effect.

^**^ *p* < 0.01.

^***^ *p* < 0.001.

Both the HIMT and ISCT groups demonstrated trivial within‐groups effects for fat mass (HIMT: *b* = −0.405, 95% CI [−0.769 to −0.042], *p* = 0.029 and *d* = −0.054 and ISCT: *b* = −0.852, 95% CI [−1.225 to −0.479], *p* < 0.001 and *d* = −0.113), lean mass (HIMT: *b* = 0.745, 95% CI [0.402–1.089], *p* < 0.001 and *d* = 0.079 and ISCT: *b* = 0.737, 95% CI [0.385–1.090], *p* < 0.001 and *d* = 0.078), body fat percentage (HIMT: *b* = −0.570, 95% CI [−0.976 to −0.163], *p* = 0.006 and *d* = −0.075 and ISCT: *b* = −1.198, 95% CI [−1.615 to −0.781], *p* < 0.001 and *d* = −0.157), lean mass percentage (HIMT: *b* = 0.855, 95% CI [0.283–1.427], *p* = 0.004 and *d* = 0.115 and ISCT: *b* = 1.135, 95% CI [0.548–1.722], *p* < 0.001 and *d* = 0.153), waist circumference (HIMT: *b* = −1.175, 95% CI [−1.714 to −0.637] *p* < 0.001 and *d* = −0.122 and ISCT: *b* = −0.818, 95% CI [−1.370 to −0.266], *p* = 0.004 and *d* = −0.085) and WHR (HIMT: *b* = −1.380, 95% CI [−0.01926 to −0.00834] *p* < 0.001 and *d* = −0.190 and ISCT: *b* = −0.711, 95% CI [−0.01271 to −0.00151], *p* = 0.013 and *d* = −0.098) (Figure 3 and Table 3). A trivial significant group × time interaction was observed for fat mass percentage in favour of the ISCT group (*b* = −0.628, 95% CI [−1.21 to −0.046], *p* = 0.035 and *d* = −0.082) (Figure 3 and Table 4). No between group differences were observed for body mass, BMI, hip circumference and bone mass (*p* > 0.05) (Table 4). The complete analysis is available in Supporting Information S1. A sensitivity analysis (i.e., including only participants who achieved 80% session attendance) produced results consistent with the primary intention to treat analysis, with no material differences in estimates, confidence intervals or *p*‐values. This analysis is available in Supporting Information S2.

**TABLE 4:** Anthropometry and body composition outcomes.

Group	HIMT							ISCT							Group × time point comparison				
Outcome	Baseline (mean ± SD)	Post‐testing (mean ± SD)	Estimate	Lower CL	Upper CL	*p*‐value	Effect size (*d*)	Baseline (mean ± SD)	Post‐testing (mean ± SD)	Estimate	Lower CL	Upper CL	*p*‐value	Effect size (d)	Estimate	Lower CL	Upper CL	*p*‐value	Effect size (*d*)
Body mass (kg)	71.4 ± 11.66	72.29 ± 11.47	0.122	−0.346	0.590	0.607	0.010	73.21 ± 13.88	73.11 ± 14.26	−0.169	−0.648	0.311	0.488	−0.013	−0.291	−0.961	0.379	0.393	−0.023
BMI (kg/m^2^)	24.9 ± 2.92	24.98 ± 2.82	−0.032	−0.201	0.137	0.707	−0.009	25.14 ± 4.44	25.08 ± 4.47	−0.112	−0.285	0.061	0.203	−0.030	−0.08	−0.322	0.162	0.515	−0.022
Waist circumference (cm)	79.74 ± 8.04	78.91 ± 8.09***	−1.175	−1.714	−0.637	< 0.001	−0.122	80.89 ± 11.22	80.09 ± 11.12**	−0.818	−1.370	−0.266	0.004	−0.085	0.357	−0.414	1.128	0.362	0.037
Hip circumference (cm)	100.64 ± 5.76	101.1 ± 5.75	0.307	−0.185	0.799	0.220	0.043	101.42 ± 8.61	101.32 ± 8.55	−0.180	−0.684	0.325	0.483	−0.025	−0.486	−1.191	0.218	0.174	−0.068
W:H	0.79 ± 0.06	0.78 ± 0.07***	−1.380	−0.01926	−0.00834	< 0.001	−0.190	0.80 ± 0.08	0.79 ± 0.08*	−0.711	−0.01271	−0.00151	0.013	−0.098	0.669	−0.00113	0.01451	0.093	0.092
Bone mass (kg)	2.6 ± 0.47	2.62 ± 0.48	0.003	−0.009	0.014	0.626	0.006	2.66 ± 0.45	2.65 ± 0.47	−0.002	−0.014	0.010	0.754	−0.004	−0.005	−0.021	0.012	0.573	−0.01
Fat mass (kg)	20.99 ± 6.42	20.73 ± 6.38*	−0.405	−0.769	−0.042	0.029	−0.054	22.78 ± 8.67	22.05 ± 8.62***	−0.852	−1.225	−0.479	< 0.001	−0.113	−0.447	−0.968	0.074	0.092	−0.059
Lean mass (kg)	47.62 ± 9.99	48.98 ± 10.16***	0.745	0.402	1.089	< 0.001	0.079	47.5 ± 8.75	48.18 ± 9.21***	0.737	0.385	1.090	< 0.001	0.078	−0.008	−0.5	0.484	0.974	−0.001
Body fat percentage (%)	29.49 ± 7.64	28.78 ± 7.61**	−0.570	−0.976	−0.163	0.006	−0.075	30.82 ± 7.75	29.75 ± 7.71*** ^,^ ^a^	−1.198	−1.615	−0.781	< 0.001	−0.157	−0.628	−1.21	−0.046	0.035	−0.082
Lean mass percentage (%)	66.61 ± 7.38	67.63 ± 7.41**	0.855	0.283	1.427	0.004	0.115	65.38 ± 7.51	66.39 ± 7.5***	1.135	0.548	1.722	< 0.001	0.153	0.28	−0.539	1.1	0.5	0.038

Abbreviations: %, percentage; BMI, body mass index; cm, centimetres; HIMT, high‐intensity multimodal training; ISCT, inter‐session concurrent training; kg, kilogramme, m^2^, metres squared W:H, waist‐to‐hip ratio.

^a^ Between‐group × time effect *p* < 0.001.

^*^ Within‐group × time effect.

^**^ *p* < 0.01.

^***^ *p* < 0.001.

## 4 Discussion

The present study examined the effects of 7‐weeks of HIMT compared with a matched active control group (ISCT) on physical health and fitness outcomes in recreationally active adults. Both training formats elicited comparable improvements in muscular strength, aerobic fitness, body composition and anthropometric measures. Although the magnitude of these adaptations was small, the consistency of responses across outcomes suggests that HIMT and ISCT can promote favourable physiological adaptations in recreationally active individuals. Collectively, these findings indicate that the observed adaptations were more strongly related to overall training exposure (given that prescribed volume was matched) rather than training structure or exercise order (i.e., combined vs. separate high‐intensity aerobic and resistance training).

### 4.1 Muscular Strength

Both groups demonstrated significant but small improvements in maximal lower and upper body strength without differences between groups. Although the magnitude of change was modest, this may be expected given the recreationally active status of participants who likely present a reduced capacity for rapid strength adaptation compared with untrained populations (American College of Sports Medicine 2025). In contrast, studies reporting moderate‐to‐large effects have predominantly been conducted in sedentary cohorts and/or without matched‐load control conditions limiting direct comparison (American College of Sports Medicine 2025; Batrakoulis et al. 2018; Currier et al. 2023; Romero‐Arenas et al. 2018; McWeeny et al. 2020; Schumann et al. 2022). Within the context of controlled, load‐matched training in an already active population, these findings suggest HIMT can elicit meaningful but conservative strength adaptations. The comparable improvements in strength between groups likely reflects the weekly matched prescribed training volume‐load. Although the HIMT group performed RT more frequently compared to the ISCT group (i.e., four vs. twice per week), the total weekly prescribed volume‐load was the same, aligning with the understanding that strength improvements are driven by volume rather than frequency (Grgic et al. 2018). Additionally, the compound exercises prescribed in the HIMT and ISCT programmes (e.g., Romanian deadlift and dumbbell bench press) involved substantial muscle mass recruitment during training, likely contributing to the observed strength outcomes.

Importantly, combining resistance and aerobic exercise within a session in the HIMT programme did not appear to compromise strength gains across a 7‐week period in recreationally active adults. These findings contrast with previous research examining the interference effect in within‐session concurrent training (i.e., combined aerobic and resistance training) (Vechin et al. 2021; Eddens et al. 2018). In particular, recent work in concurrent training suggests that longer work periods (i.e., > 60 s at 95%–100% maximal aerobic power) and lighter external loads during resistance exercise (i.e., > 10 repetition maximum [RM]) can negatively impact strength adaptations (Vechin et al. 2021). Notably, the present intervention prescribed aerobic intervals (i.e., work periods < 60 s) and resistance loads (i.e., 10RM) outside of these ranges, which may partly explain the absence of reduced strength gains in the HIMT group. However, without direct examination of the molecular or hormonal pathways that underpin the interference effect, this interpretation of the findings remains speculative. Individual training status may also provide context for the current findings. Although resistance and aerobic exercise are known to activate distinct signalling pathways, novice individuals may exhibit less specialised adaptive responses, potentially contributing to a more homogenous training stimulus (Fyfe and Loenneke 2018). Despite the eligibility criteria of this study (i.e., recreationally active), the diverse training status of participants must be considered. For example, some participants had not previously participated in the specific resistance exercises or training intensity prescribed within the current programme. This may have contributed to more rapid initial adaptations for these HIMT participants and may have masked any subtle interference effects (Fyfe and Loenneke 2018). Therefore, a longer training duration may have demonstrated more modest strength outcomes as the training age of participants matured.

Given the current complex understanding of the interference effect in concurrent training (i.e., contributing biochemical mechanisms, individual factors and perceptual responses to exercise), it remains unclear whether the observed strength outcomes were primarily driven by the prescribed training format (i.e., HIMT vs. ISCT) or other underlying factors. Future research would benefit from further investigations of the acute and/or chronic anabolic hormonal (i.e., growth hormone and testosterone) and molecular signalling pathways (i.e., AMP‐activated protein kinase [AMPK] and mTOR pathways) in HIMT to better understand the biochemical mechanisms underpinning the proposed interference effect.

### 4.2 Aerobic Fitness

Participation in 7‐weeks of HIMT and ISCT resulted in statistically significant but small improvements in aerobic fitness (i.e., $\dot{V}$O_2peak_ and Work_max_) in recreationally active adults without between‐group differences. These adaptations are likely attributed to the high‐intensity aerobic workloads prescribed (i.e., RPE ≥ 7), whole body movements and interval type prescription of the programmes (Kilpatrick et al. 2014; Batrakoulis et al. 2021). However, the modest magnitude of change may reflect the recreationally active status of participants who typically exhibit blunted improvements in aerobic fitness compared with untrained populations. Although this is broadly consistent with previous HIMT and high‐intensity aerobic training studies, many of these have been conducted in sedentary cohorts or demonstrate poor methodological rigour, limiting direct comparisons across studies (Ramos‐Campo et al. 2021; Schmidt et al. 2016; Ajjimaporn et al. 2019; Hesketh et al. 2021; Buckley et al. 2015). It is likely that the ‘short’ interval prescription used in the HIMT and ISCT training sessions (i.e., 30–60 s work: 15–30 s rest) promoted an accumulation of time at or near $\dot{V}$O_2max_, driving the observed adaptations in $\dot{V}$O_2peak_ (Buchheit and Laursen 2013; Gibala et al. 2012). The minimal rest periods (i.e., transitions between exercises) in both groups may have also promoted a sustained elevation in heart rate (HR), eliciting a cumulative aerobic stimulus. HIMT has been previously shown to stimulate the AMPK/PGC‐1α pathway which is associated with mitochondrial biogenesis and other central and peripheral aerobic adaptations that are comparable to those well understood in HIIT (i.e., ISCT aerobic sessions) (Fyfe et al. 2014; Ben‐Zeev and Okun 2021). Furthermore, the sequenced combination of exercise modalities with reduced rest in HIMT may have contributed to an ‘interaction’ effect, whereby greater physiological work and central or peripheral fatigue was accumulated during a session compared to continuous single mode aerobic training (i.e., ergometry only) (Carroll et al. 2016). However, the alternate aerobic and resistance exercise (with minimal rest) in HIMT may have instead contributed to greater perceived cumulative fatigue reducing the subjective training quality and intensity of effort during sessions (i.e., perceived RPE remained at the prescribed intensity, whereas actual work [repetitions and distance] reduced and/or training quality was reduced) (Senna et al. 2011; Gavanda et al. 2022; Shell et al. 2023). Nevertheless, both HIMT and ISCT promoted similar positive outcomes for aerobic fitness over a 7‐week training period in recreationally active adults.

### 4.3 Body Composition and Anthropometry

Both the HIMT and ISCT groups demonstrated small but significant improvements in body composition following the 7‐week intervention with no meaningful differences between groups. Although changes were significant, effect sizes were trivial, indicating limited practical impact. Moreover, body mass in both groups remained stable from pre to post intervention (Table 4). Although previous HIMT studies in recreationally active adults have reported moderate improvements in body composition, these interventions were generally of longer duration and/or employed disparate training prescriptions limiting direct comparisons (Ramos‐Campo et al. 2021; Feito et al. 2018; Kapsis et al. 2022; Posnakidis et al. 2022; Murawska‐Cialowicz et al. 2015). Accordingly, the smaller effects observed in the present study may reflect both the shorter intervention period. The comparable outcomes between groups (HIMT and ISCT) are likely explained by the matched prescribed weekly training volume and the use of whole‐body movements, which likely promoted a sufficient level of energy expenditure to support modest changes in body composition. Although the ISCT group demonstrated slightly greater reductions in body fat percentage, this should be interpreted cautiously given the short intervention duration and trivial effect sizes. In addition to reductions in body fat, both the HIMT and ISCT groups demonstrated comparable increases in lean muscle mass and lean muscle percentage following the 7‐week intervention. These findings align with the matched prescribed resistance exercise dose between groups. The use of compound exercises (e.g., goblet squat and Romanian deadlift) in training likely promoted greater muscle mass recruitment compared to the prescription of accessory exercises only (e.g., bicep curl). Additionally, all resistance exercises were prescribed in a range conducive to muscle hypertrophy (i.e., 8–12 reps at 70%–85% 1RM), which may explain the demonstrated increases in lean body mass (American College of Sports Medicine 2025). However, it is acknowledged that DEXA derived changes in lean body mass are an indirect measure of hypertrophy as lean mass may reflect additional factors (e.g., fluid retention and glycogen storage). Both groups also demonstrated favourable but trivial changes in waist circumference and WHR. Maintenance of hip circumference in both groups may reflect the preservation of muscle mass around the gynoid and lower body region due to the prescribed resistance training volume in both groups. The observed reduction in WHR may further support this interpretation. Overall, the findings suggest that both training approaches (i.e., HIMT and ISCT) can produce modest improvements in body composition over 7‐weeks in recreationally active adults. However, the magnitude of these changes is likely limited within a short intervention period.

## 5 Practical Applications

Participation in 7‐weeks of HIMT and ISCT revealed small comparable improvements in maximal strength, aerobic fitness and trivial changes in body composition in recreationally active adults. These findings suggest that strength adaptations are more closely related to the total training volume prescribed rather than the specific exercise format when prescribed in alignment with this intervention. Accordingly, HIMT appears to be an effective approach for eliciting physical health and performance adaptations within this population. Despite the understanding that low‐volume or very high‐load prescription (> 75% 1RM) aligns with greater magnitudes of strength adaptations, the findings of the present study suggest recreationally active populations may still achieve strength adaptations following a more modest high‐intensity prescription (≥ 7 RPE, 10RM and corresponding to ∼75% 1RM) (Currier et al. 2023; Bishop et al. 2025; Schoenfeld et al. 2017). In community gym‐based settings, this prescription may provide a more feasible approach compared to traditional very‐high intensity prescriptions for strength adaptations. Similarly, the interval, rest and intensity prescription used in this study may support cardiorespiratory health outcomes even if the aerobic adaptations were modest.

### 5.1 Strengths and Limitations

A key strength of this study was the matched prescribed training dose between groups. By equating total prescribed weekly training volume, intensity and exercise selection, any differences in outcomes can be attributed to the training format (i.e., combined vs. separate sessions) rather than discrepancies in exercise prescription. Another strength was the use of RPE and RIR to prescribe and monitor training intensity as these subjective metrics may more appropriately capture the combined training stimulus of HIMT compared to %HR_max_ or %1RM. Although the prescribed intensity for HIMT and ISCT did reflect a ‘higher’ load of resistance (i.e., ∼75% 1RM), some participants may not have achieved this consistently due to interindividual variability in RPE and RIR, potentially affecting external load in resistance exercises or effort in aerobic work. To minimise this variability, participants completed an anchoring exercise at baseline and were further familiarised across three exercise familiarisation sessions before randomisation. Additionally, the large sample size represents a key strength of the study, with the anticipated attrition rate not observed, thereby increasing the statistical power of the findings (Collins et al. 2022). The pragmatic community‐based design further enhances ecological validity, although location‐level variation in delivery may be perceived as a limitation. Such variation is inherent to pragmatic trials and reflects real‐world implementation, thereby increasing the practical relevance of the findings.

Observation of nutritional intake, alcohol consumption and energy expenditure was beyond the scope of this investigation and was not controlled for. Therefore, the body composition outcomes of this study should be interpreted with caution given these factors may have impacted the findings. However, participants did receive educational resources about fuelling prior to high‐intensity exercise (e.g., carbohydrate and fluid intake). Although participants were asked to refrain from additional high‐intensity activity and reported low‐to‐moderate activity weekly (Godin LTPAQ), external exercise could not be fully controlled (Godin 2011). The specificity of 1RM testing must also be acknowledged as a limitation, wherein previous research suggests training at a high %1RM may contribute to greater familiarity in 1RM testing. To reduce familiarisation and learning effects in testing, training sessions were not prescribed at this maximal intensity for this reason. However, this may have reduced the specificity of the training for maximal strength adaptations. Furthermore, this training programme was prescribed as a circuit format where HIMT sessions involved the strict alternation of aerobic and resistances exercises. Given the inherent variability associated with the numerous styles of HIMT (exercise order, selection, volume and intensity), these findings may not generalise to other styles (e.g., CrossFit and bootcamps). In particular, the occurrence of the interference effect is suggested to be dependent on exercise intensity, volume and exercise selection, therefore limiting the translation of these findings to circuit‐based HIMT only (Vechin et al. 2021; Eddens et al. 2018; Fyfe and Loenneke 2018). Similarly, the ISCT group findings are limited to a controlled circuit format, which may not reflect common methods of concurrent training in the community (e.g., prescribed sets × repetitions of RT with longer rest periods and blocked AT modalities). Moreover, findings apply only to a 7‐week intervention in recreationally active adults. This duration may favour individuals with lower resistance‐training age (i.e., novices), whereas experienced participants may require longer exposure to avoid plateaus. (i.e., baseline activity levels of the participants may have precluded greater magnitudes of physical adaptations). Finally, due to the number of outcome variables involved (*n* = 16) in the linear mixed effects regression model, findings were reported against both a significance value of *p* < 0.05 and *p* < 0.003 (i.e., Bonferri correction). This conservative approach increases the likelihood of type II error; therefore, results for waist circumference and waist‐to‐hip ratio in ISCT and fat mass, fat‐mass percentage and lean‐mass percentage in HIMT should be interpreted cautiously. The group × time interaction for fat‐mass percentage also fell below the adjusted threshold warranting cautious interpretation.

## 6 Conclusion

The findings of this study demonstrate that HIMT is an effective strategy for improving physical health and performance outcomes in recreationally active adults. Separating high‐intensity aerobic and resistance exercise on different training days may provide a modest advantage for reducing body fat percentage. However, gains in lean mass were comparable between HIMT and ISCT formats. Despite the positive physical outcomes observed, the absence of notable between‐group differences, coupled with the complex understanding of the interference effect, makes it difficult to determine whether the observed outcomes were underpinned by the prescribed training format (i.e., HIMT vs. ISCT) or other associated factors such as biochemical mechanisms or perceived responses to exercise. Therefore, future research should seek to more clearly understand these biochemical and perceptual responses to HIMT better understand the mechanisms of the interference and/or interaction effect. This may facilitate more targeted goal‐based service delivery in the community and contribute to sustainable physical activity behaviours.

**Abbreviations**
ACSM: American College of Sports Medicine
AMPK: AMP‐activated protein kinase
AT: aerobic training
BMC: bone mineral content
BMI: body mass index
CERT: Consensus on Exercise Reporting Template
cm: centimetres
CONSORT: CONsolidated Standards of Reporting Trials
CT: concurrent training
DEXA: dual energy X‐ray absorptiometry
F: female
FM: fat mass
GXT: graded exercise test
HIIT: high‐intensity interval training
HIMT: high‐intensity multimodal training
HR: heart rate
HR_max_
: heart rate maximum
ISCT: inter‐session concurrent training
kg: kilogrammes
L: litres
LM: lean mass
LTPAQ: Leisure Time Physical Activity Questionnaire
M: male
m^2^
: metres squared
min: minutes
mL: millilitres
mTOR: mechanistic target of rapamycin
*n*
: number
PGC‐1 *α*
: peroxisome proliferator‐activated receptor gamma coactivator 1‐alpha
RM: repetition maximum
RPE: rating of perceived exertion
RT: resistance training
S‐RPE: session rating of perceived exertion
W: watts
W:H: waist‐to‐hip ratio
Work_max_
: maximal aerobic power
y: years
1RM: one repetition maximum
1RM_bench_
: one repetition maximum bench
1RM_squat_
: one repetition maximum squat

## Funding

This research was funded by a Government Research Training Programme, University Faculty funding and with an in kind donation.

## Ethics Statement

Ethical approval for this trial was obtained from the University Research Ethics Board (reference no ETH24‐9364). Participants provided informed consent and completed the ESSA APSS with the primary researcher prior to all baseline assessments. Participants were also informed that they may withdraw from the study at any time, for any reason, without consequence. Amendments to the study protocol publicly are available via the Australian and New Zealand Clinical Trials Registry (trial number: ACTRN12624000556549). Data management procedures were conducted by the first and last authors. All collected data were deidentified using participant’s codes and stored electronically in a password‐protected drive at the University. The subsequent findings of the study will be disseminated through national and international academic meetings, through peer‐reviewed publication and by web‐based activities (e.g., podcasts and research webinars). Additionally, findings may also be disseminated through social media (e.g., Facebook and Twitter). Finally, participants received a personal and deidentified group results summary of the study findings.

## Consent

The authors have nothing to report.

## Conflicts of Interest

The last author may hold a perceived conflicts of interest as a previous colleague of the research partner. However, the last author is no longer associated with this organisation and holds no financial or nonfinancial interests that may interfere with this research. All other members of the research team declare they have no conflicts of interest (financial or nonfinancial) related to this research.

## Supporting information


Supporting Information S1

Supporting Information S2

## Data Availability

The data that support the findings of this study are available on request from the corresponding author. The data are not publicly available due to privacy or ethical restrictions.
