# Supplementary material for: Physical Adaptations to High‐Intensity Multimodal Training in Recreationally Active Adults: A Randomised Control Trial
Source: Eur J Sport Sci. 2026 Jun 12;26(7):e70199. doi: 10.1002/ejsc.70199 (PMC13263161; doi:10.1002/ejsc.70199)

**Supplementary Material 1** Intention to Treat Statistical Analysis

# Outcome: x1rmsquatrelative_kg

## Number of Participants Included: 67

## Distribution of DV at Baseline


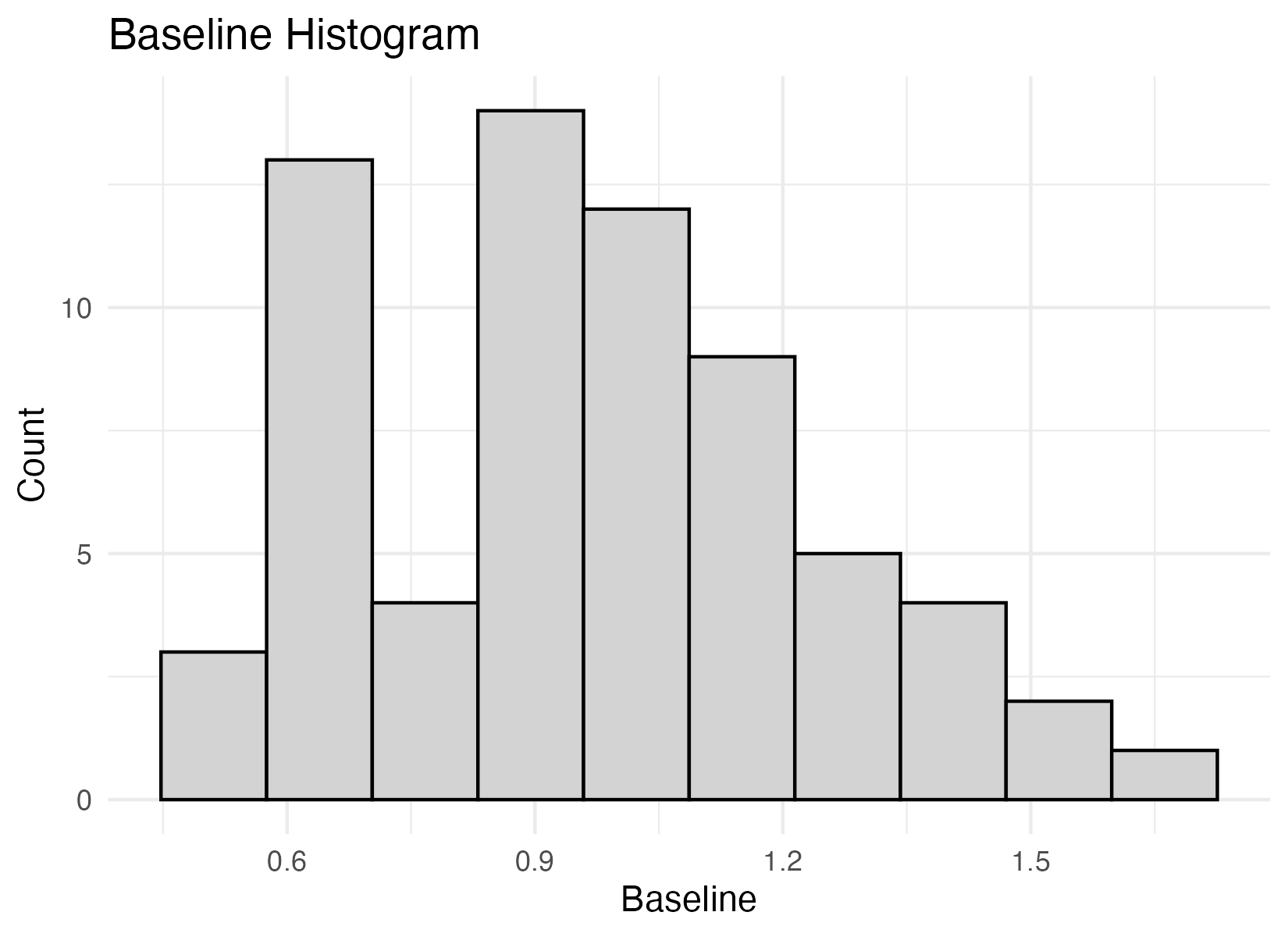


## Fitted vs Residuals


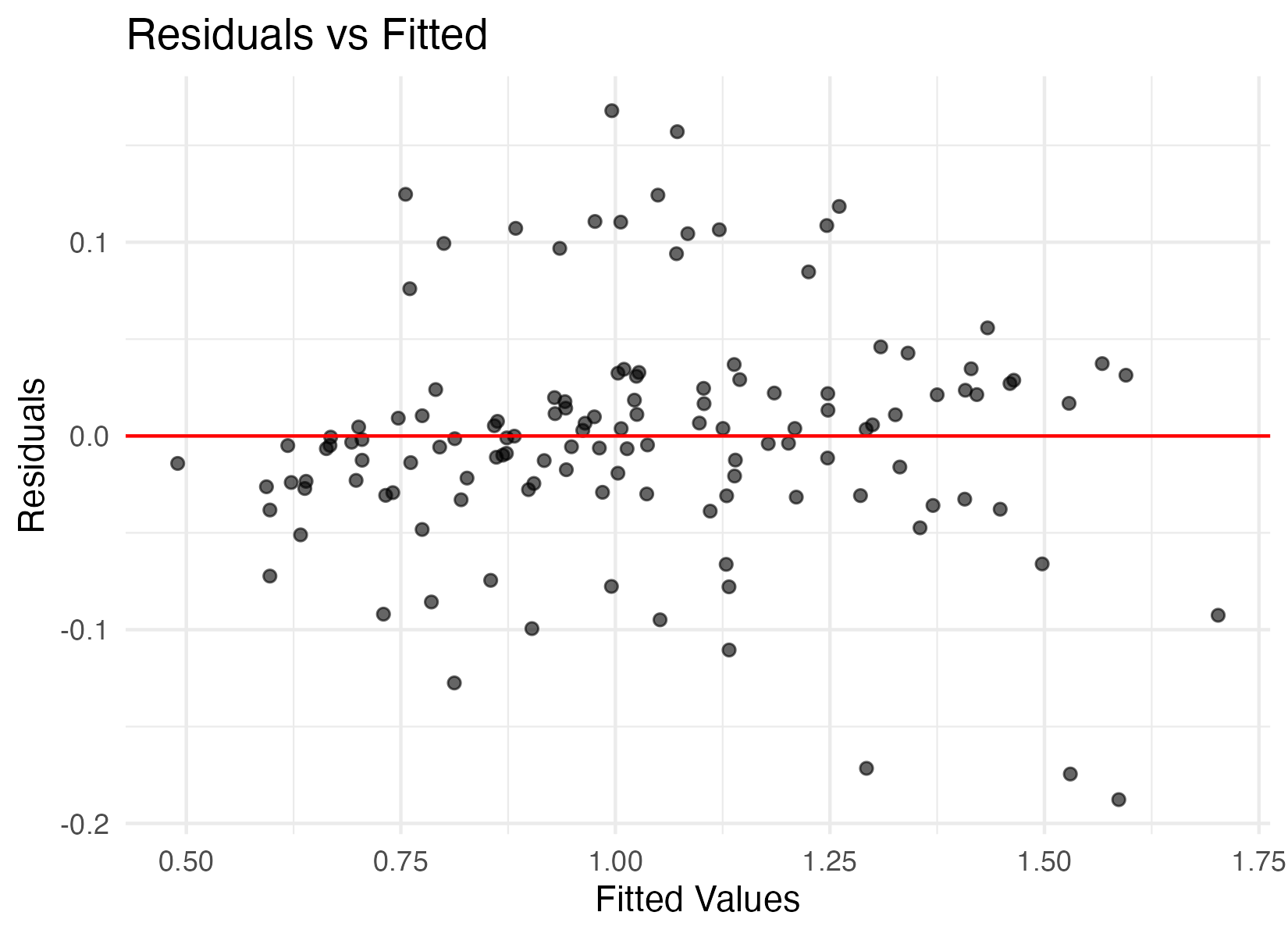


## QQ Plot


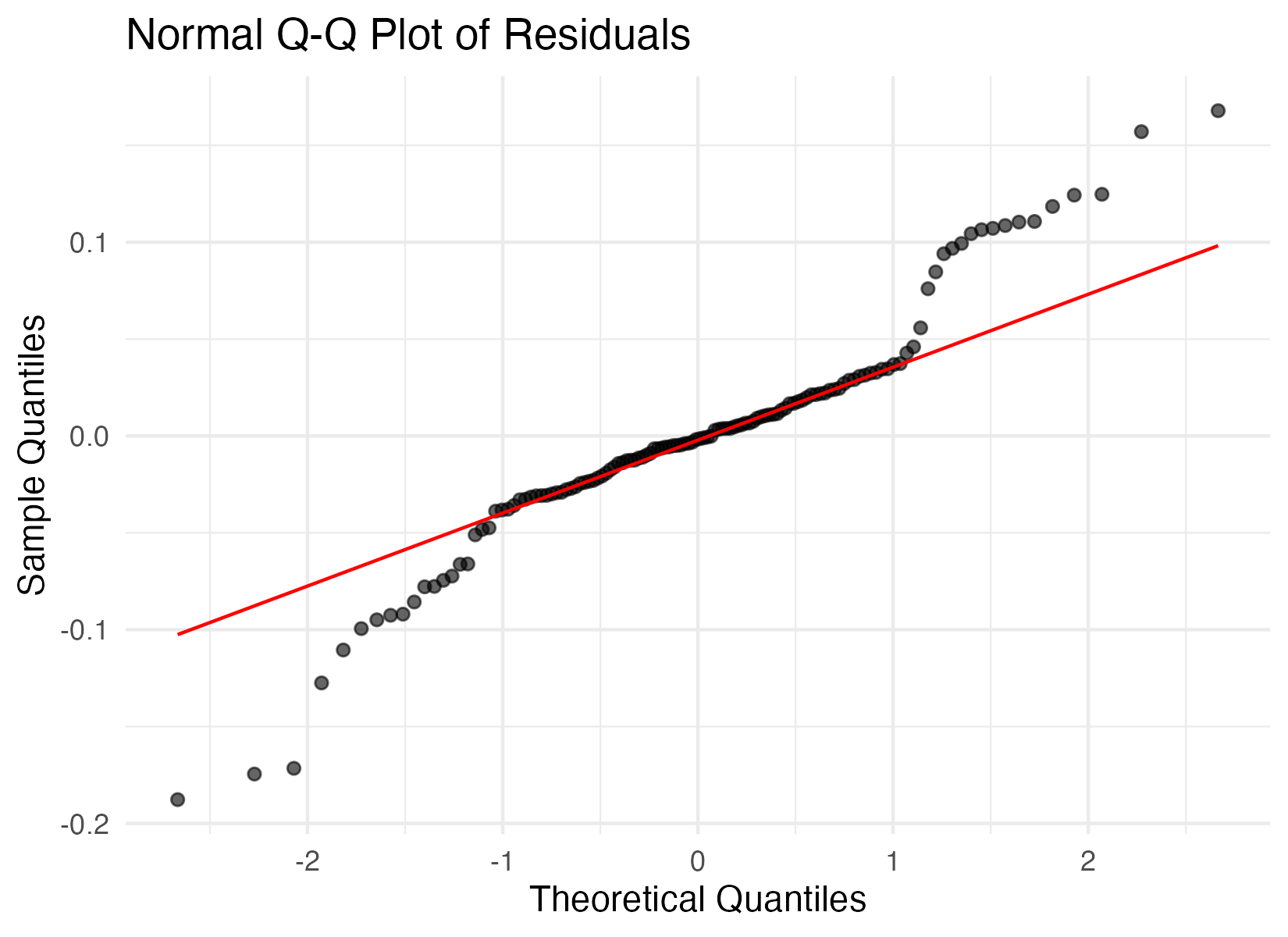


## Within-group change (baseline to follow-up)

| contrast | group | estimate | SE | df | lower.CL | upper.CL | t.ratio | p.value | effect_size |
| --- | --- | --- | --- | --- | --- | --- | --- | --- | --- |
| followup - baseline | C | 0.107 | 0.014 | 119 | 0.079 | 0.136 | 7.473 | <0.001 | 0.397 |
| followup - baseline | S | 0.122 | 0.015 | 119 | 0.092 | 0.153 | 8.011 | <0.001 | 0.452 |

## Between-group difference in change (interaction)

| timepoint_revpairwise | group_revpairwise | estimate | SE | df | lower.CL | upper.CL | t.ratio | p.value | effect_size |
| --- | --- | --- | --- | --- | --- | --- | --- | --- | --- |
| followup - baseline | S - C | 0.015 | 0.021 | 119 | -0.027 | 0.056 | 0.711 | 0.478 | 0.055 |

## Adjusted Means Over Time (with 95% CI)


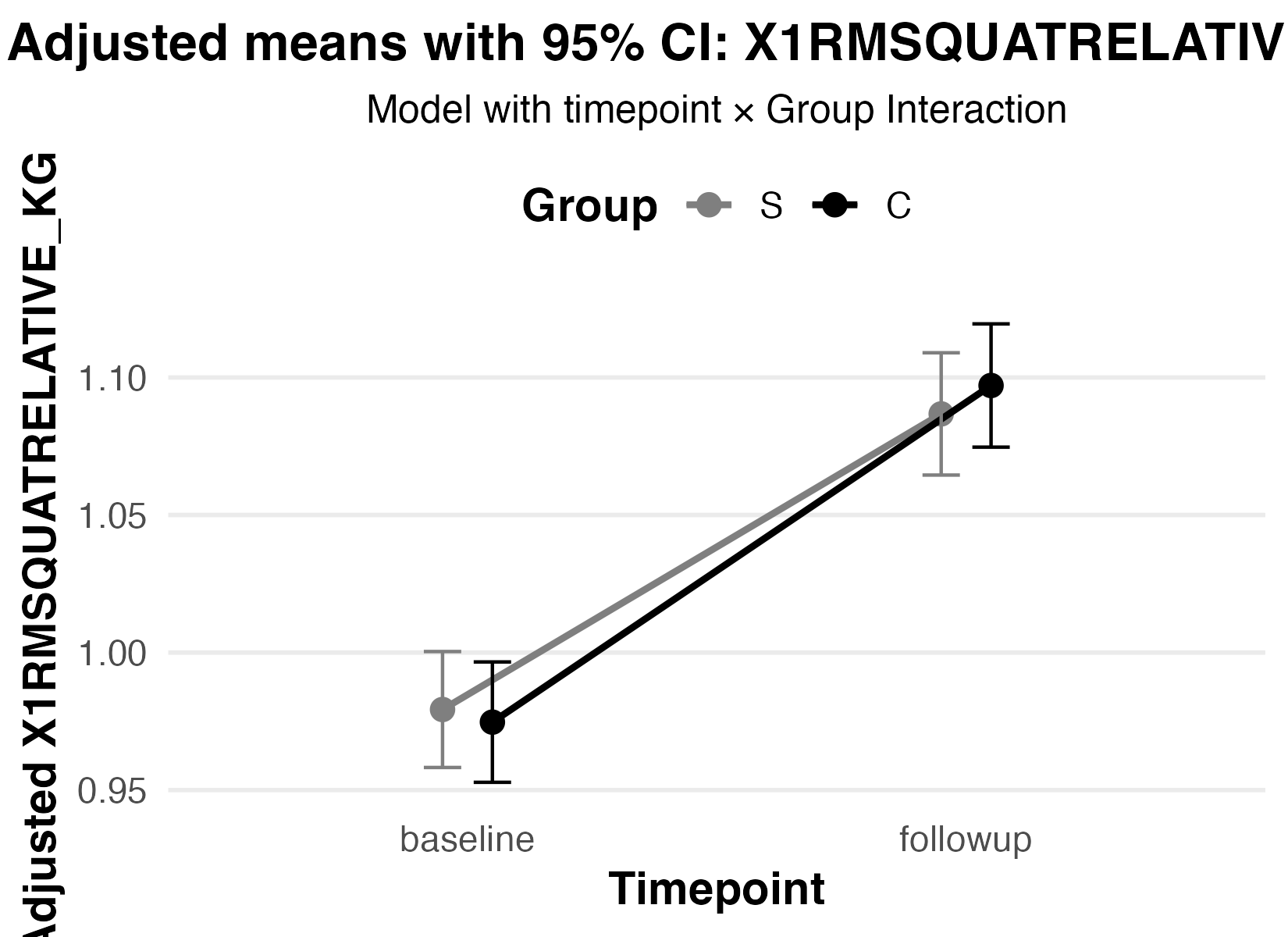


# Outcome: x1rmbench_relative_kg

## Number of Participants Included: 79

## Distribution of DV at Baseline


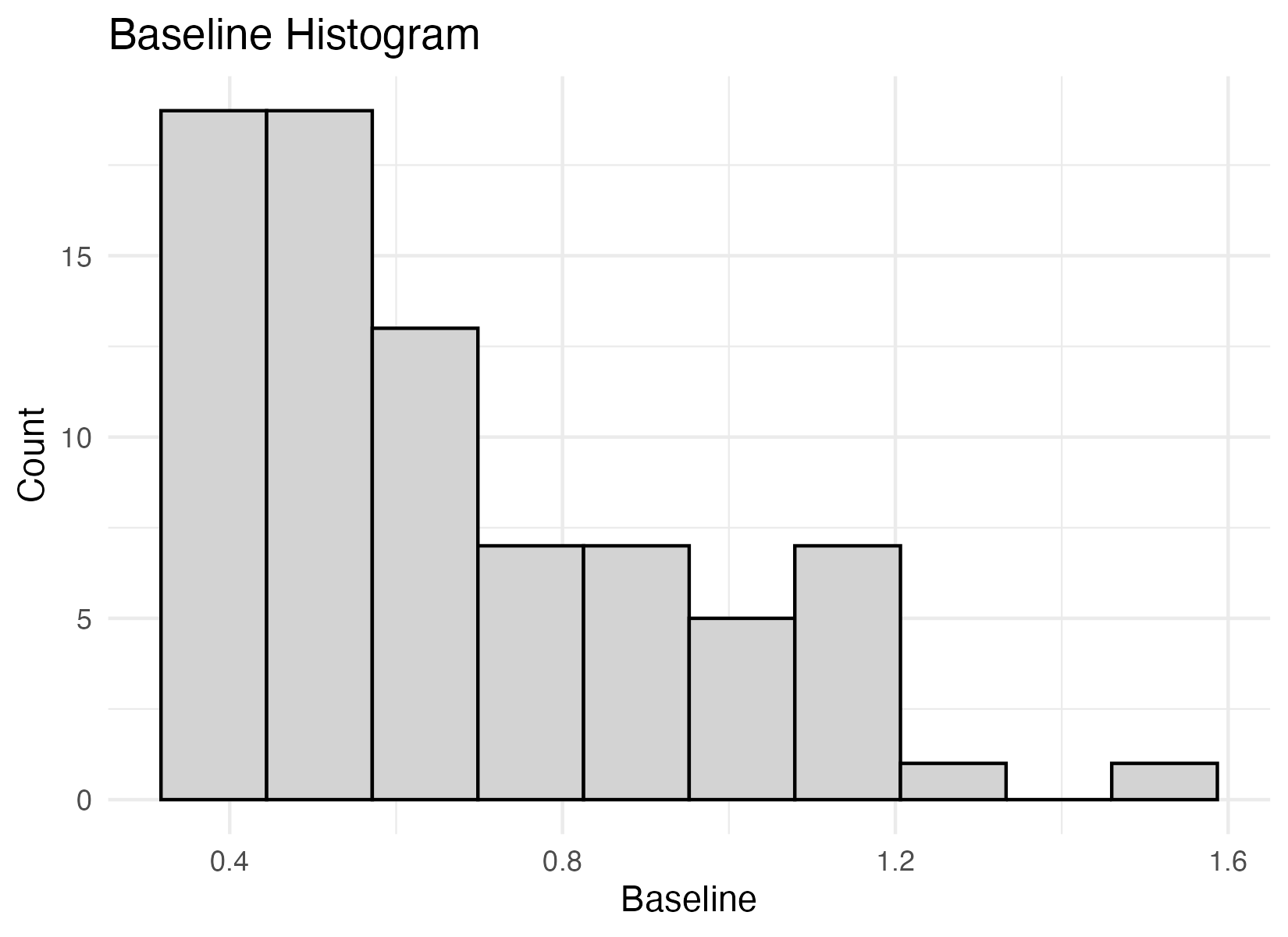


## Fitted vs Residuals


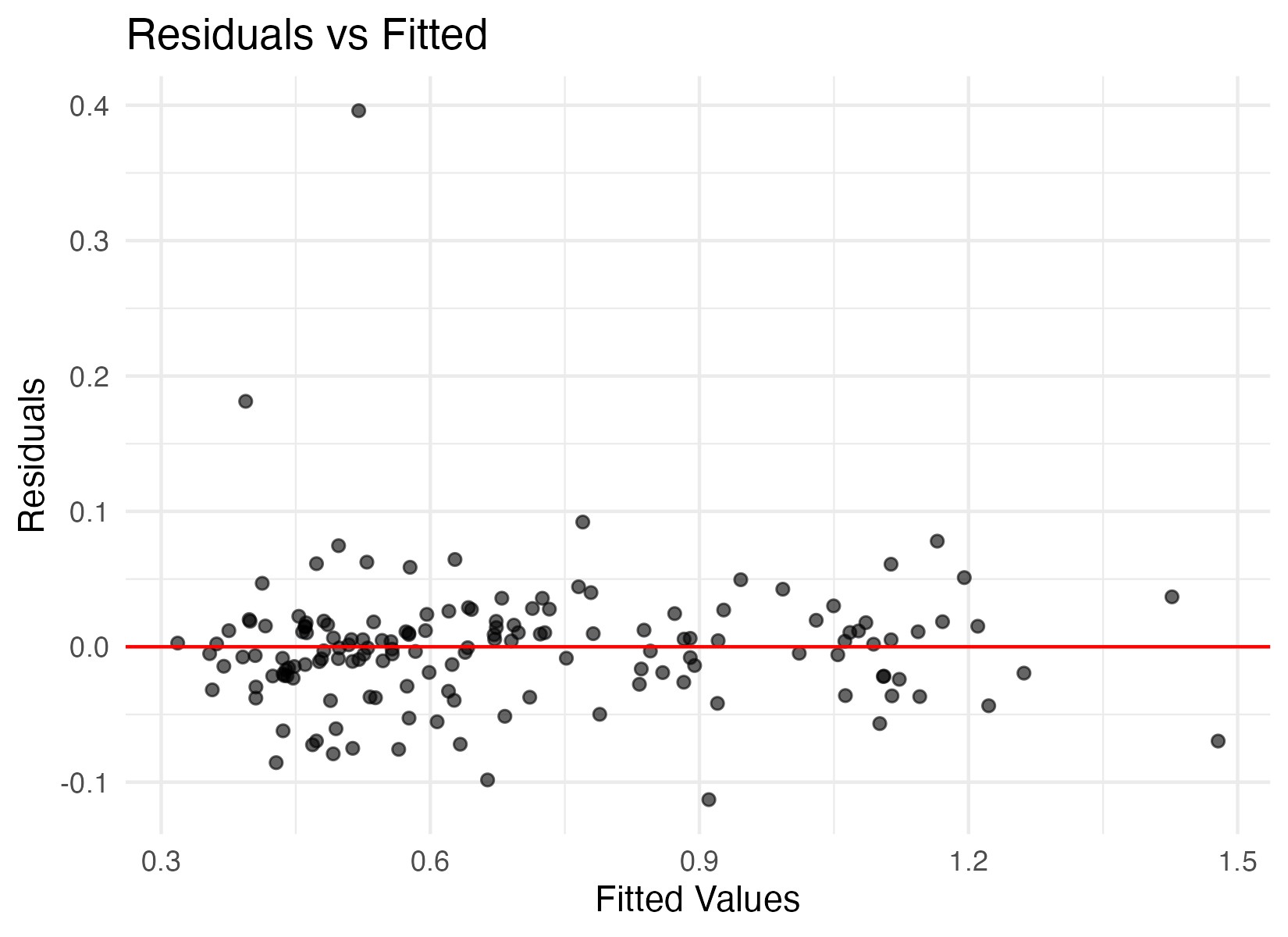


## QQ Plot


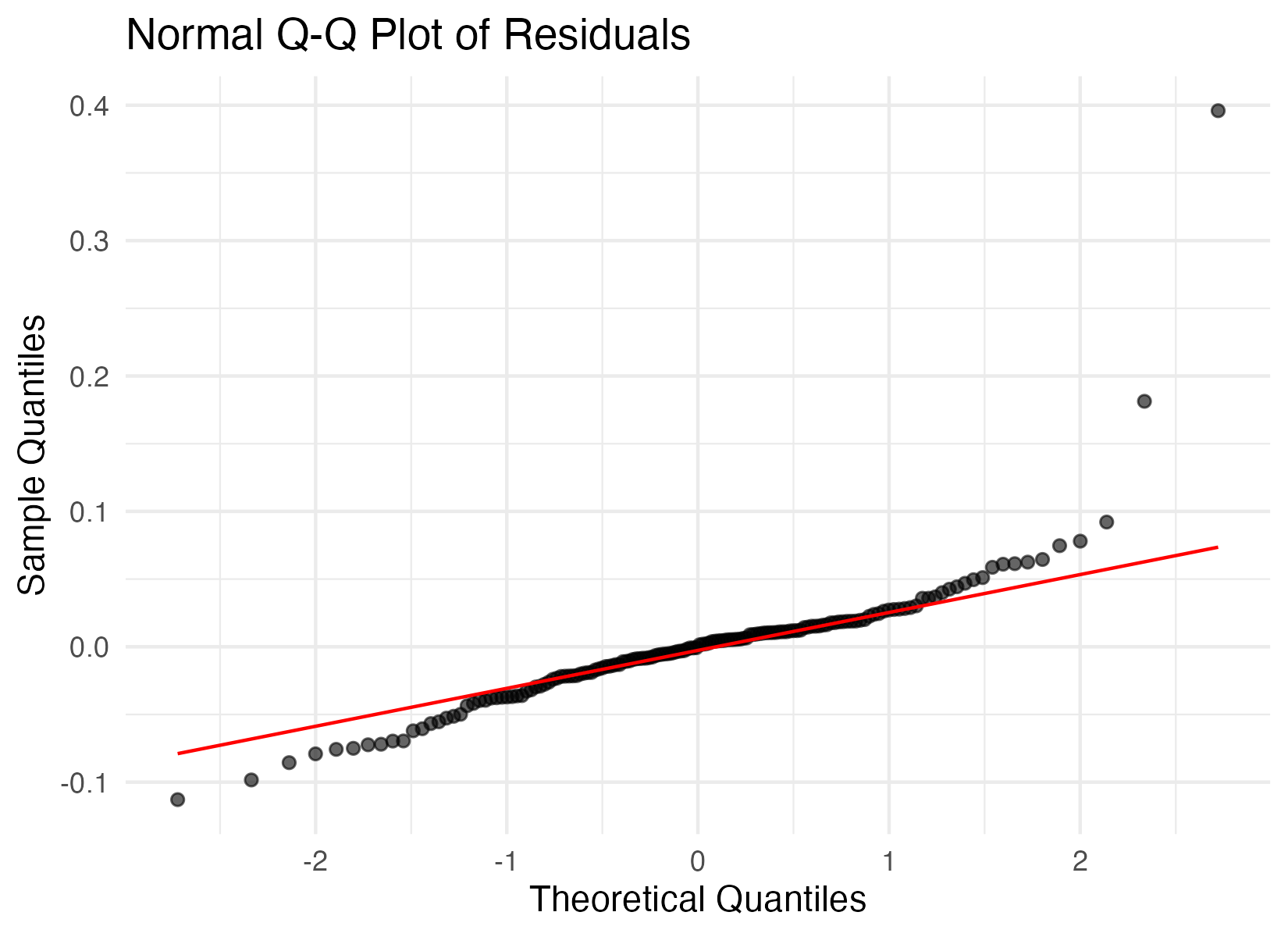


## Within-group change (baseline to follow-up)

| contrast | group | estimate | SE | df | lower.CL | upper.CL | t.ratio | p.value | effect_size |
| --- | --- | --- | --- | --- | --- | --- | --- | --- | --- |
| followup - baseline | C | 0.051 | 0.011 | 143 | 0.029 | 0.074 | 4.610 | <0.001 | 0.195 |
| followup - baseline | S | 0.037 | 0.011 | 143 | 0.015 | 0.060 | 3.252 | 0.001 | 0.141 |

## Between-group difference in change (interaction)

| timepoint_revpairwise | group_revpairwise | estimate | SE | df | lower.CL | upper.CL | t.ratio | p.value | effect_size |
| --- | --- | --- | --- | --- | --- | --- | --- | --- | --- |
| followup - baseline | S - C | -0.014 | 0.016 | 143 | -0.046 | 0.017 | -0.891 | 0.375 | -0.054 |

## Adjusted Means Over Time (with 95% CI)


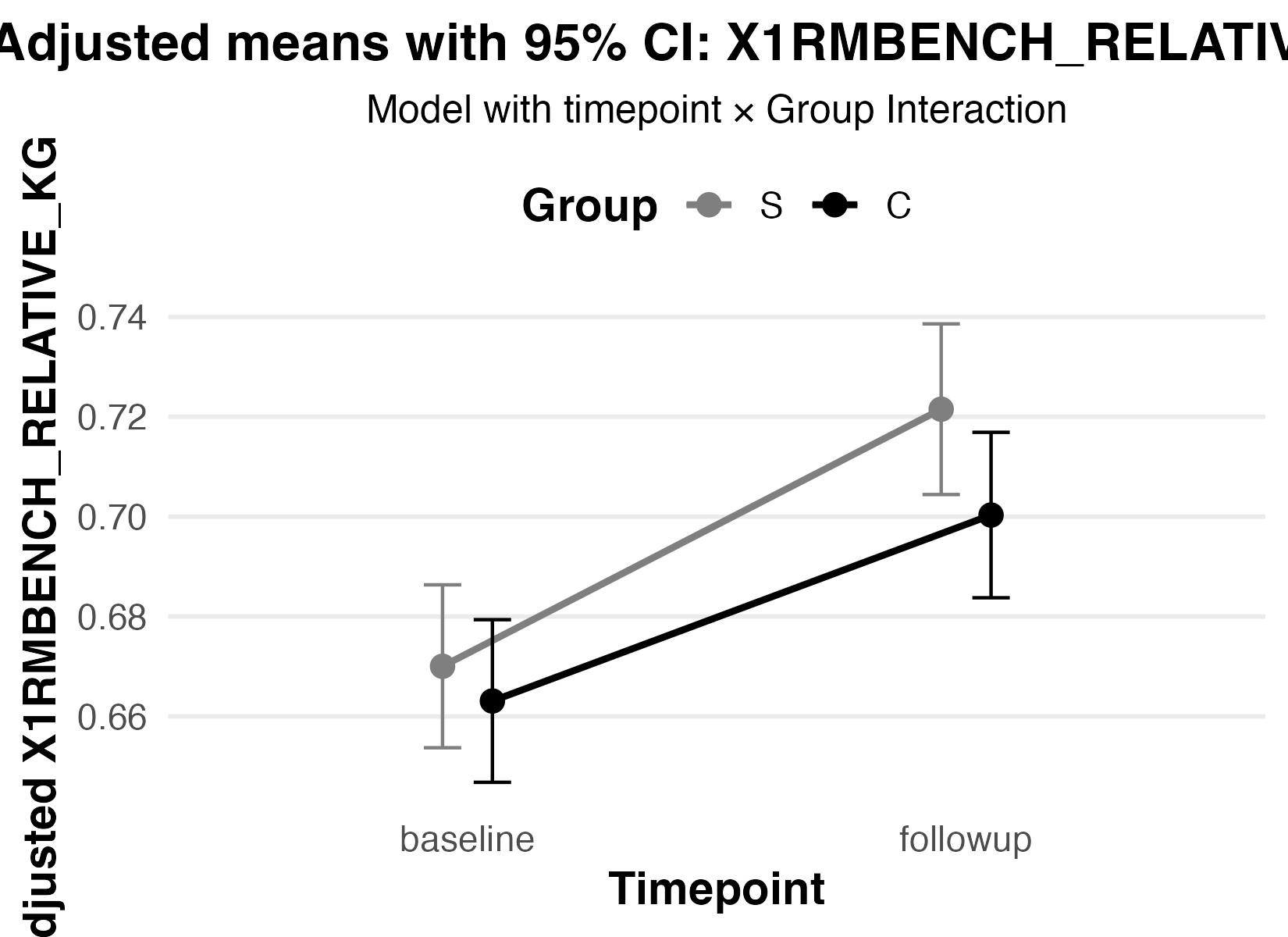


# Outcome: bodymass_kg

## Number of Participants Included: 79

## Distribution of DV at Baseline


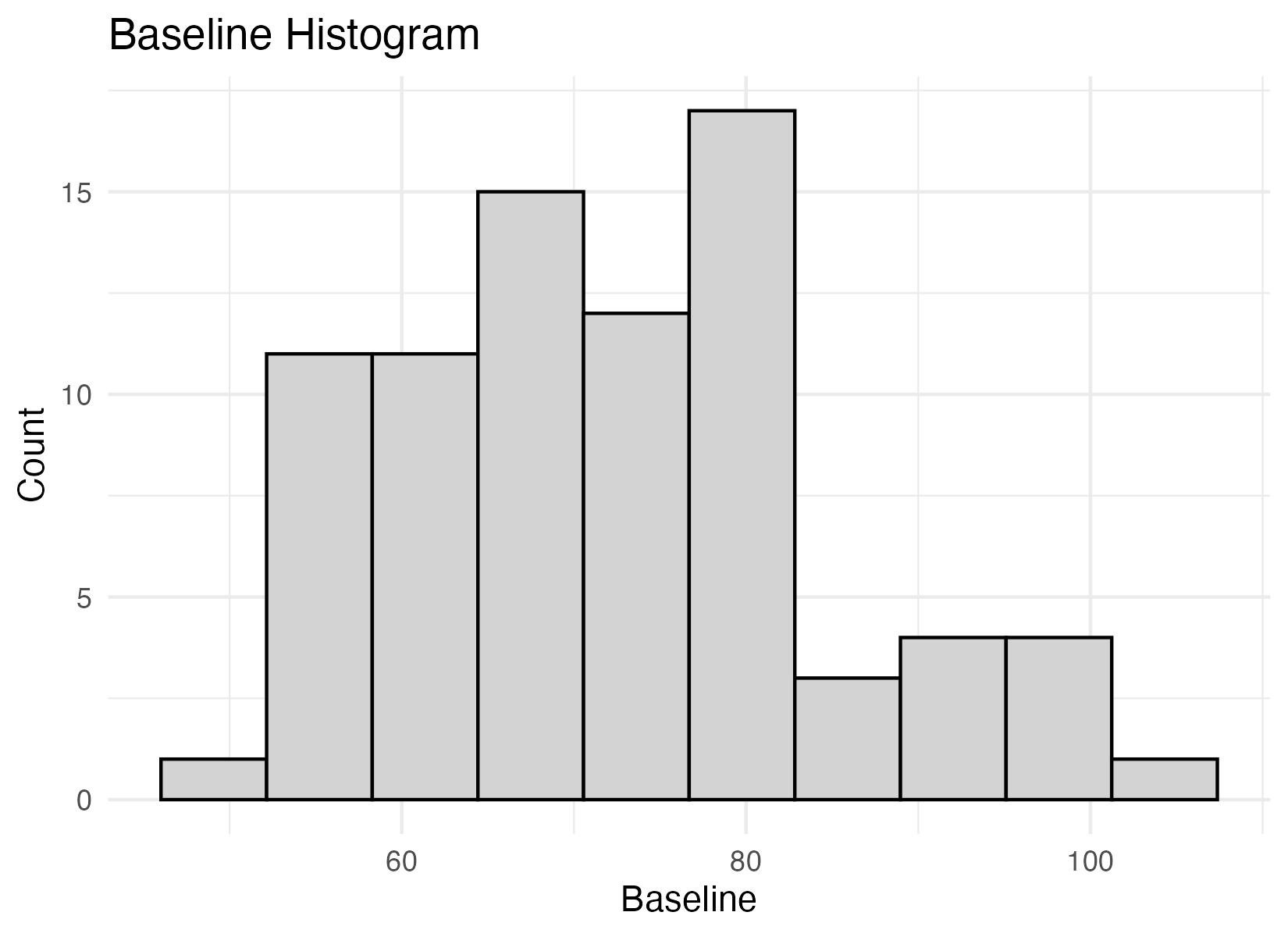


## Fitted vs Residuals


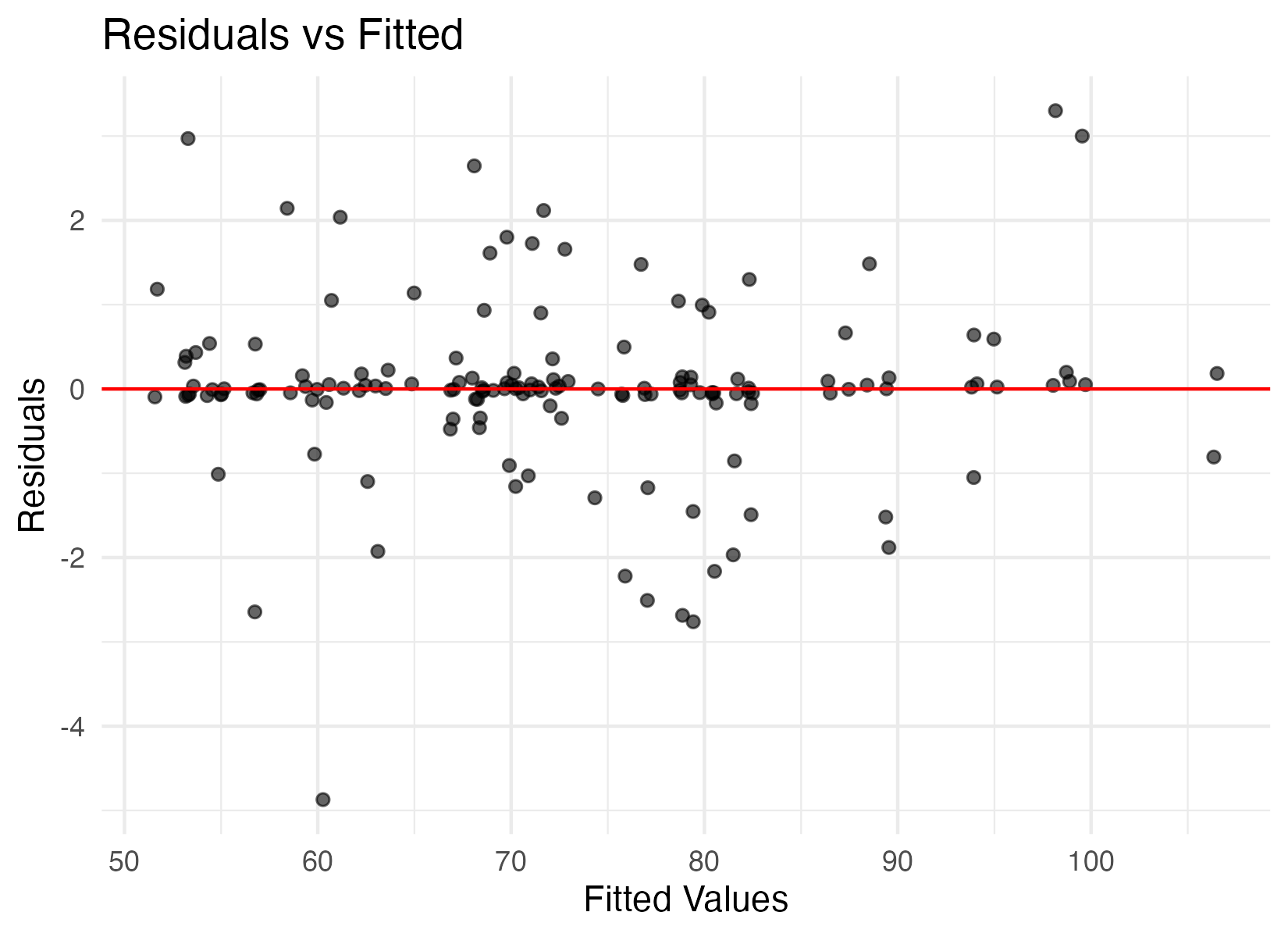


## QQ Plot


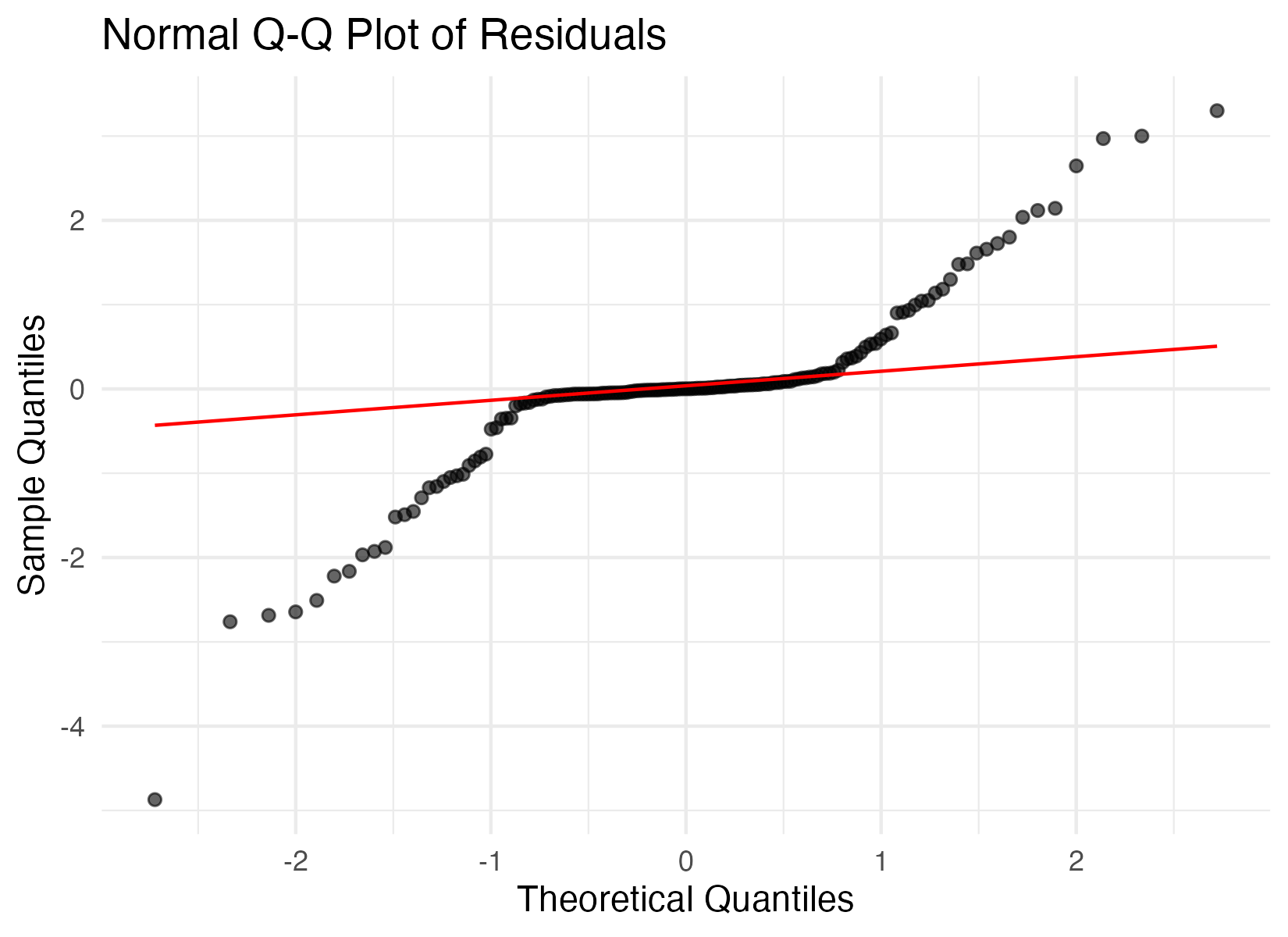


## Within-group change (baseline to follow-up)

| contrast | group | estimate | SE | df | lower.CL | upper.CL | t.ratio | p.value | effect_size |
| --- | --- | --- | --- | --- | --- | --- | --- | --- | --- |
| followup - baseline | C | 0.122 | 0.237 | 143 | -0.346 | 0.590 | 0.515 | 0.607 | 0.010 |
| followup - baseline | S | -0.169 | 0.243 | 143 | -0.648 | 0.311 | -0.695 | 0.488 | -0.013 |

## Between-group difference in change (interaction)

| timepoint_revpairwise | group_revpairwise | estimate | SE | df | lower.CL | upper.CL | t.ratio | p.value | effect_size |
| --- | --- | --- | --- | --- | --- | --- | --- | --- | --- |
| followup - baseline | S - C | -0.291 | 0.339 | 143 | -0.961 | 0.379 | -0.857 | 0.393 | -0.023 |

## Adjusted Means Over Time (with 95% CI)


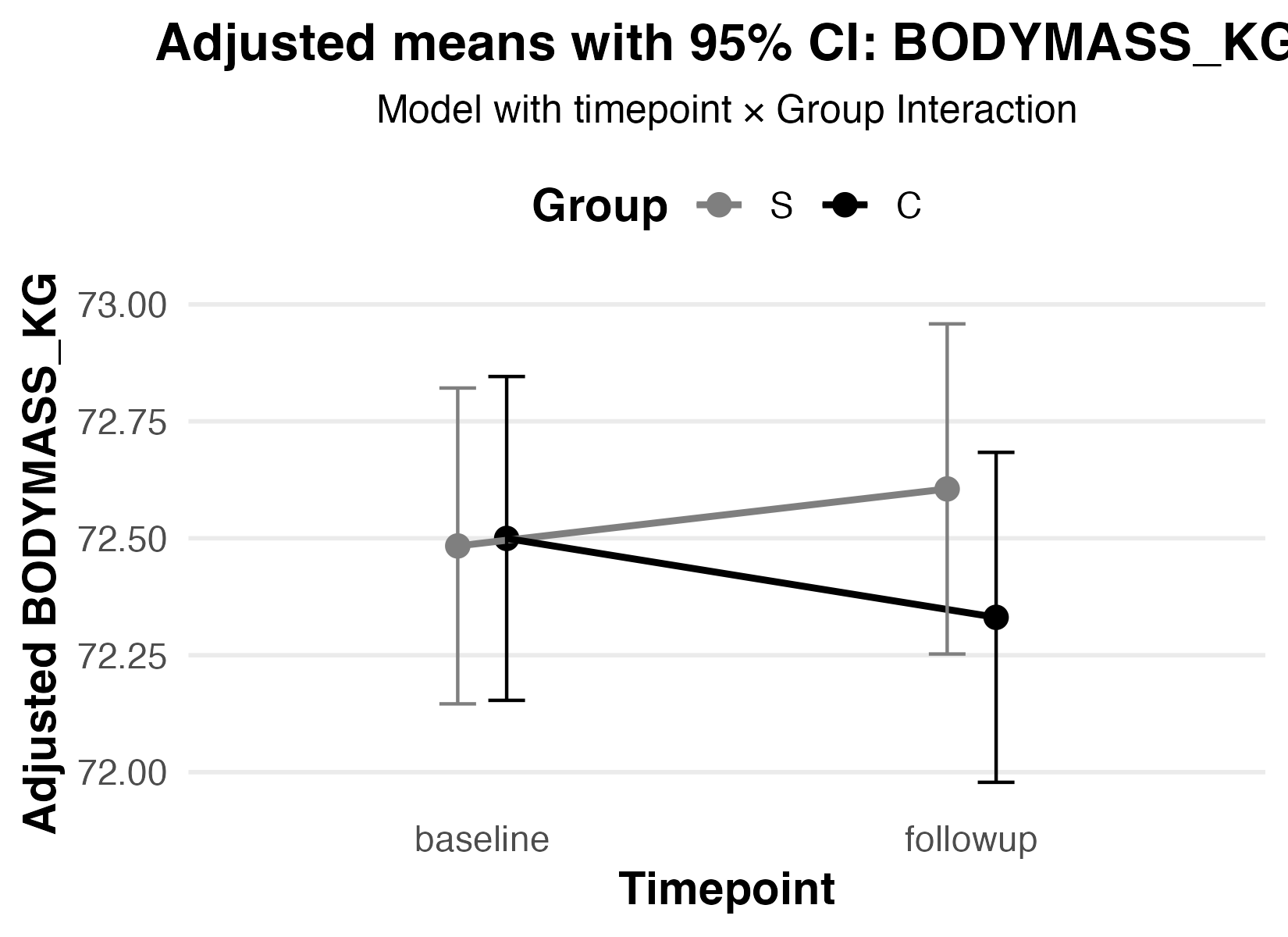


# Outcome: bmi

## Number of Participants Included: 79

## Distribution of DV at Baseline


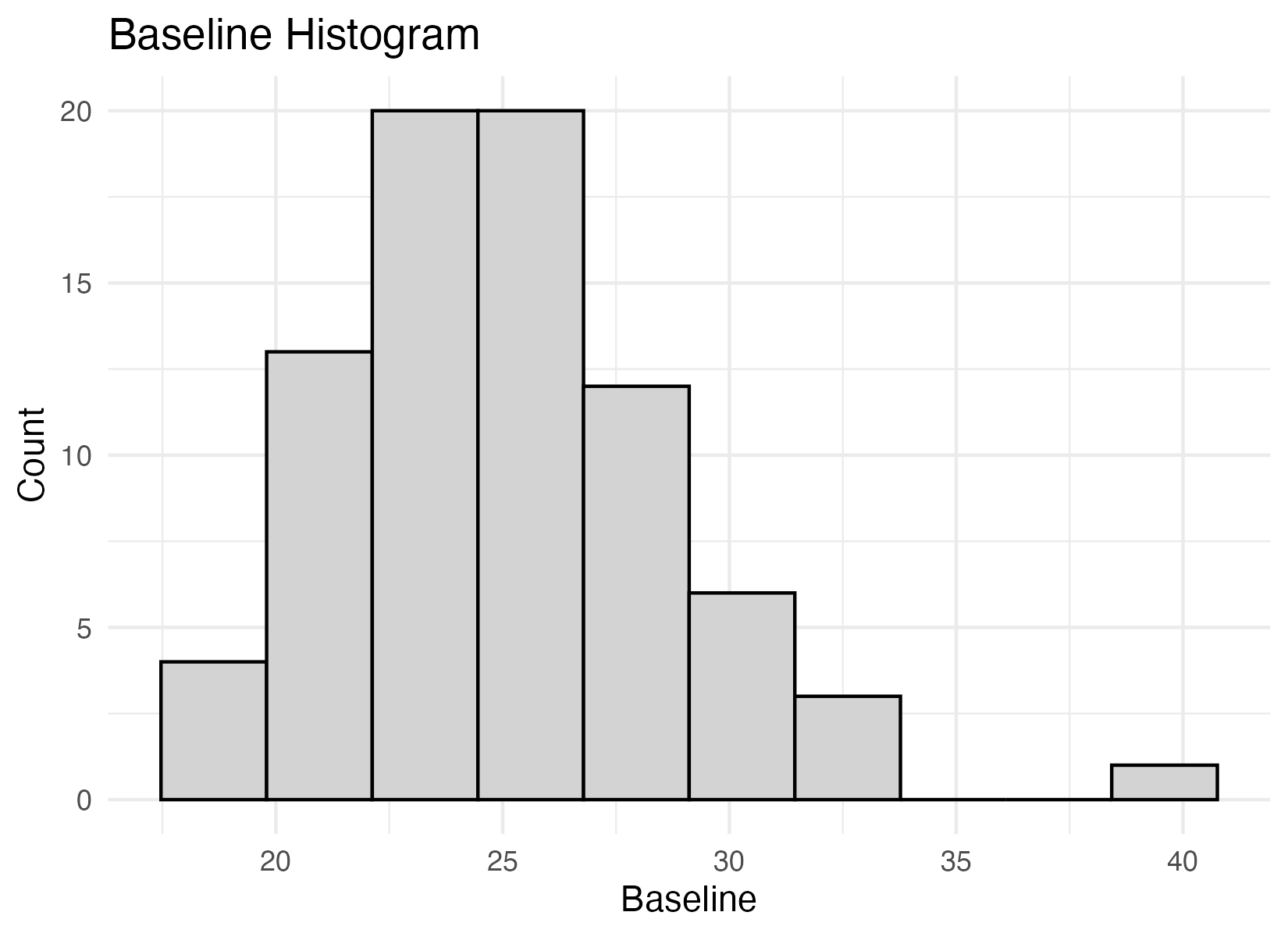


## Fitted vs Residuals


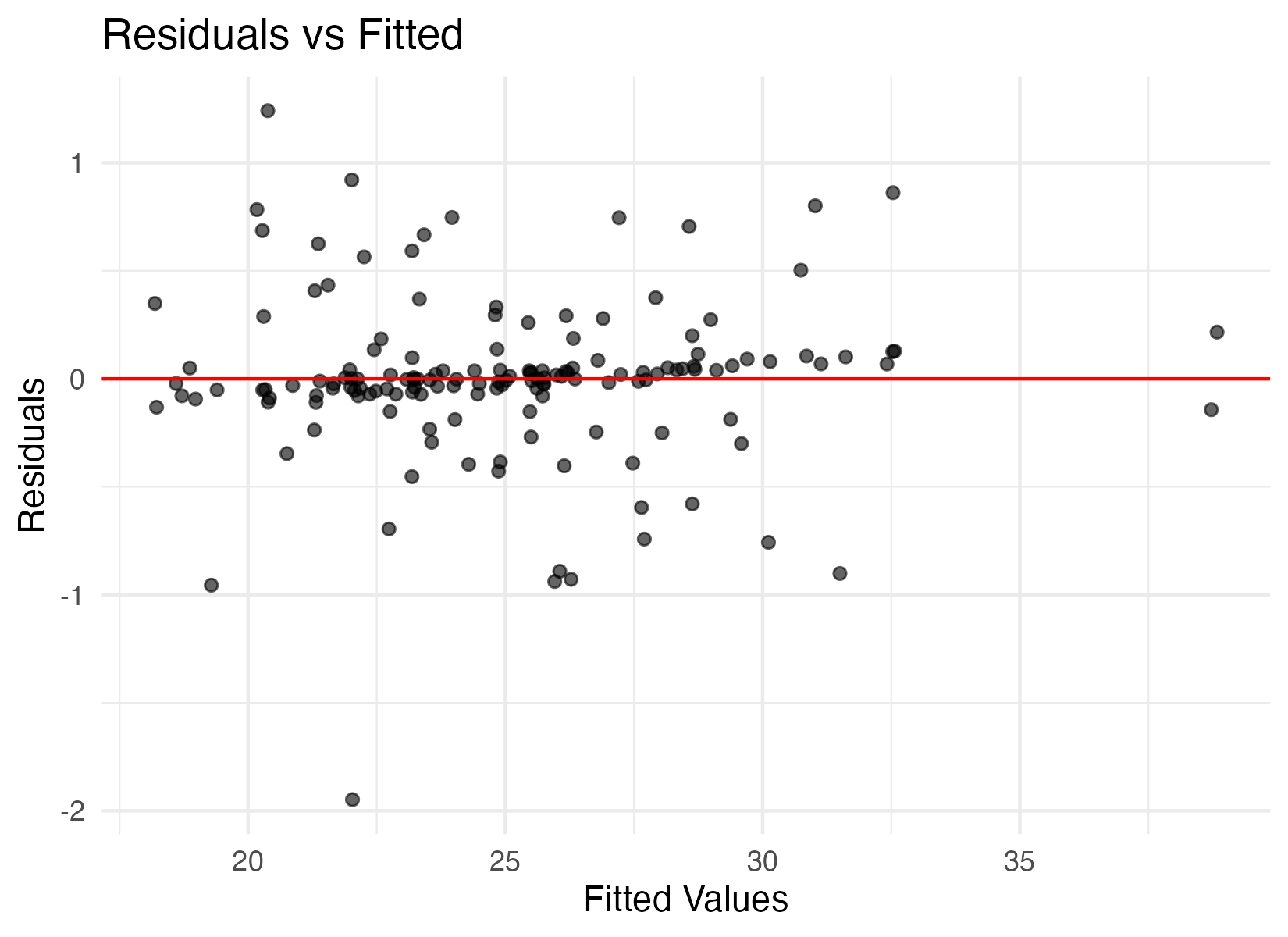


## QQ Plot


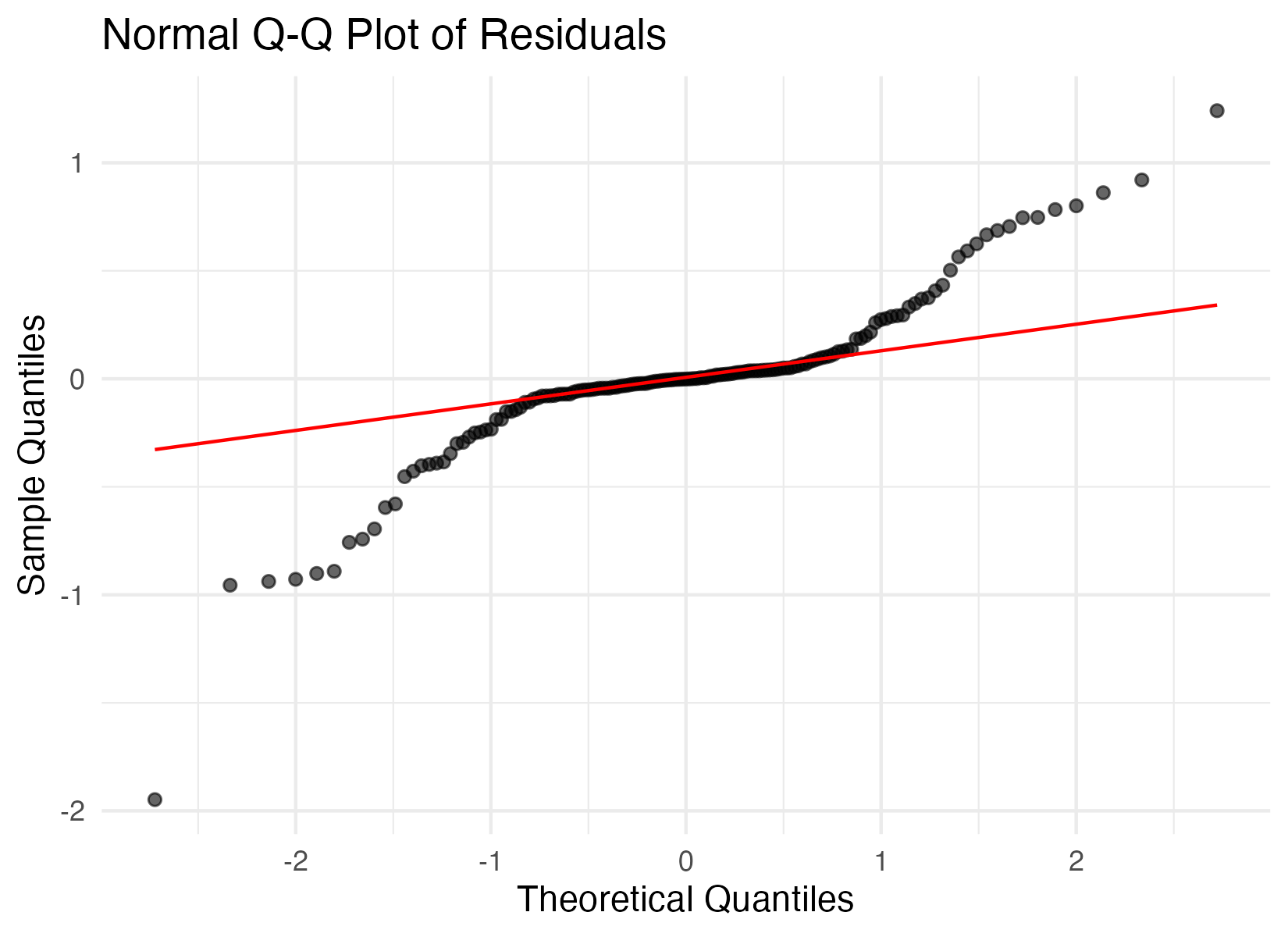


## Within-group change (baseline to follow-up)

| contrast | group | estimate | SE | df | lower.CL | upper.CL | t.ratio | p.value | effect_size |
| --- | --- | --- | --- | --- | --- | --- | --- | --- | --- |
| followup - baseline | C | -0.032 | 0.085 | 143 | -0.201 | 0.137 | -0.376 | 0.707 | -0.009 |
| followup - baseline | S | -0.112 | 0.088 | 143 | -0.285 | 0.061 | -1.279 | 0.203 | -0.030 |

## Between-group difference in change (interaction)

| timepoint_revpairwise | group_revpairwise | estimate | SE | df | lower.CL | upper.CL | t.ratio | p.value | effect_size |
| --- | --- | --- | --- | --- | --- | --- | --- | --- | --- |
| followup - baseline | S - C | -0.08 | 0.122 | 143 | -0.322 | 0.162 | -0.653 | 0.515 | -0.022 |

## Adjusted Means Over Time (with 95% CI)


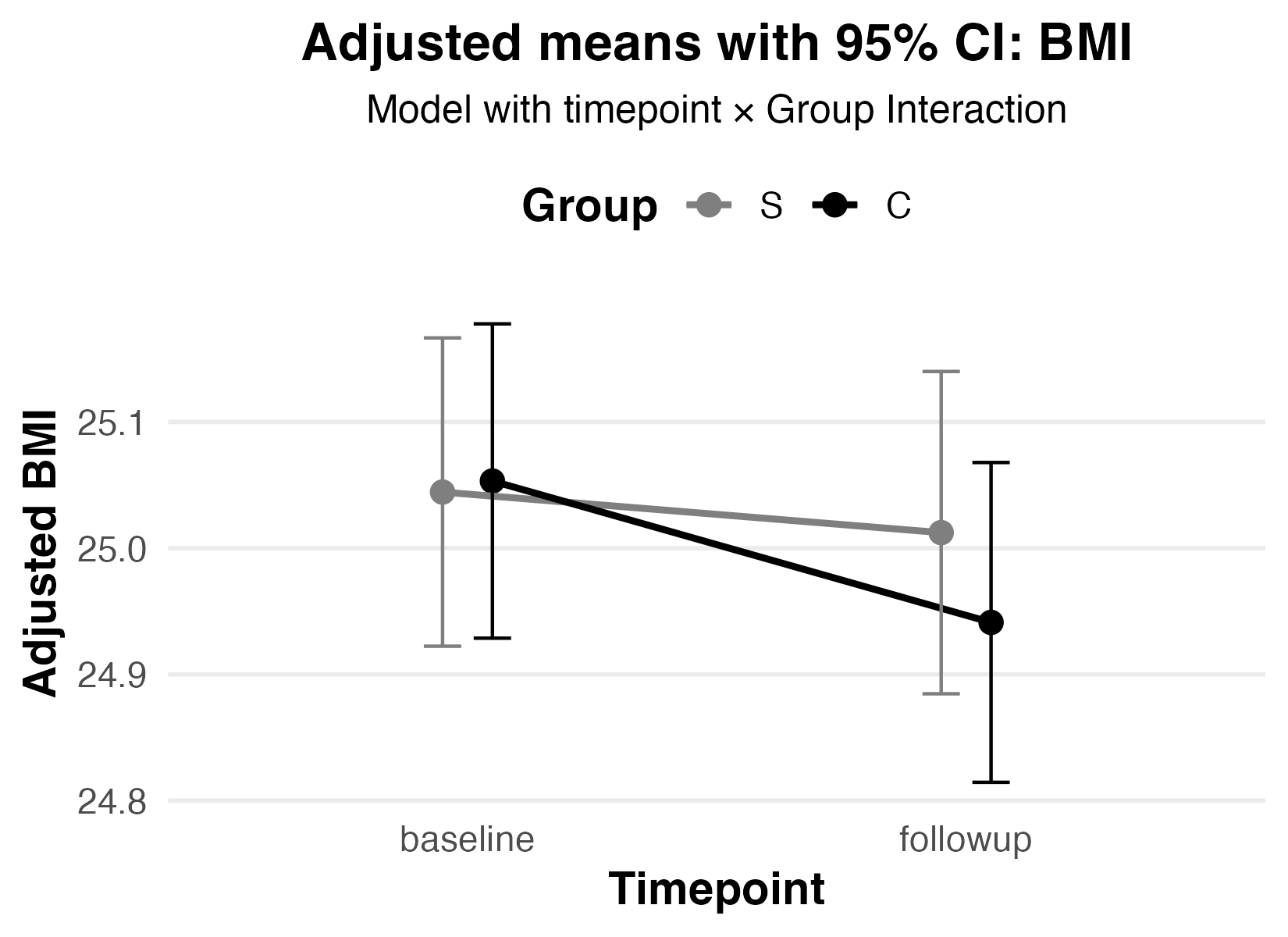


# Outcome: waist_cm

## Number of Participants Included: 79

## Distribution of DV at Baseline


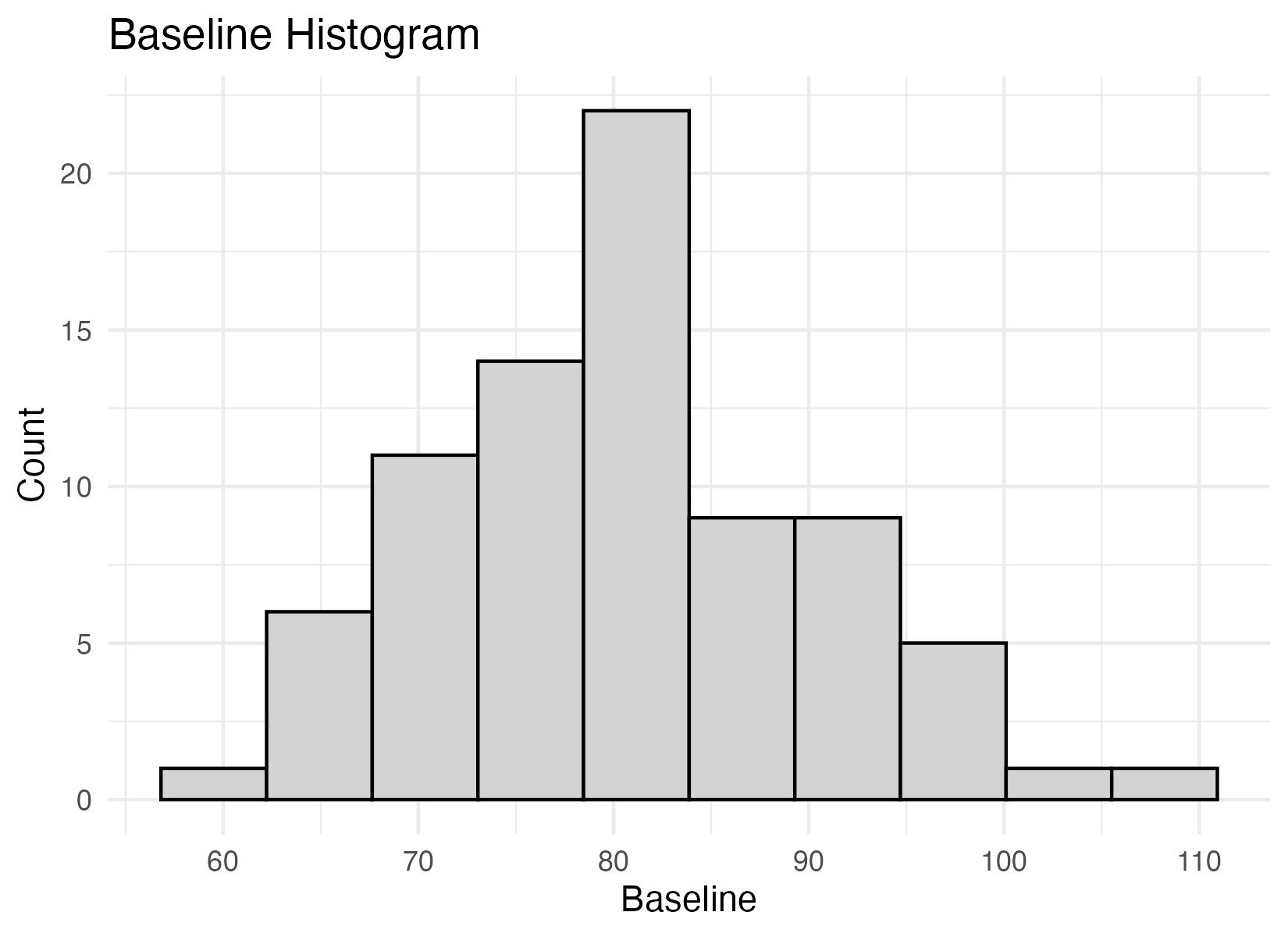


## Fitted vs Residuals


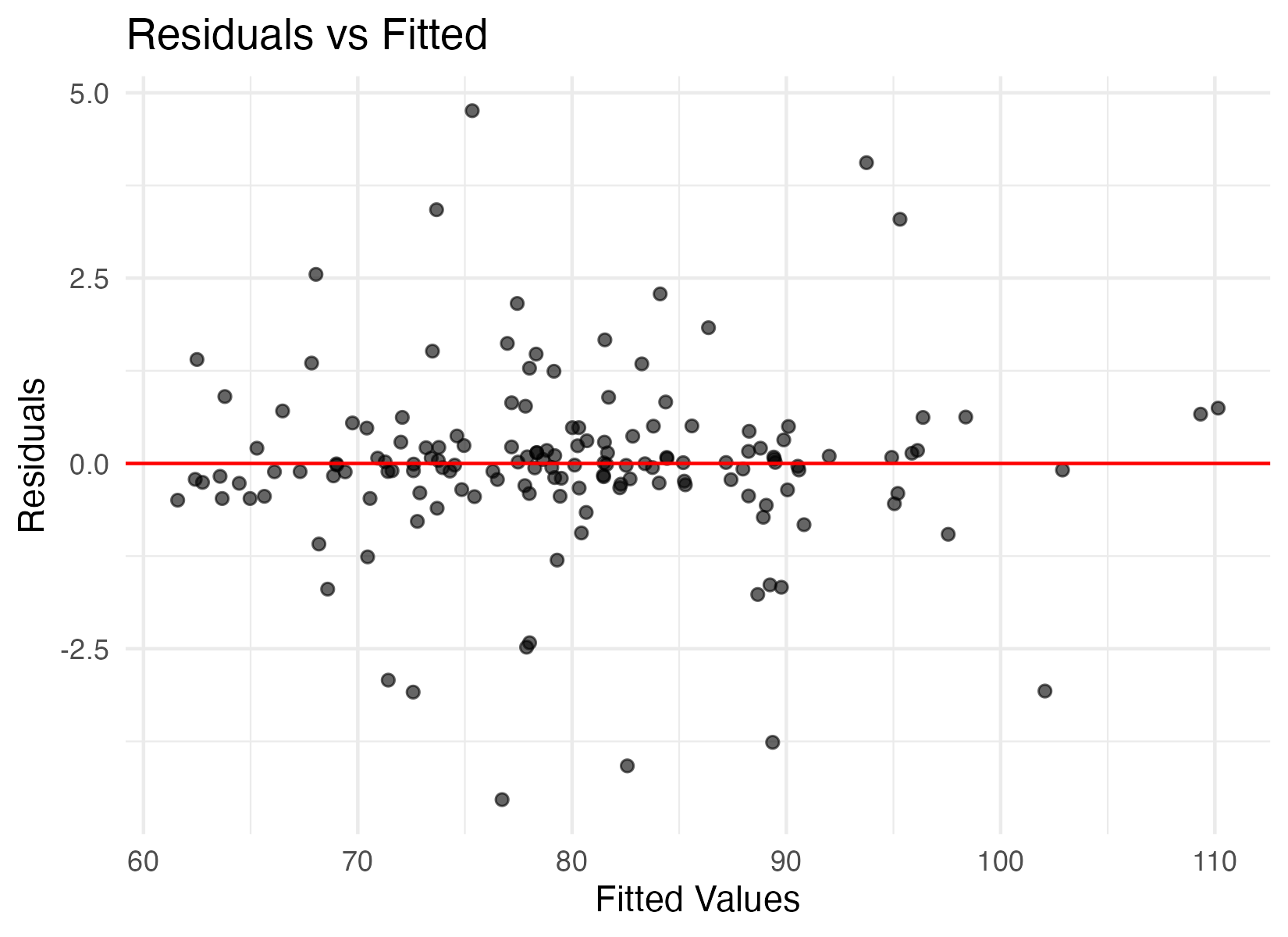


## QQ Plot


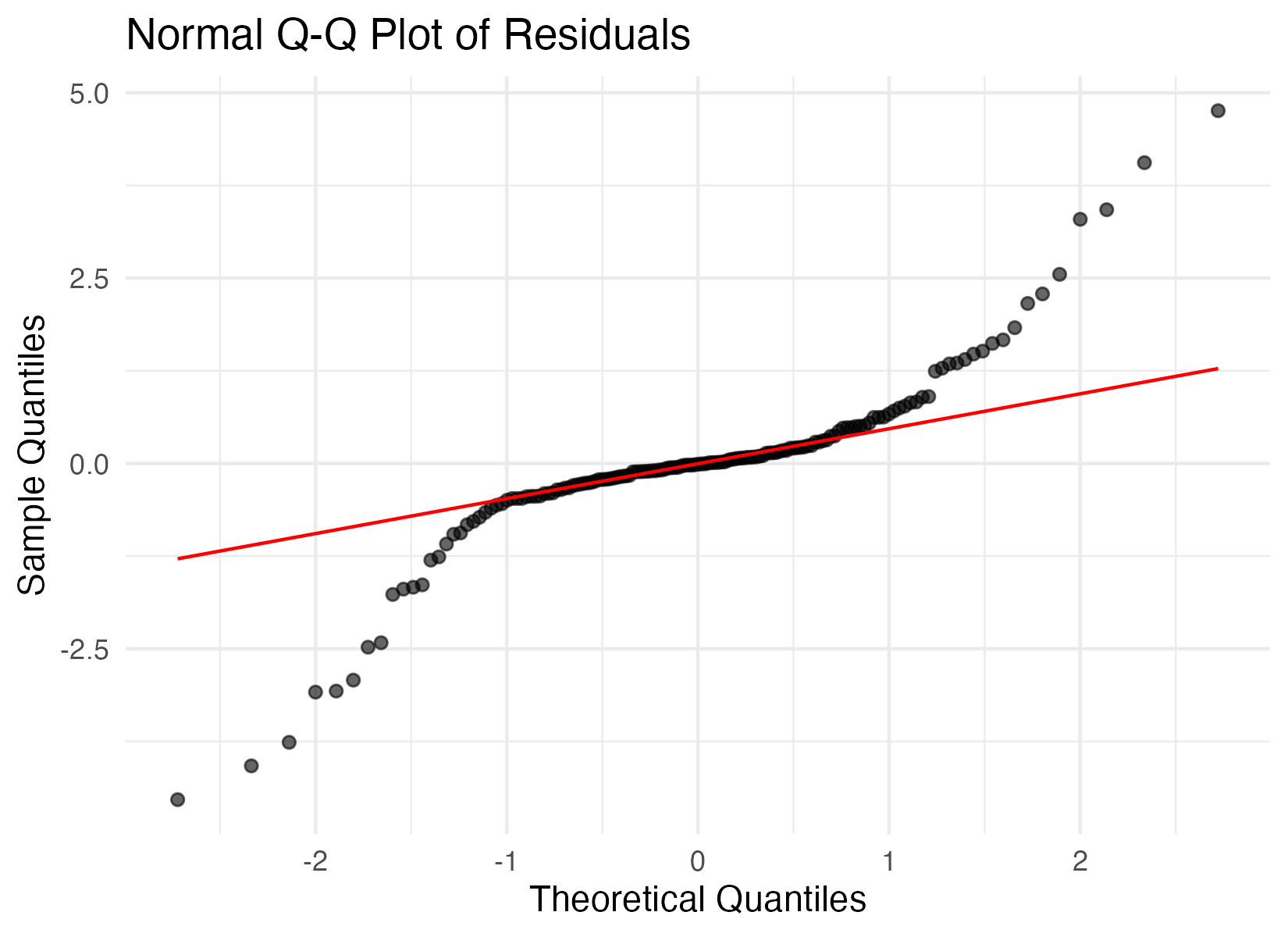


## Within-group change (baseline to follow-up)

| contrast | group | estimate | SE | df | lower.CL | upper.CL | t.ratio | p.value | effect_size |
| --- | --- | --- | --- | --- | --- | --- | --- | --- | --- |
| followup - baseline | C | -1.175 | 0.272 | 143 | -1.714 | -0.637 | -4.316 | <0.001 | -0.122 |
| followup - baseline | S | -0.818 | 0.279 | 143 | -1.370 | -0.266 | -2.931 | 0.004 | -0.085 |

## Between-group difference in change (interaction)

| timepoint_revpairwise | group_revpairwise | estimate | SE | df | lower.CL | upper.CL | t.ratio | p.value | effect_size |
| --- | --- | --- | --- | --- | --- | --- | --- | --- | --- |
| followup - baseline | S - C | 0.357 | 0.39 | 143 | -0.414 | 1.128 | 0.915 | 0.362 | 0.037 |

## Adjusted Means Over Time (with 95% CI)


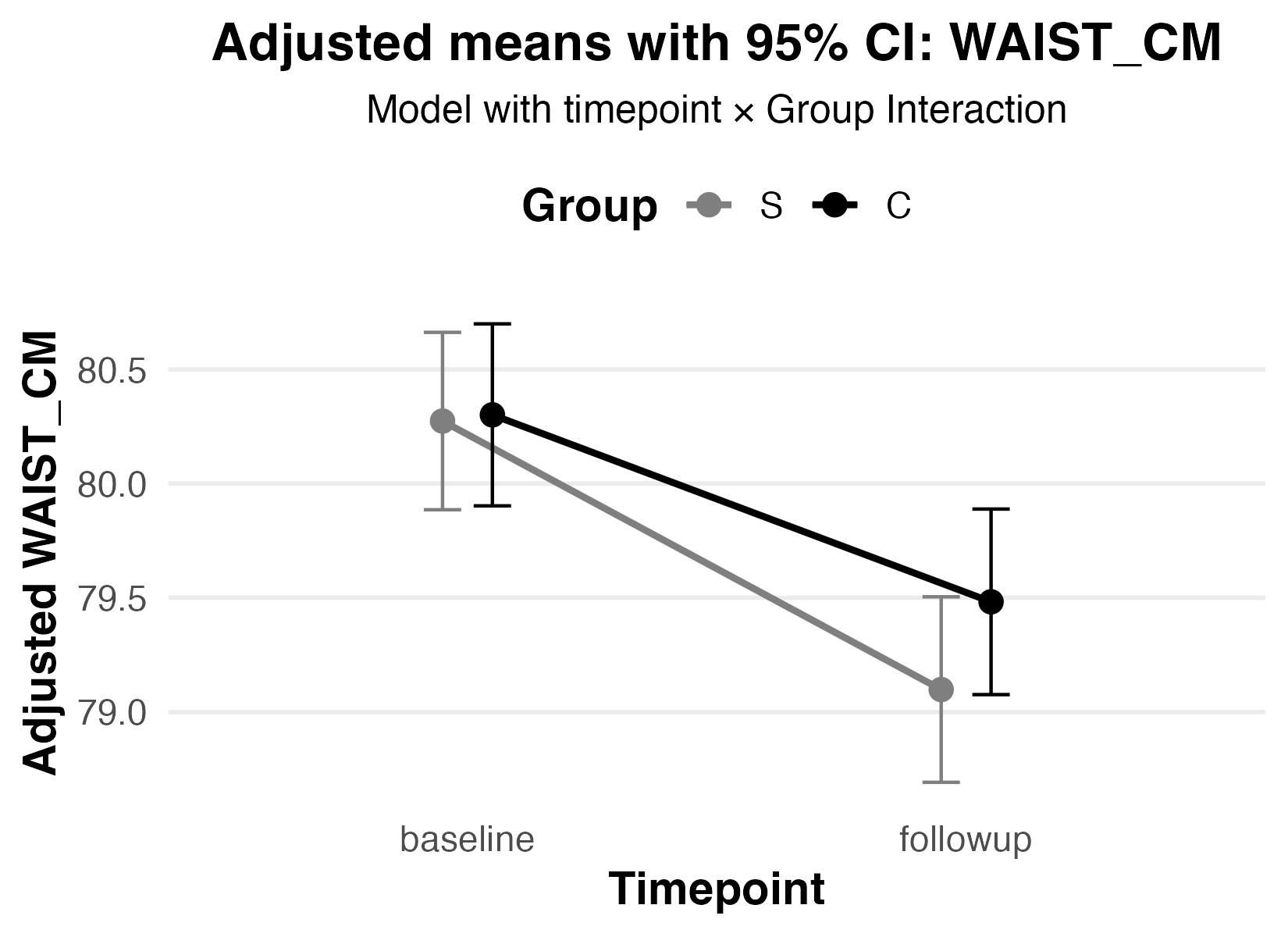


# Outcome: hip_cm

## Number of Participants Included: 79

## Distribution of DV at Baseline


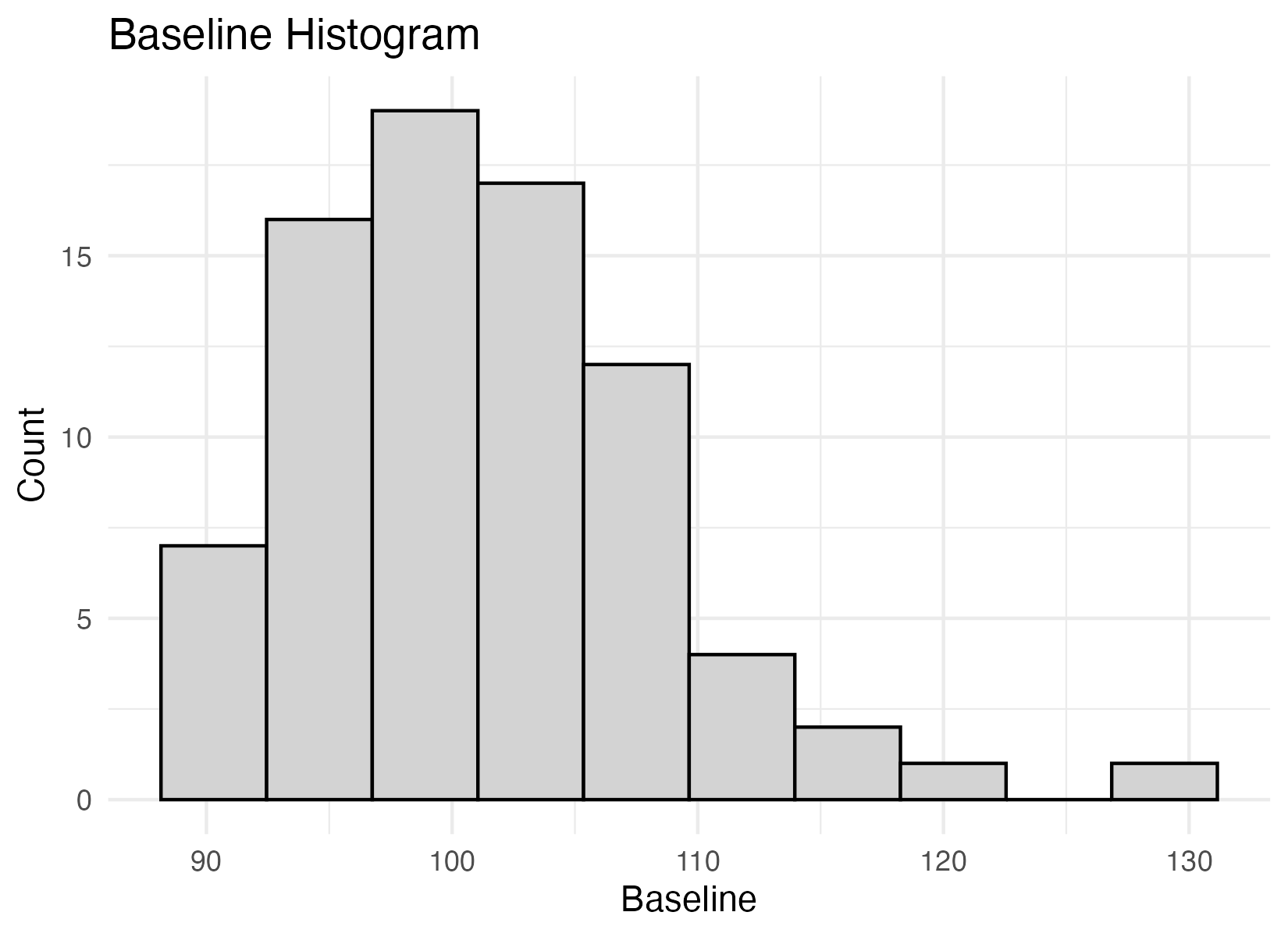


## Fitted vs Residuals


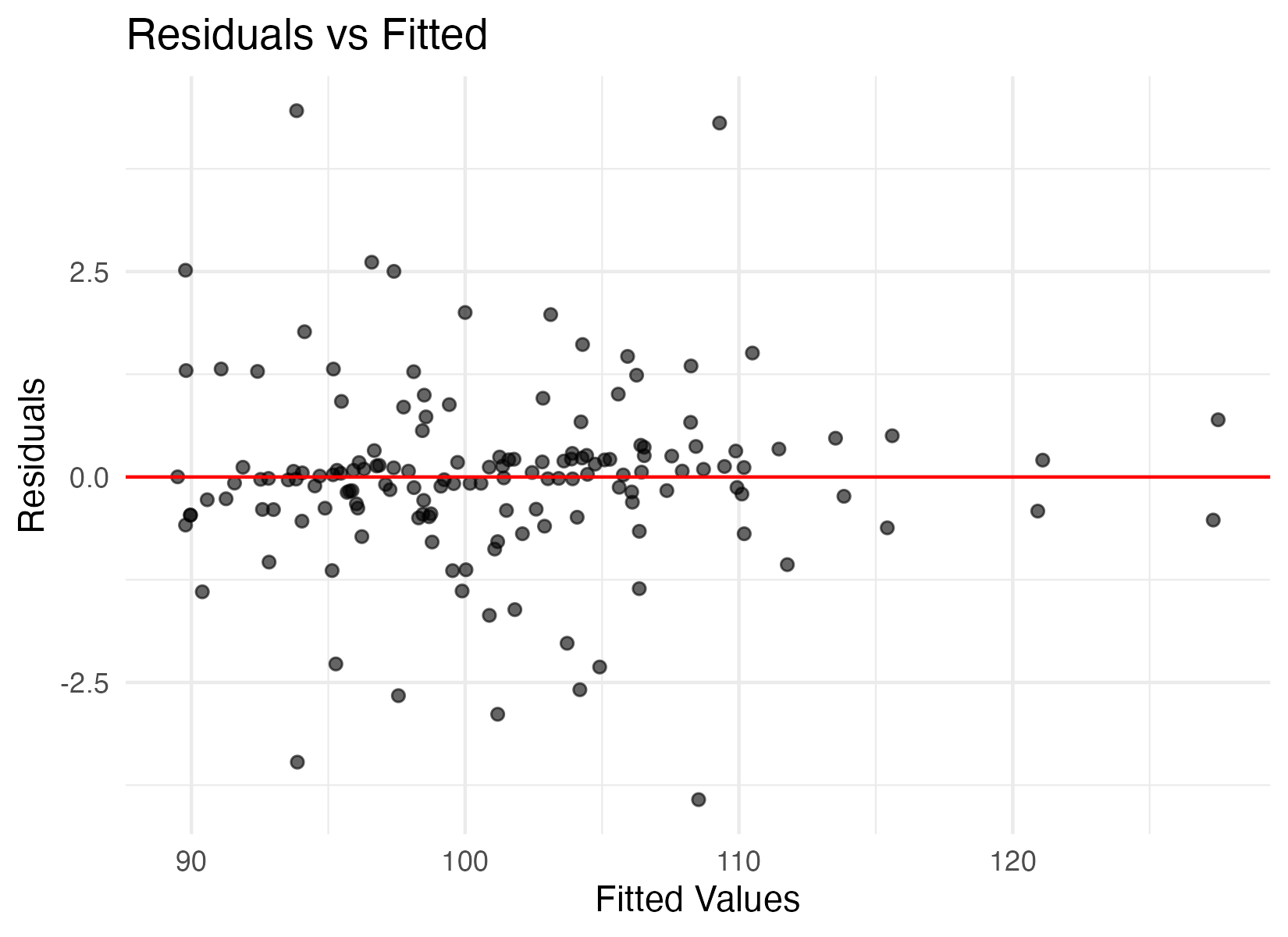


## QQ Plot


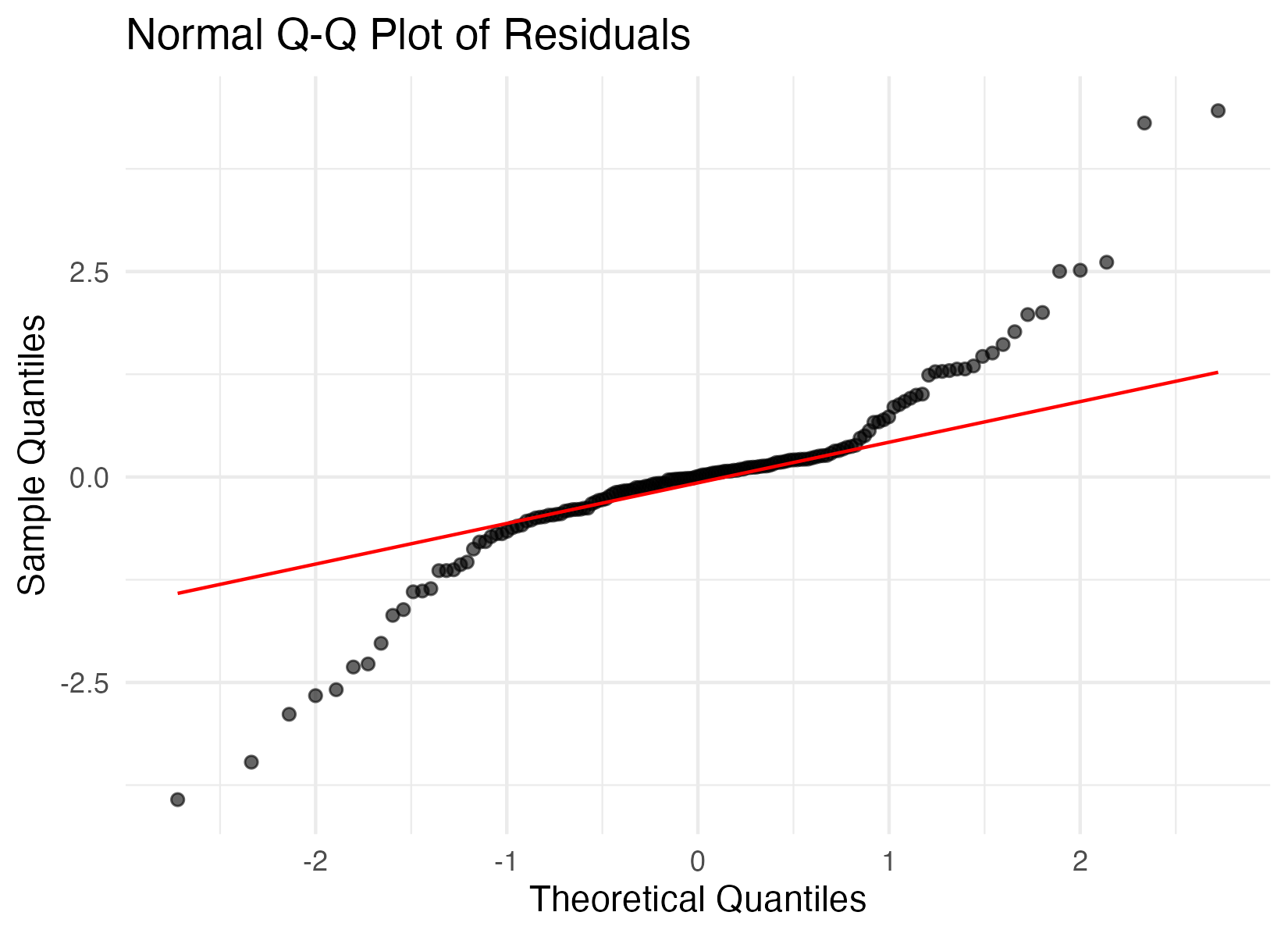


## Within-group change (baseline to follow-up)

| contrast | group | estimate | SE | df | lower.CL | upper.CL | t.ratio | p.value | effect_size |
| --- | --- | --- | --- | --- | --- | --- | --- | --- | --- |
| followup - baseline | C | 0.307 | 0.249 | 143 | -0.185 | 0.799 | 1.233 | 0.220 | 0.043 |
| followup - baseline | S | -0.180 | 0.255 | 143 | -0.684 | 0.325 | -0.704 | 0.483 | -0.025 |

## Between-group difference in change (interaction)

| timepoint_revpairwise | group_revpairwise | estimate | SE | df | lower.CL | upper.CL | t.ratio | p.value | effect_size |
| --- | --- | --- | --- | --- | --- | --- | --- | --- | --- |
| followup - baseline | S - C | -0.486 | 0.356 | 143 | -1.191 | 0.218 | -1.365 | 0.174 | -0.068 |

## Adjusted Means Over Time (with 95% CI)


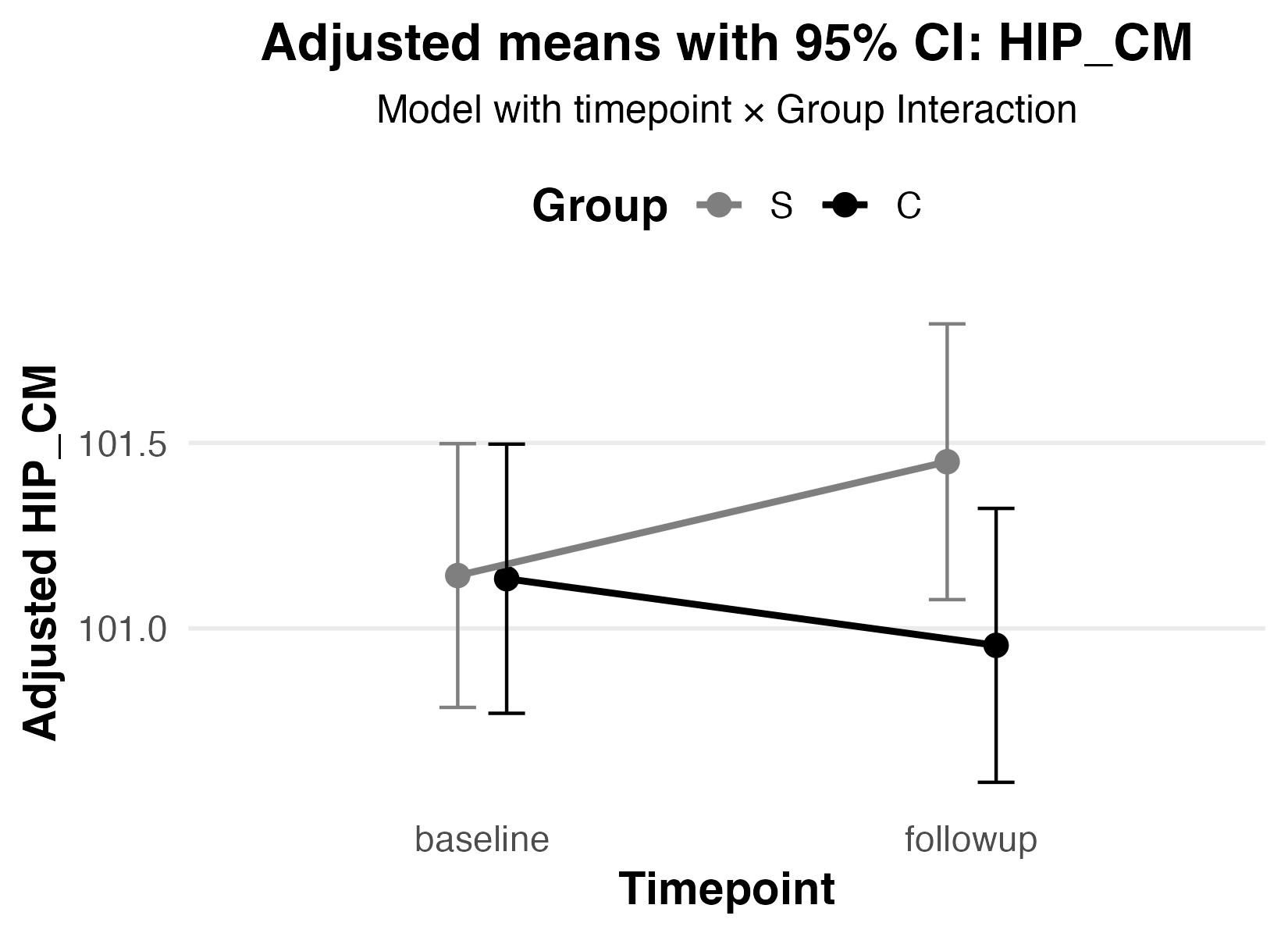


# Outcome: whr

## Number of Participants Included: 79

## Distribution of DV at Baseline


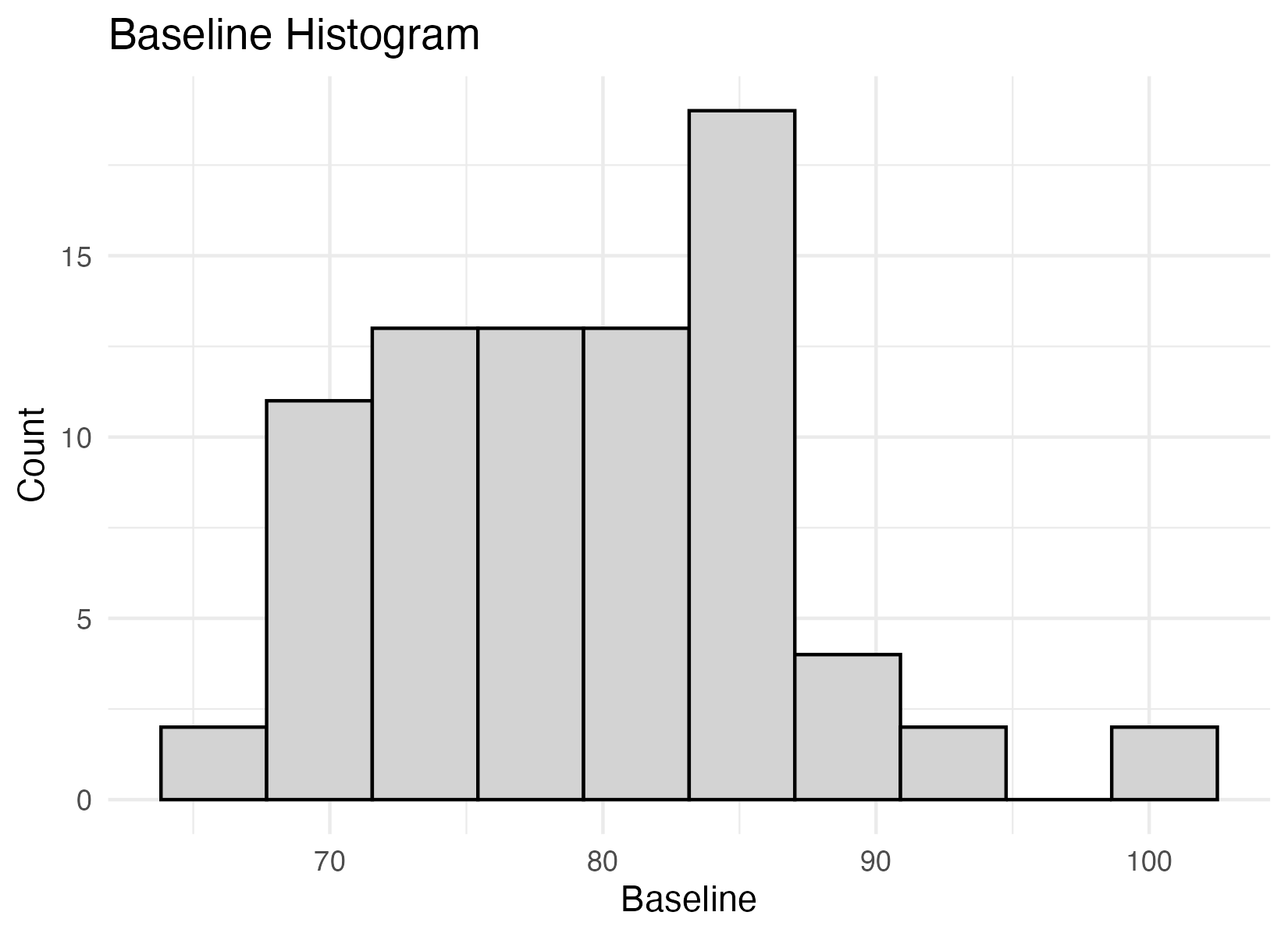


## Fitted vs Residuals


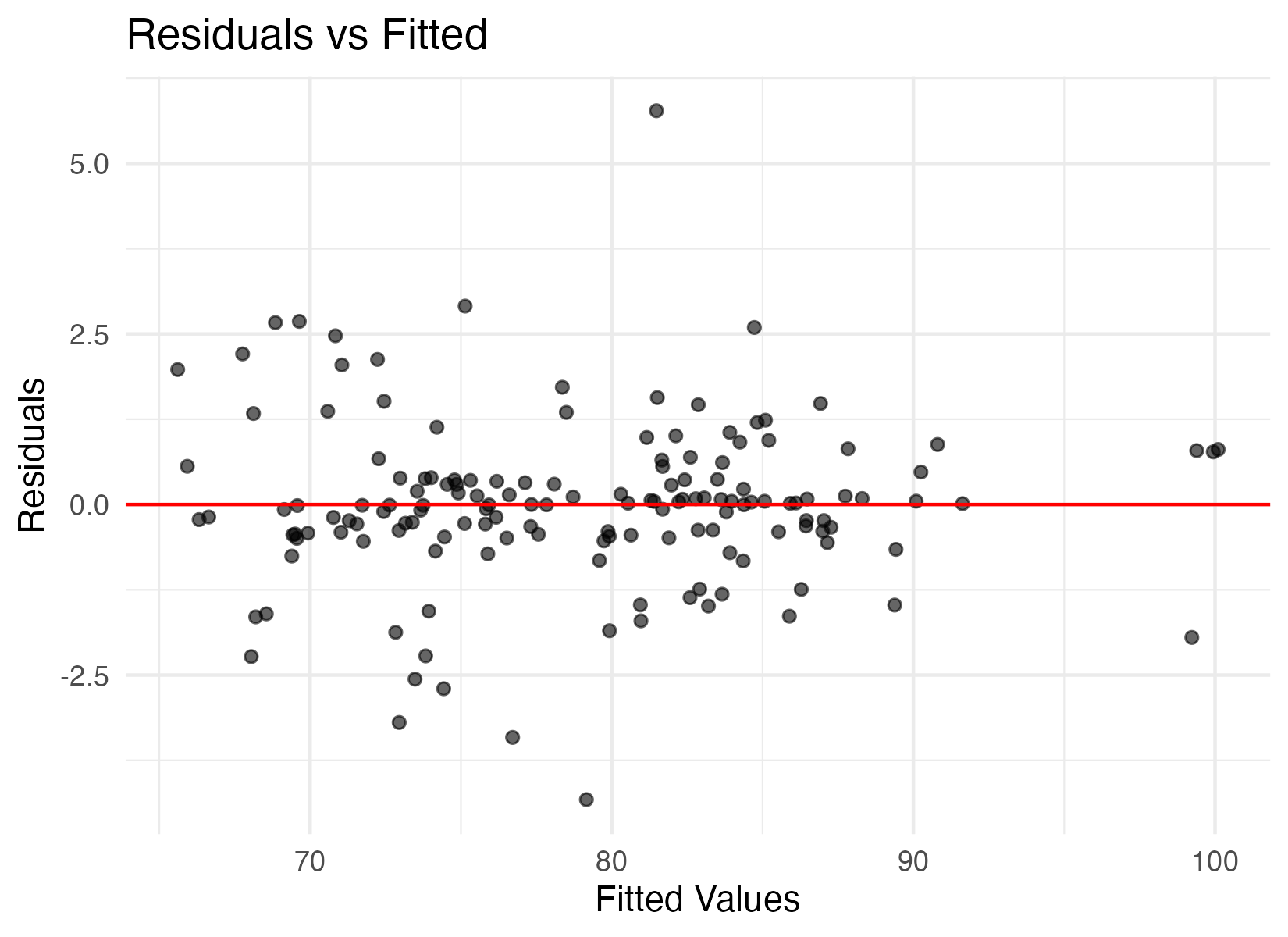


## QQ Plot


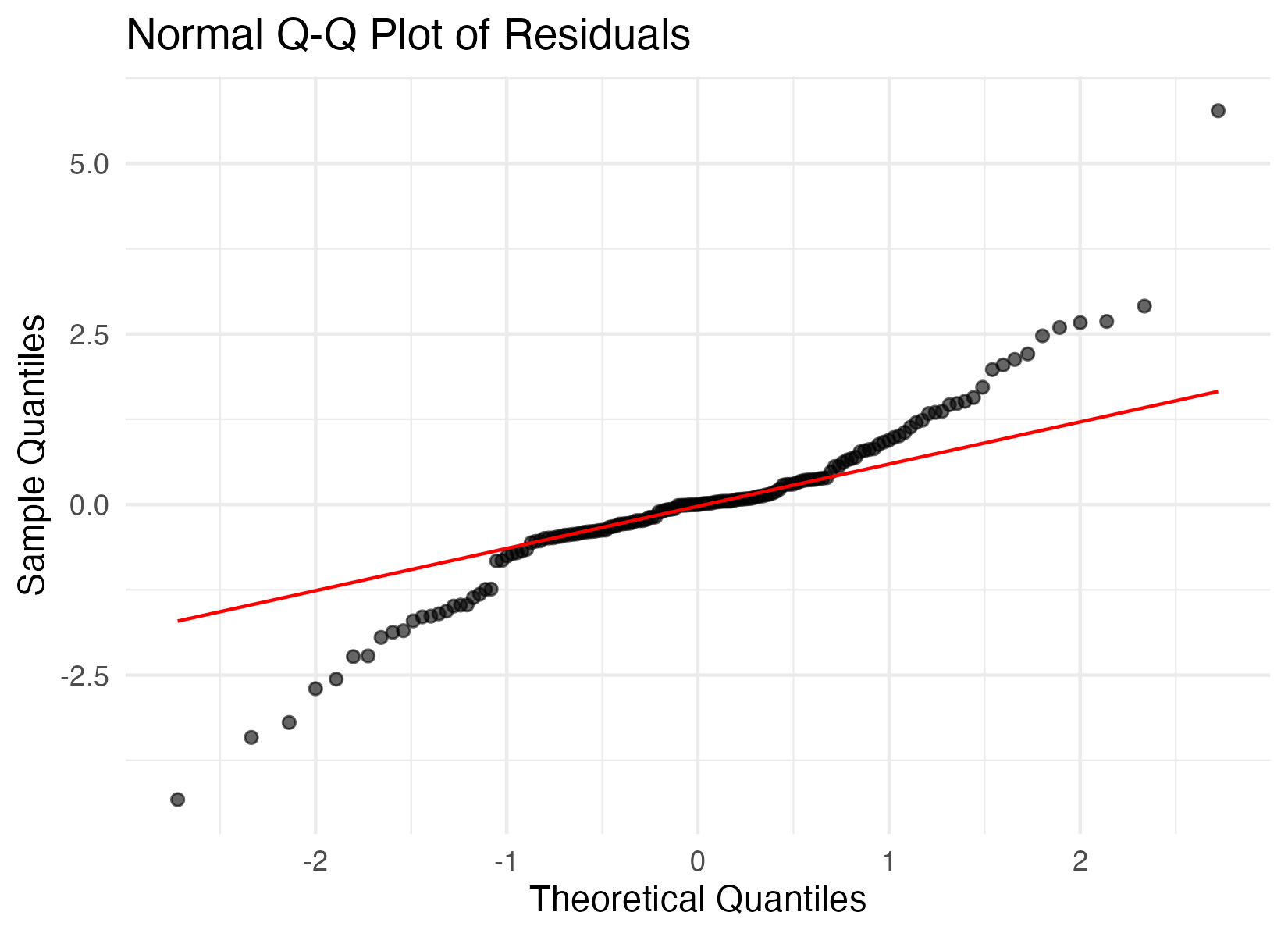


## Within-group change (baseline to follow-up)

| contrast | group | estimate | SE | df | lower.CL | upper.CL | t.ratio | p.value | effect_size |
| --- | --- | --- | --- | --- | --- | --- | --- | --- | --- |
| followup - baseline | C | -1.380 | 0.276 | 143 | -1.926 | -0.834 | -4.998 | <0.001 | -0.190 |
| followup - baseline | S | -0.711 | 0.283 | 143 | -1.271 | -0.151 | -2.511 | 0.013 | -0.098 |

## Between-group difference in change (interaction)

| timepoint_revpairwise | group_revpairwise | estimate | SE | df | lower.CL | upper.CL | t.ratio | p.value | effect_size |
| --- | --- | --- | --- | --- | --- | --- | --- | --- | --- |
| followup - baseline | S - C | 0.669 | 0.395 | 143 | -0.113 | 1.451 | 1.692 | 0.093 | 0.092 |

## Adjusted Means Over Time (with 95% CI)


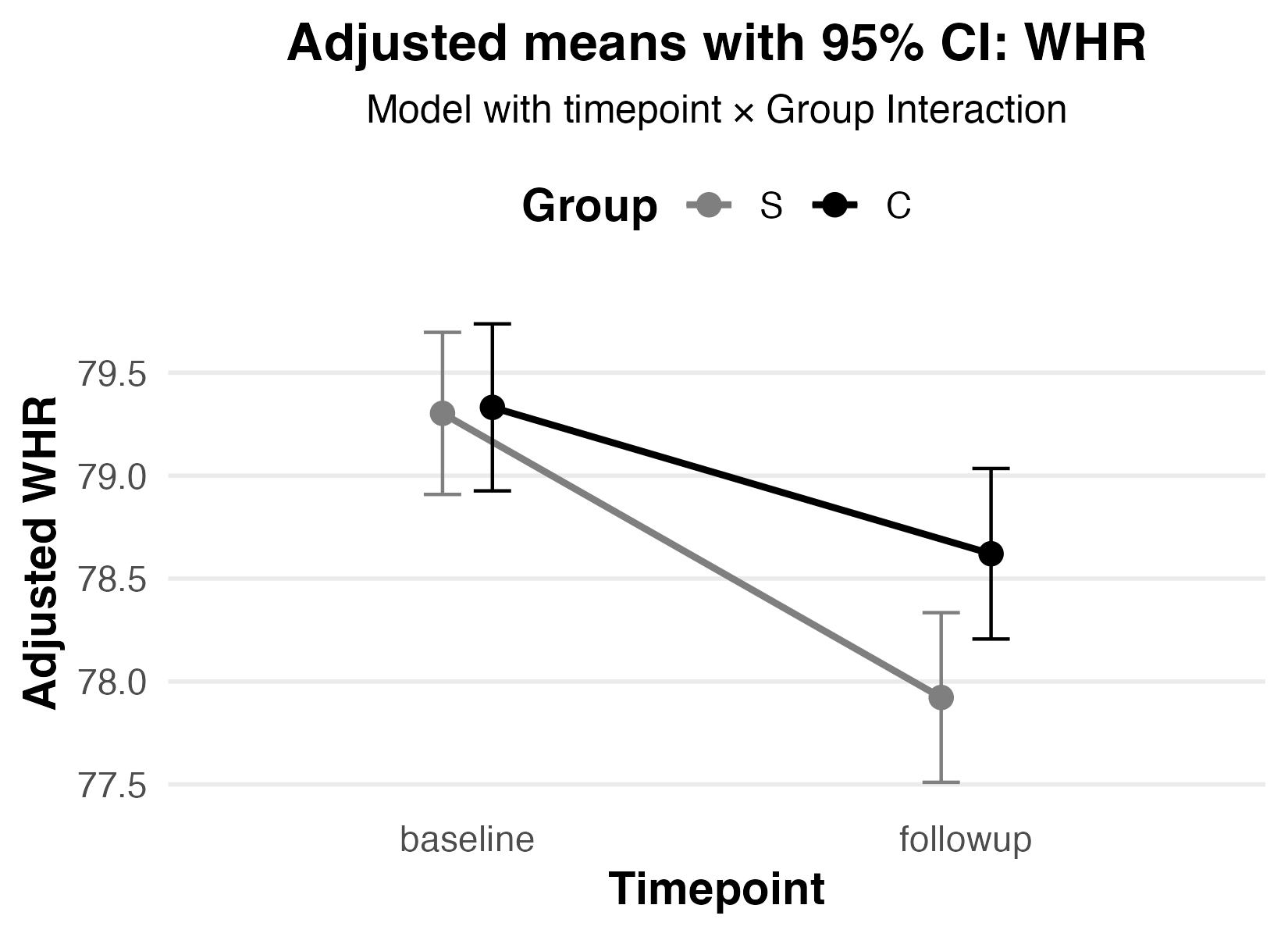


# Outcome: bonemass_kg

## Number of Participants Included: 79

## Distribution of DV at Baseline


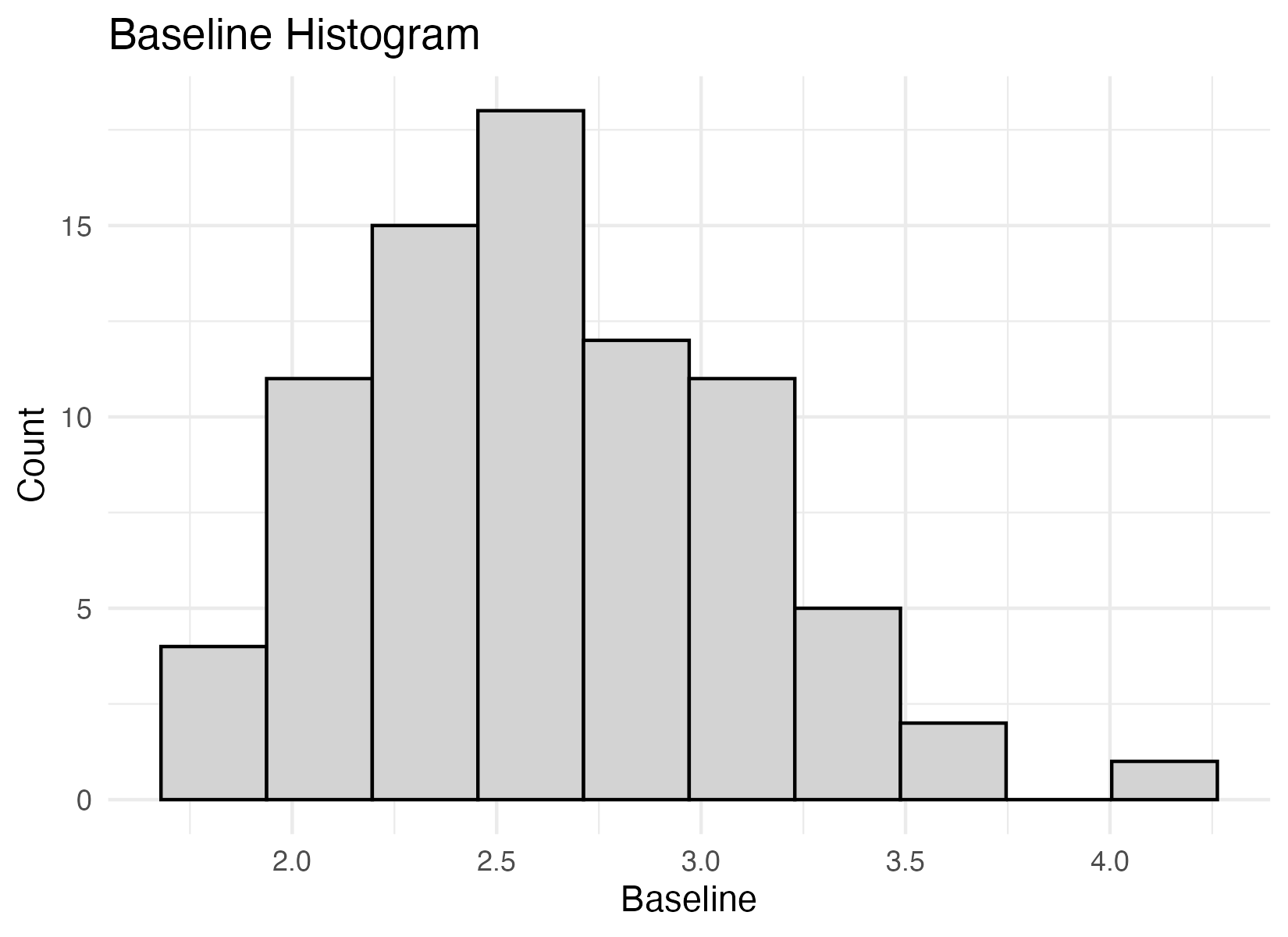


## Fitted vs Residuals


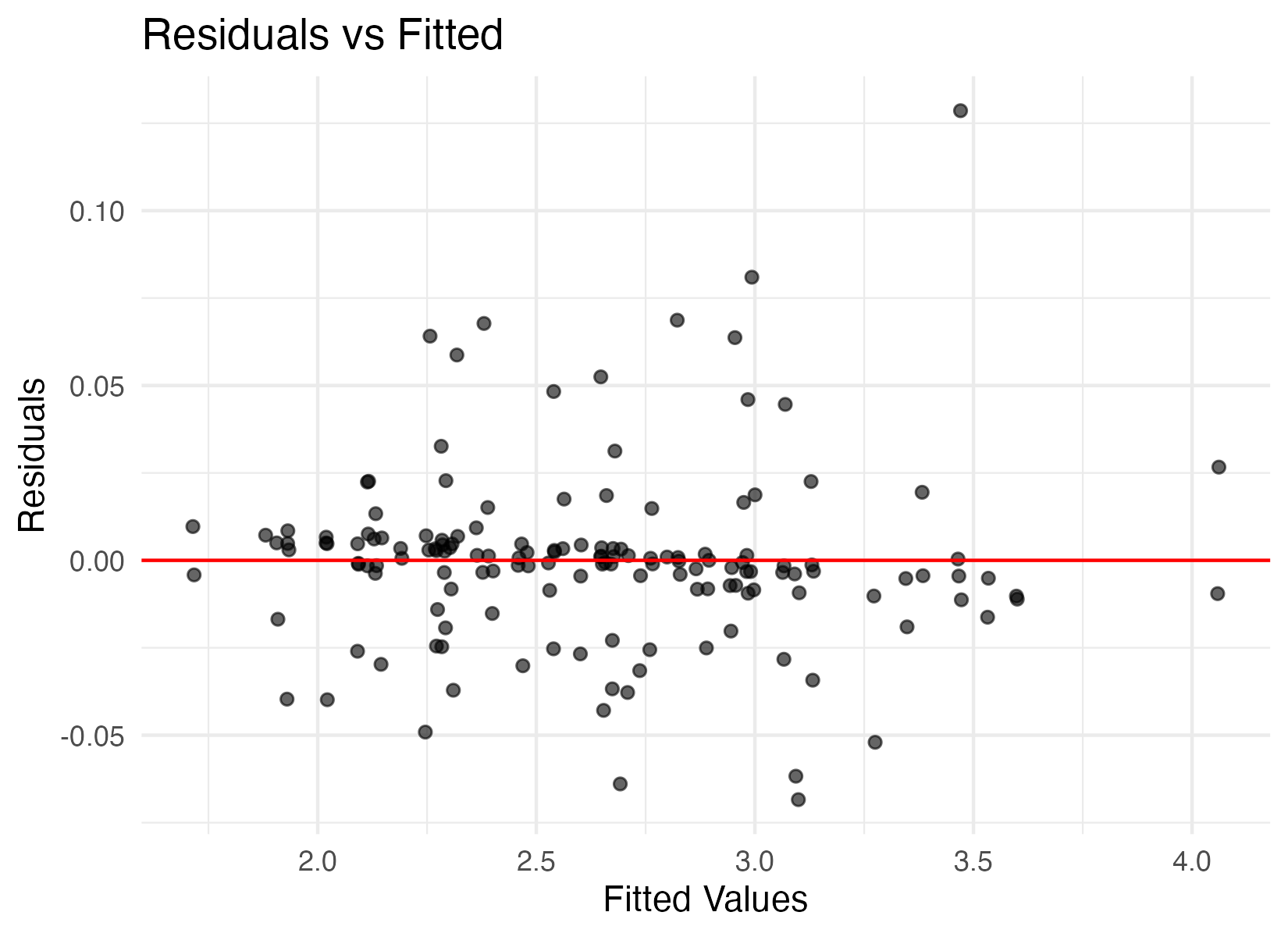


## QQ Plot


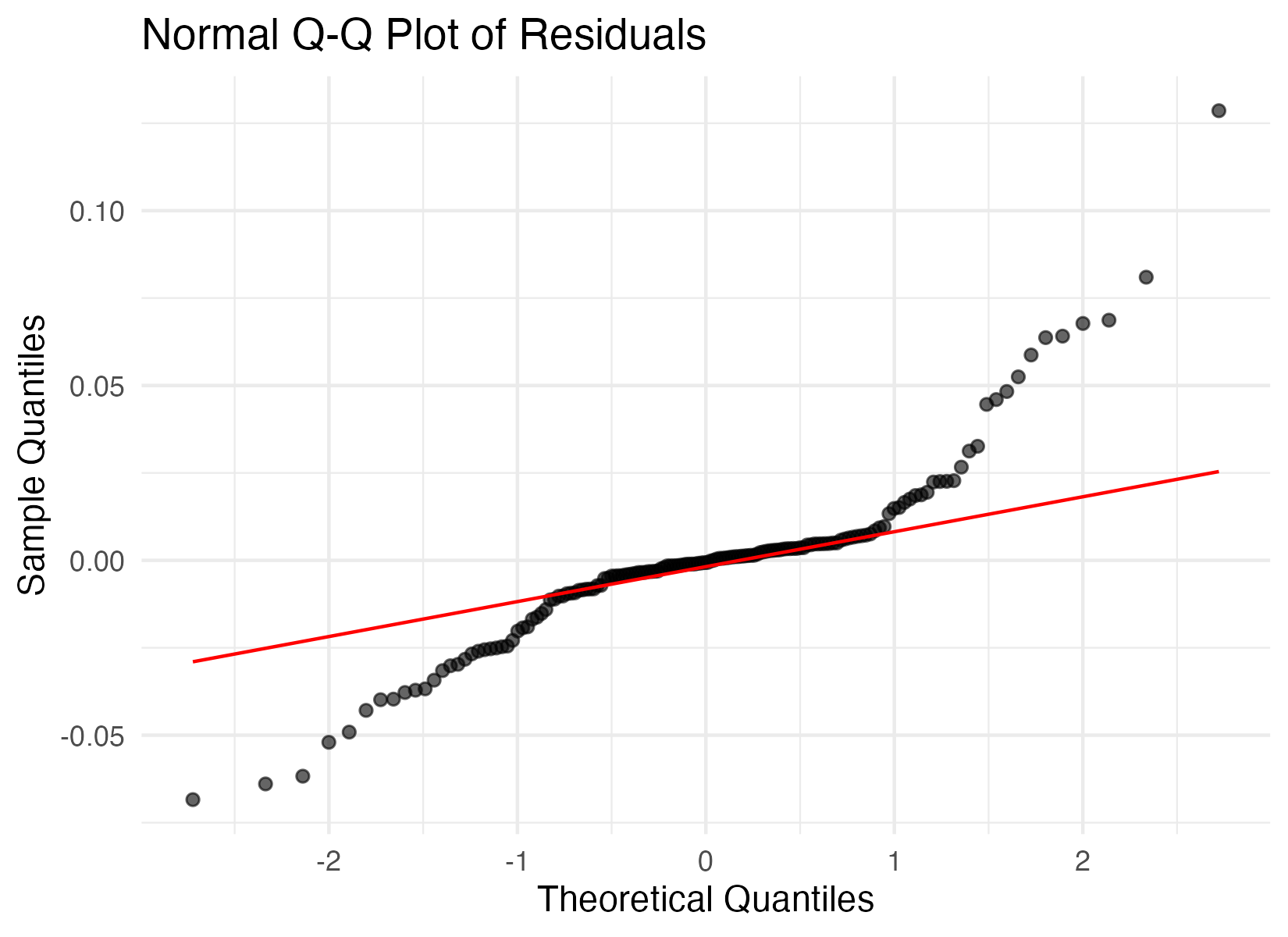


## Within-group change (baseline to follow-up)

| contrast | group | estimate | SE | df | lower.CL | upper.CL | t.ratio | p.value | effect_size |
| --- | --- | --- | --- | --- | --- | --- | --- | --- | --- |
| followup - baseline | C | 0.003 | 0.006 | 143 | -0.009 | 0.014 | 0.488 | 0.626 | 0.006 |
| followup - baseline | S | -0.002 | 0.006 | 143 | -0.014 | 0.010 | -0.313 | 0.754 | -0.004 |

## Between-group difference in change (interaction)

| timepoint_revpairwise | group_revpairwise | estimate | SE | df | lower.CL | upper.CL | t.ratio | p.value | effect_size |
| --- | --- | --- | --- | --- | --- | --- | --- | --- | --- |
| followup - baseline | S - C | -0.005 | 0.008 | 143 | -0.021 | 0.012 | -0.566 | 0.573 | -0.01 |

## Adjusted Means Over Time (with 95% CI)


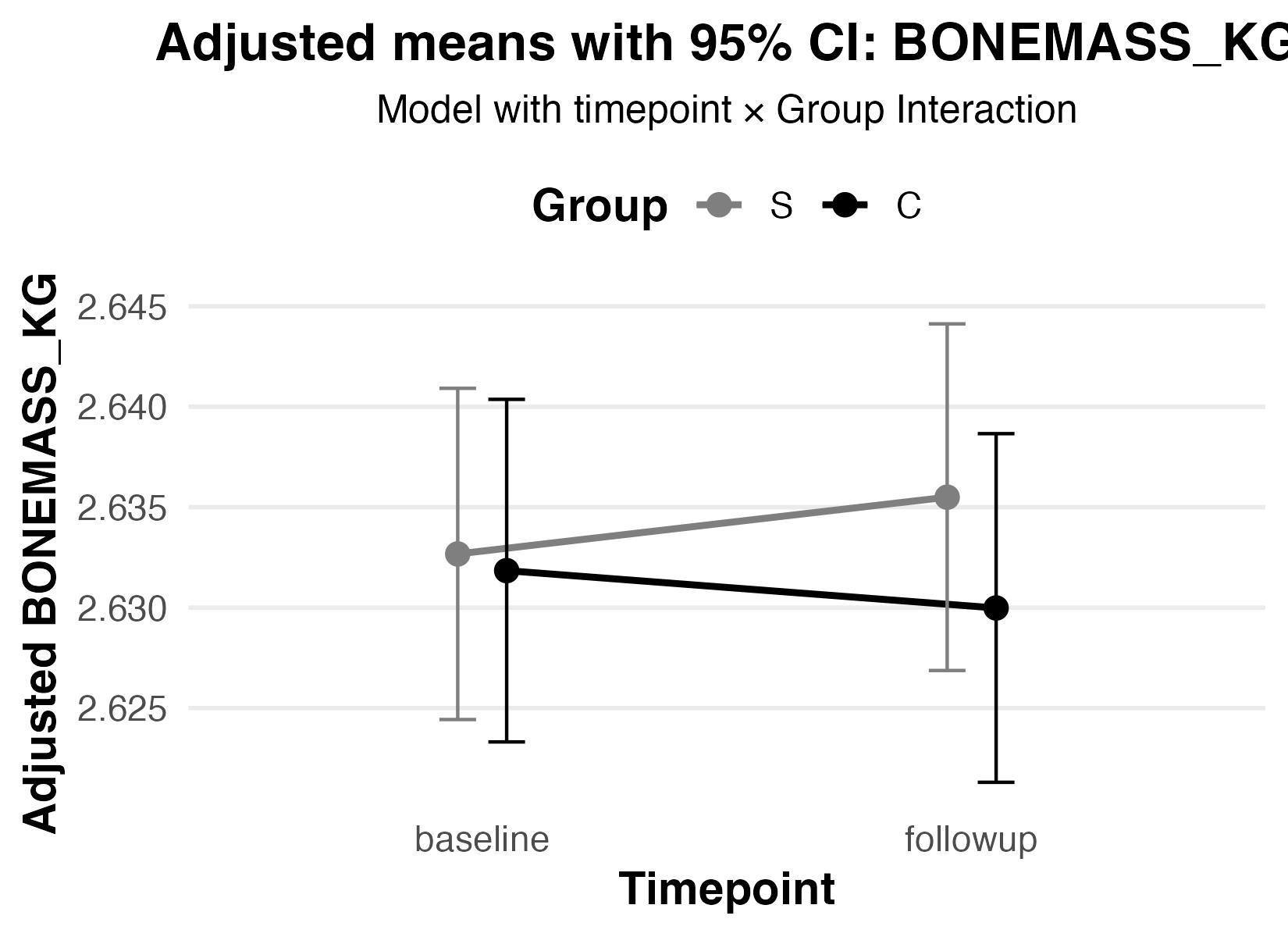


# Outcome: fatmass_kg

## Number of Participants Included: 79

## Distribution of DV at Baseline


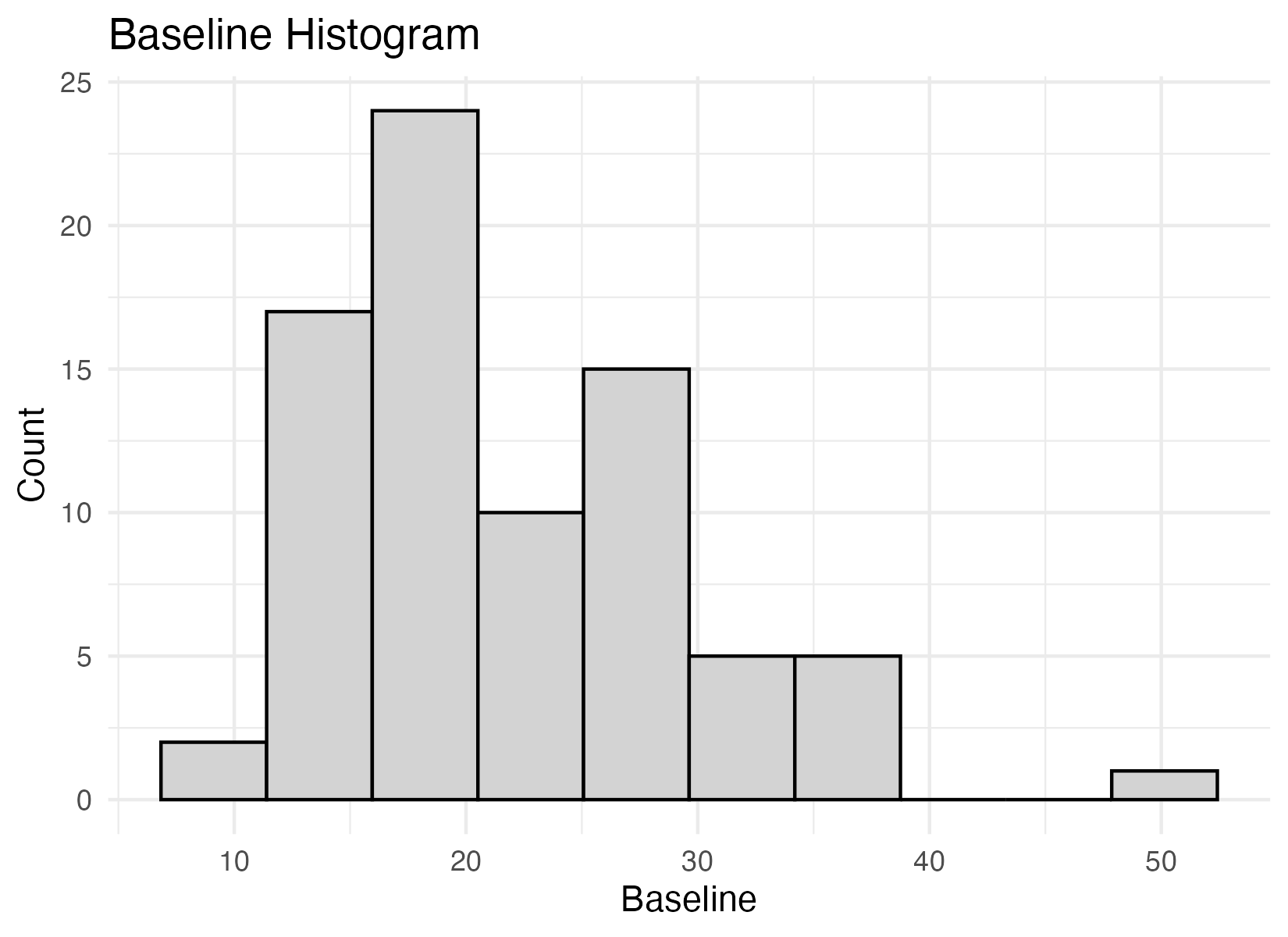


## Fitted vs Residuals


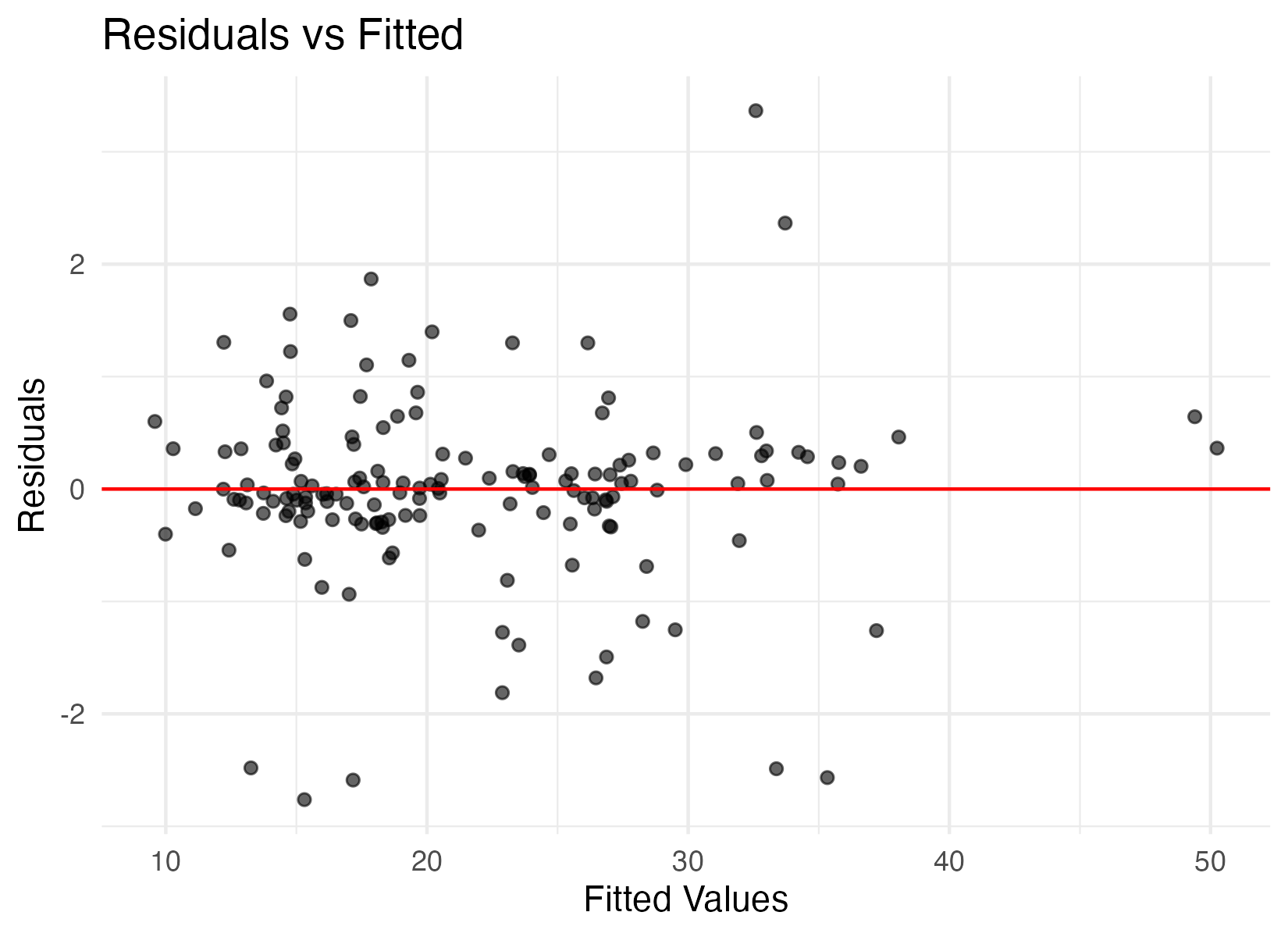


## QQ Plot


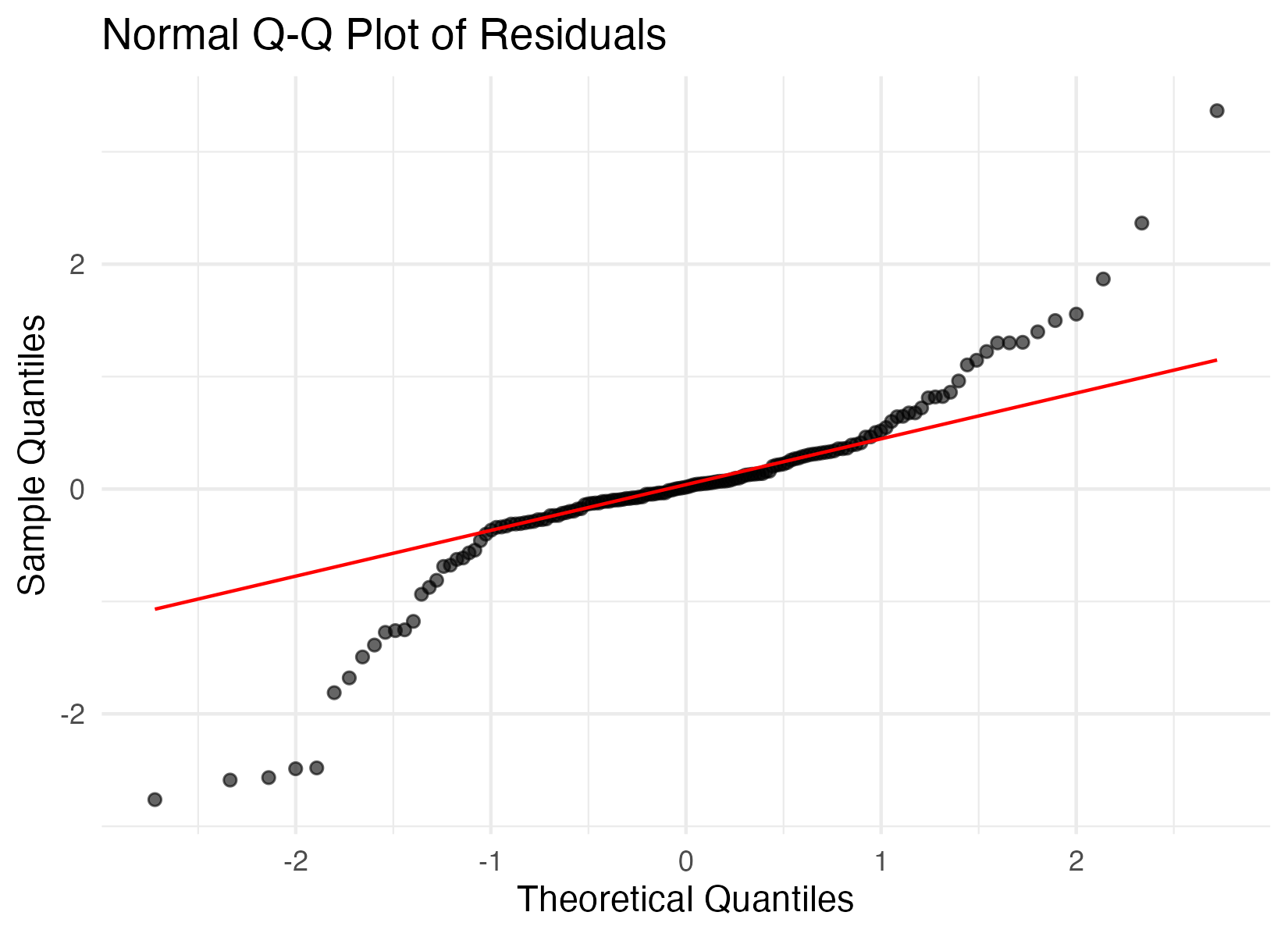


## Within-group change (baseline to follow-up)

| contrast | group | estimate | SE | df | lower.CL | upper.CL | t.ratio | p.value | effect_size |
| --- | --- | --- | --- | --- | --- | --- | --- | --- | --- |
| followup - baseline | C | -0.405 | 0.184 | 143 | -0.769 | -0.042 | -2.204 | 0.029 | -0.054 |
| followup - baseline | S | -0.852 | 0.189 | 143 | -1.225 | -0.479 | -4.517 | <0.001 | -0.113 |

## Between-group difference in change (interaction)

| timepoint_revpairwise | group_revpairwise | estimate | SE | df | lower.CL | upper.CL | t.ratio | p.value | effect_size |
| --- | --- | --- | --- | --- | --- | --- | --- | --- | --- |
| followup - baseline | S - C | -0.447 | 0.263 | 143 | -0.968 | 0.074 | -1.696 | 0.092 | -0.059 |

## Adjusted Means Over Time (with 95% CI)


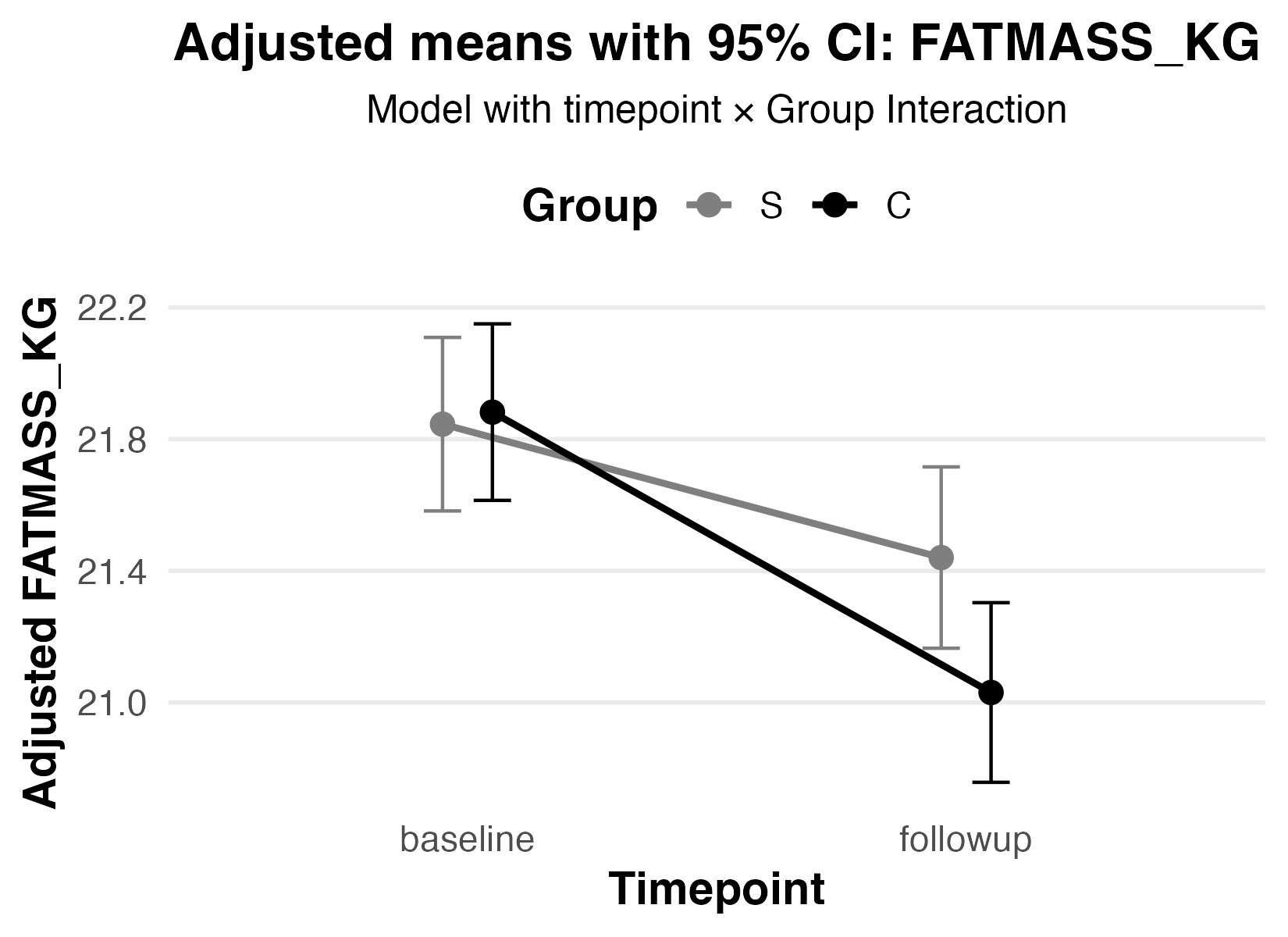


# Outcome: leanmass_kg

## Number of Participants Included: 79

## Distribution of DV at Baseline


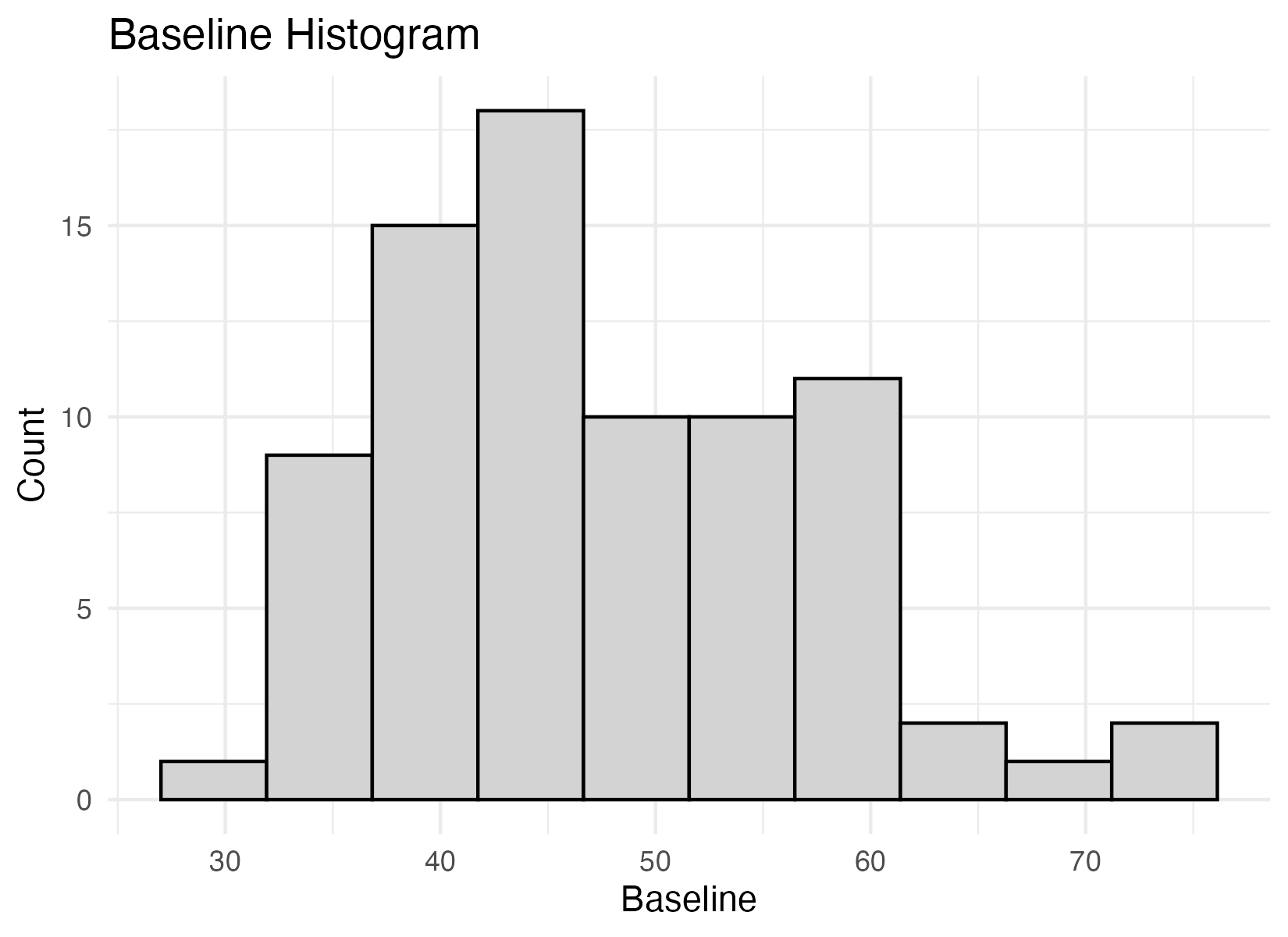


## Fitted vs Residuals


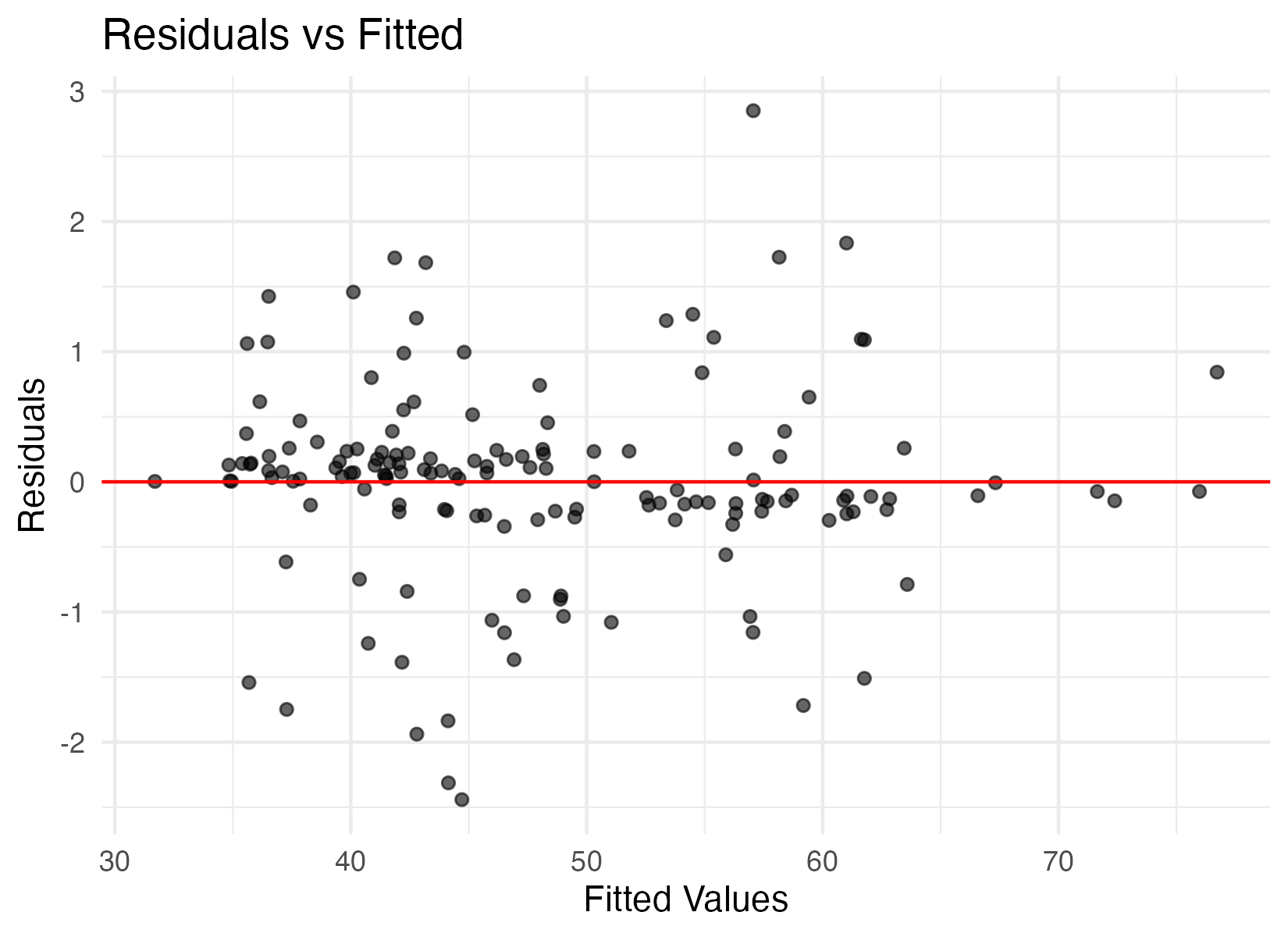


## QQ Plot


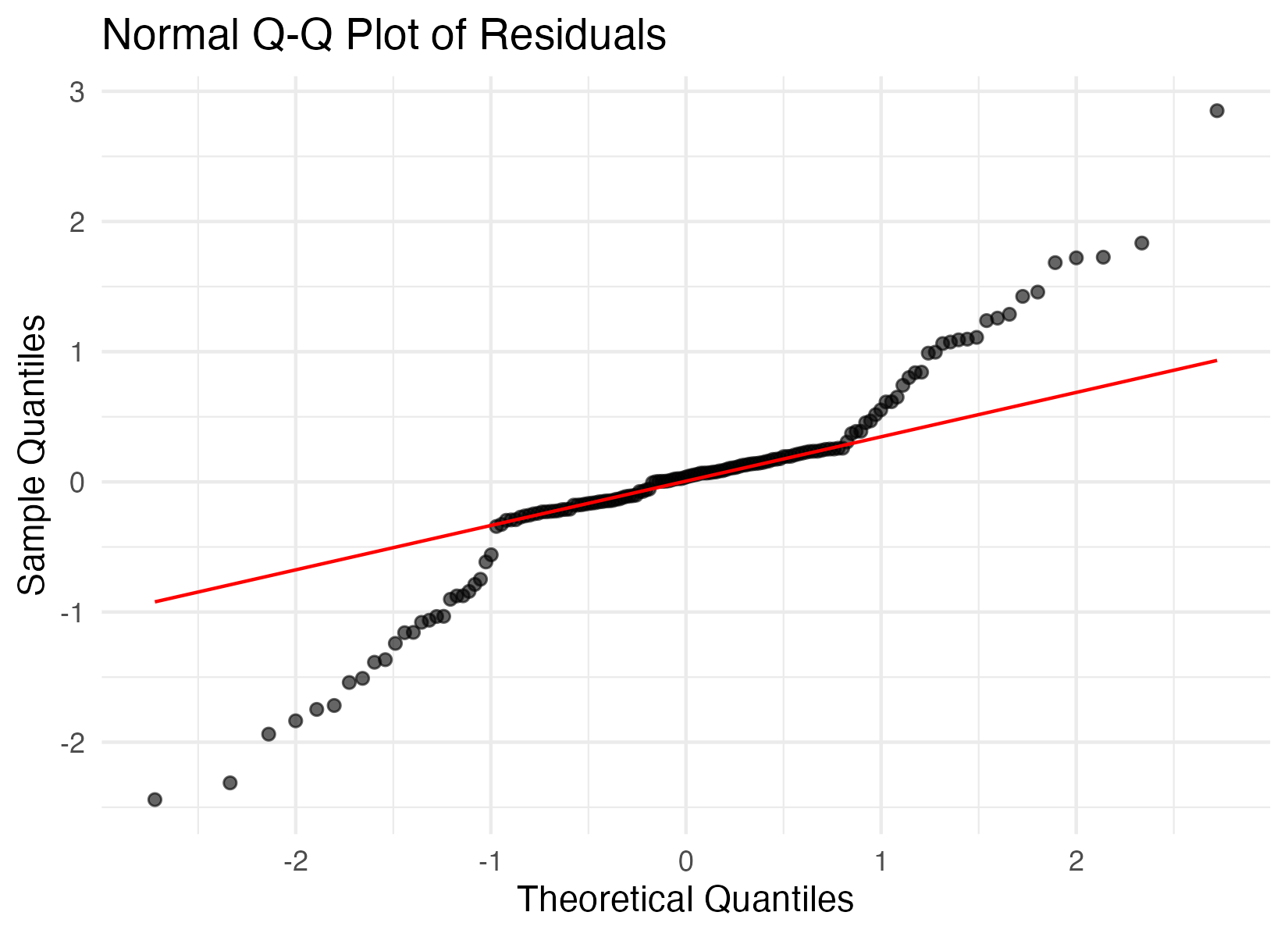


## Within-group change (baseline to follow-up)

| contrast | group | estimate | SE | df | lower.CL | upper.CL | t.ratio | p.value | effect_size |
| --- | --- | --- | --- | --- | --- | --- | --- | --- | --- |
| followup - baseline | C | 0.745 | 0.174 | 143 | 0.402 | 1.089 | 4.286 | <0.001 | 0.079 |
| followup - baseline | S | 0.737 | 0.178 | 143 | 0.385 | 1.090 | 4.135 | <0.001 | 0.078 |

## Between-group difference in change (interaction)

| timepoint_revpairwise | group_revpairwise | estimate | SE | df | lower.CL | upper.CL | t.ratio | p.value | effect_size |
| --- | --- | --- | --- | --- | --- | --- | --- | --- | --- |
| followup - baseline | S - C | -0.008 | 0.249 | 143 | -0.5 | 0.484 | -0.033 | 0.974 | -0.001 |

## Adjusted Means Over Time (with 95% CI)


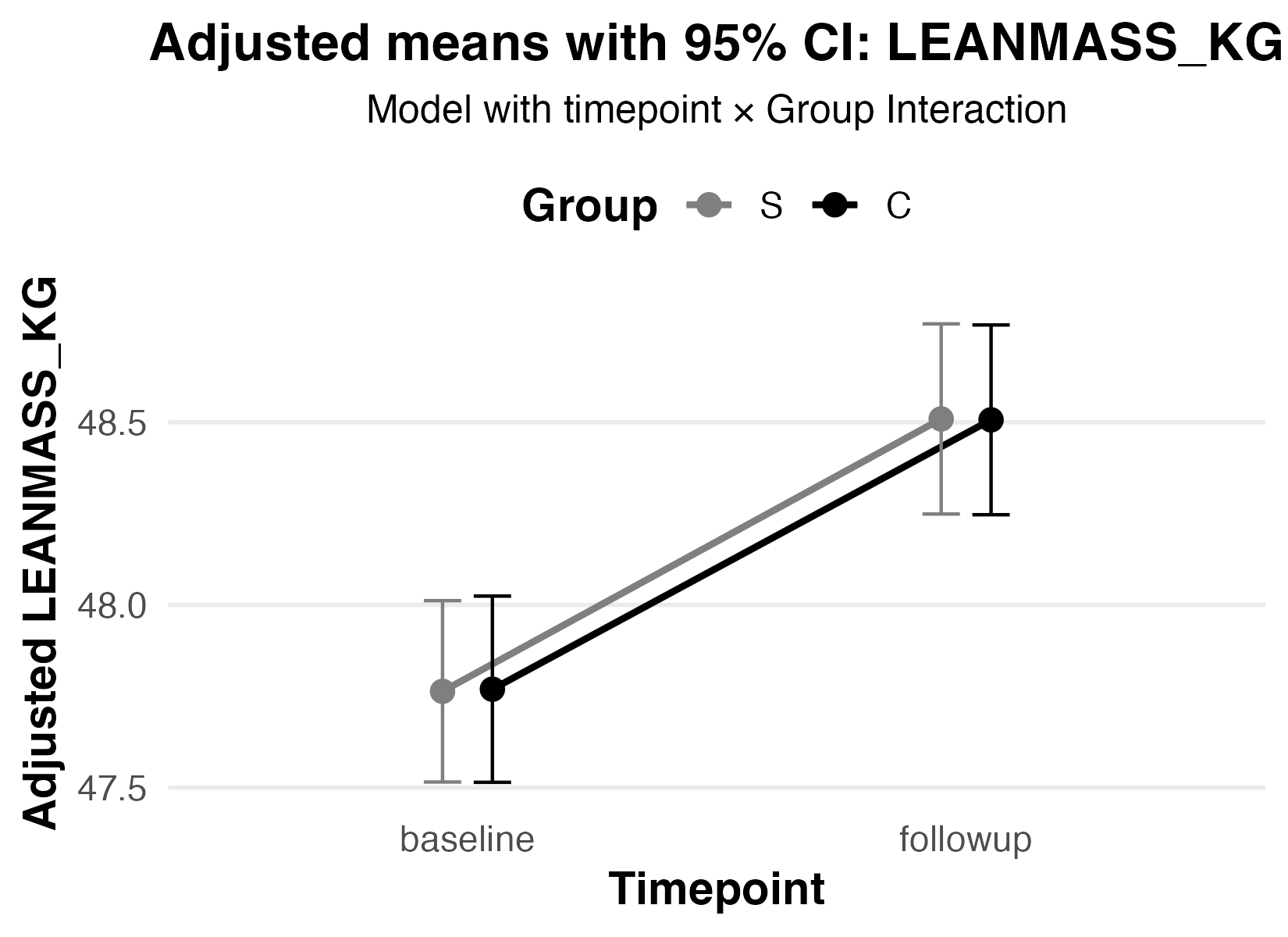


# Outcome: fatmass_percent

## Number of Participants Included: 79

## Distribution of DV at Baseline


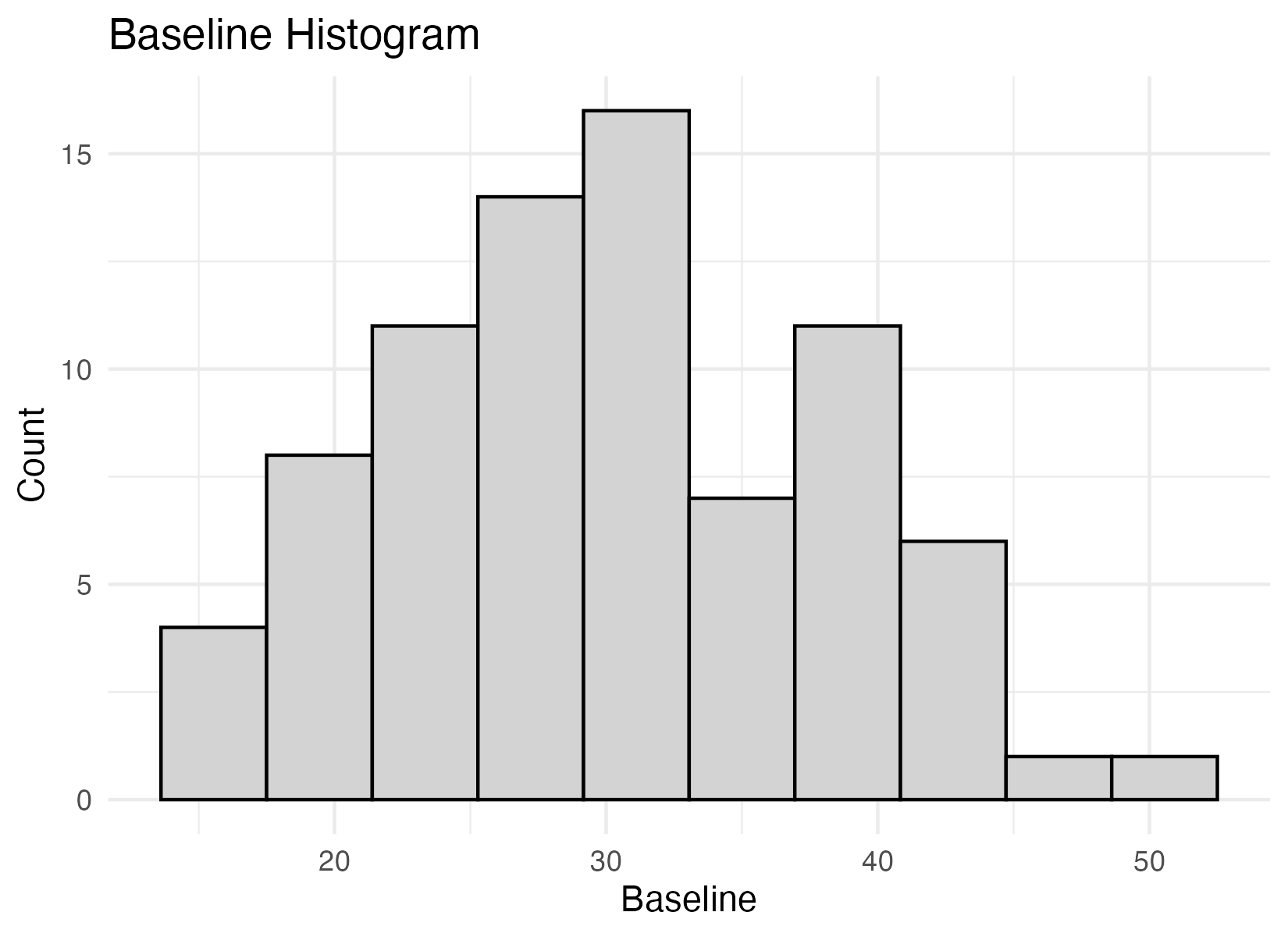


## Fitted vs Residuals


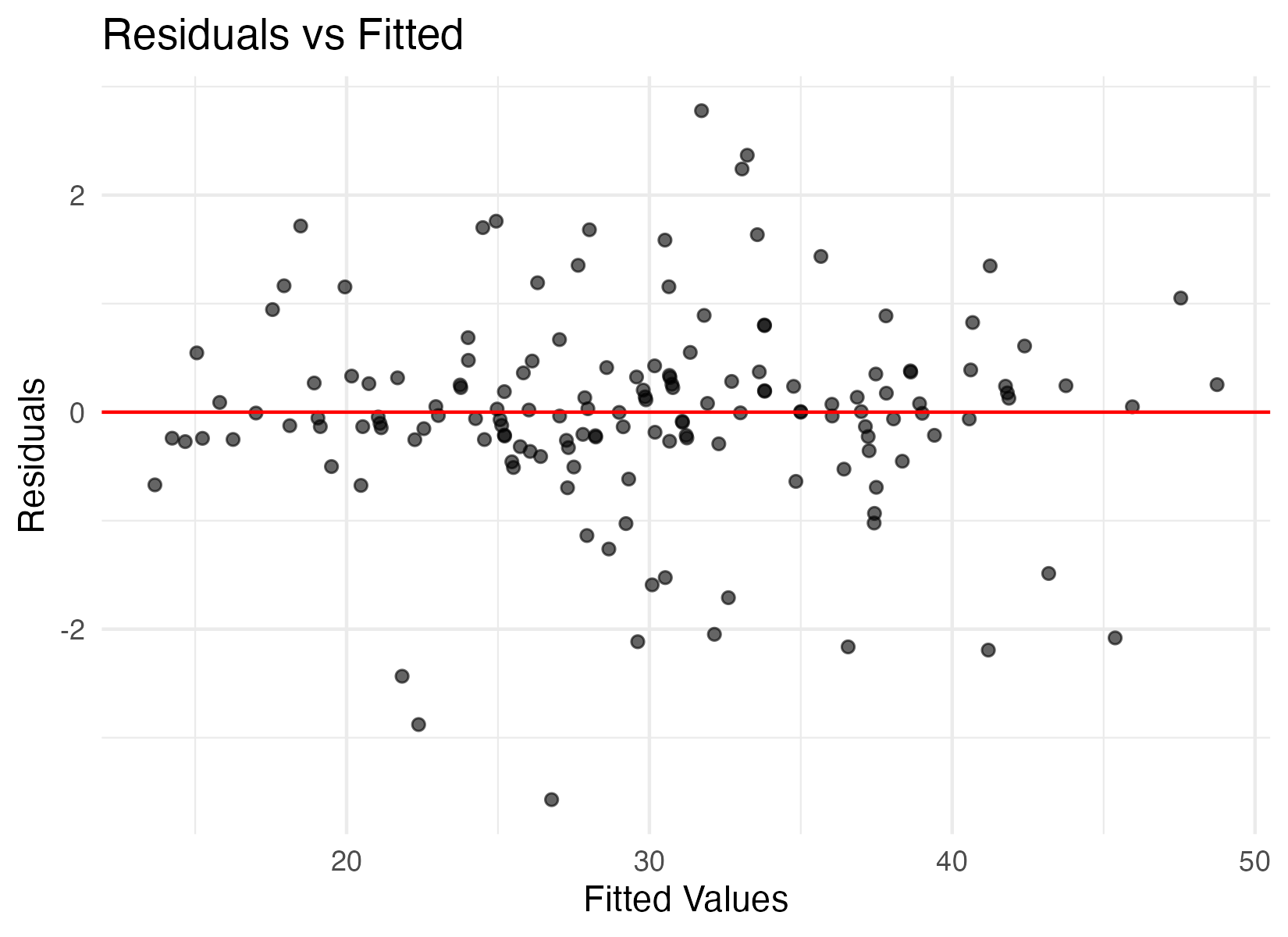


## QQ Plot


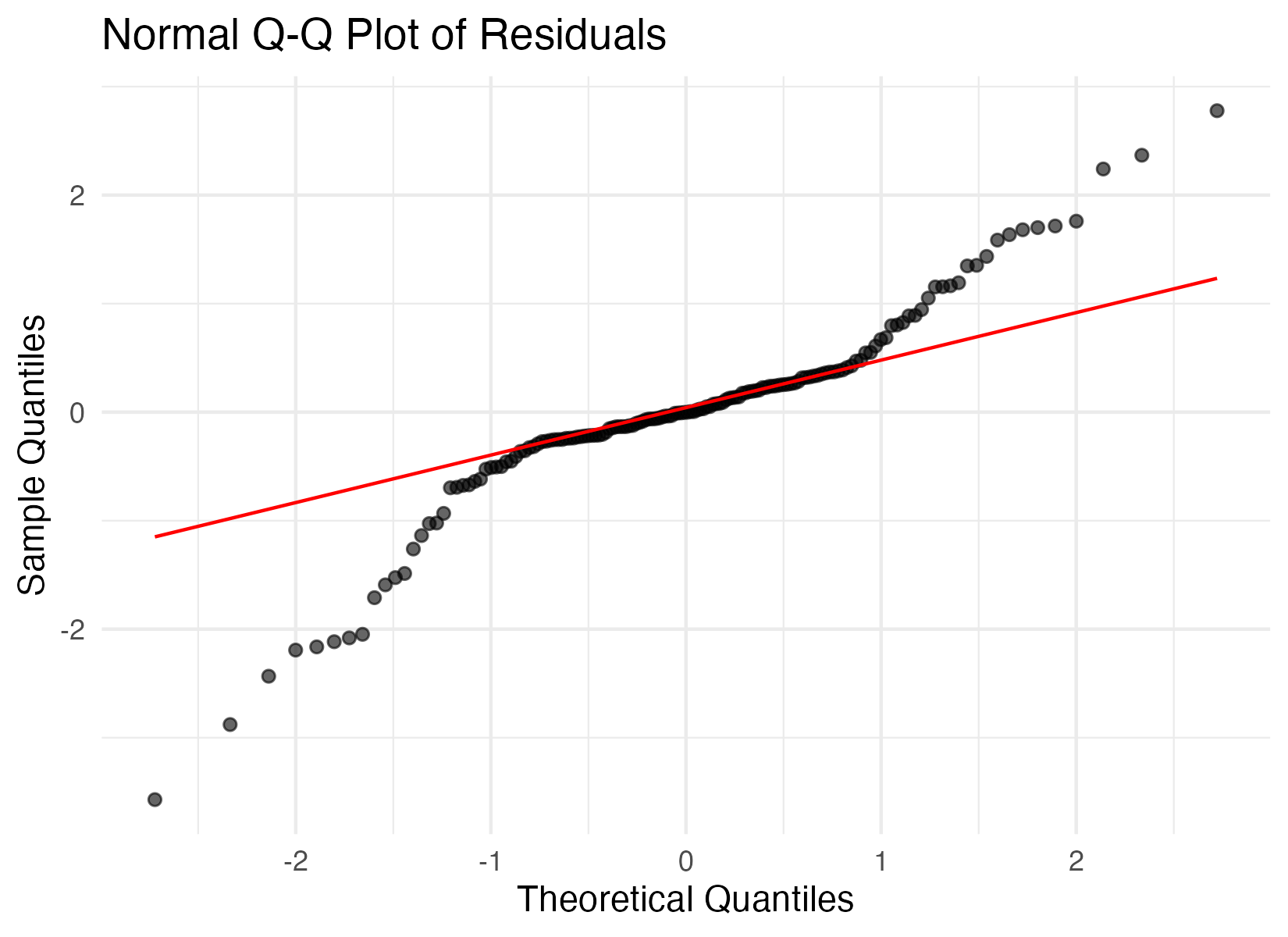


## Within-group change (baseline to follow-up)

| contrast | group | estimate | SE | df | lower.CL | upper.CL | t.ratio | p.value | effect_size |
| --- | --- | --- | --- | --- | --- | --- | --- | --- | --- |
| followup - baseline | C | -0.570 | 0.206 | 143 | -0.976 | -0.163 | -2.770 | 0.006 | -0.075 |
| followup - baseline | S | -1.198 | 0.211 | 143 | -1.615 | -0.781 | -5.679 | <0.001 | -0.157 |

## Between-group difference in change (interaction)

| timepoint_revpairwise | group_revpairwise | estimate | SE | df | lower.CL | upper.CL | t.ratio | p.value | effect_size |
| --- | --- | --- | --- | --- | --- | --- | --- | --- | --- |
| followup - baseline | S - C | -0.628 | 0.295 | 143 | -1.21 | -0.046 | -2.132 | 0.035 | -0.082 |

## Adjusted Means Over Time (with 95% CI)


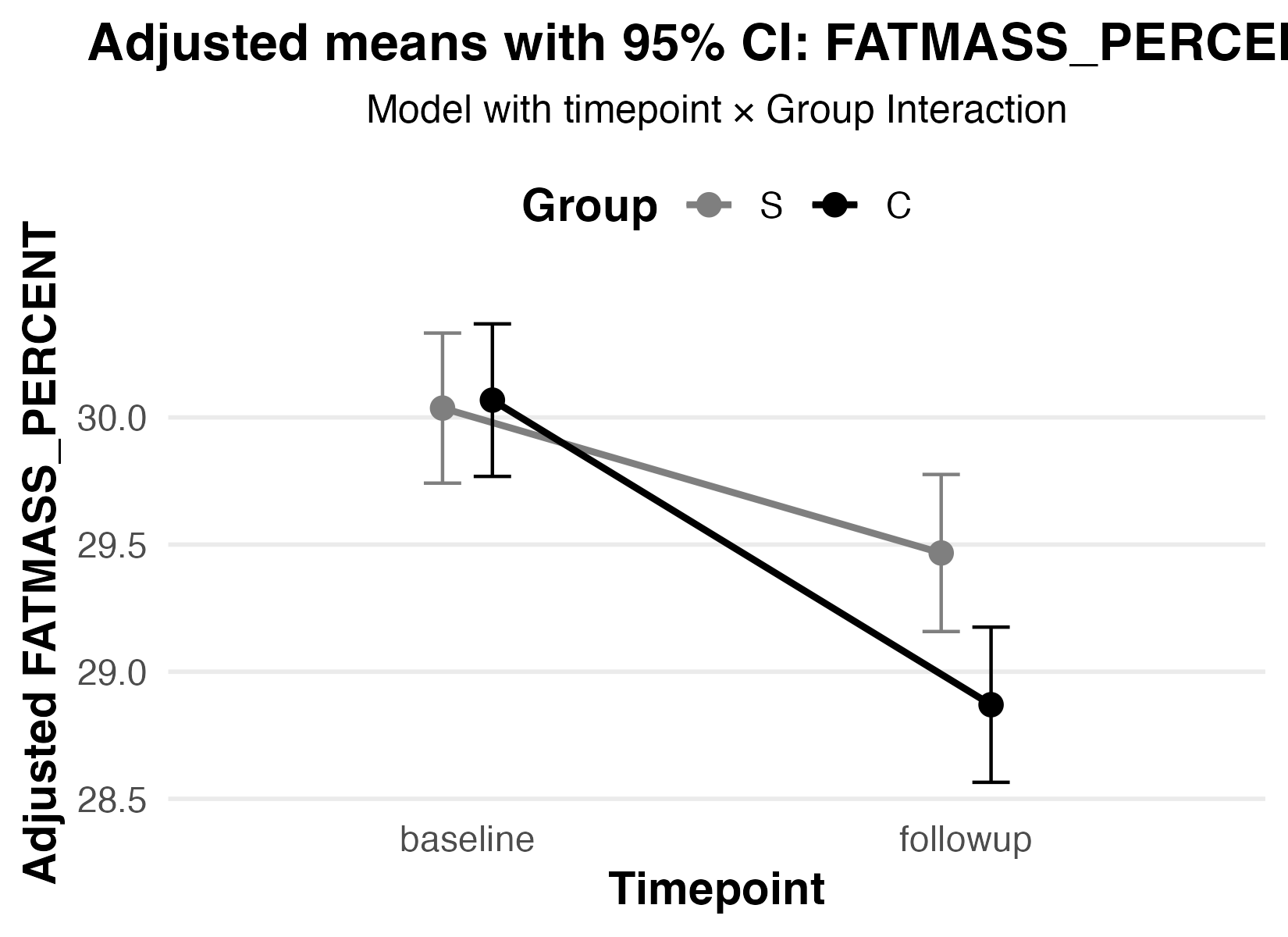


# Outcome: leanmass_percent

## Number of Participants Included: 79

## Distribution of DV at Baseline


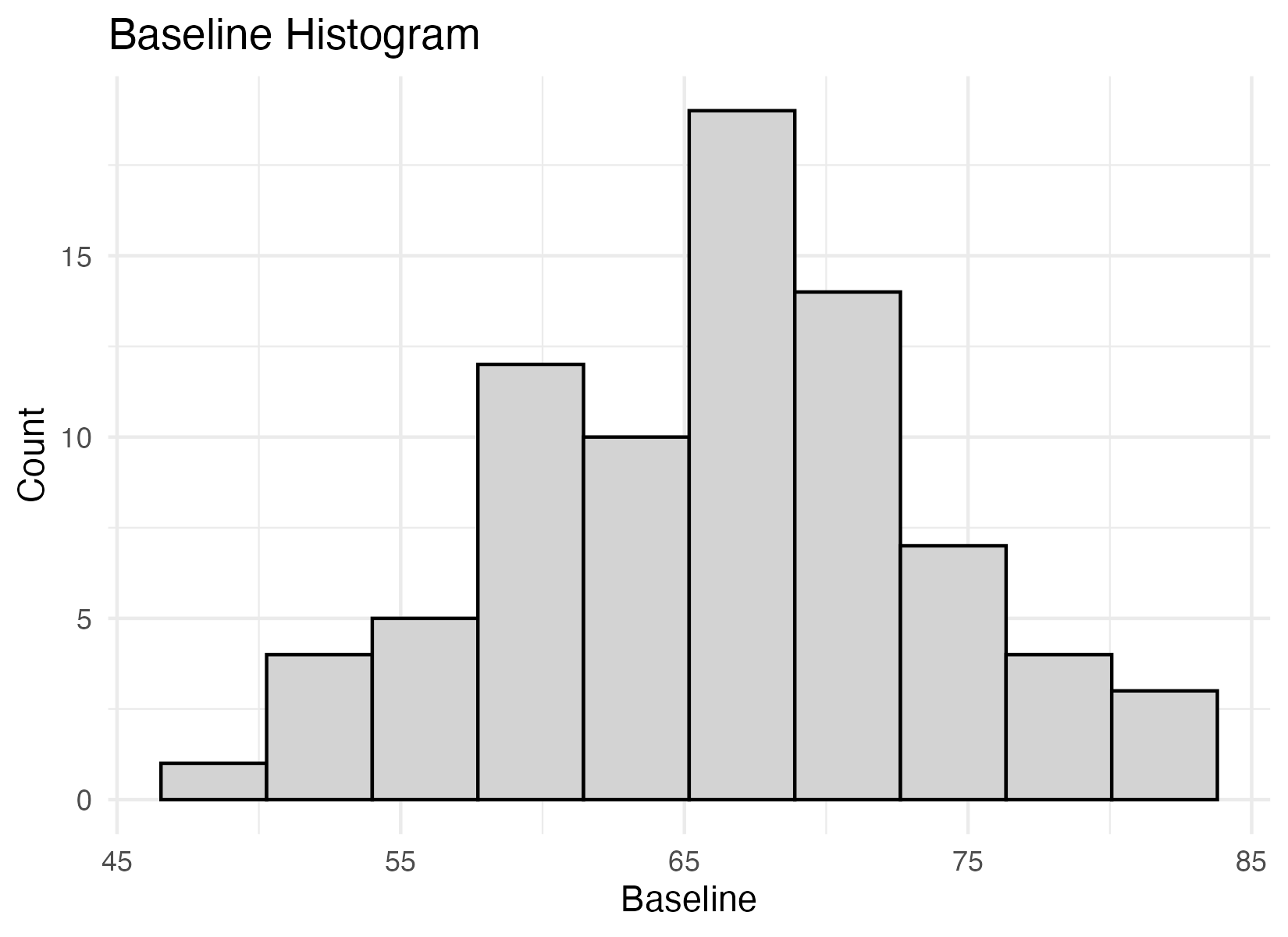


## Fitted vs Residuals


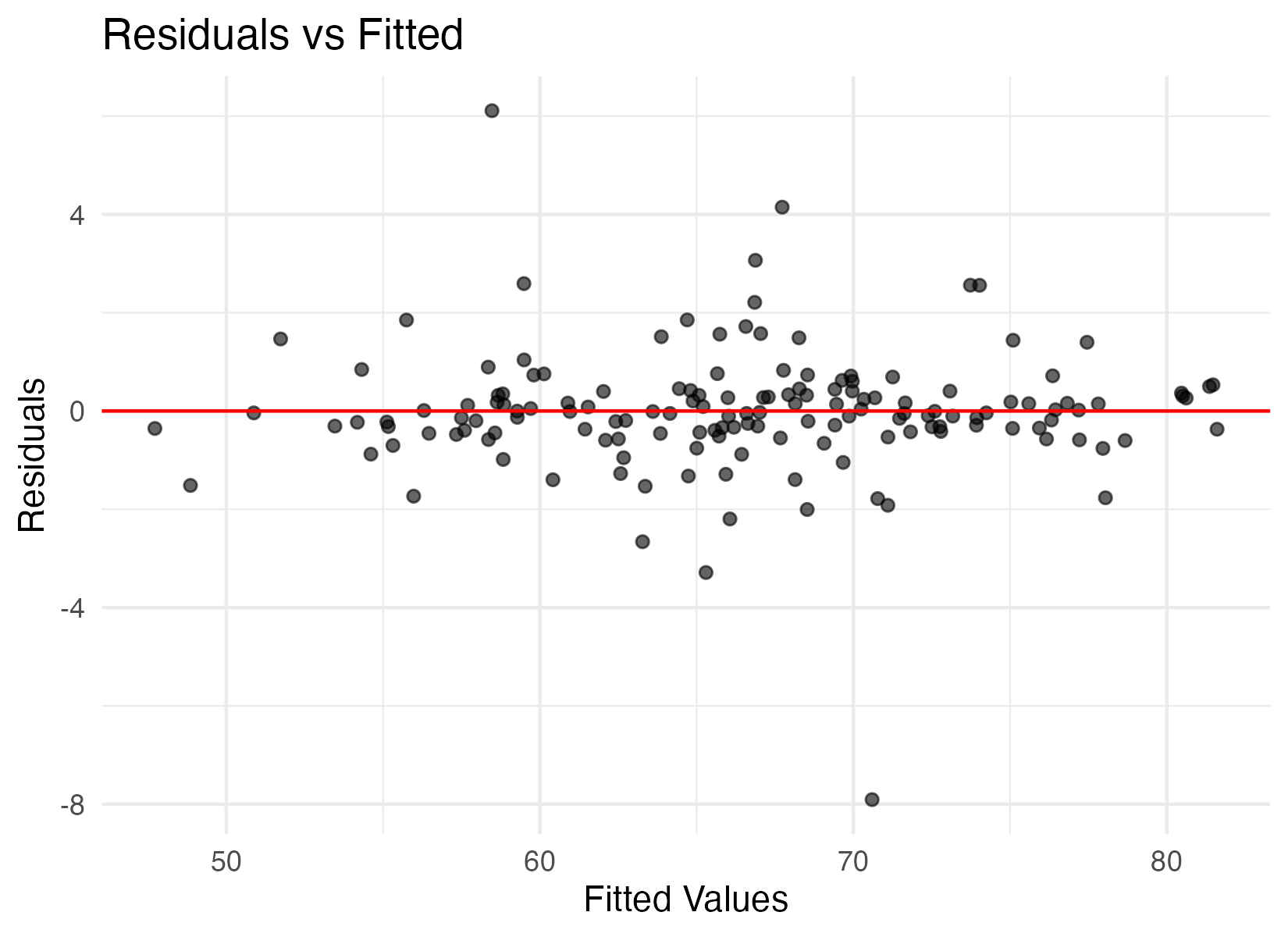


## QQ Plot


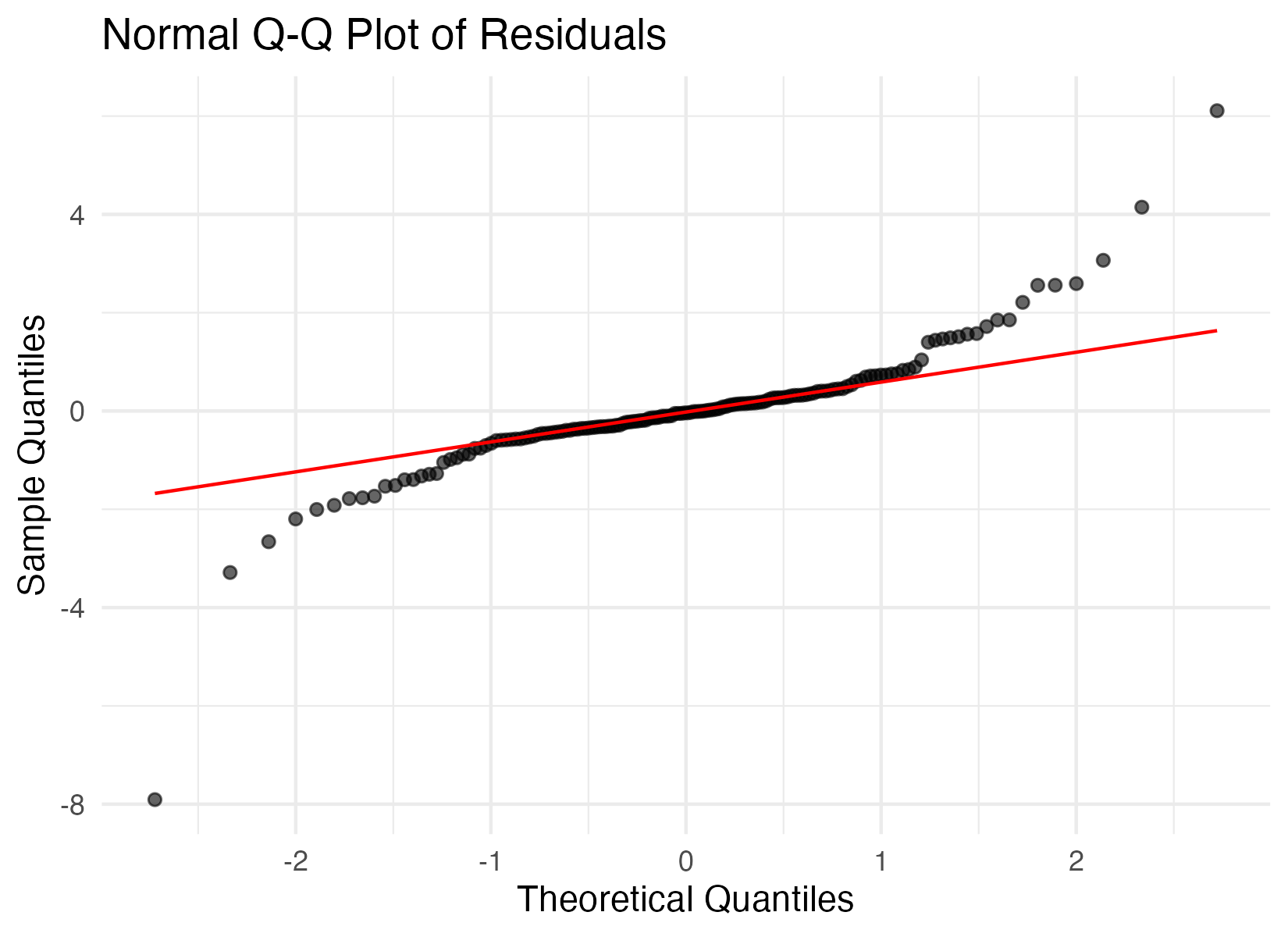


## Within-group change (baseline to follow-up)

| contrast | group | estimate | SE | df | lower.CL | upper.CL | t.ratio | p.value | effect_size |
| --- | --- | --- | --- | --- | --- | --- | --- | --- | --- |
| followup - baseline | C | 0.855 | 0.289 | 143 | 0.283 | 1.427 | 2.953 | 0.004 | 0.115 |
| followup - baseline | S | 1.135 | 0.297 | 143 | 0.548 | 1.722 | 3.825 | <0.001 | 0.153 |

## Between-group difference in change (interaction)

| timepoint_revpairwise | group_revpairwise | estimate | SE | df | lower.CL | upper.CL | t.ratio | p.value | effect_size |
| --- | --- | --- | --- | --- | --- | --- | --- | --- | --- |
| followup - baseline | S - C | 0.28 | 0.414 | 143 | -0.539 | 1.1 | 0.676 | 0.5 | 0.038 |

## Adjusted Means Over Time (with 95% CI)


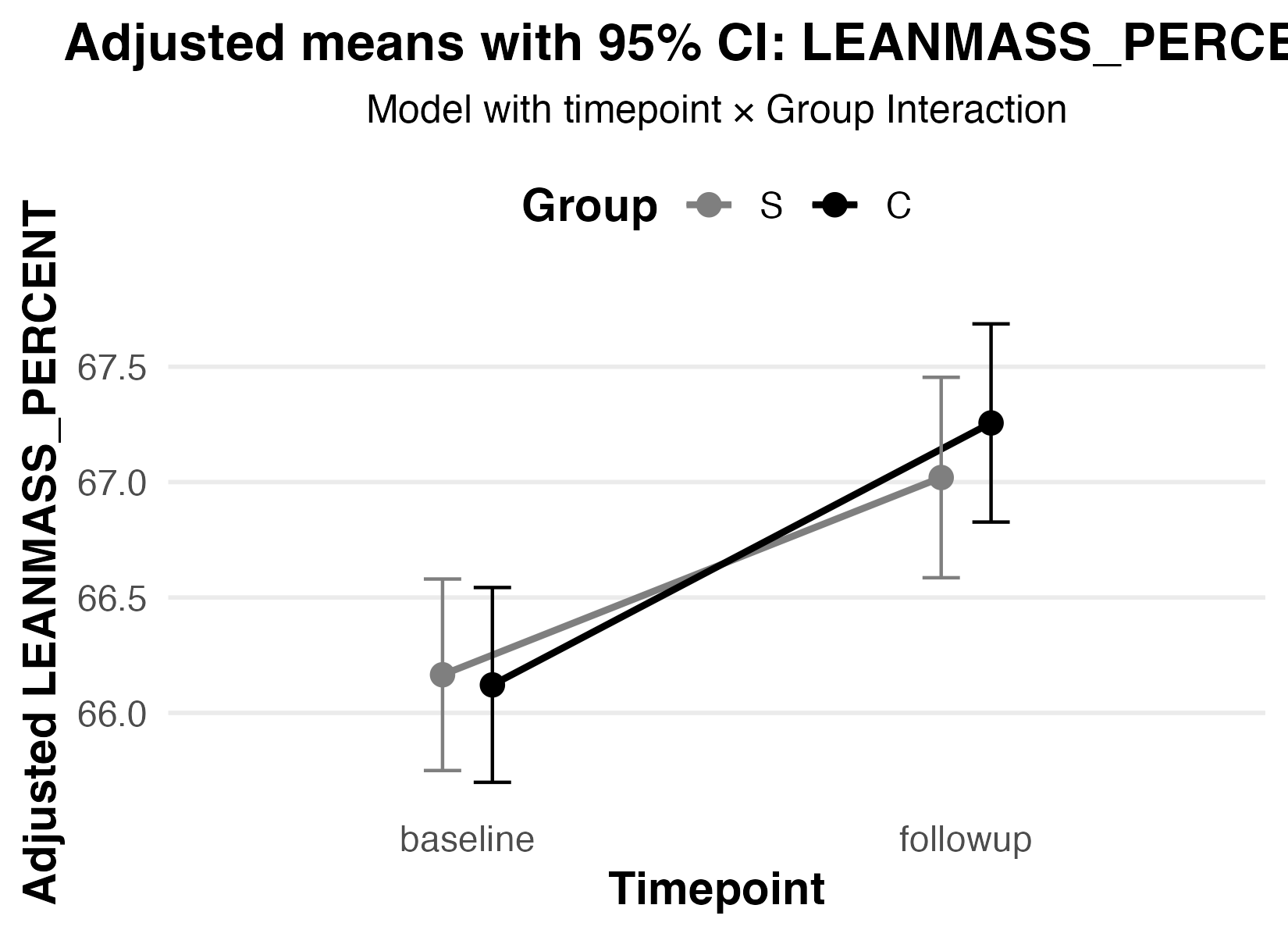


# Outcome: vo2peak_ml_kg_min

## Number of Participants Included: 79

## Distribution of DV at Baseline


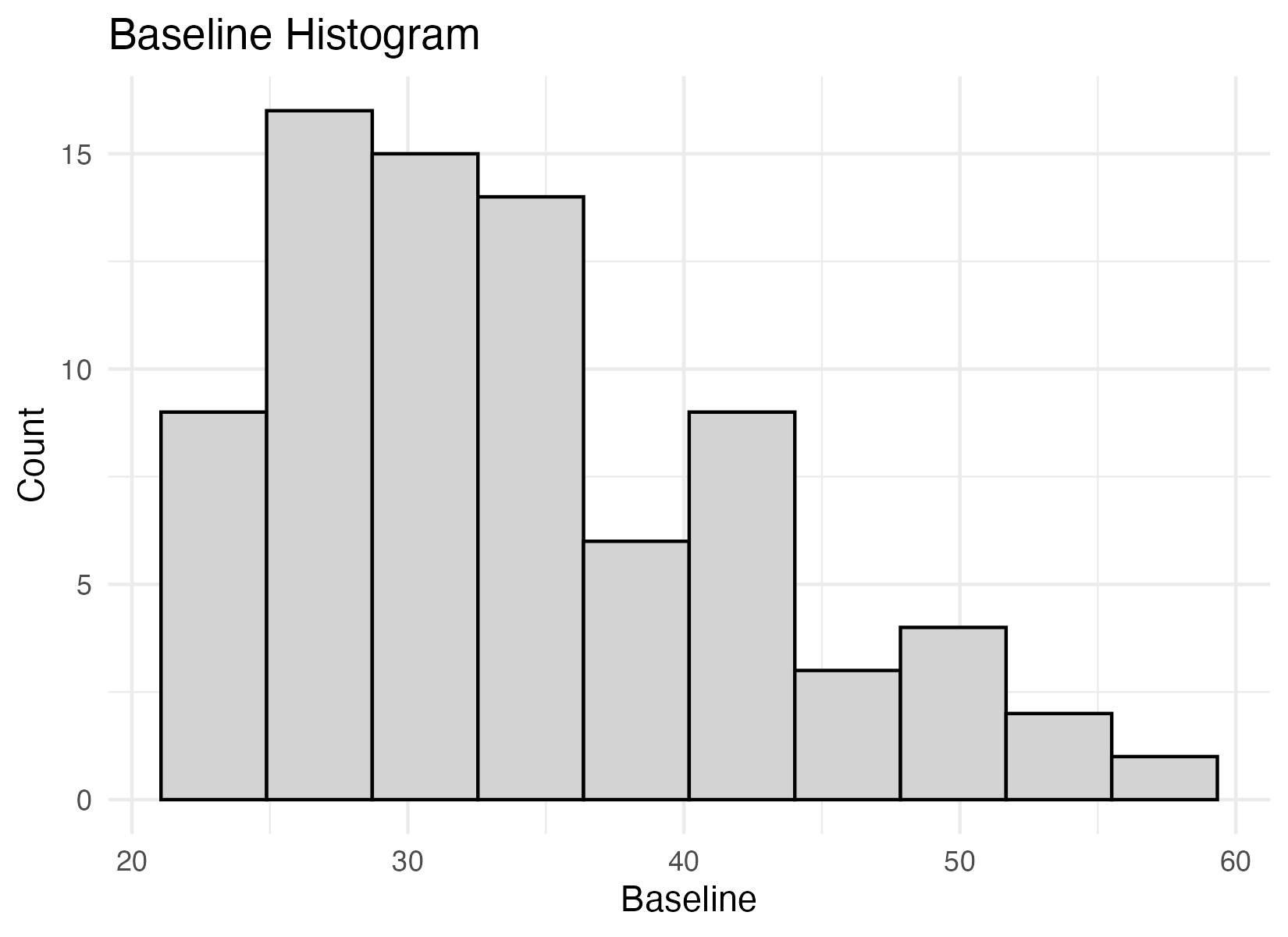


## Fitted vs Residuals


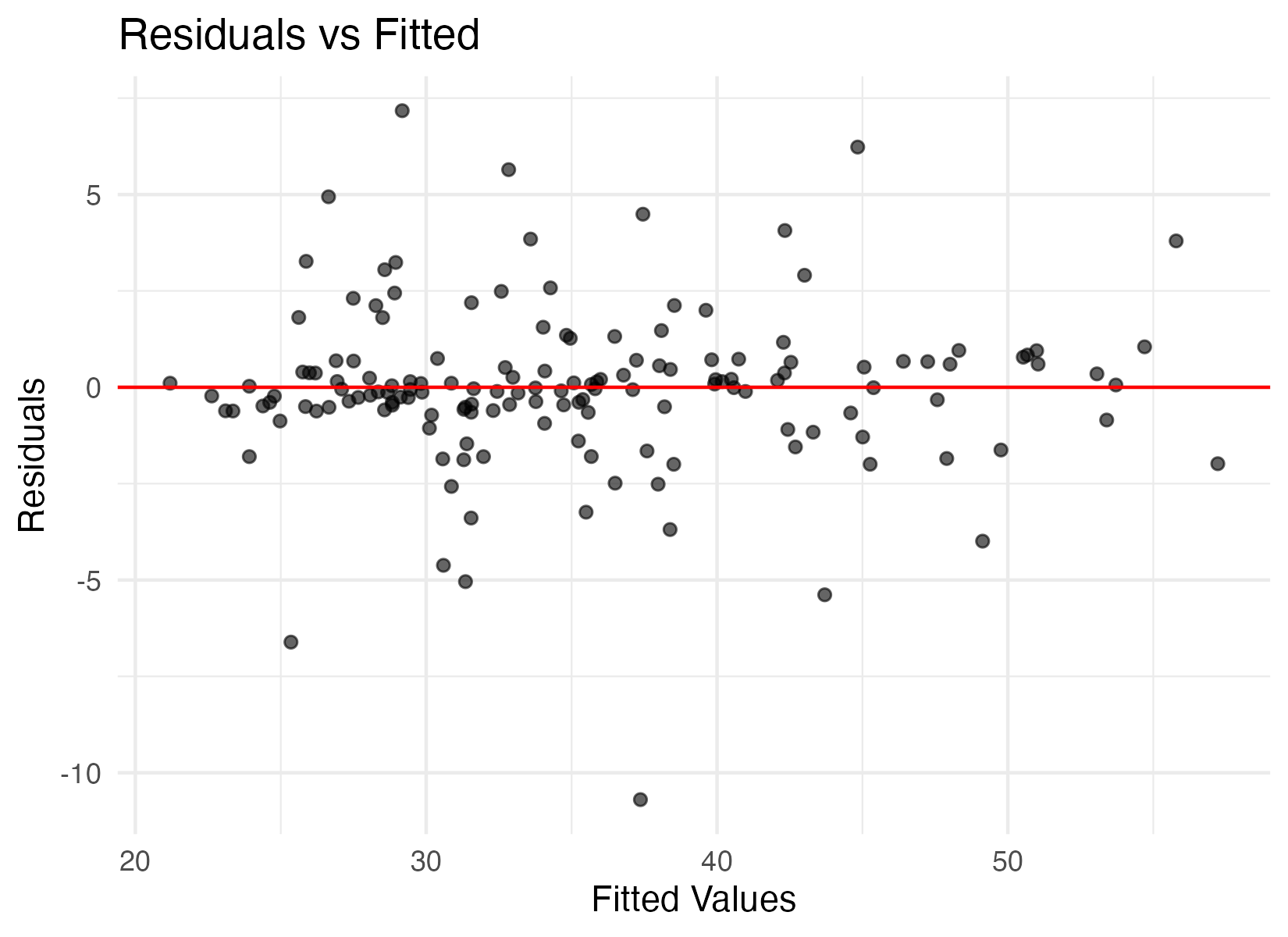


## QQ Plot


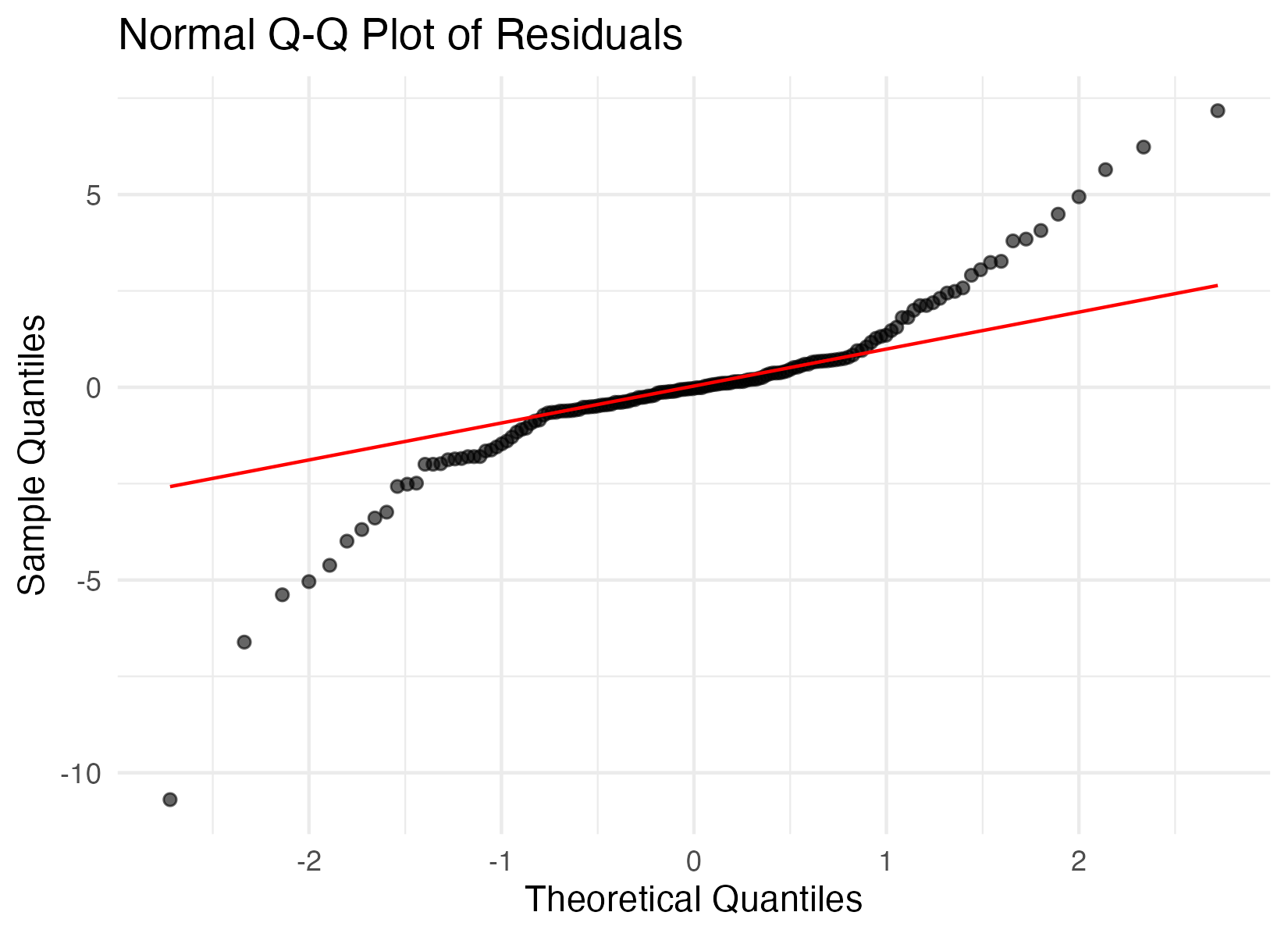


## Within-group change (baseline to follow-up)

| contrast | group | estimate | SE | df | lower.CL | upper.CL | t.ratio | p.value | effect_size |
| --- | --- | --- | --- | --- | --- | --- | --- | --- | --- |
| followup - baseline | C | 2.517 | 0.476 | 143 | 1.577 | 3.457 | 5.292 | <0.001 | 0.303 |
| followup - baseline | S | 2.724 | 0.488 | 143 | 1.760 | 3.689 | 5.586 | <0.001 | 0.328 |

## Between-group difference in change (interaction)

| timepoint_revpairwise | group_revpairwise | estimate | SE | df | lower.CL | upper.CL | t.ratio | p.value | effect_size |
| --- | --- | --- | --- | --- | --- | --- | --- | --- | --- |
| followup - baseline | S - C | 0.207 | 0.681 | 143 | -1.139 | 1.554 | 0.304 | 0.761 | 0.025 |

## Adjusted Means Over Time (with 95% CI)


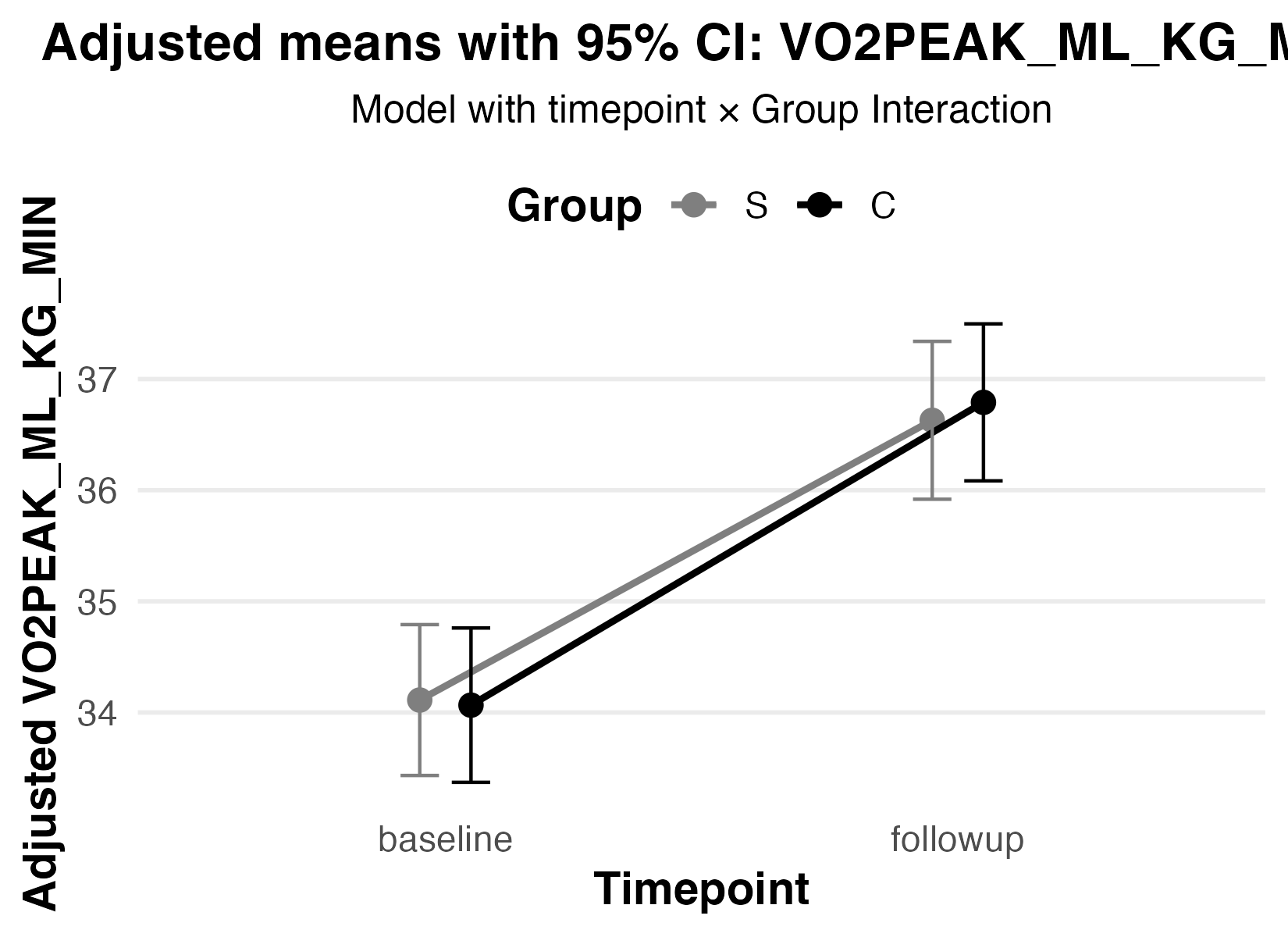


# Outcome: vo2_l_min

## Number of Participants Included: 79

## Distribution of DV at Baseline


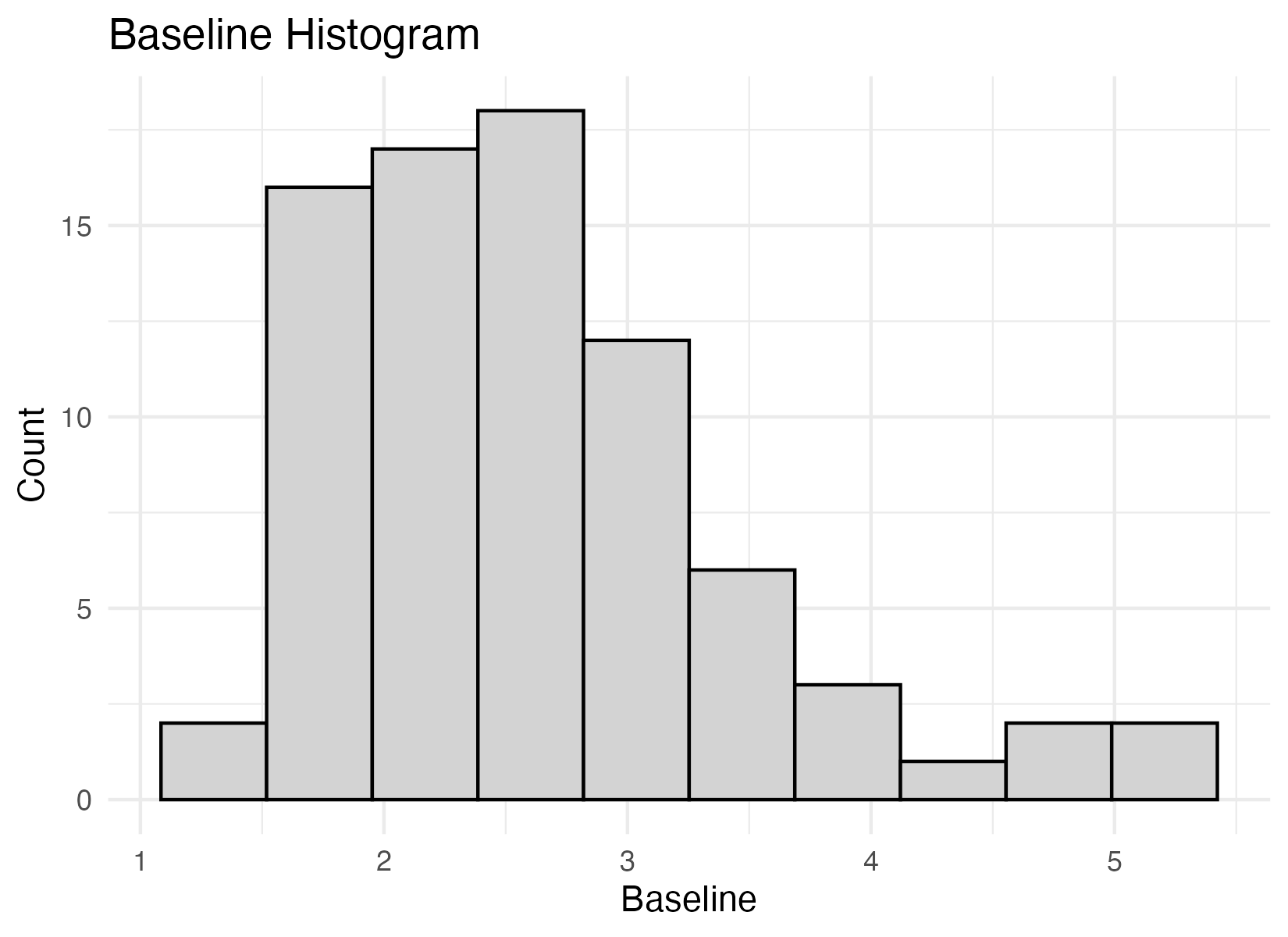


## Fitted vs Residuals


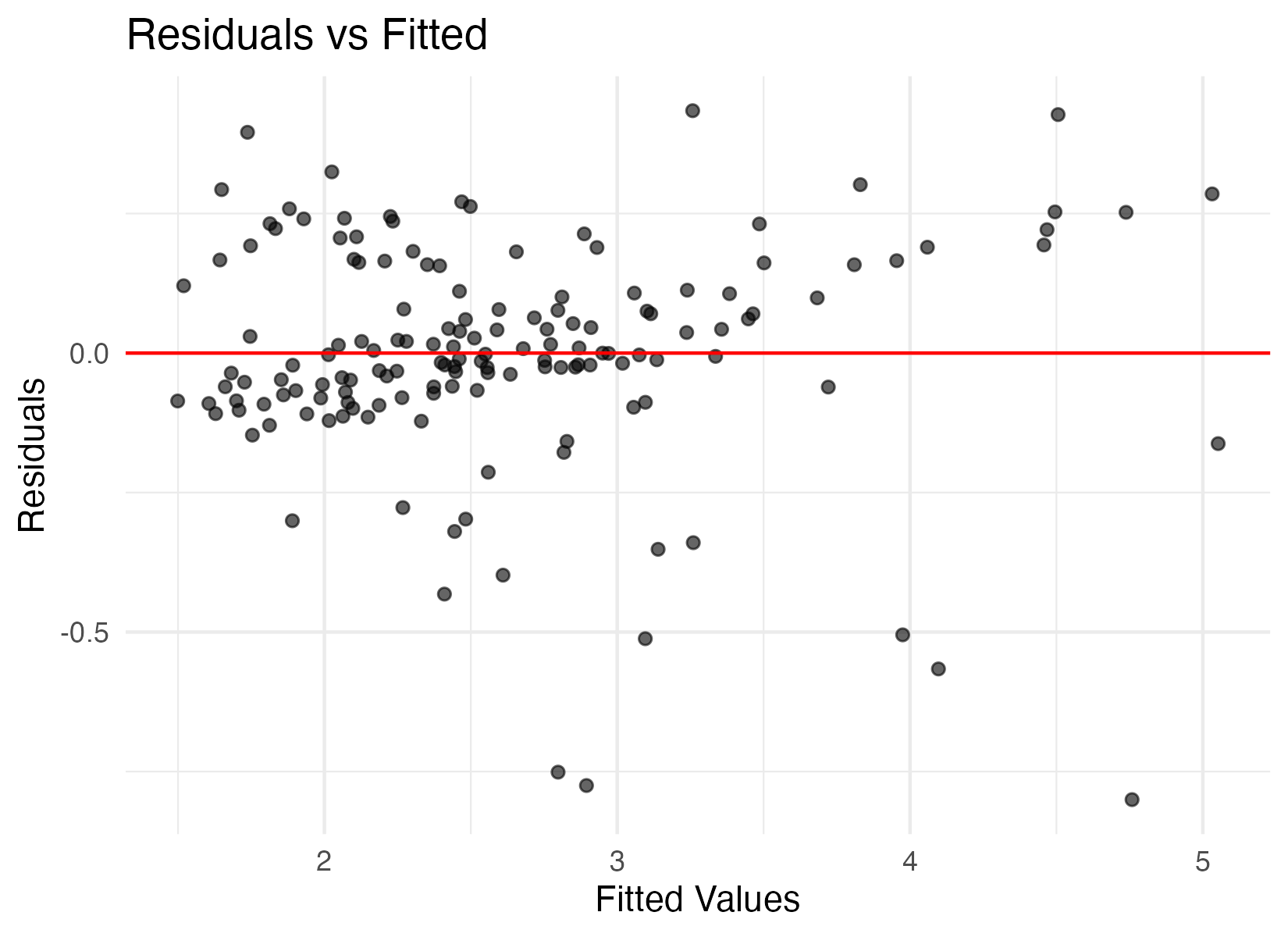


## QQ Plot


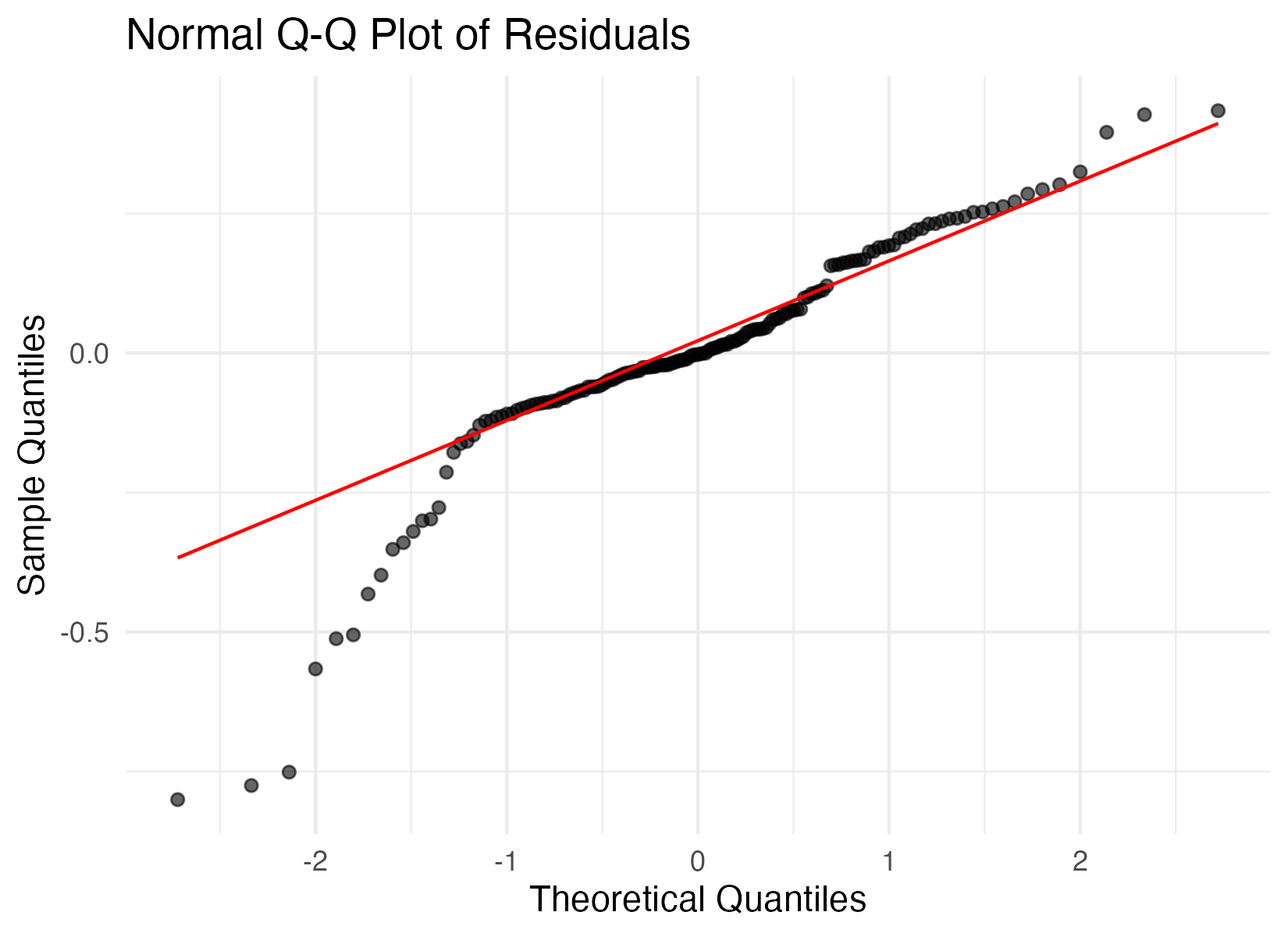


## Within-group change (baseline to follow-up)

| contrast | group | estimate | SE | df | lower.CL | upper.CL | t.ratio | p.value | effect_size |
| --- | --- | --- | --- | --- | --- | --- | --- | --- | --- |
| followup - baseline | C | 0.020 | 0.046 | 143 | -0.070 | 0.111 | 0.447 | 0.656 | 0.026 |
| followup - baseline | S | 0.038 | 0.047 | 143 | -0.055 | 0.131 | 0.803 | 0.424 | 0.048 |

## Between-group difference in change (interaction)

| timepoint_revpairwise | group_revpairwise | estimate | SE | df | lower.CL | upper.CL | t.ratio | p.value | effect_size |
| --- | --- | --- | --- | --- | --- | --- | --- | --- | --- |
| followup - baseline | S - C | 0.017 | 0.066 | 143 | -0.113 | 0.147 | 0.263 | 0.793 | 0.022 |

## Adjusted Means Over Time (with 95% CI)


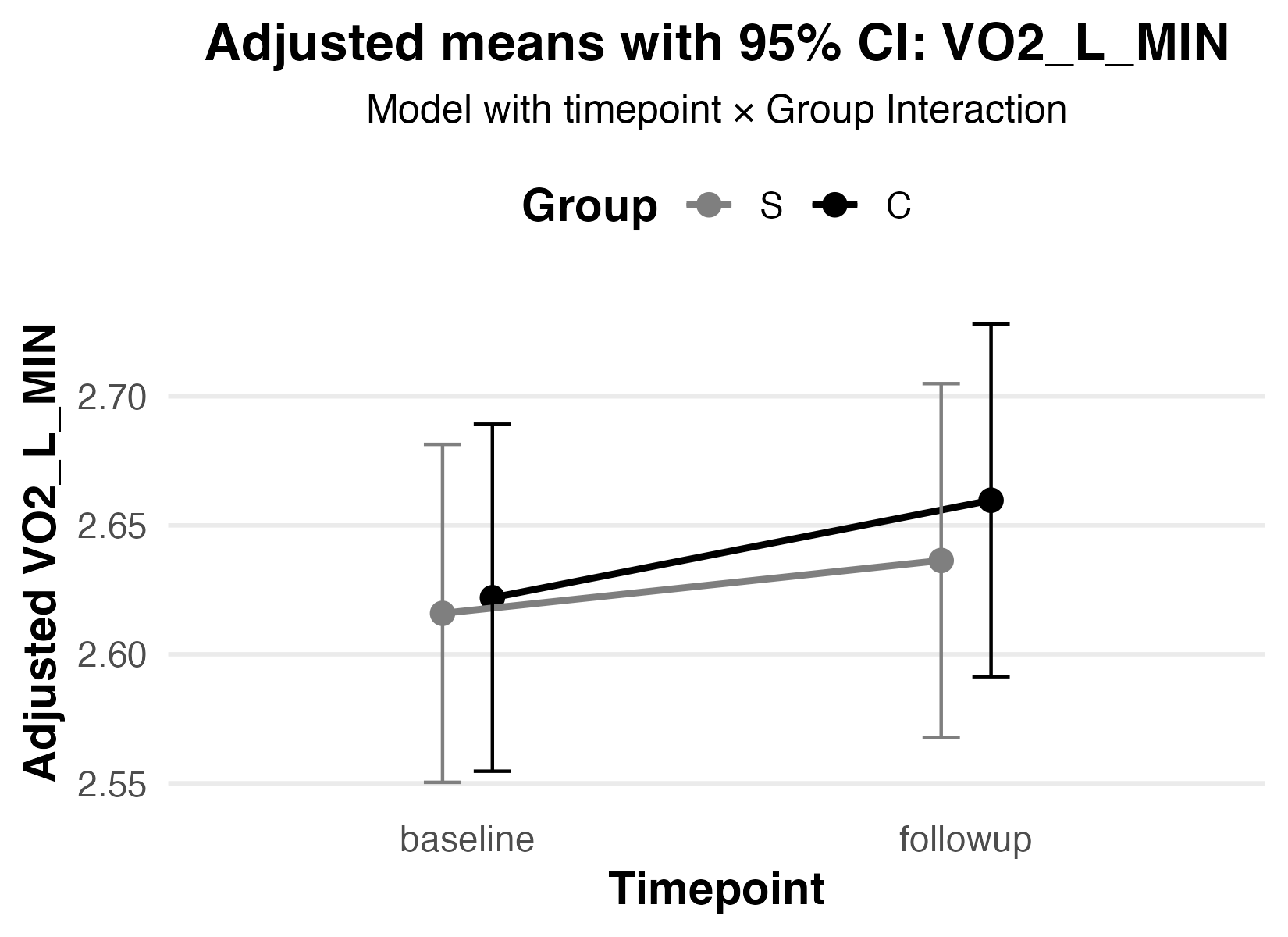


# Outcome: workmax

## Number of Participants Included: 79

## Distribution of DV at Baseline


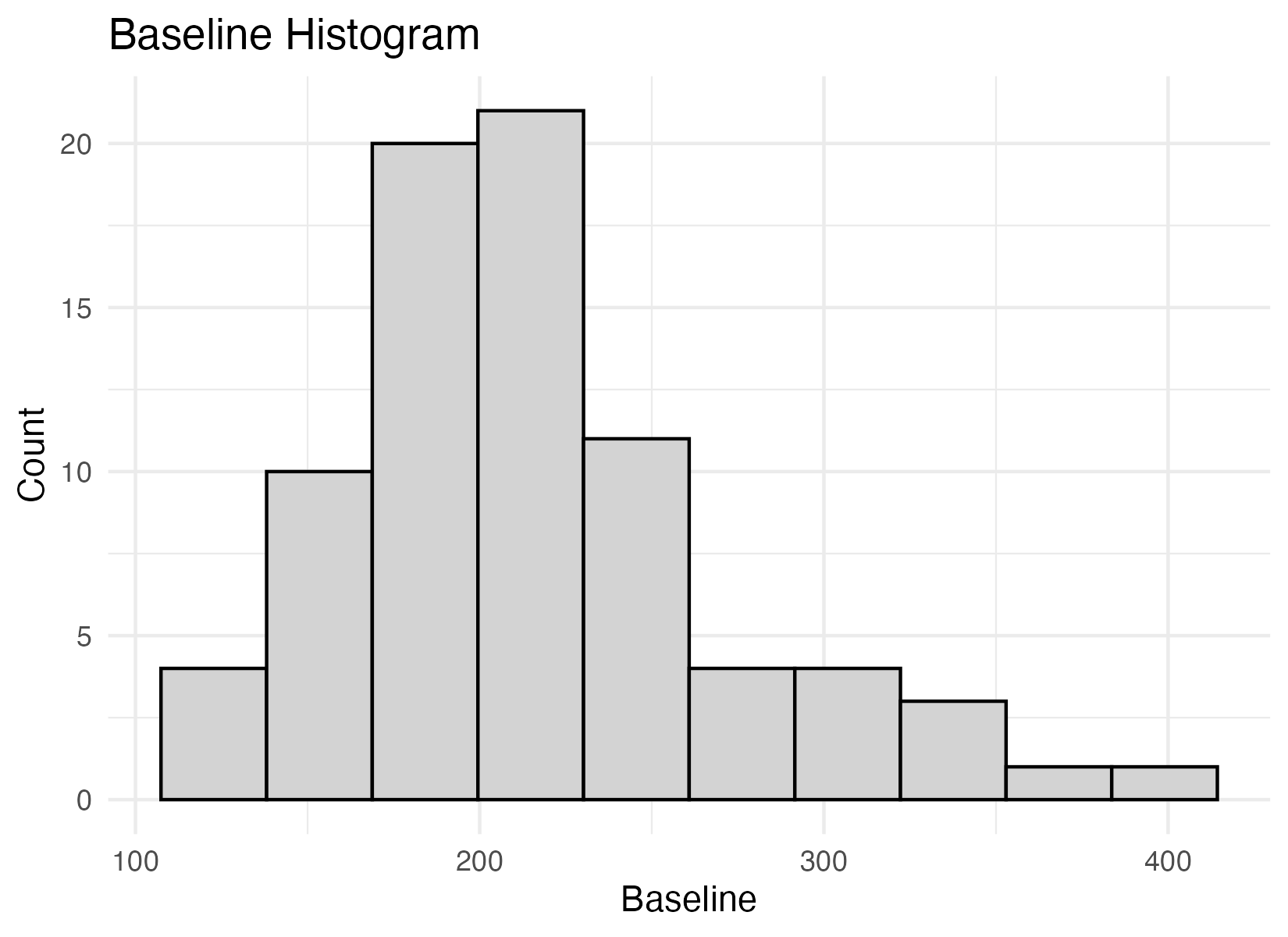


## Fitted vs Residuals


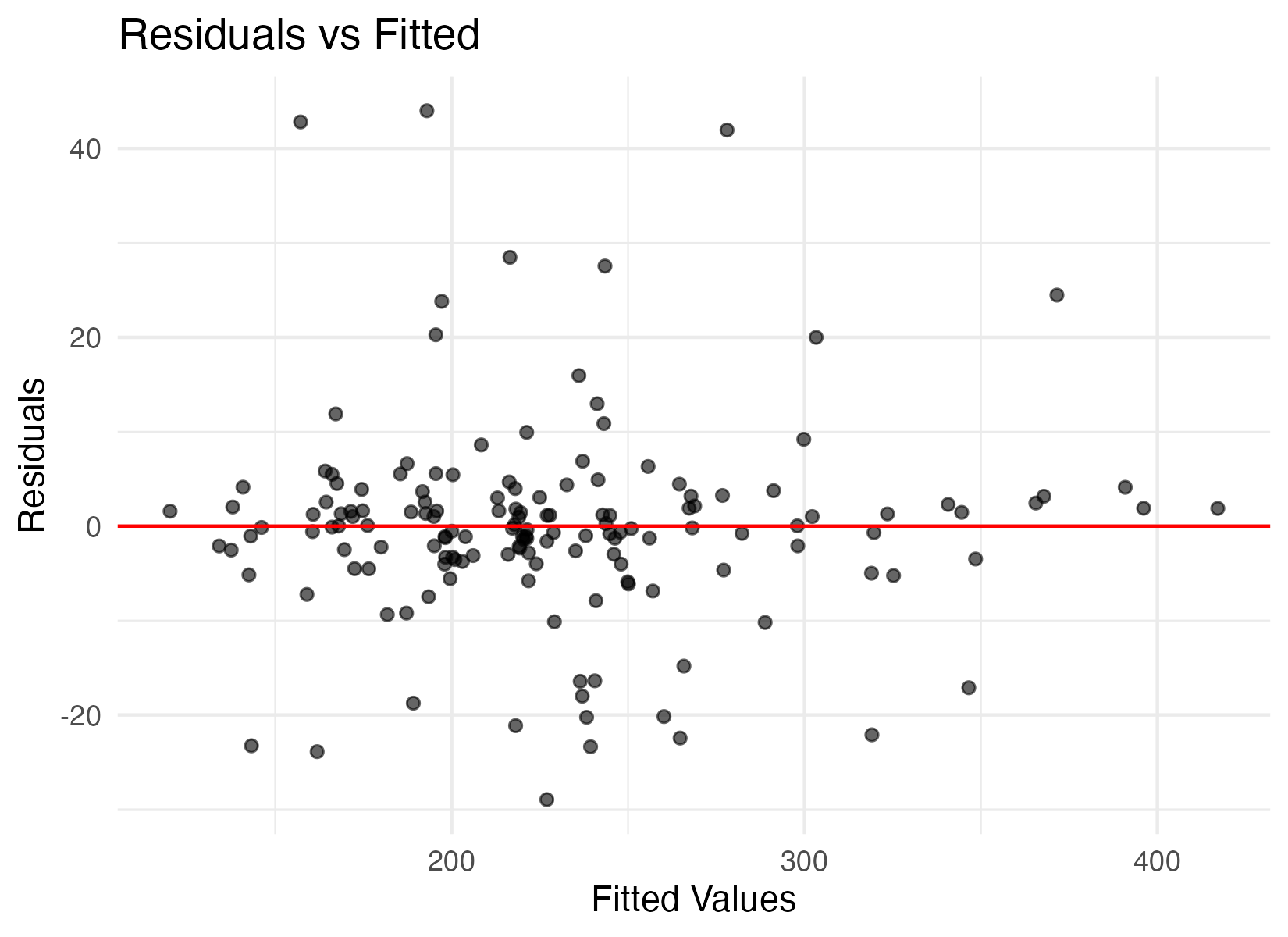


## QQ Plot


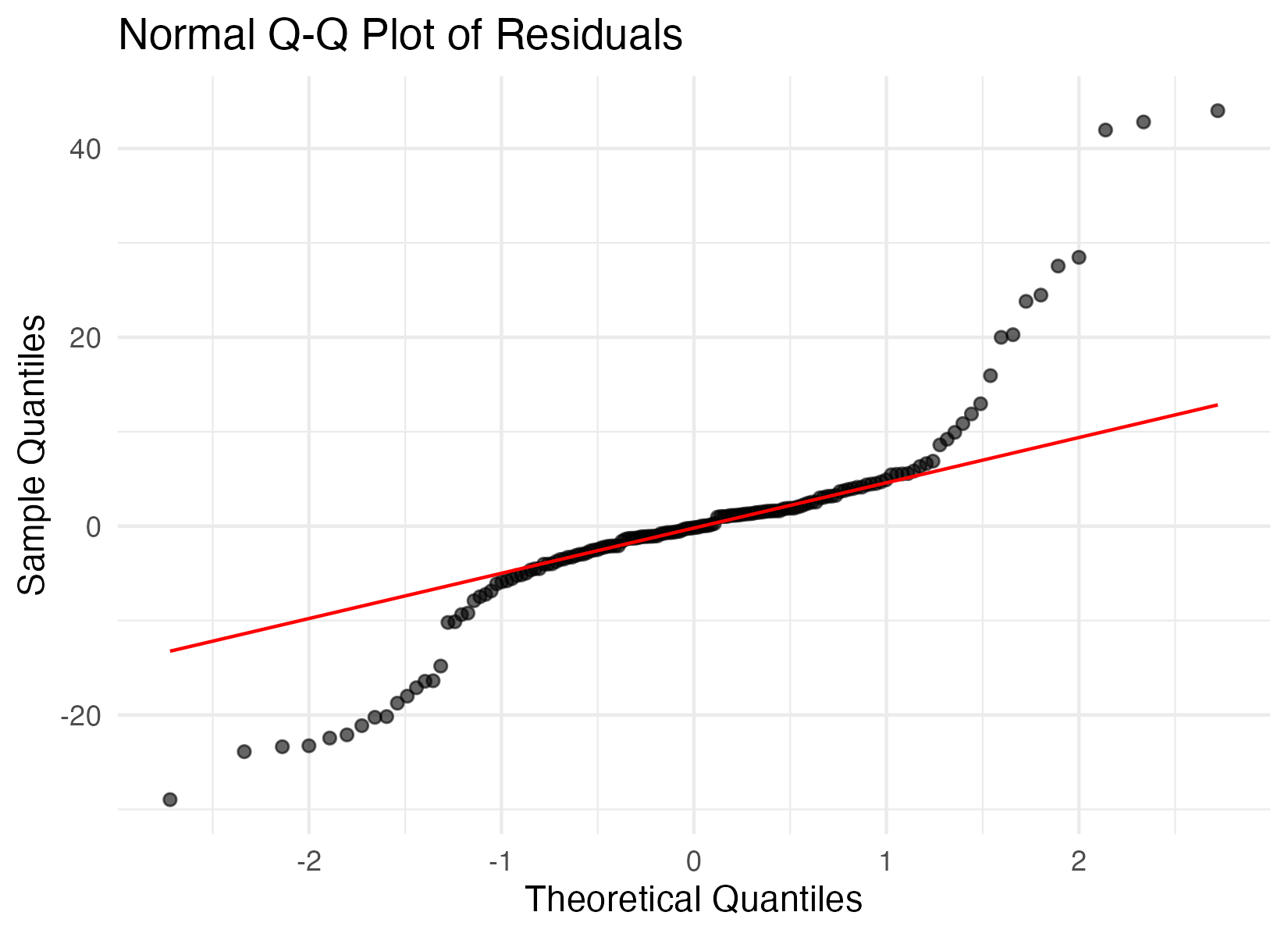


## Within-group change (baseline to follow-up)

| contrast | group | estimate | SE | df | lower.CL | upper.CL | t.ratio | p.value | effect_size |
| --- | --- | --- | --- | --- | --- | --- | --- | --- | --- |
| followup - baseline | C | 21.016 | 2.450 | 143 | 16.174 | 25.858 | 8.580 | <0.001 | 0.362 |
| followup - baseline | S | 23.049 | 2.511 | 143 | 18.085 | 28.014 | 9.178 | <0.001 | 0.397 |

## Between-group difference in change (interaction)

| timepoint_revpairwise | group_revpairwise | estimate | SE | df | lower.CL | upper.CL | t.ratio | p.value | effect_size |
| --- | --- | --- | --- | --- | --- | --- | --- | --- | --- |
| followup - baseline | S - C | 2.033 | 3.508 | 143 | -4.901 | 8.966 | 0.58 | 0.563 | 0.035 |

## Adjusted Means Over Time (with 95% CI)


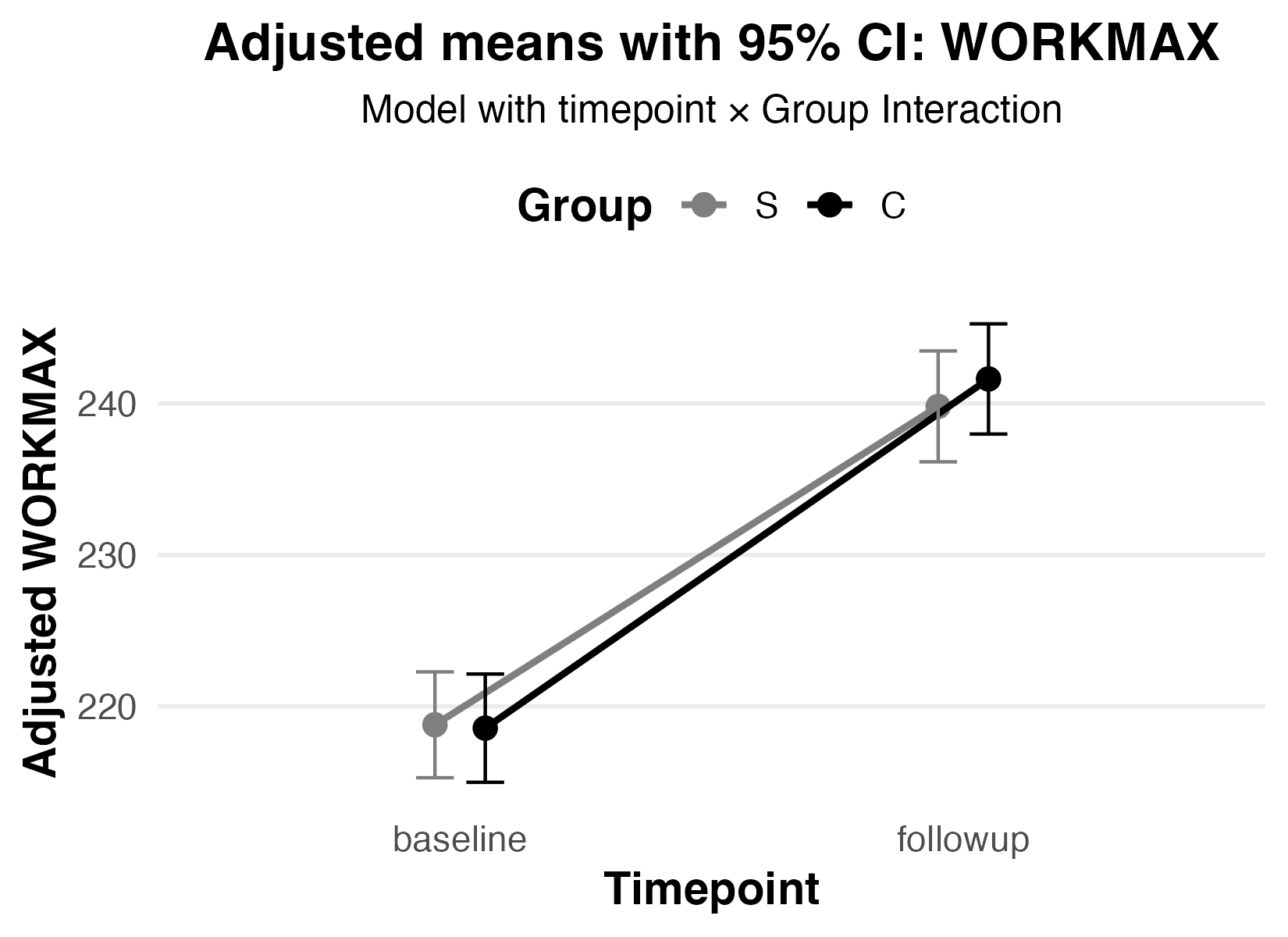


# Outcome: maxhr

## Number of Participants Included: 79

## Distribution of DV at Baseline


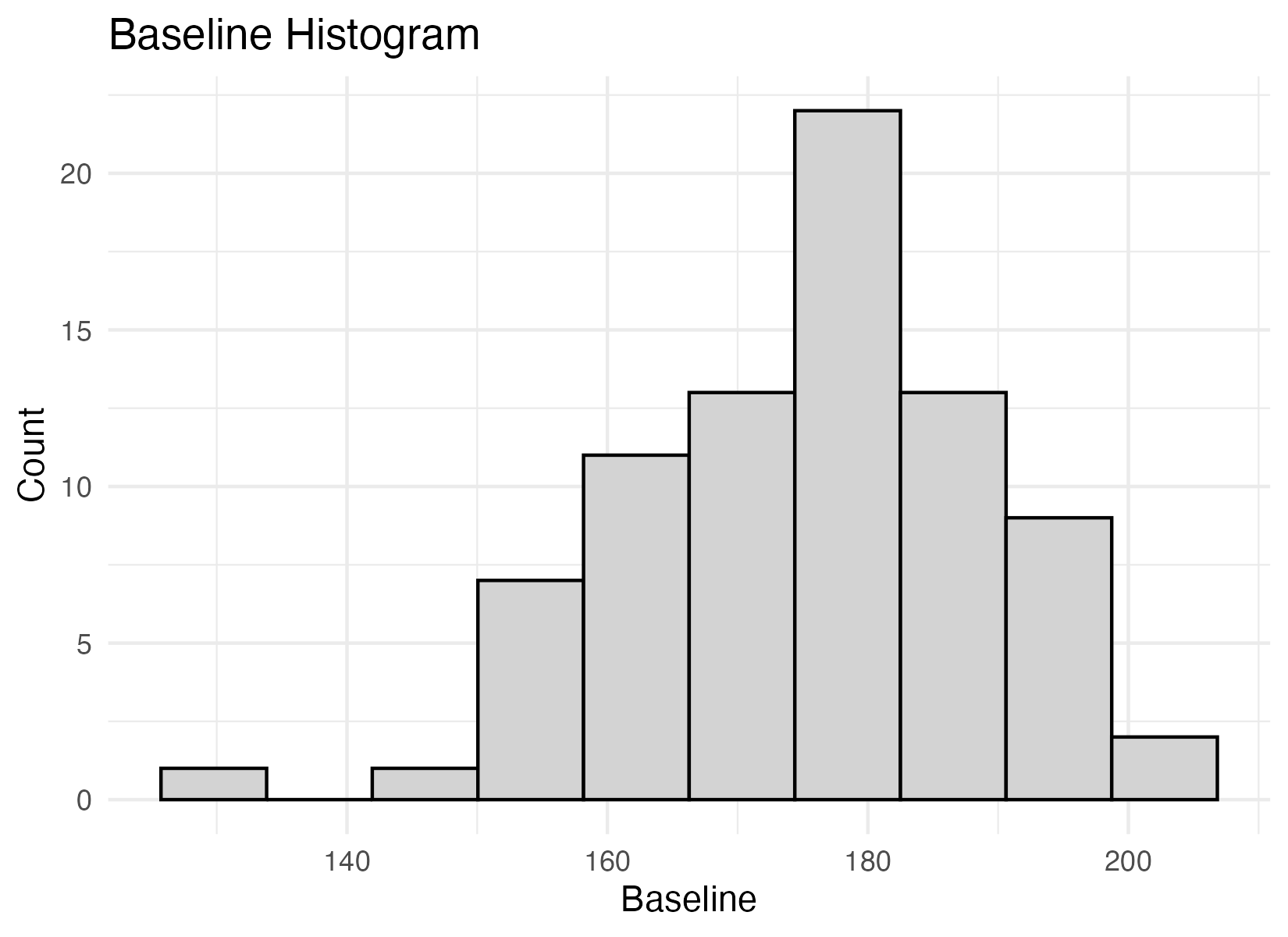


## Fitted vs Residuals


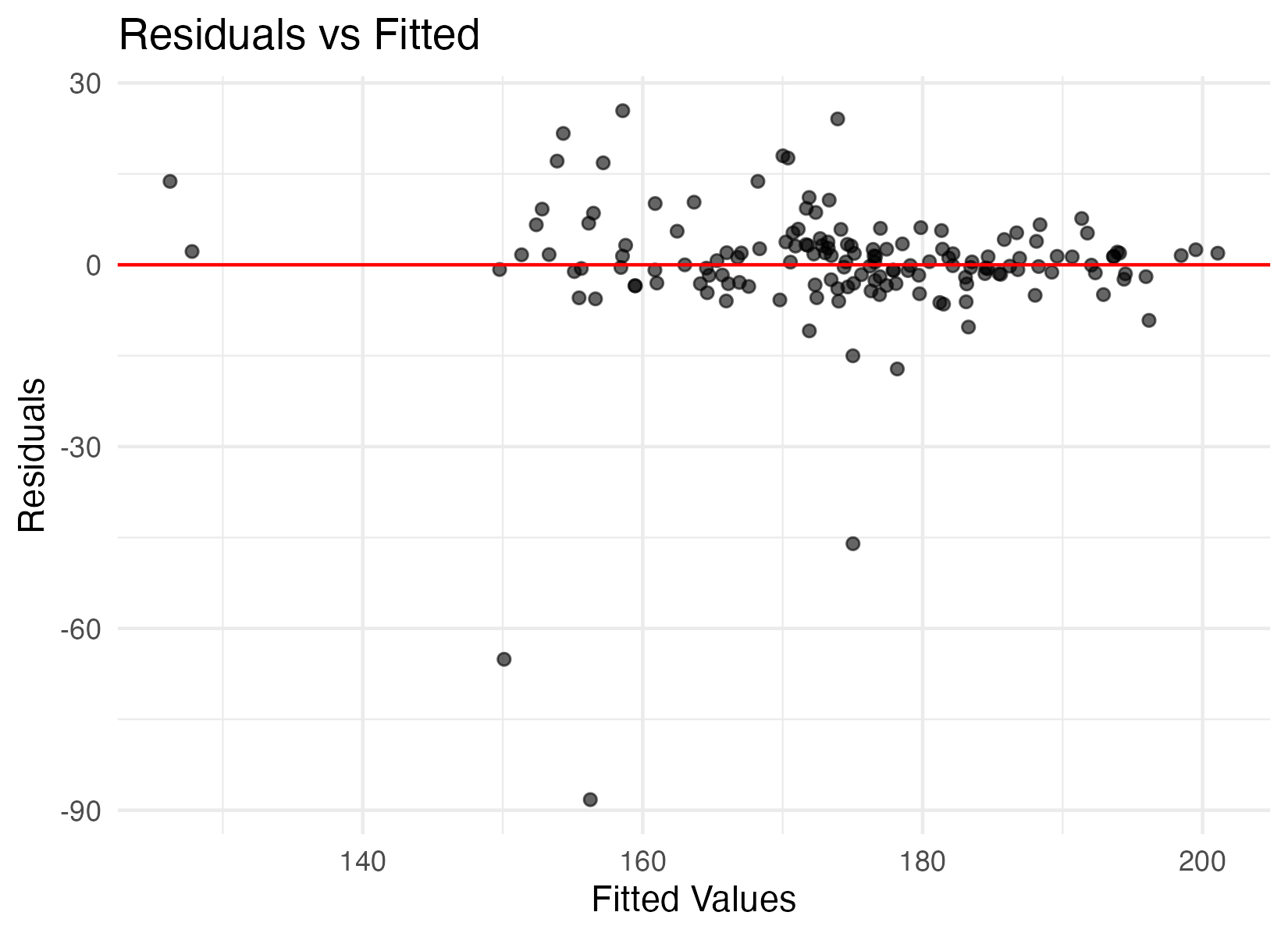


## QQ Plot


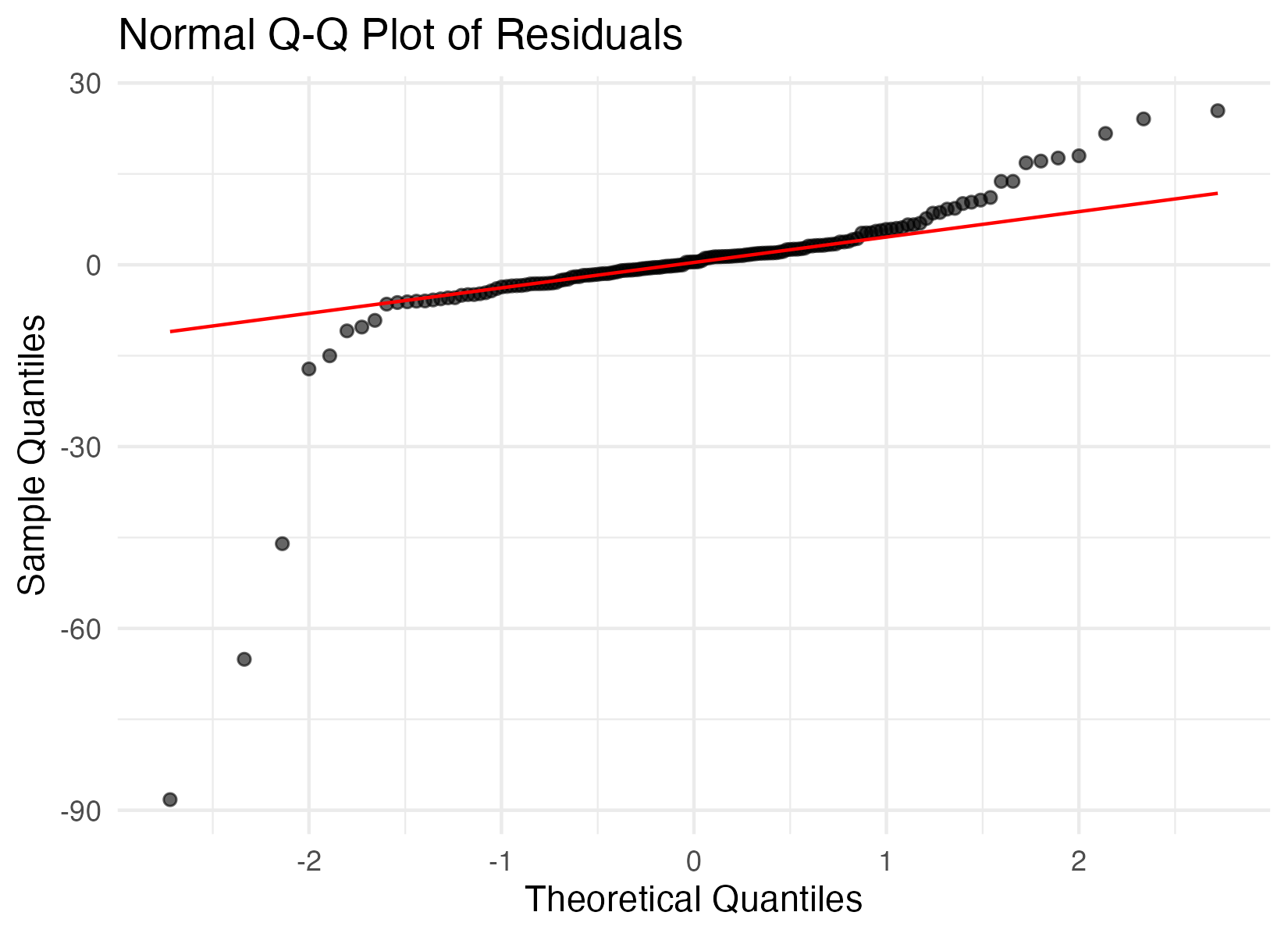


## Within-group change (baseline to follow-up)

| contrast | group | estimate | SE | df | lower.CL | upper.CL | t.ratio | p.value | effect_size |
| --- | --- | --- | --- | --- | --- | --- | --- | --- | --- |
| followup - baseline | C | -2.299 | 2.596 | 143 | -7.431 | 2.832 | -0.886 | 0.377 | -0.133 |
| followup - baseline | S | -1.570 | 2.662 | 143 | -6.832 | 3.692 | -0.590 | 0.556 | -0.091 |

## Between-group difference in change (interaction)

| timepoint_revpairwise | group_revpairwise | estimate | SE | df | lower.CL | upper.CL | t.ratio | p.value | effect_size |
| --- | --- | --- | --- | --- | --- | --- | --- | --- | --- |
| followup - baseline | S - C | 0.73 | 3.718 | 143 | -6.619 | 8.078 | 0.196 | 0.845 | 0.042 |

## Adjusted Means Over Time (with 95% CI)


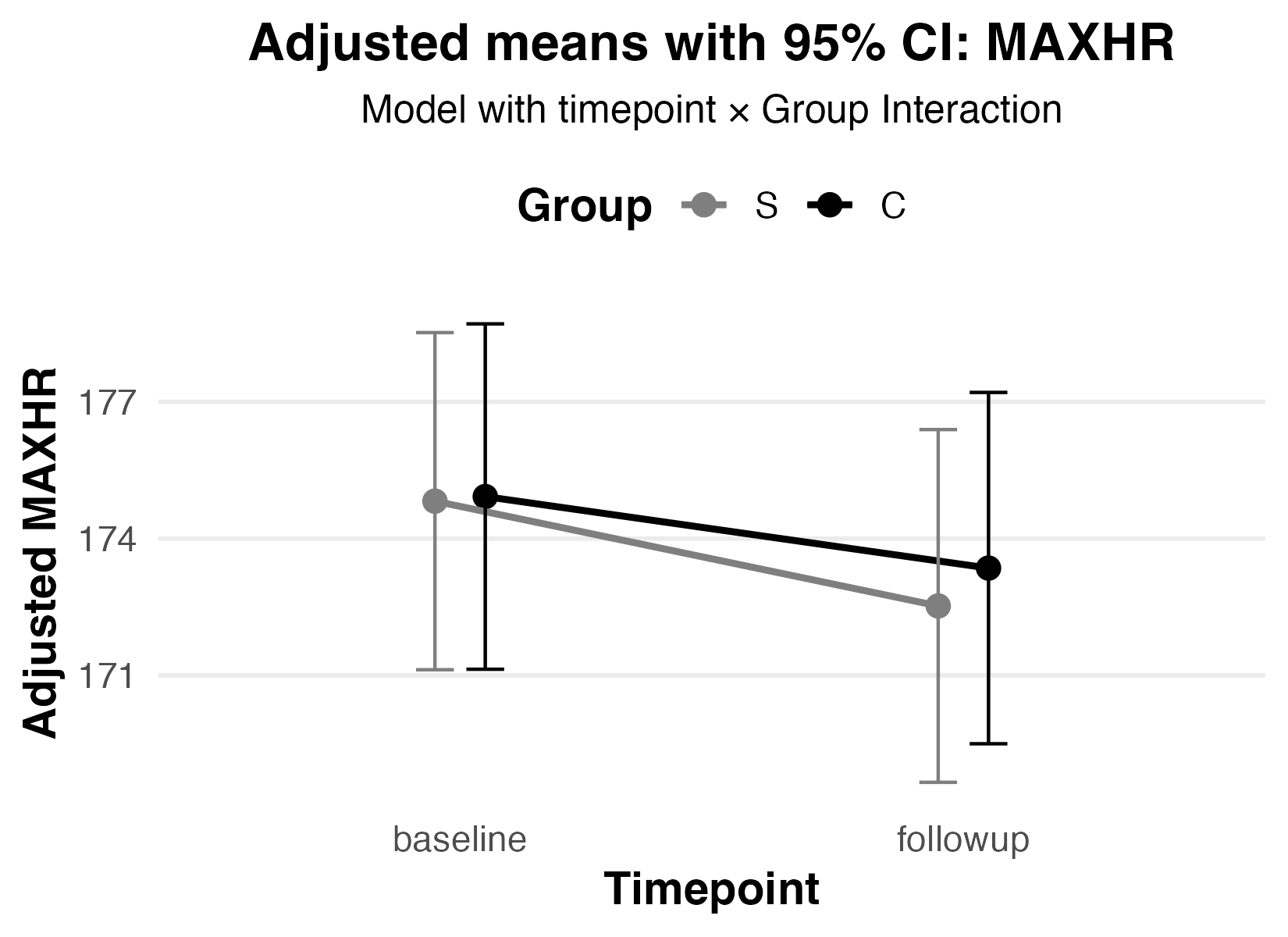

Supplement: Supplementary file 1 — Supporting Information S1 [file EJSC-26-e70199-s002.docx]
