# Supplementary material for: Physical Adaptations to High‐Intensity Multimodal Training in Recreationally Active Adults: A Randomised Control Trial
Source: Eur J Sport Sci. 2026 Jun 12;26(7):e70199. doi: 10.1002/ejsc.70199 (PMC13263161; doi:10.1002/ejsc.70199)

**Supplementary Material 2** Per-Protocol Statistical Analysis

# Outcome: x1rmsquatrelative_kg

## Number of Participants Included: 52

## Distribution of DV at Baseline


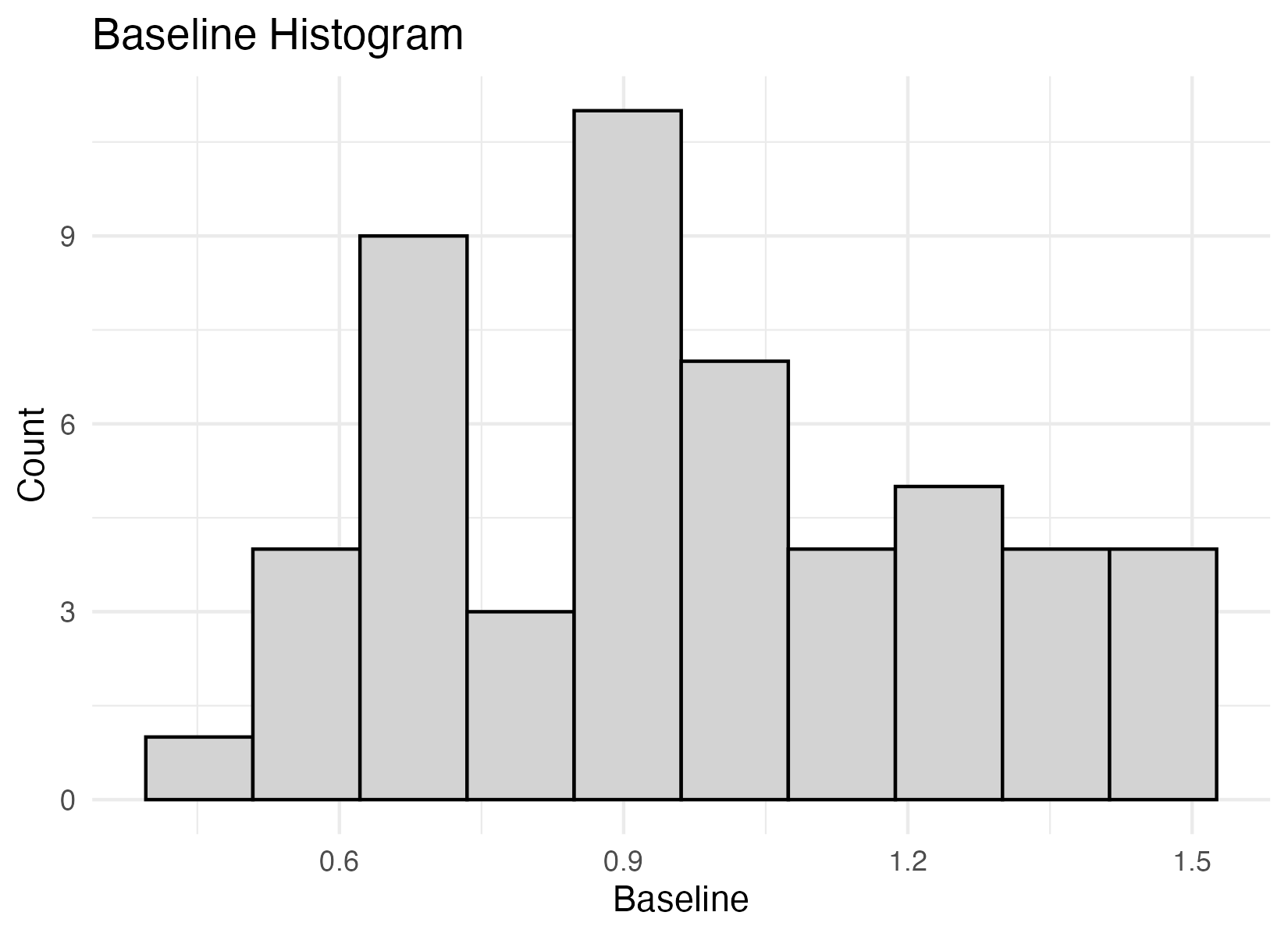


## Fitted vs Residuals


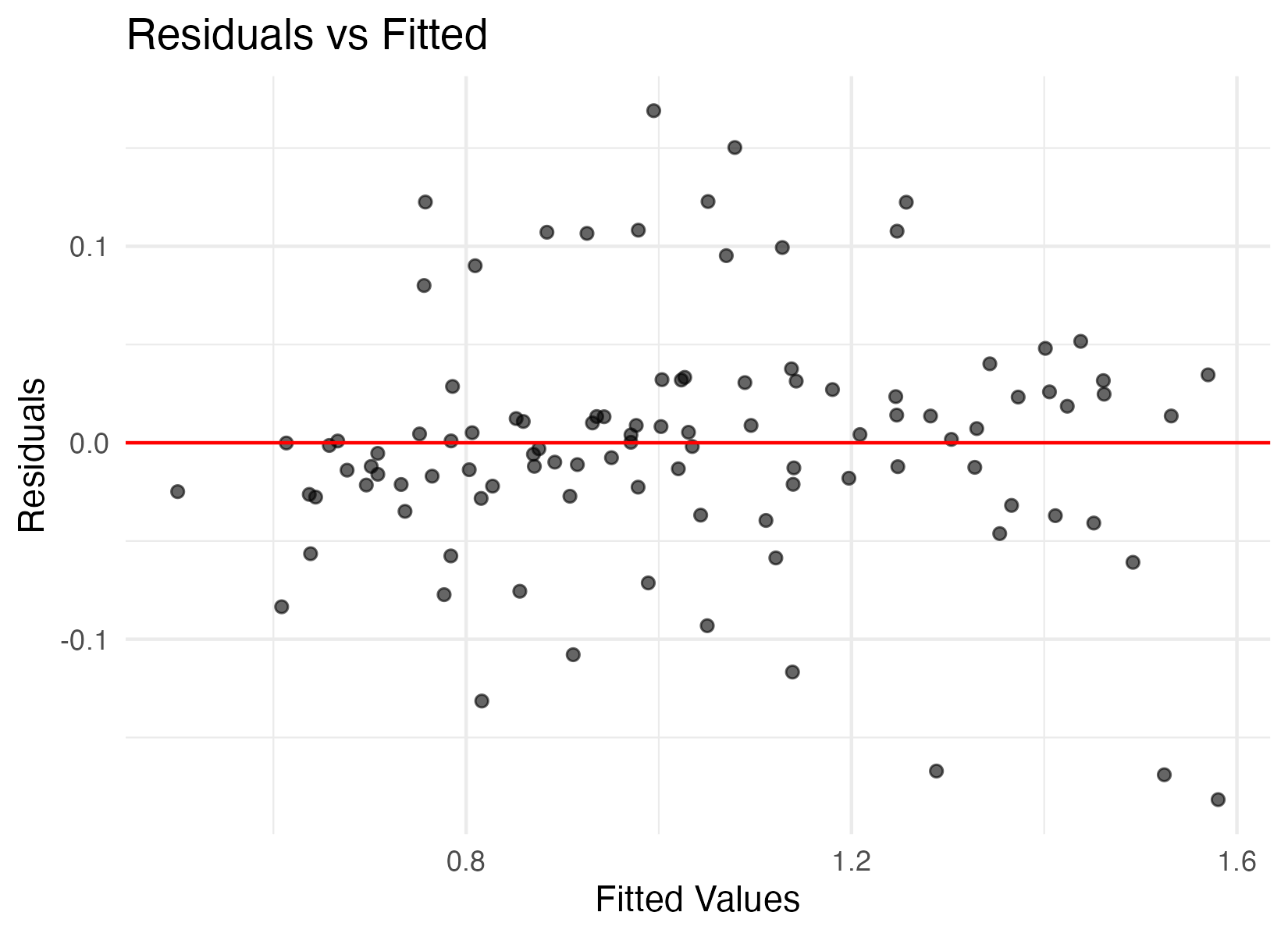


## QQ Plot


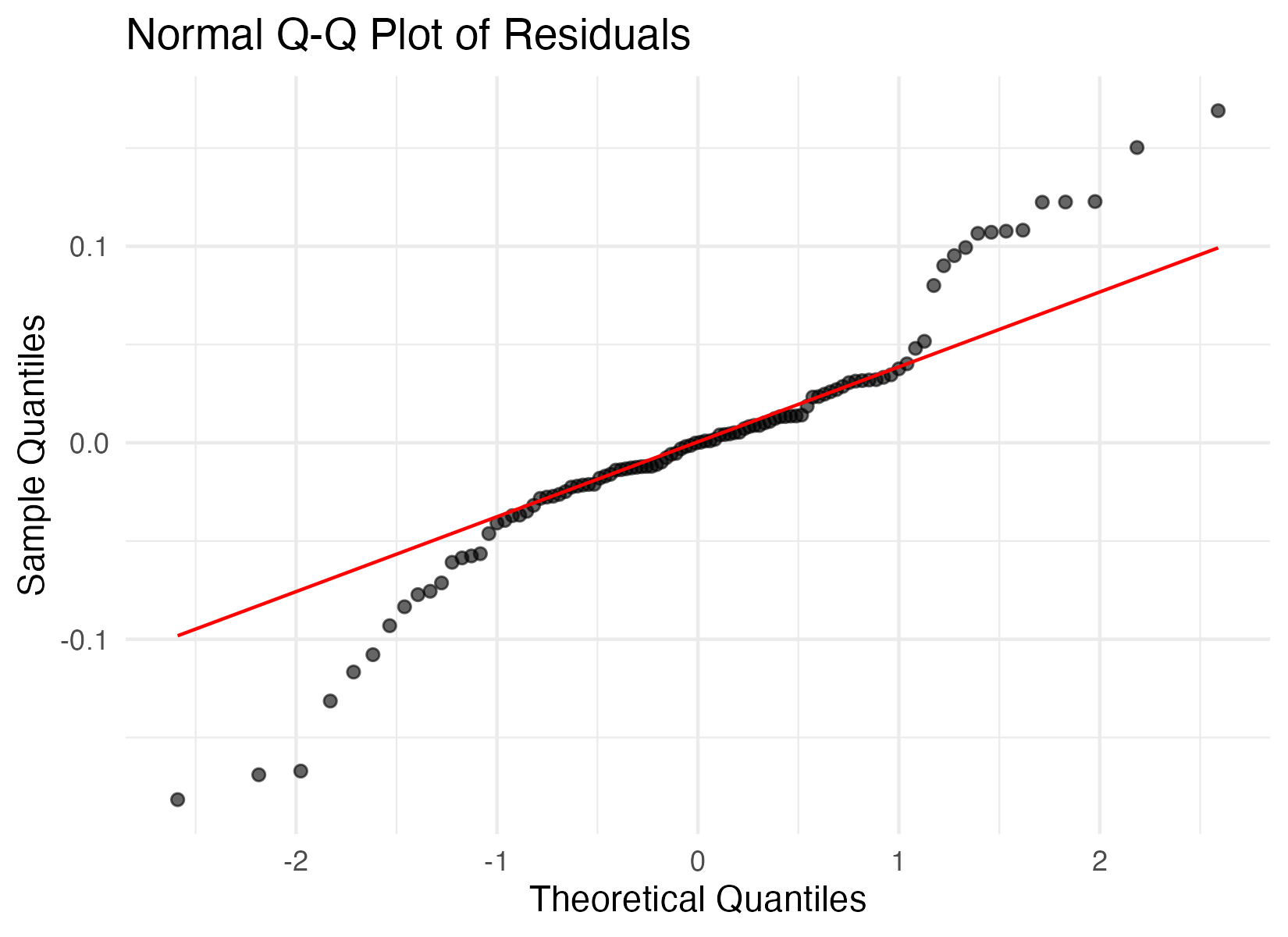


## Within-group change (baseline to follow-up)

| contrast | group | estimate | SE | df | lower.CL | upper.CL | t.ratio | p.value | effect_size |
| --- | --- | --- | --- | --- | --- | --- | --- | --- | --- |
| followup - baseline | C | 0.108 | 0.017 | 93 | 0.074 | 0.142 | 6.321 | <0.001 | 0.401 |
| followup - baseline | S | 0.119 | 0.017 | 93 | 0.085 | 0.153 | 6.980 | <0.001 | 0.443 |

## Between-group difference in change (interaction)

| timepoint_revpairwise | group_revpairwise | estimate | SE | df | lower.CL | upper.CL | t.ratio | p.value | effect_size |
| --- | --- | --- | --- | --- | --- | --- | --- | --- | --- |
| followup - baseline | S - C | 0.011 | 0.024 | 93 | -0.037 | 0.059 | 0.466 | 0.642 | 0.042 |

## Adjusted Means Over Time (with 95% CI)


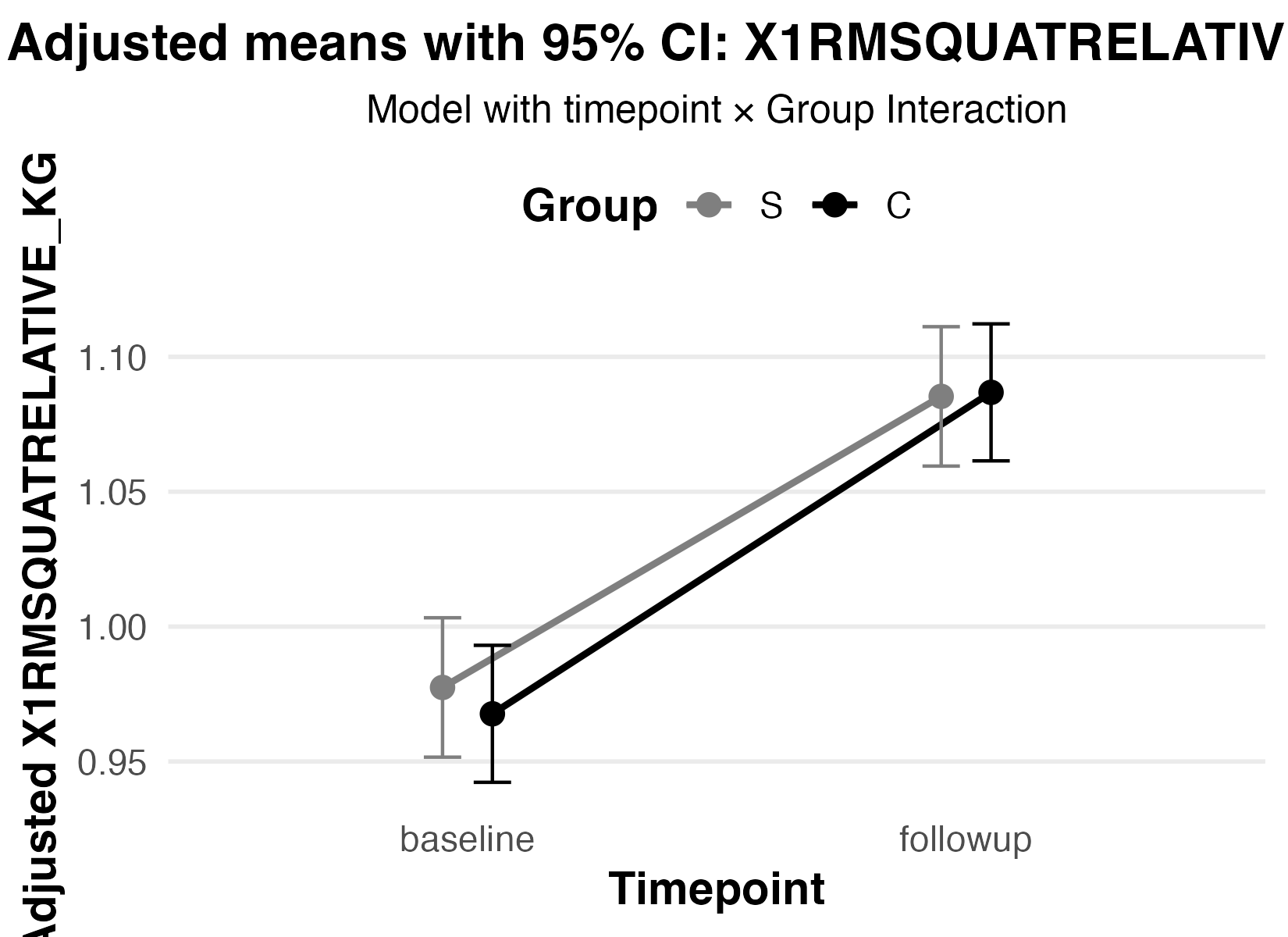


# Outcome: x1rmbench_relative_kg

## Number of Participants Included: 63

## Distribution of DV at Baseline


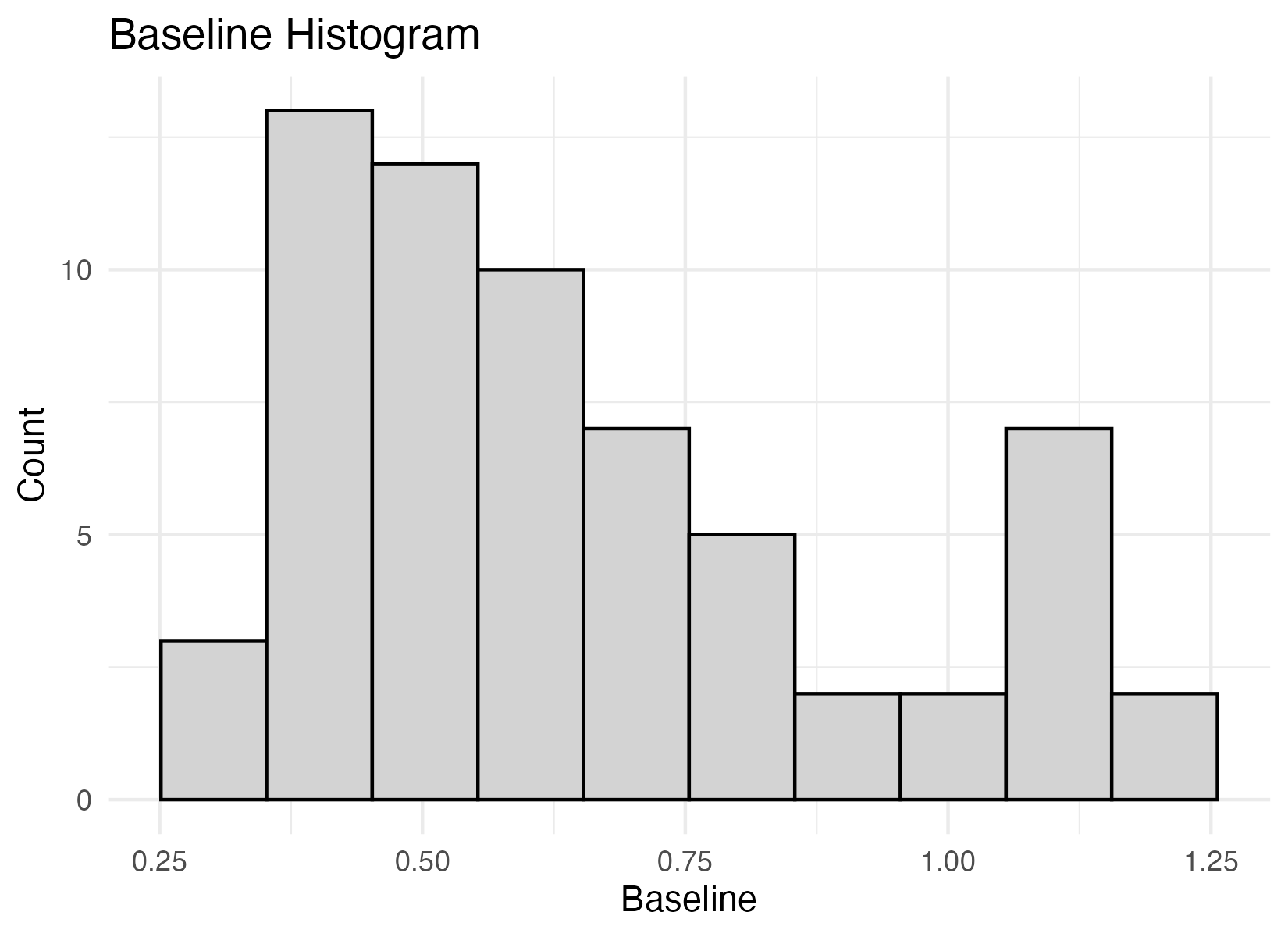


## Fitted vs Residuals


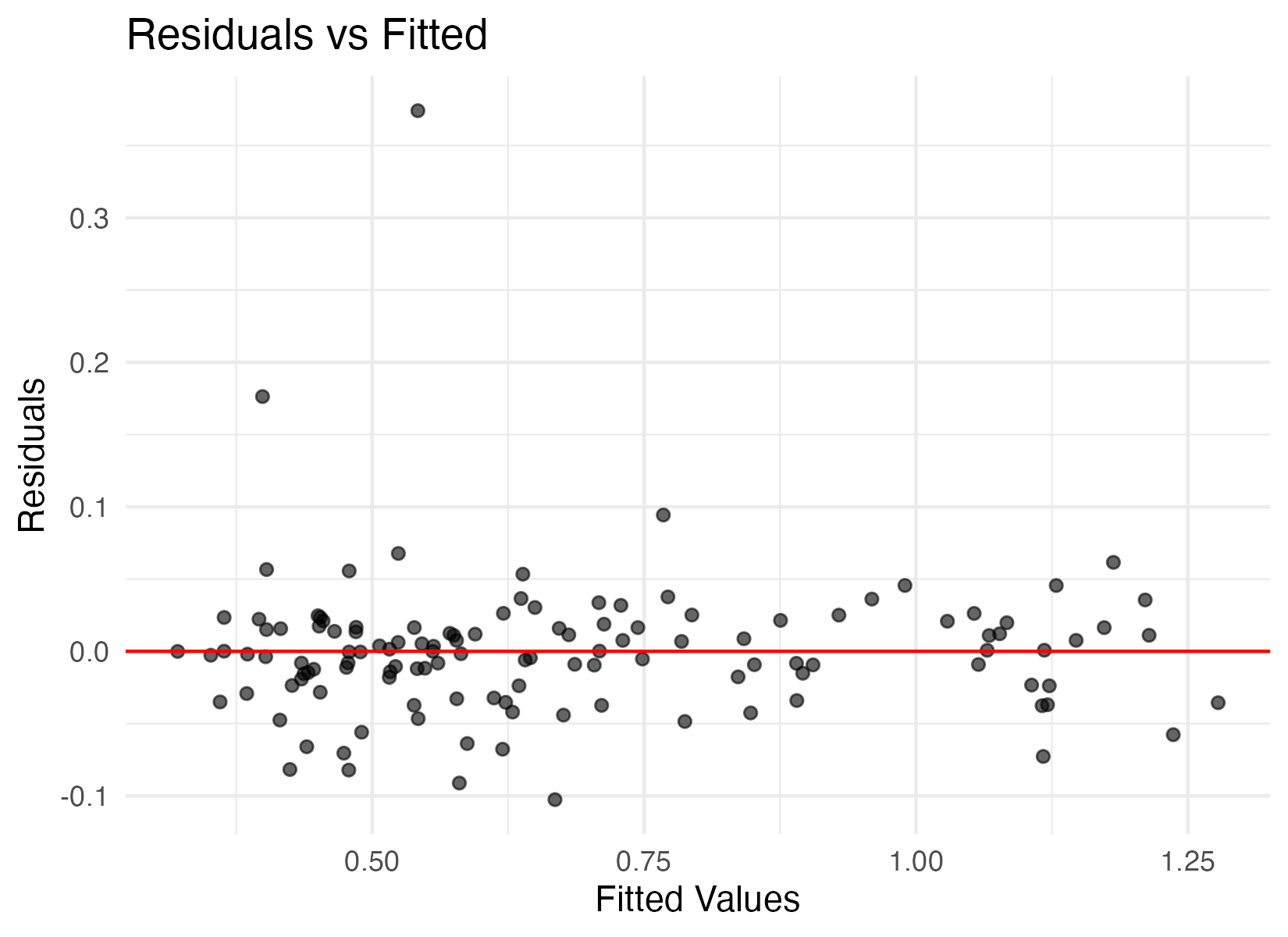


## QQ Plot


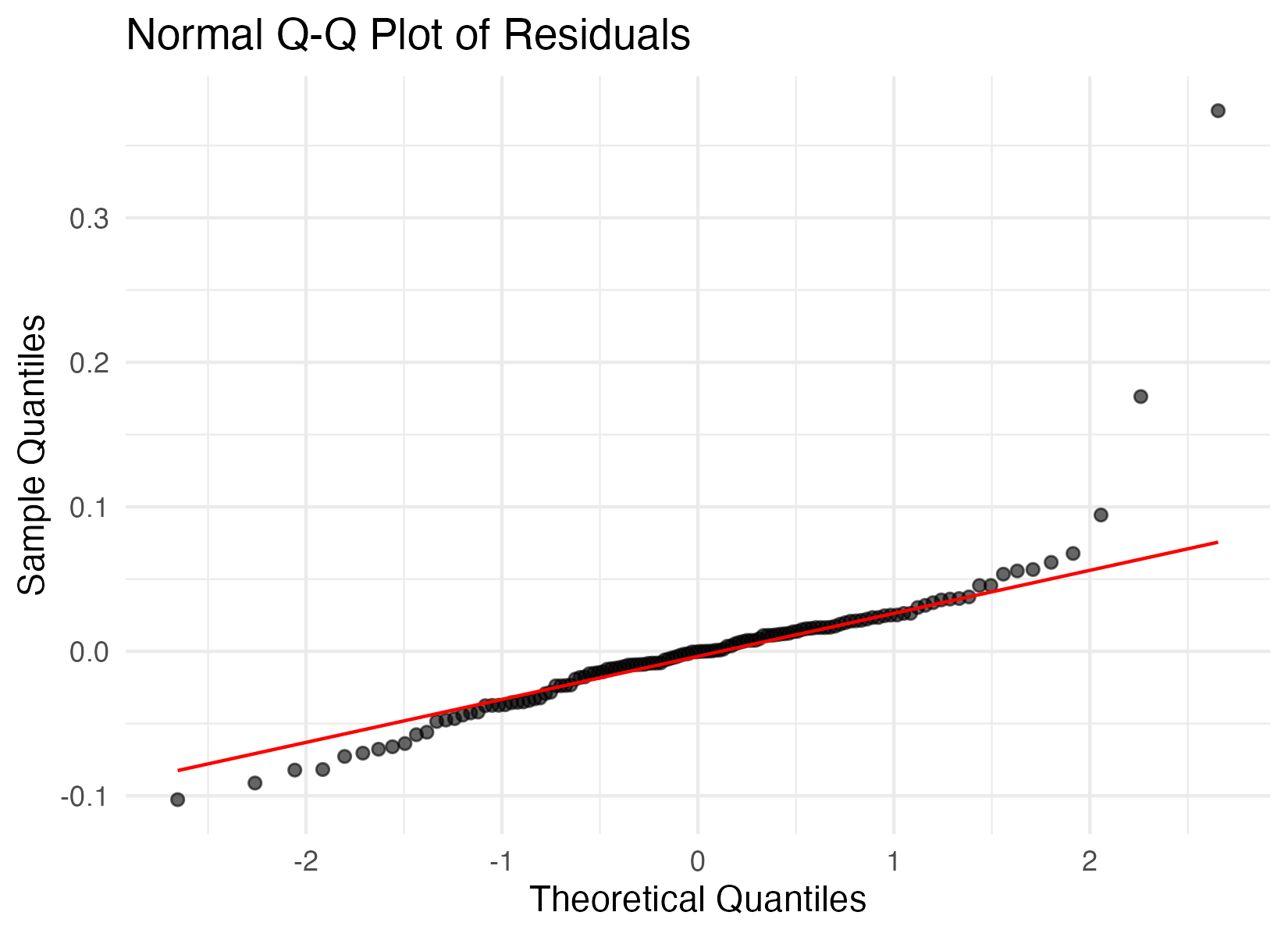


## Within-group change (baseline to follow-up)

| contrast | group | estimate | SE | df | lower.CL | upper.CL | t.ratio | p.value | effect_size |
| --- | --- | --- | --- | --- | --- | --- | --- | --- | --- |
| followup - baseline | C | 0.064 | 0.013 | 115 | 0.038 | 0.089 | 4.92 | <0.001 | 0.249 |
| followup - baseline | S | 0.039 | 0.012 | 115 | 0.015 | 0.063 | 3.17 | 0.002 | 0.153 |

## Between-group difference in change (interaction)

| timepoint_revpairwise | group_revpairwise | estimate | SE | df | lower.CL | upper.CL | t.ratio | p.value | effect_size |
| --- | --- | --- | --- | --- | --- | --- | --- | --- | --- |
| followup - baseline | S - C | -0.024 | 0.018 | 115 | -0.06 | 0.011 | -1.373 | 0.172 | -0.096 |

## Adjusted Means Over Time (with 95% CI)


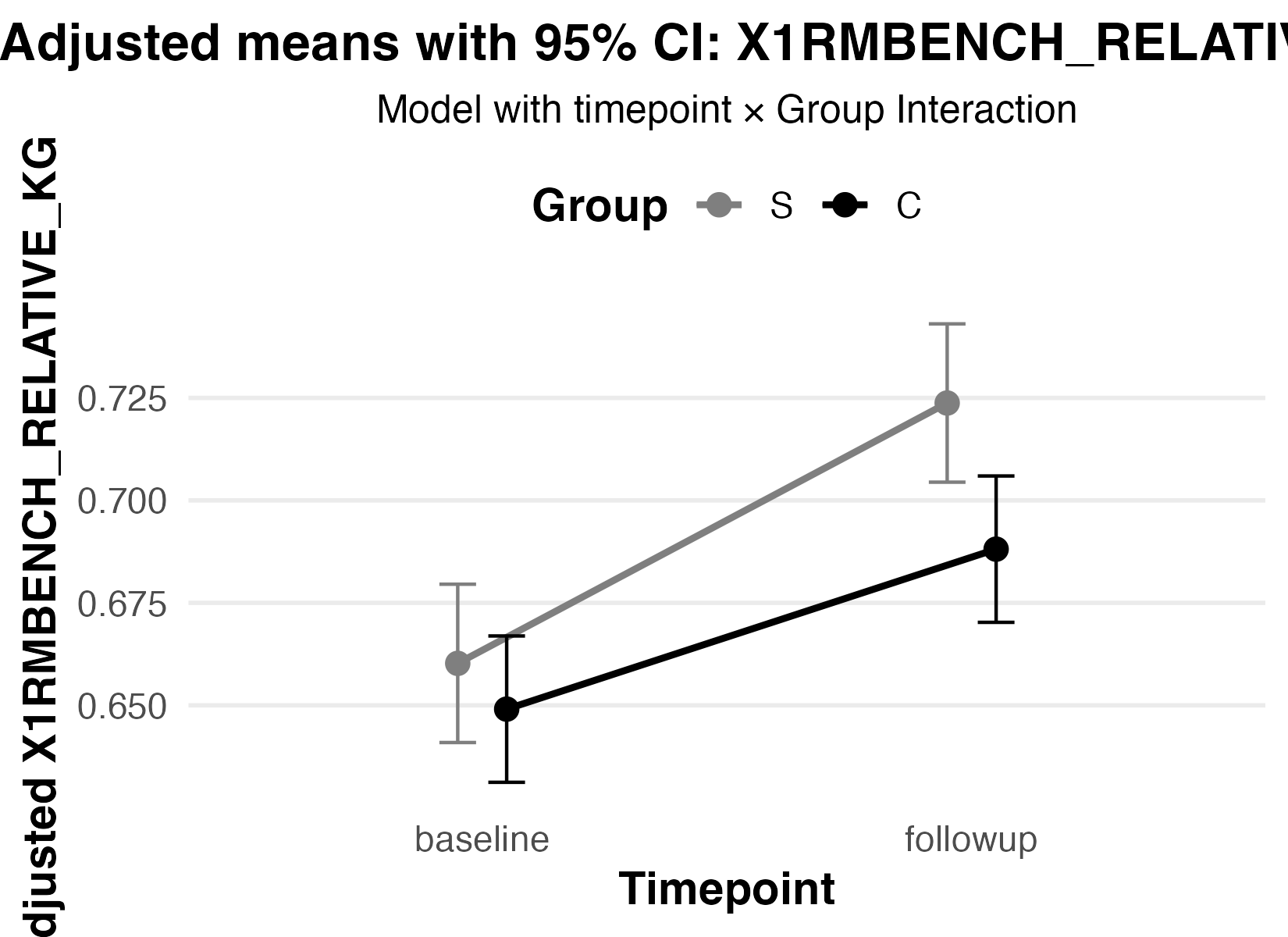


# Outcome: bodymass_kg

## Number of Participants Included: 63

## Distribution of DV at Baseline


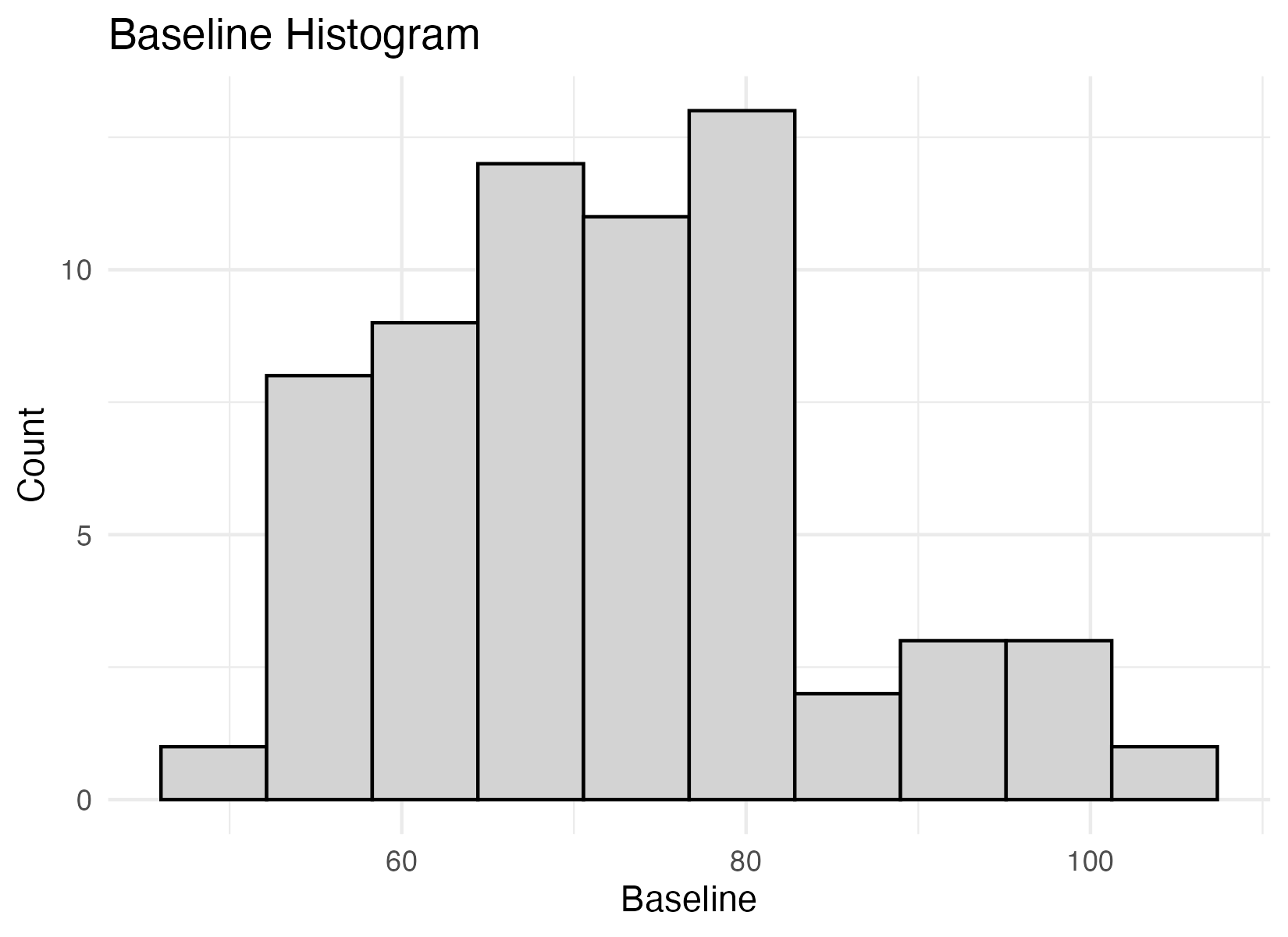


## Fitted vs Residuals


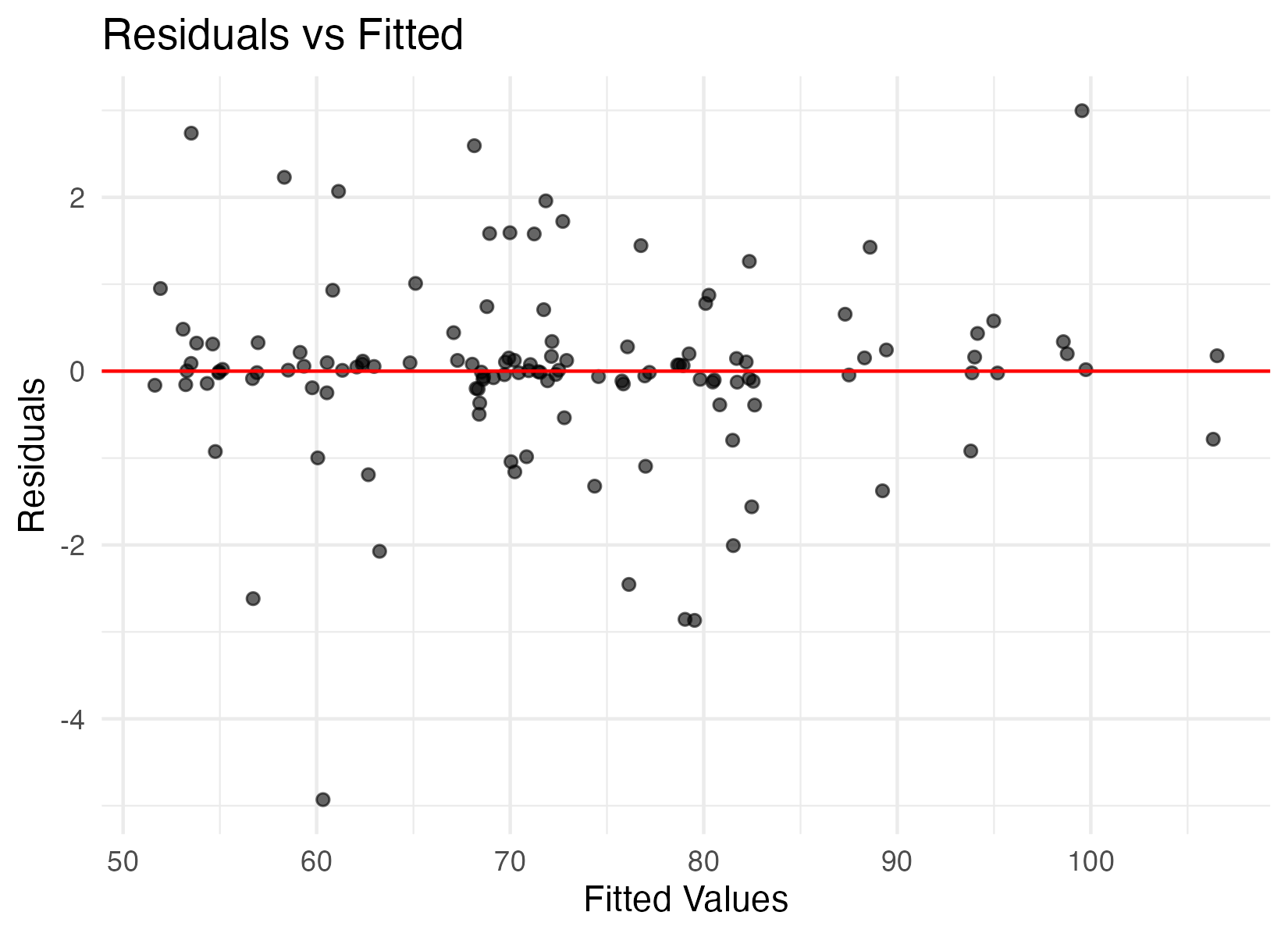


## QQ Plot


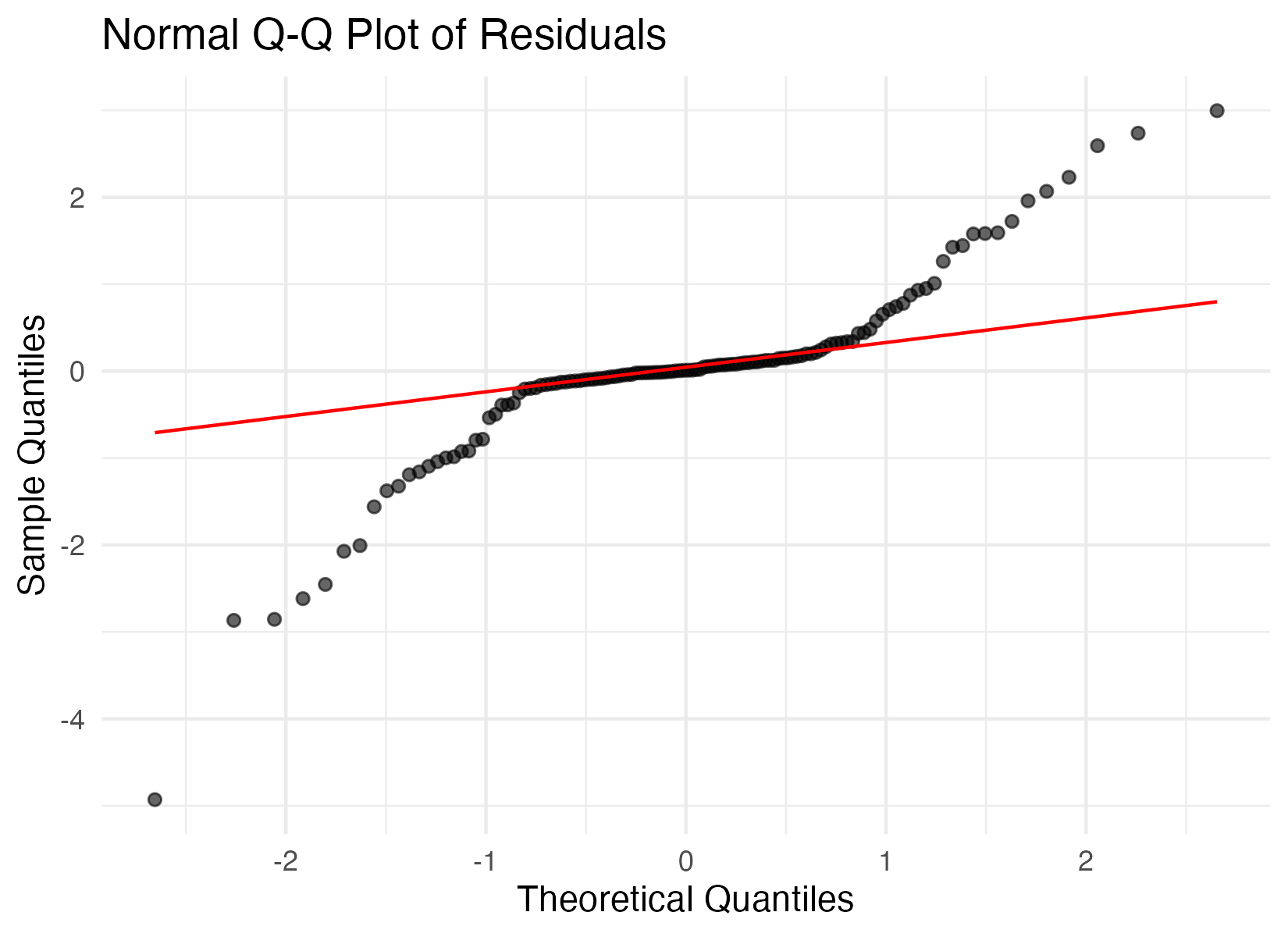


## Within-group change (baseline to follow-up)

| contrast | group | estimate | SE | df | lower.CL | upper.CL | t.ratio | p.value | effect_size |
| --- | --- | --- | --- | --- | --- | --- | --- | --- | --- |
| followup - baseline | C | 0.287 | 0.272 | 115 | -0.251 | 0.825 | 1.055 | 0.294 | 0.023 |
| followup - baseline | S | -0.199 | 0.259 | 115 | -0.713 | 0.314 | -0.770 | 0.443 | -0.016 |

## Between-group difference in change (interaction)

| timepoint_revpairwise | group_revpairwise | estimate | SE | df | lower.CL | upper.CL | t.ratio | p.value | effect_size |
| --- | --- | --- | --- | --- | --- | --- | --- | --- | --- |
| followup - baseline | S - C | -0.486 | 0.375 | 115 | -1.23 | 0.258 | -1.295 | 0.198 | -0.038 |

## Adjusted Means Over Time (with 95% CI)


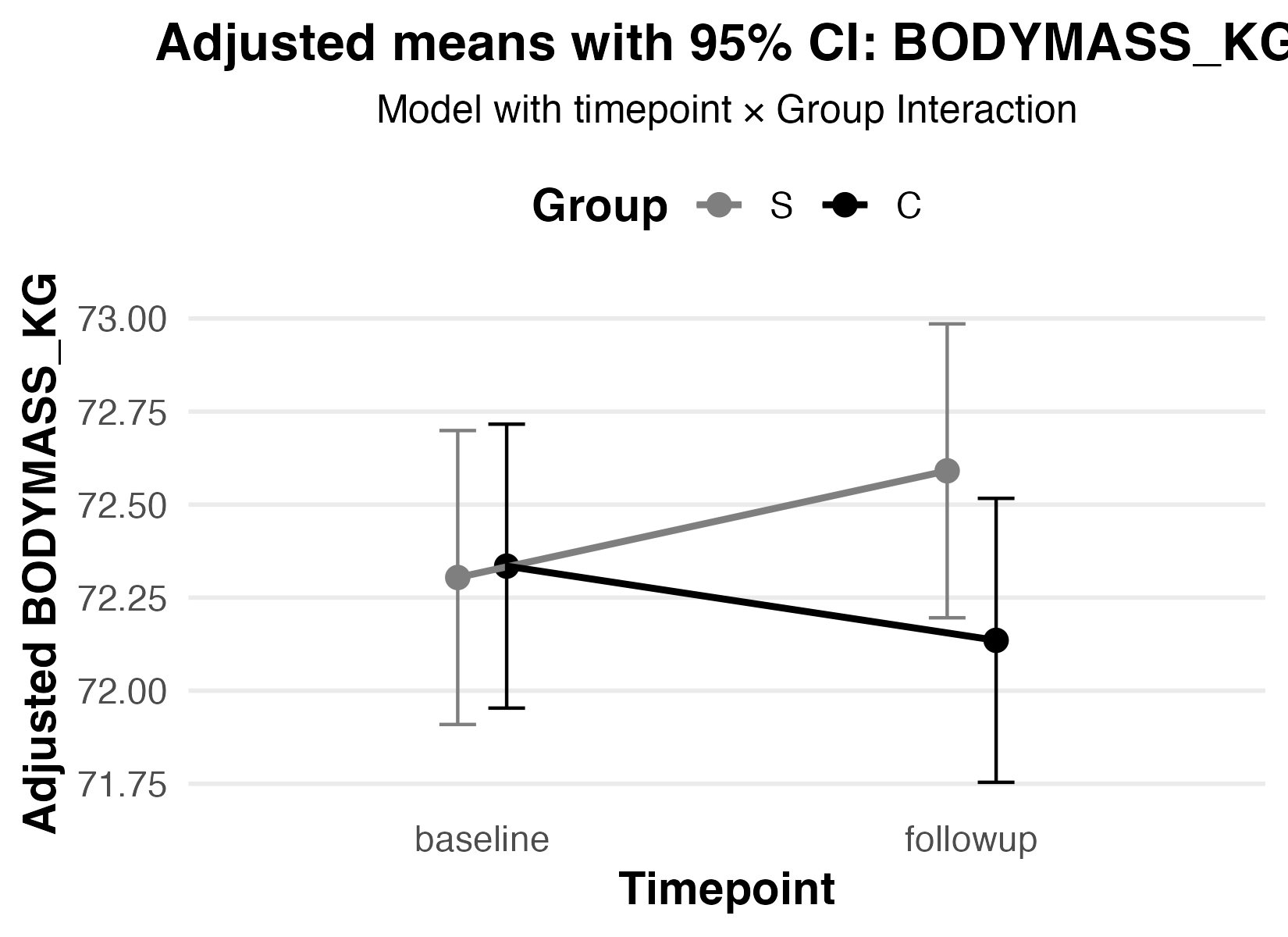


# Outcome: bmi

## Number of Participants Included: 63

## Distribution of DV at Baseline


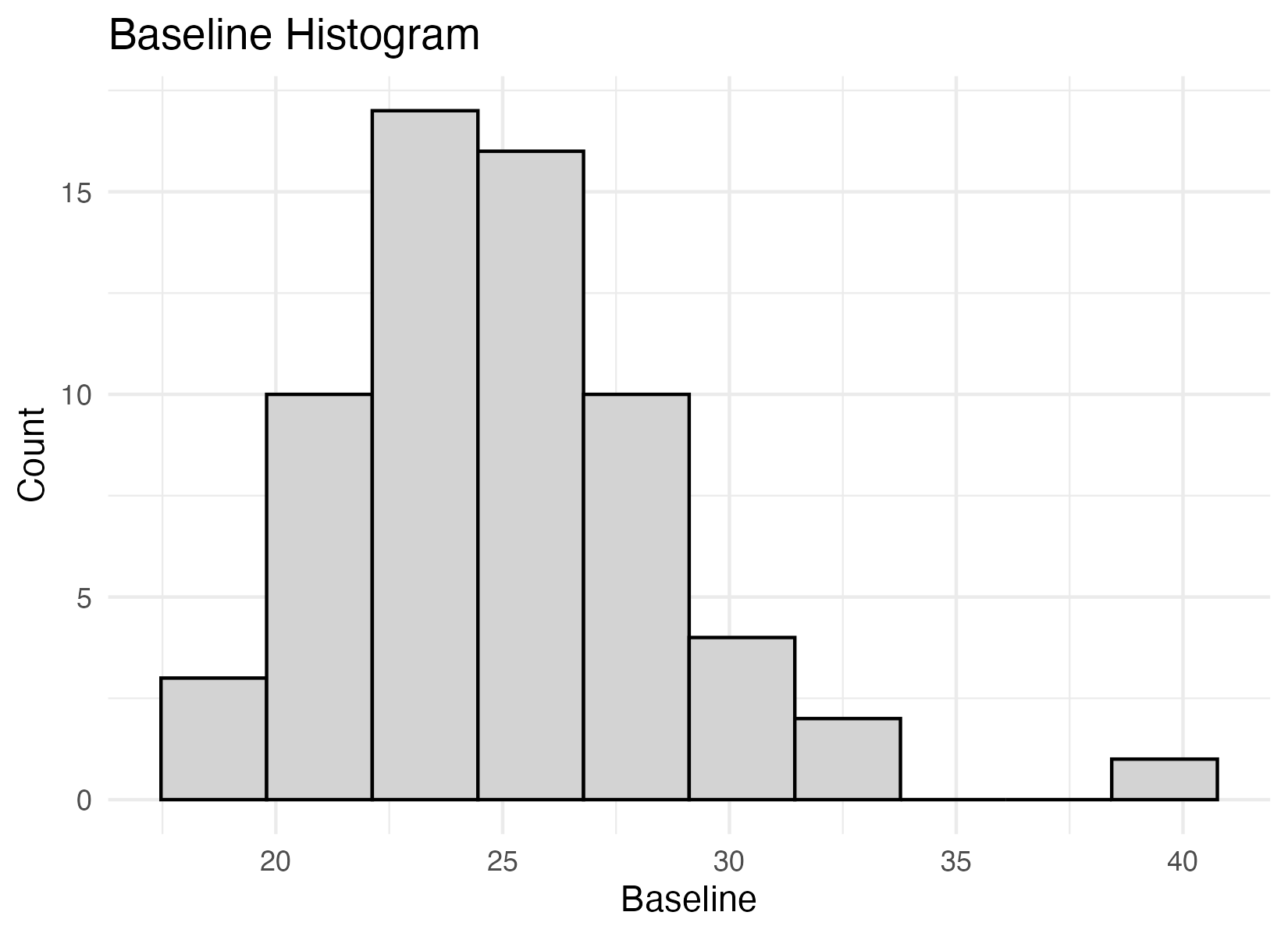


## Fitted vs Residuals


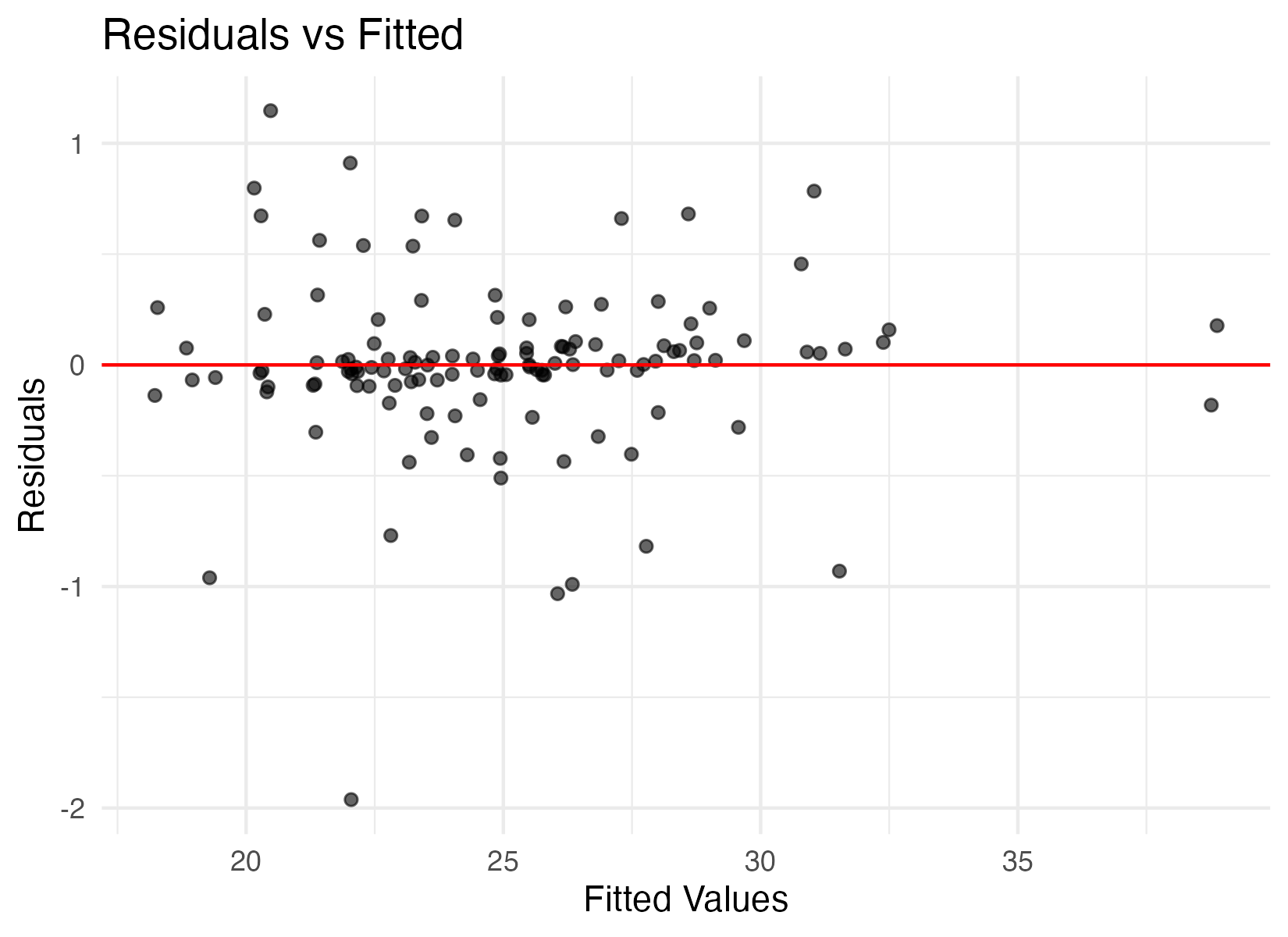


## QQ Plot


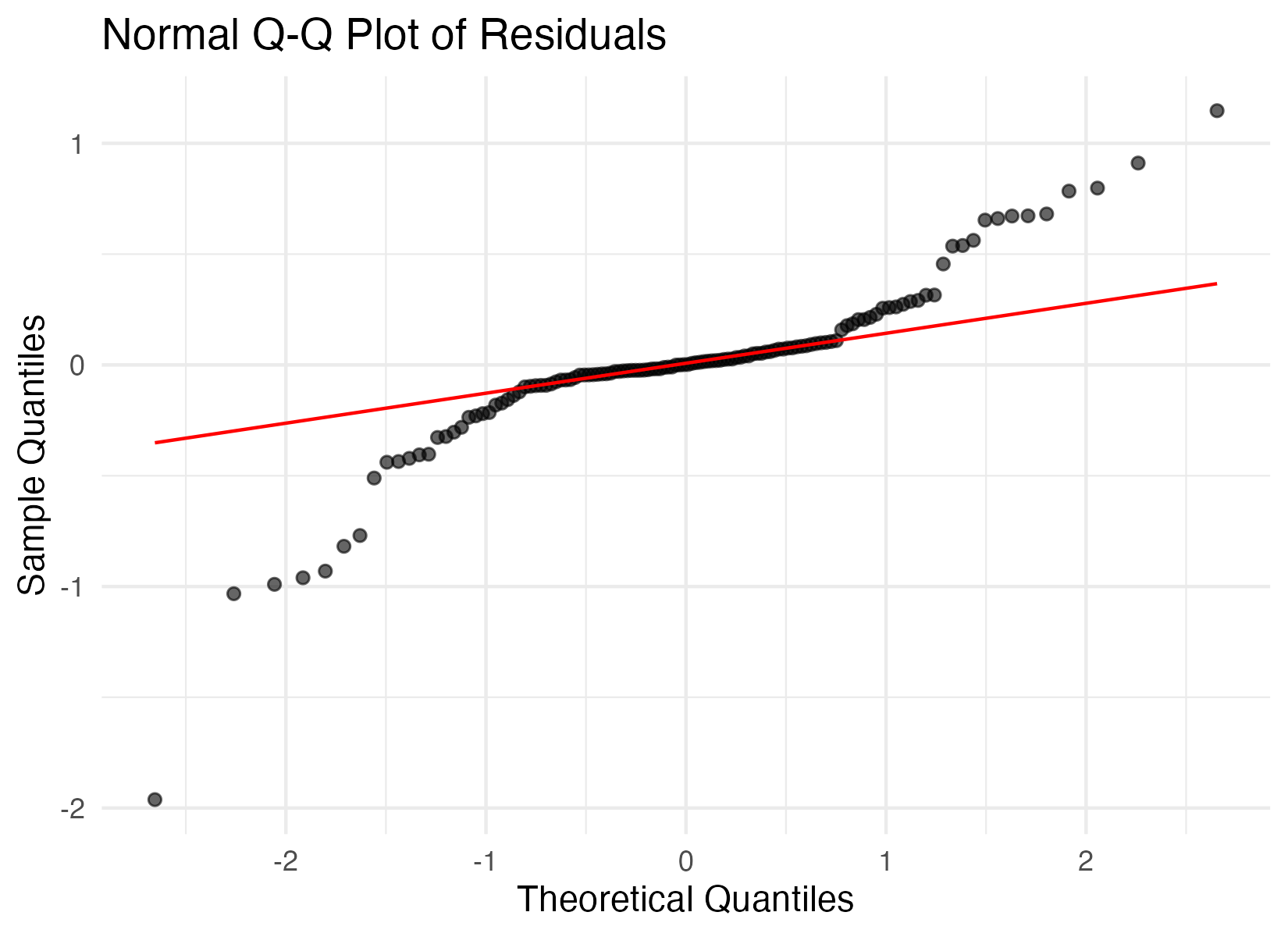


## Within-group change (baseline to follow-up)

| contrast | group | estimate | SE | df | lower.CL | upper.CL | t.ratio | p.value | effect_size |
| --- | --- | --- | --- | --- | --- | --- | --- | --- | --- |
| followup - baseline | C | 0.051 | 0.099 | 115 | -0.146 | 0.248 | 0.517 | 0.606 | 0.014 |
| followup - baseline | S | -0.112 | 0.095 | 115 | -0.300 | 0.075 | -1.186 | 0.238 | -0.031 |

## Between-group difference in change (interaction)

| timepoint_revpairwise | group_revpairwise | estimate | SE | df | lower.CL | upper.CL | t.ratio | p.value | effect_size |
| --- | --- | --- | --- | --- | --- | --- | --- | --- | --- |
| followup - baseline | S - C | -0.164 | 0.137 | 115 | -0.436 | 0.108 | -1.192 | 0.236 | -0.045 |

## Adjusted Means Over Time (with 95% CI)


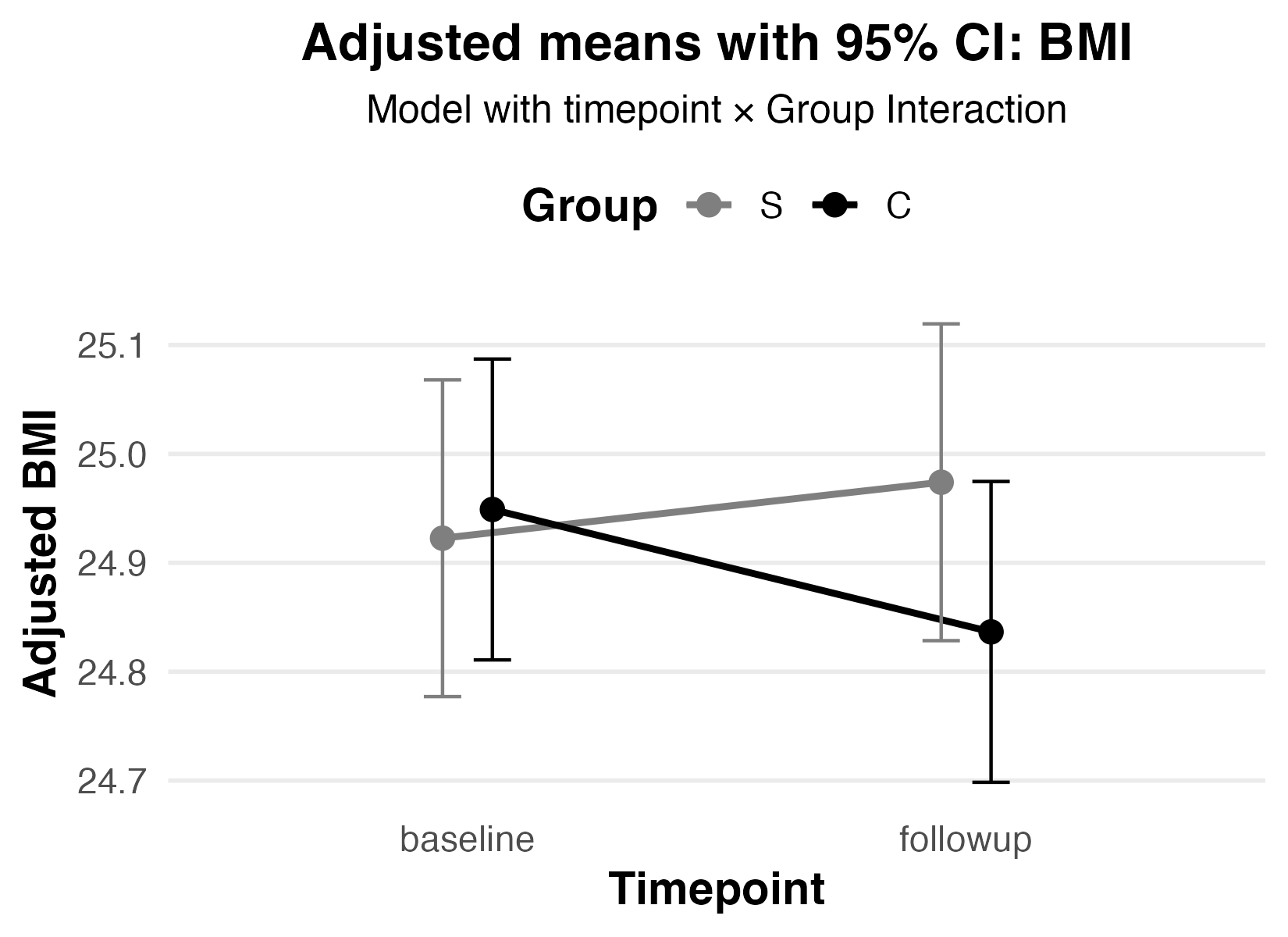


# Outcome: waist_cm

## Number of Participants Included: 63

## Distribution of DV at Baseline


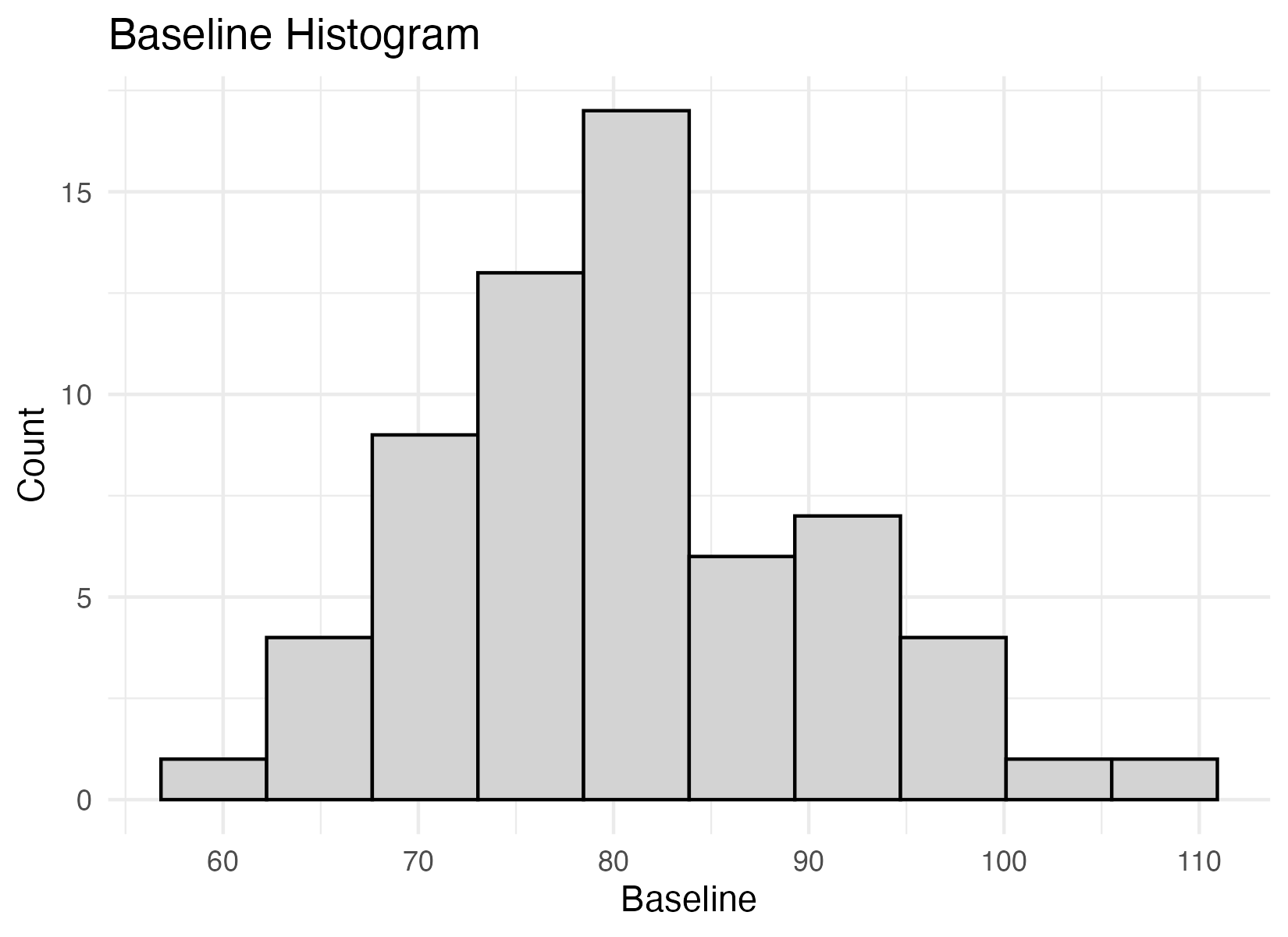


## Fitted vs Residuals


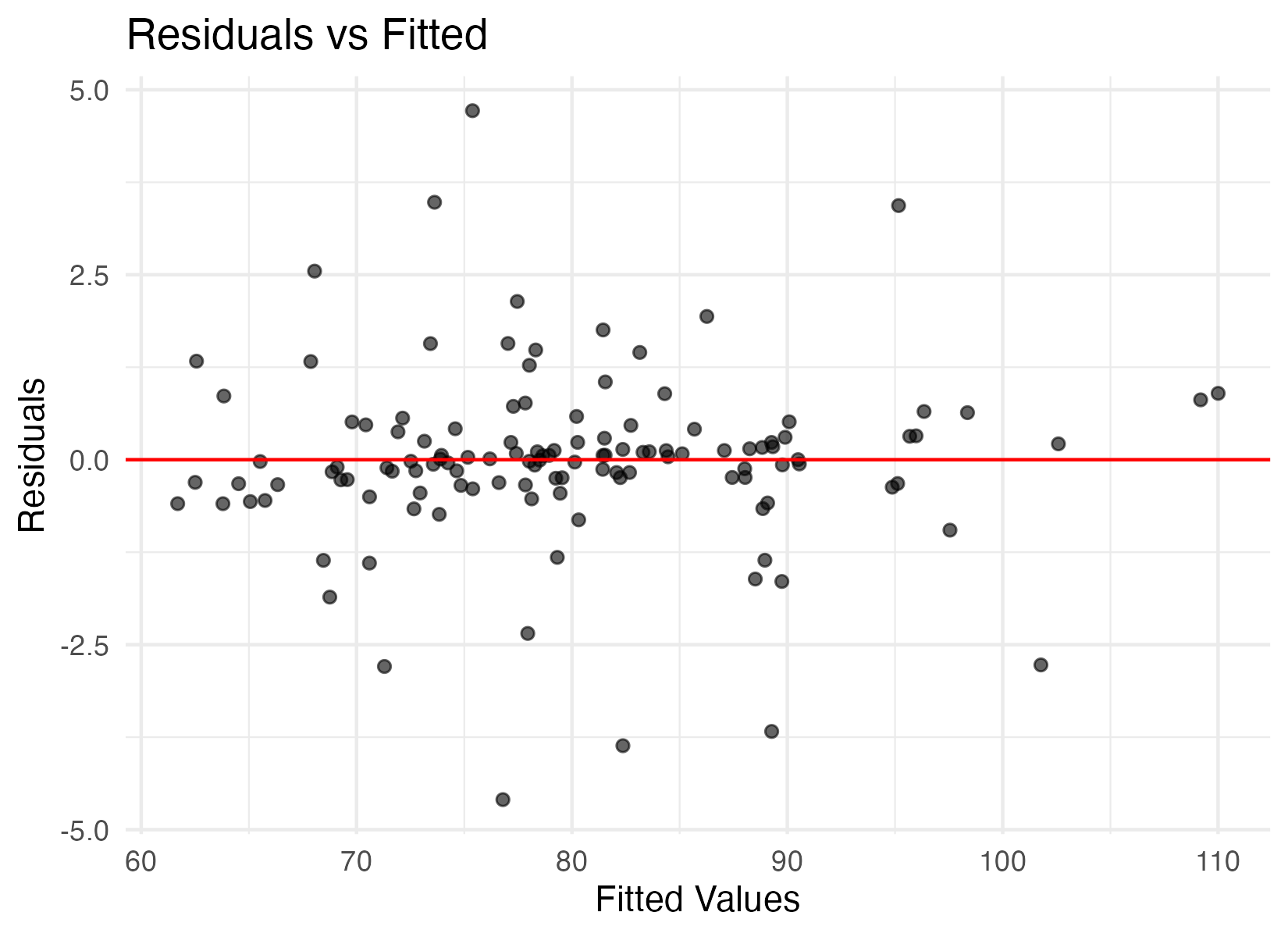


## QQ Plot


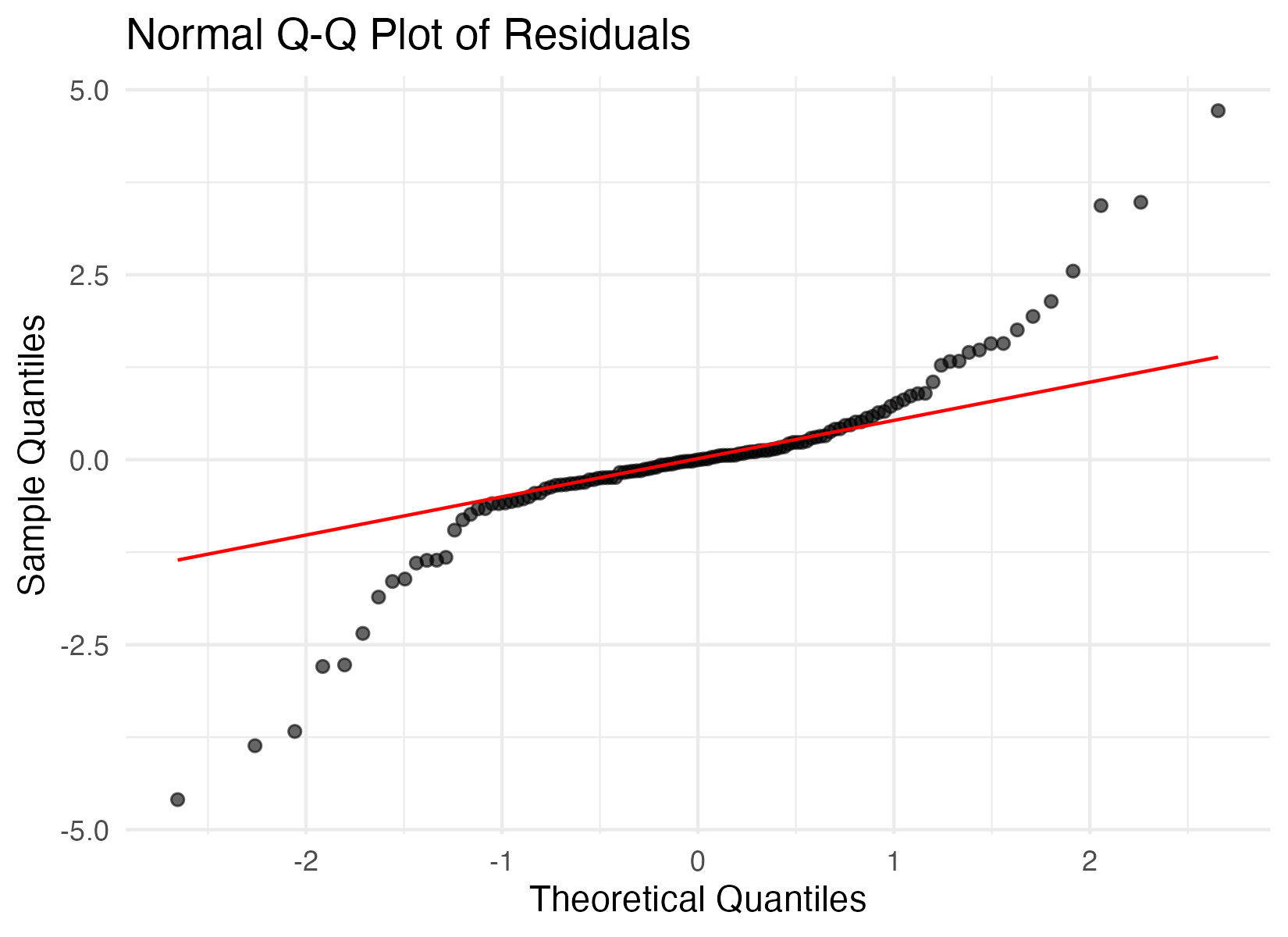


## Within-group change (baseline to follow-up)

| contrast | group | estimate | SE | df | lower.CL | upper.CL | t.ratio | p.value | effect_size |
| --- | --- | --- | --- | --- | --- | --- | --- | --- | --- |
| followup - baseline | C | -1.227 | 0.310 | 115 | -1.840 | -0.613 | -3.959 | <0.001 | -0.126 |
| followup - baseline | S | -0.812 | 0.295 | 115 | -1.397 | -0.227 | -2.749 | 0.007 | -0.083 |

## Between-group difference in change (interaction)

| timepoint_revpairwise | group_revpairwise | estimate | SE | df | lower.CL | upper.CL | t.ratio | p.value | effect_size |
| --- | --- | --- | --- | --- | --- | --- | --- | --- | --- |
| followup - baseline | S - C | 0.415 | 0.428 | 115 | -0.434 | 1.263 | 0.968 | 0.335 | 0.043 |

## Adjusted Means Over Time (with 95% CI)


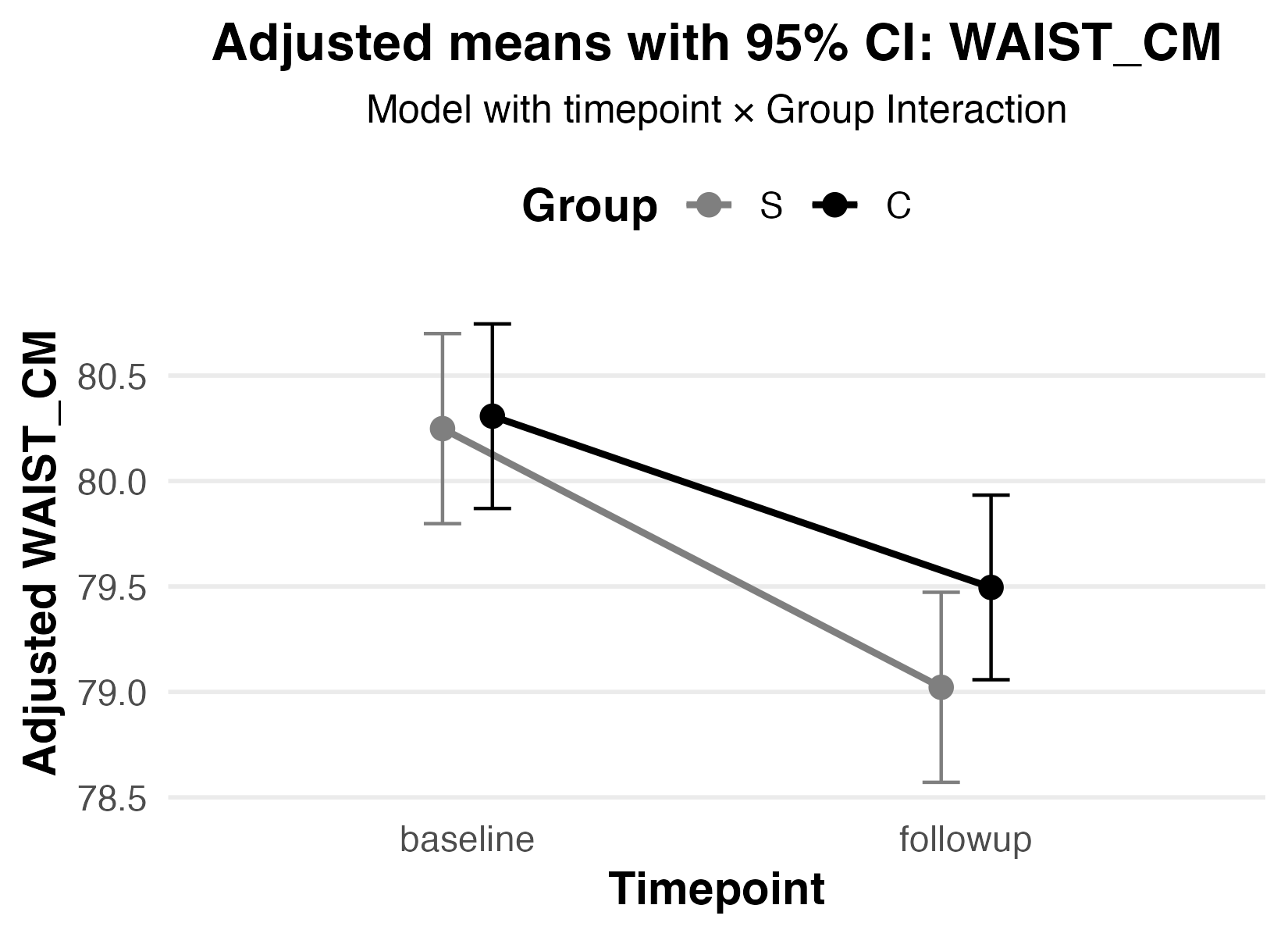


# Outcome: hip_cm

## Number of Participants Included: 63

## Distribution of DV at Baseline


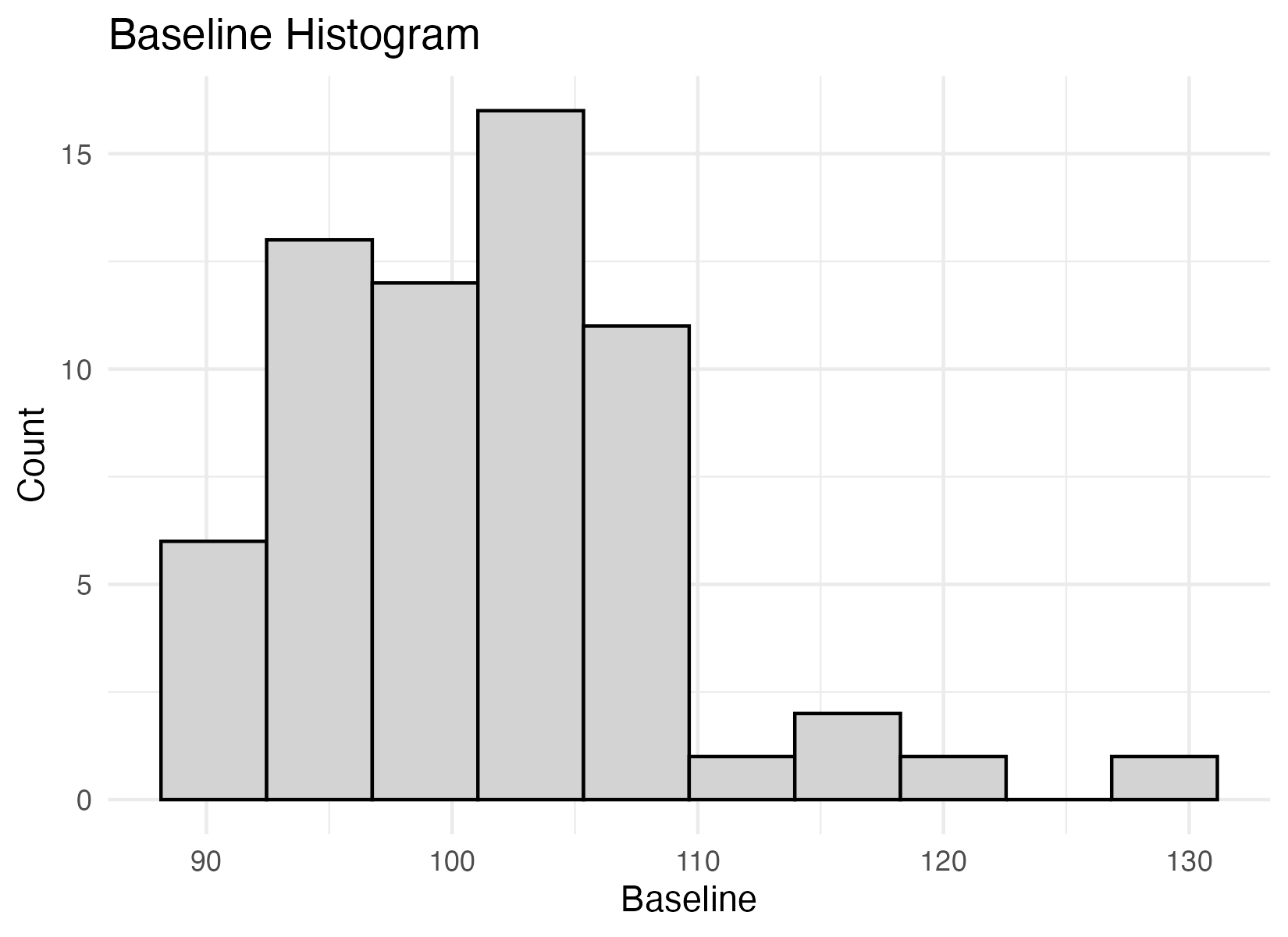


## Fitted vs Residuals


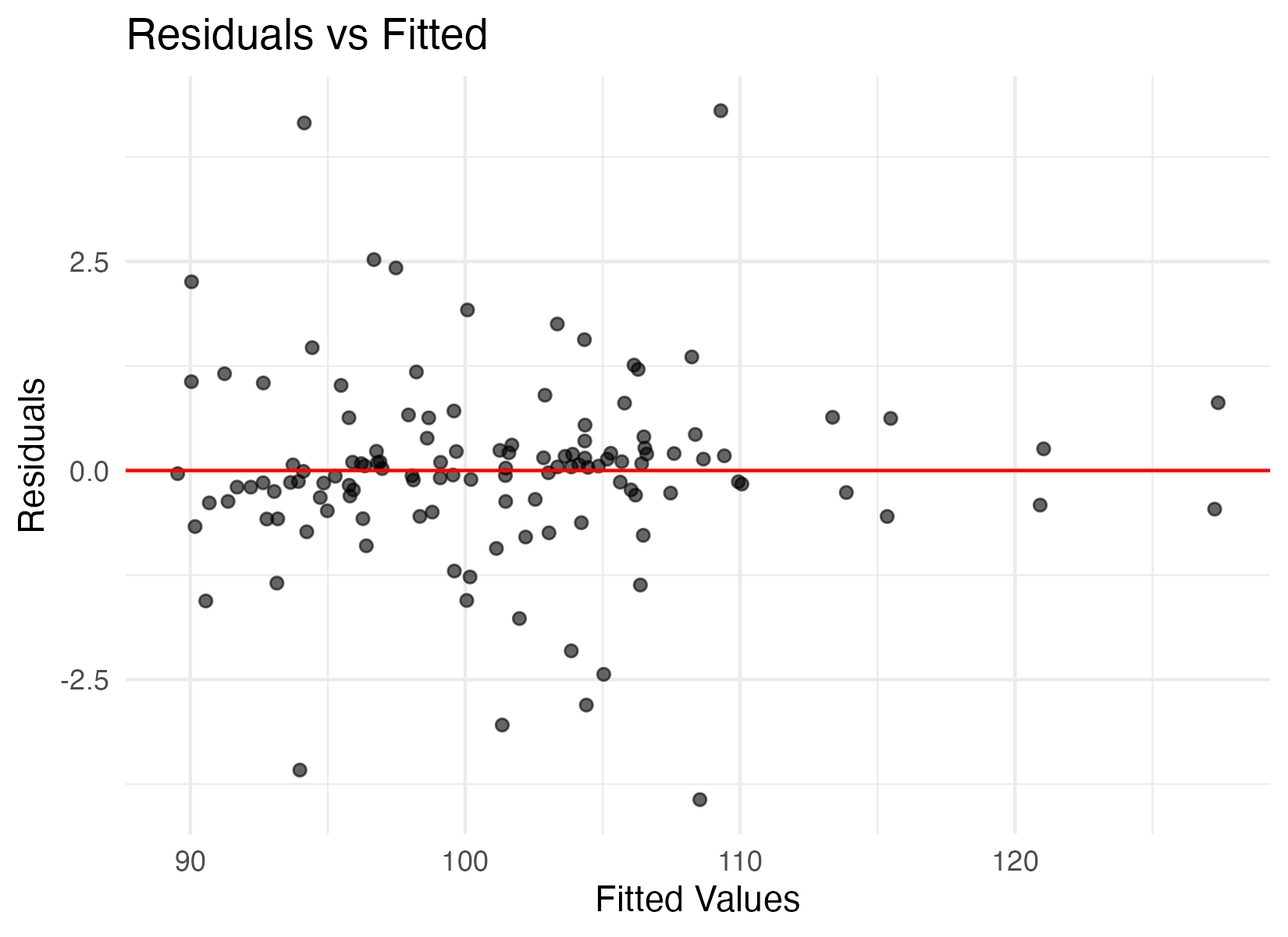


## QQ Plot


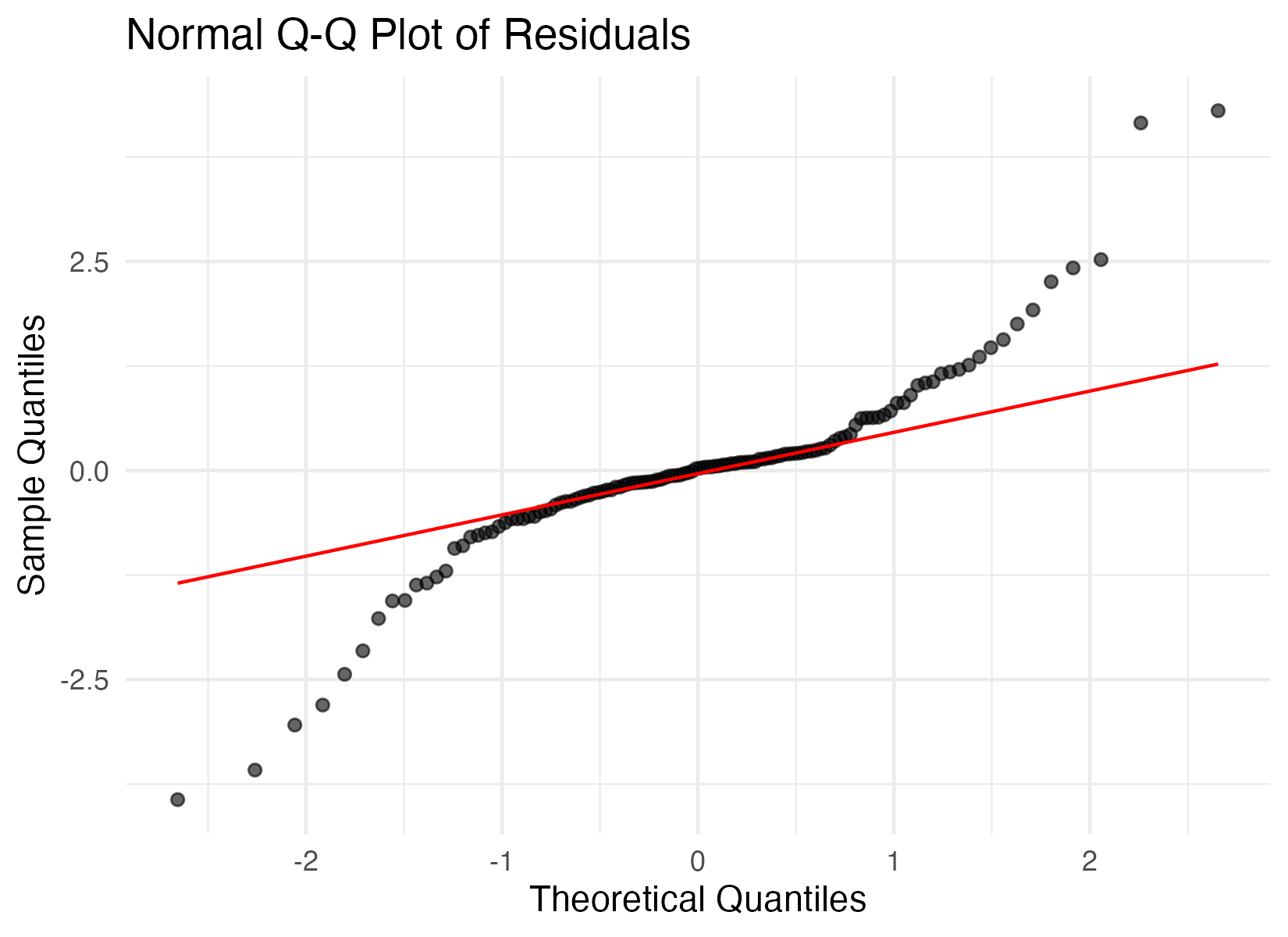


## Within-group change (baseline to follow-up)

| contrast | group | estimate | SE | df | lower.CL | upper.CL | t.ratio | p.value | effect_size |
| --- | --- | --- | --- | --- | --- | --- | --- | --- | --- |
| followup - baseline | C | 0.500 | 0.292 | 115 | -0.079 | 1.079 | 1.711 | 0.090 | 0.068 |
| followup - baseline | S | -0.127 | 0.279 | 115 | -0.679 | 0.425 | -0.457 | 0.649 | -0.017 |

## Between-group difference in change (interaction)

| timepoint_revpairwise | group_revpairwise | estimate | SE | df | lower.CL | upper.CL | t.ratio | p.value | effect_size |
| --- | --- | --- | --- | --- | --- | --- | --- | --- | --- |
| followup - baseline | S - C | -0.627 | 0.404 | 115 | -1.427 | 0.172 | -1.554 | 0.123 | -0.086 |

## Adjusted Means Over Time (with 95% CI)


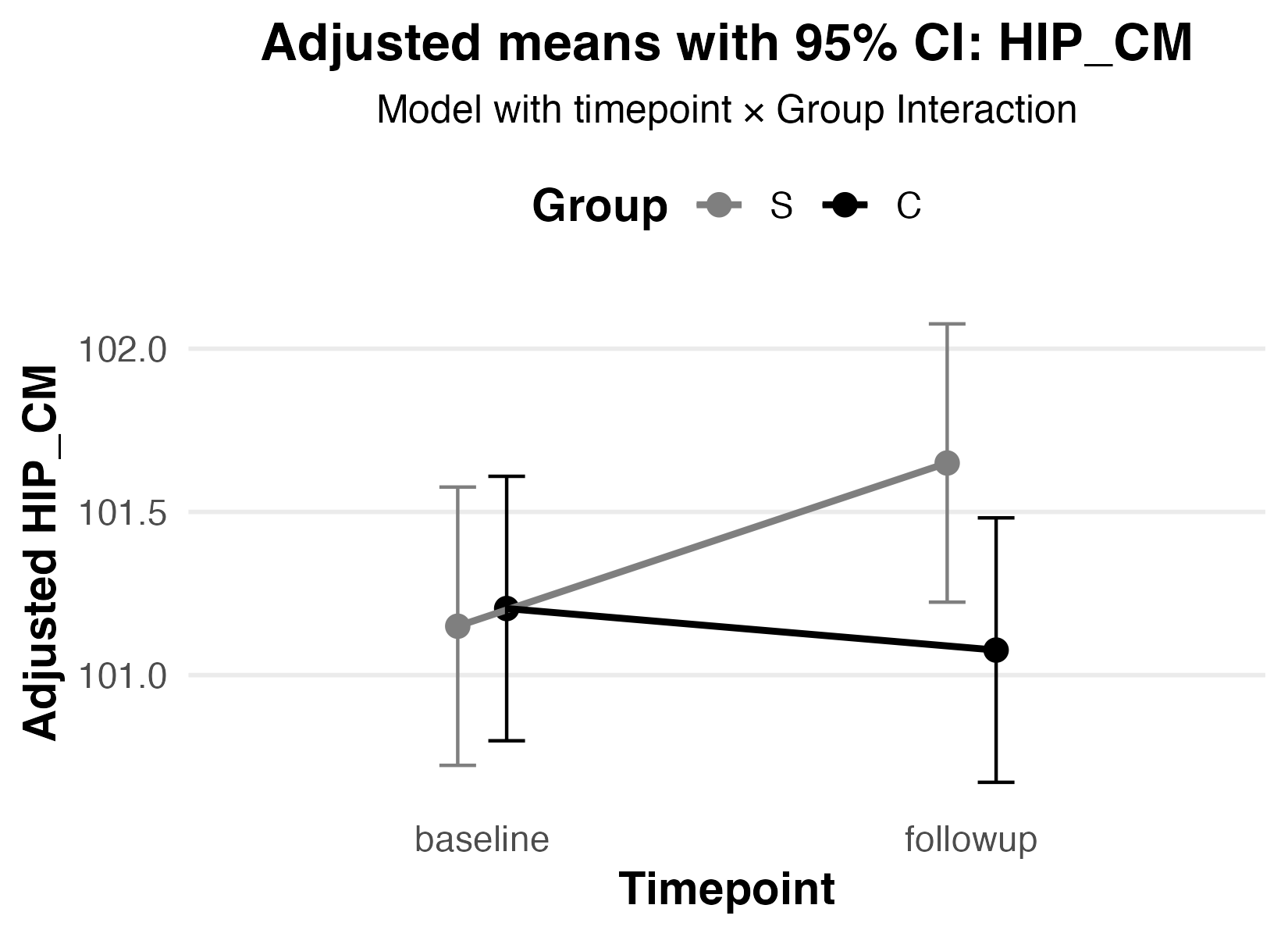


# Outcome: whr

## Number of Participants Included: 63

## Distribution of DV at Baseline


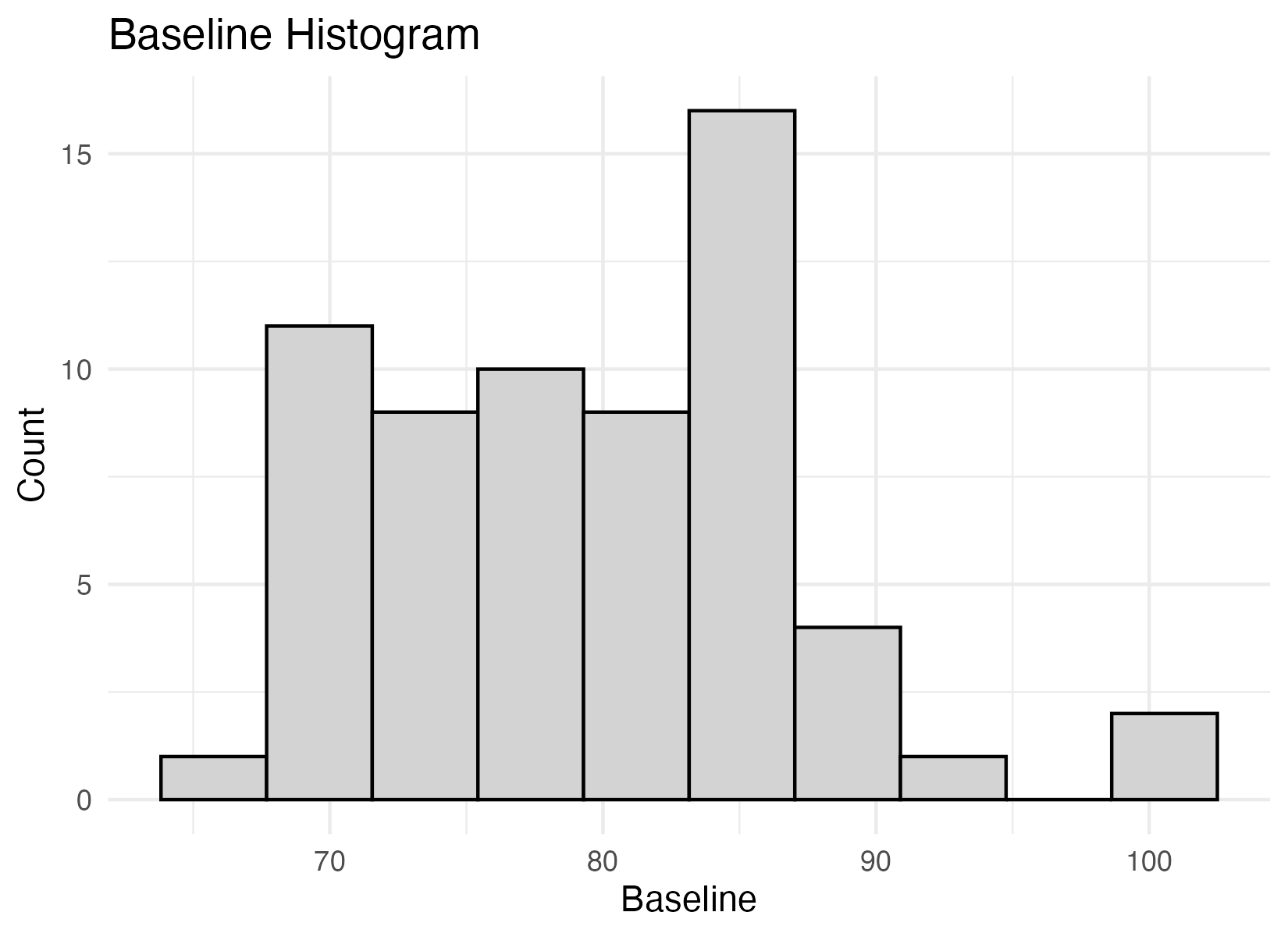


## Fitted vs Residuals


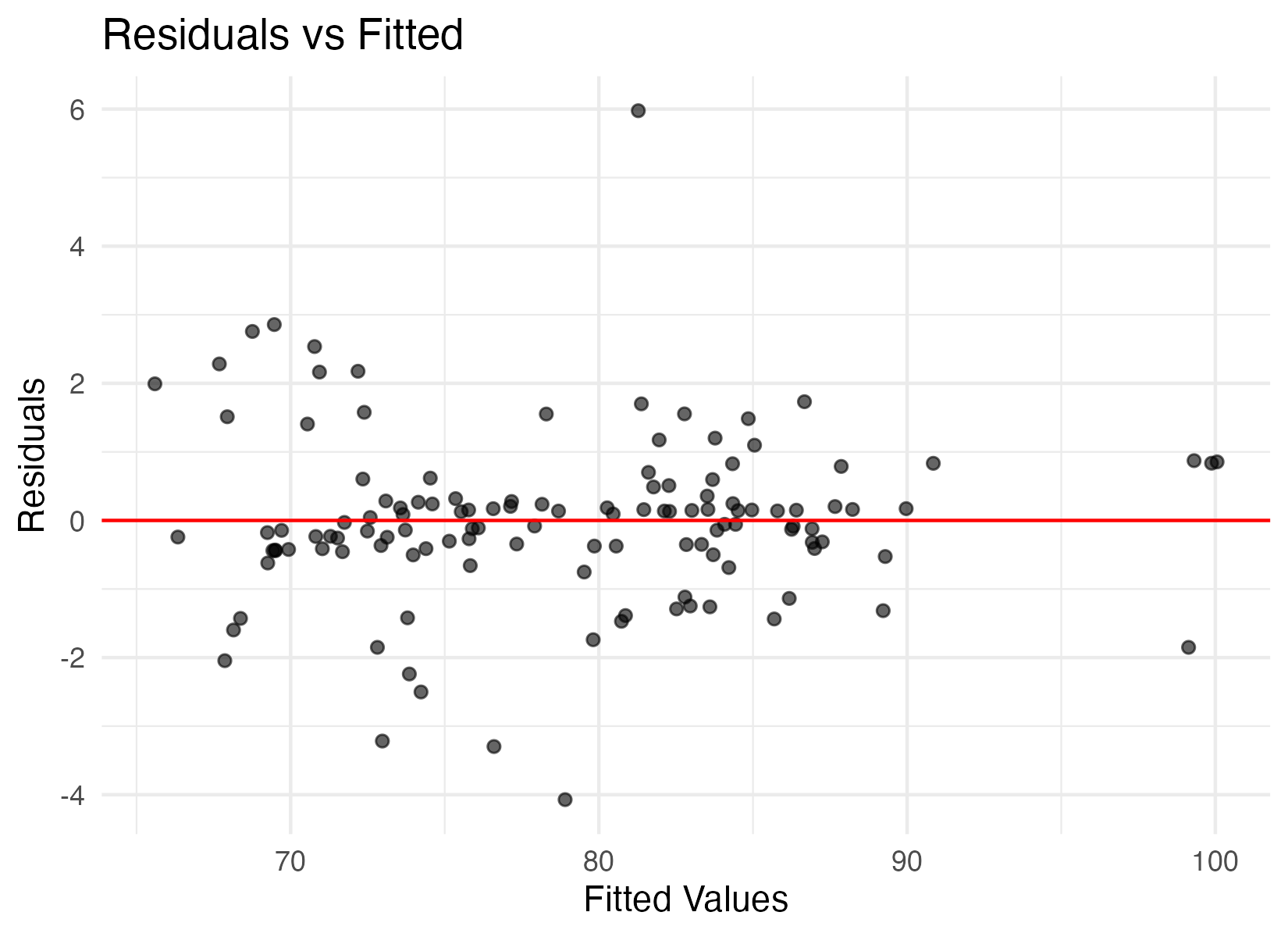


## QQ Plot


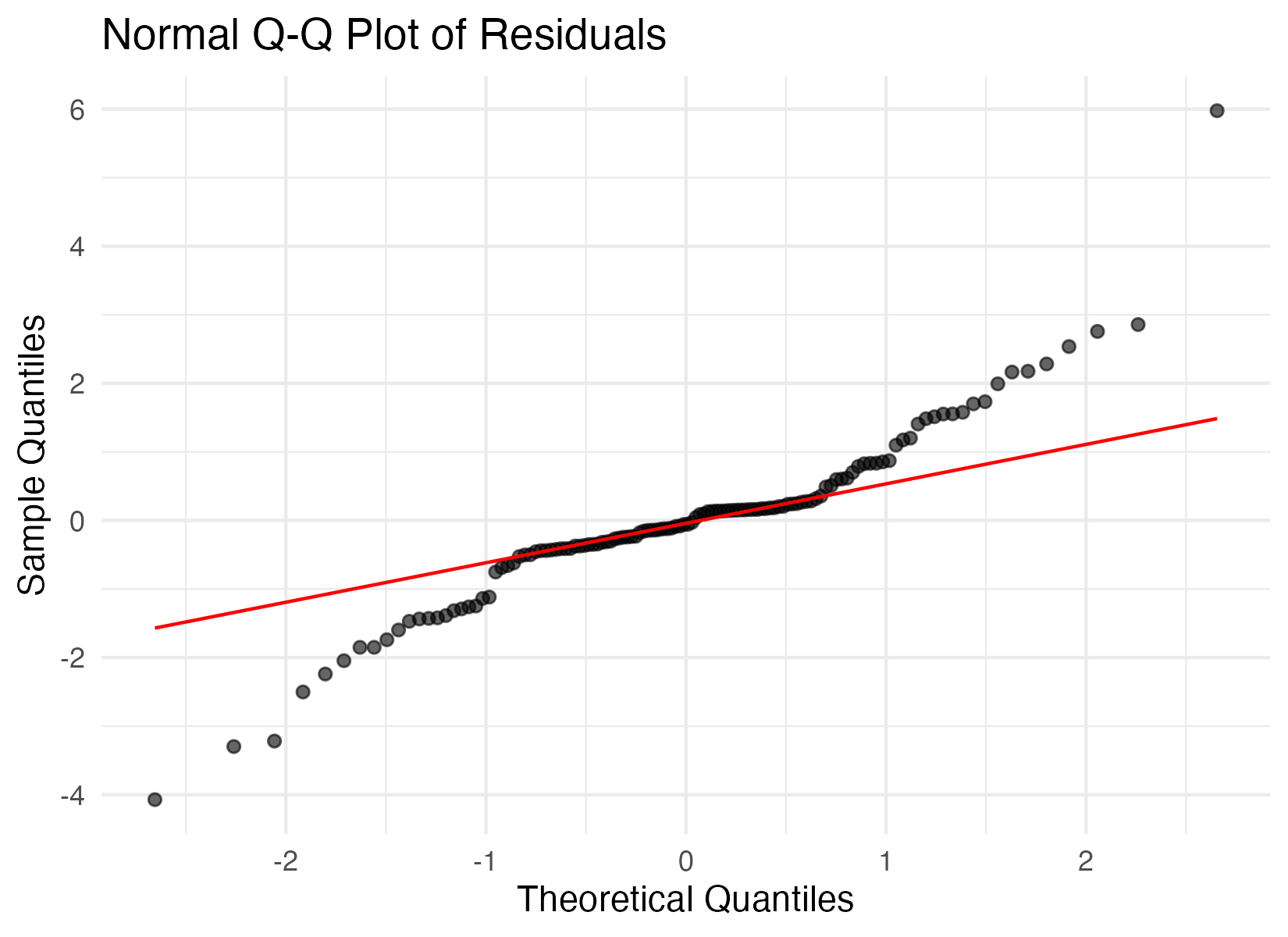


## Within-group change (baseline to follow-up)

| contrast | group | estimate | SE | df | lower.CL | upper.CL | t.ratio | p.value | effect_size |
| --- | --- | --- | --- | --- | --- | --- | --- | --- | --- |
| followup - baseline | C | -1.559 | 0.324 | 115 | -2.201 | -0.917 | -4.813 | <0.001 | -0.209 |
| followup - baseline | S | -0.746 | 0.309 | 115 | -1.358 | -0.134 | -2.415 | 0.017 | -0.100 |

## Between-group difference in change (interaction)

| timepoint_revpairwise | group_revpairwise | estimate | SE | df | lower.CL | upper.CL | t.ratio | p.value | effect_size |
| --- | --- | --- | --- | --- | --- | --- | --- | --- | --- |
| followup - baseline | S - C | 0.813 | 0.448 | 115 | -0.073 | 1.7 | 1.817 | 0.072 | 0.109 |

## Adjusted Means Over Time (with 95% CI)


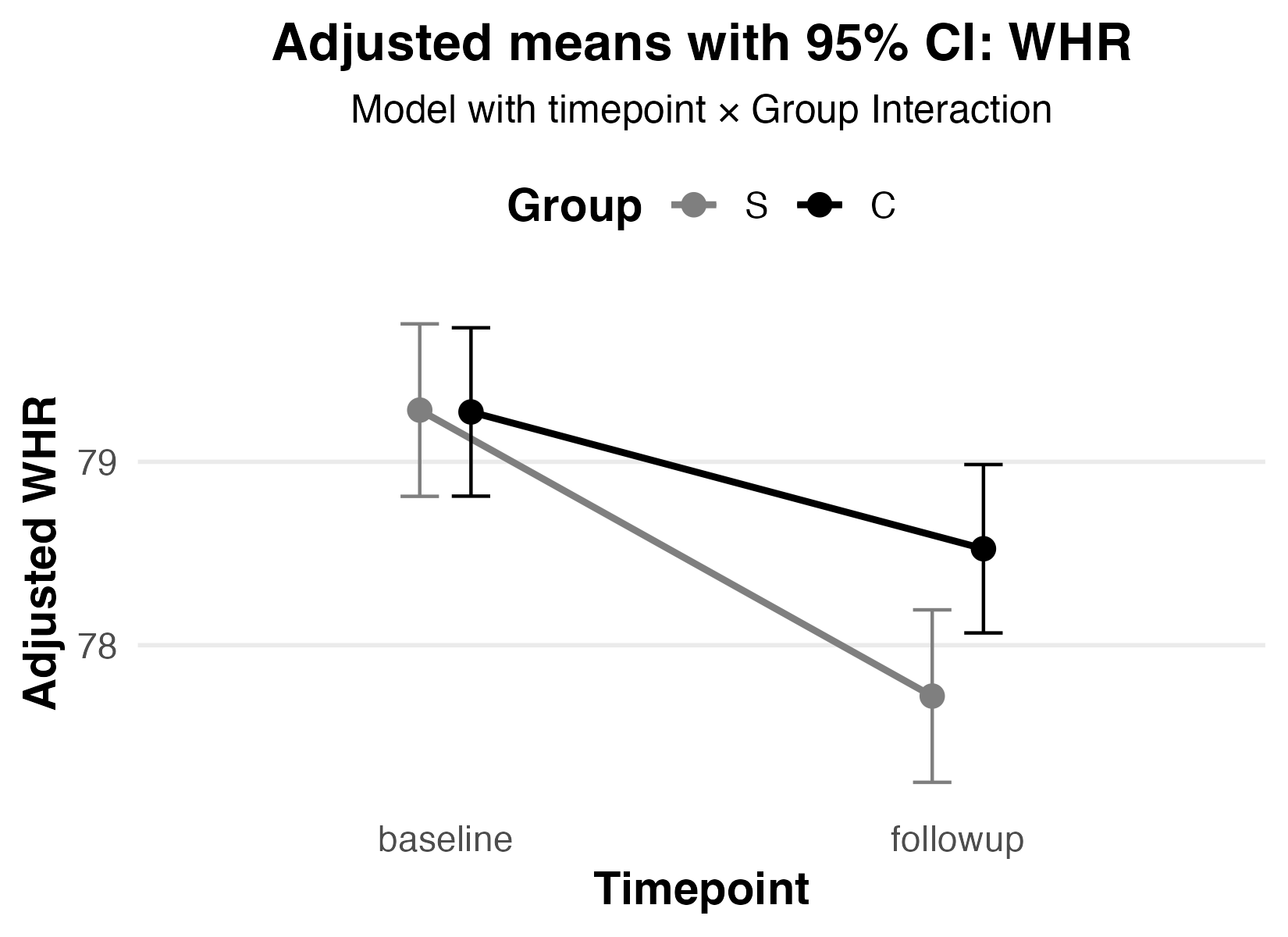


# Outcome: bonemass_kg

## Number of Participants Included: 63

## Distribution of DV at Baseline


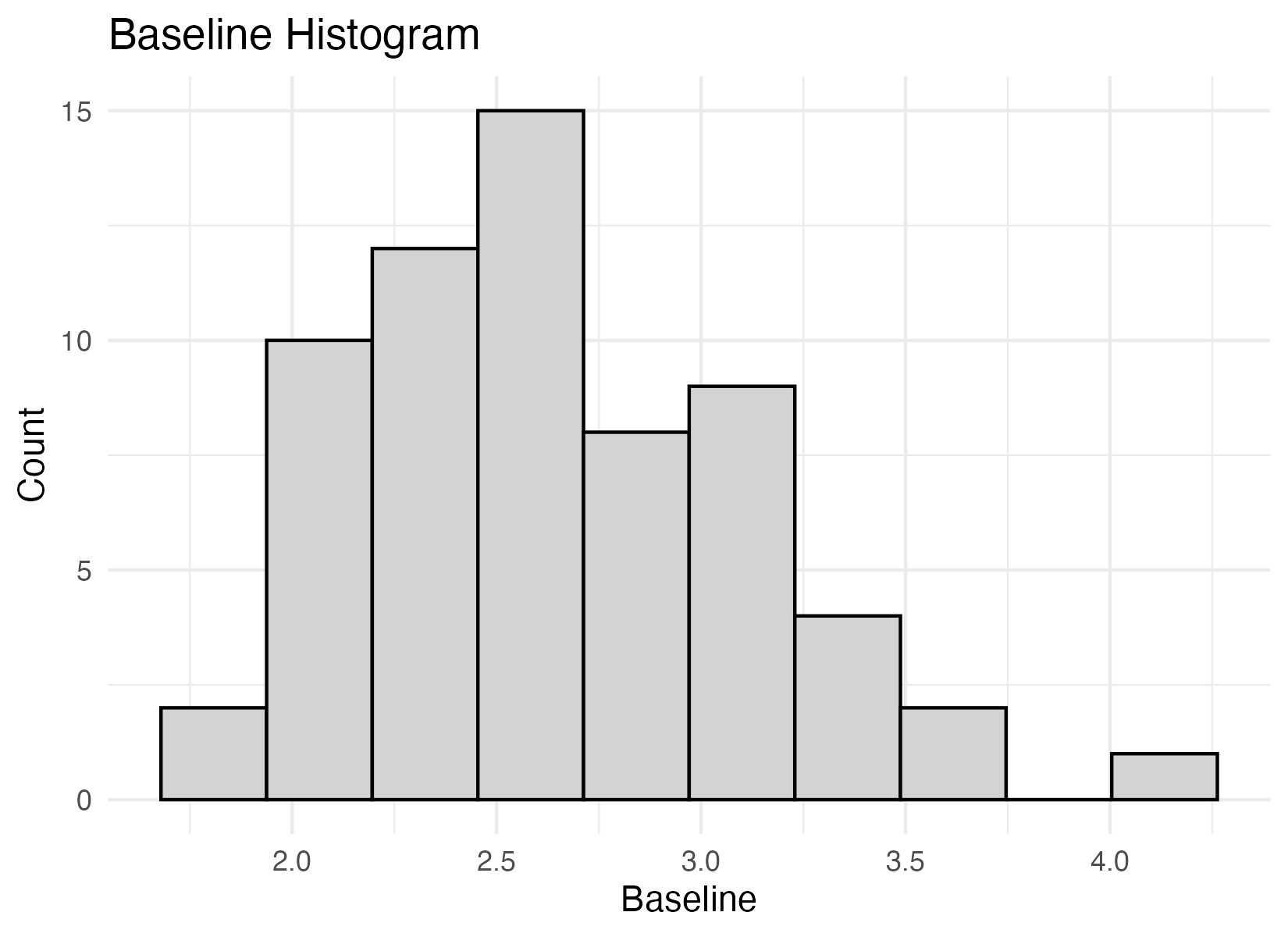


## Fitted vs Residuals


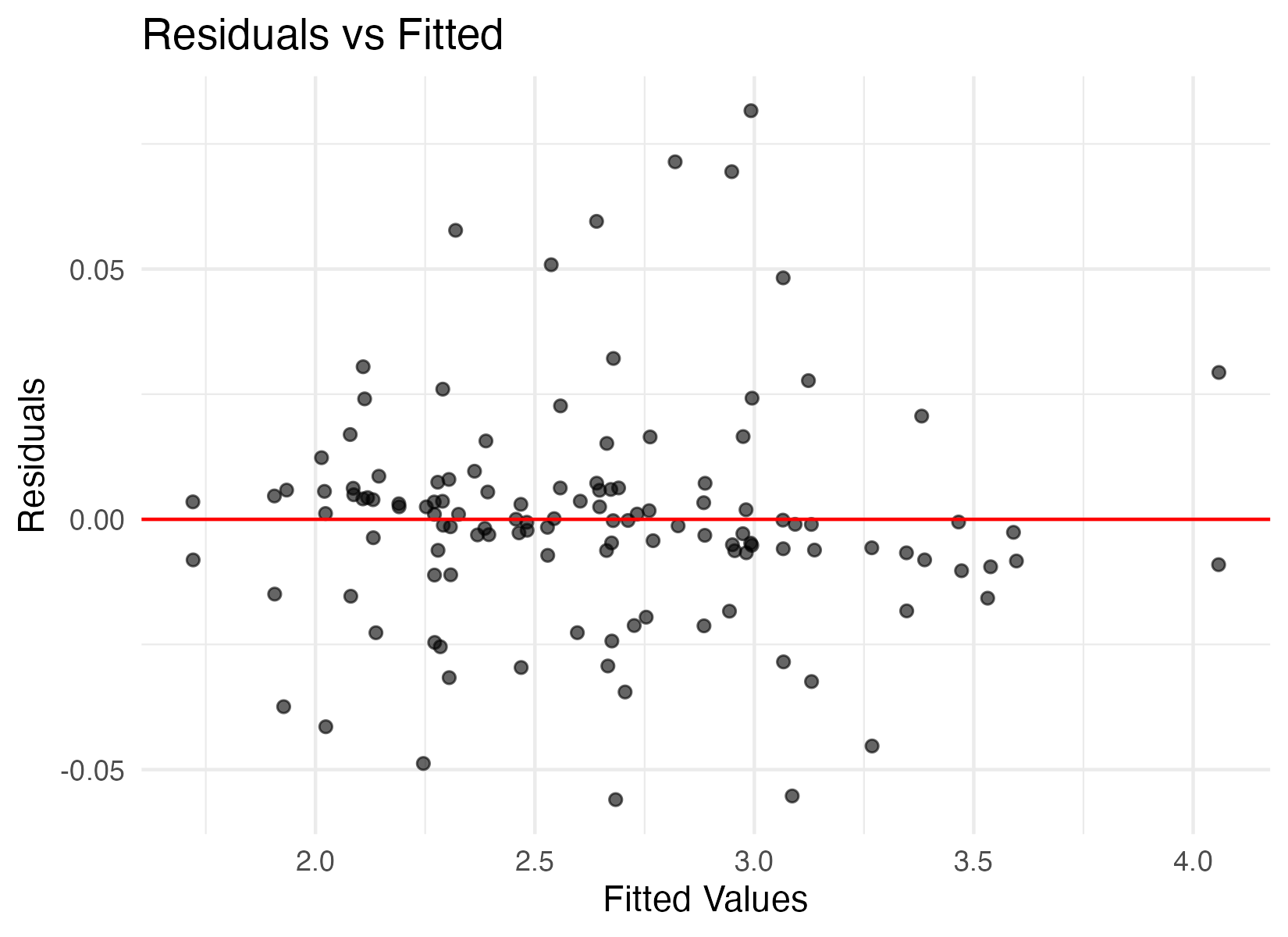


## QQ Plot


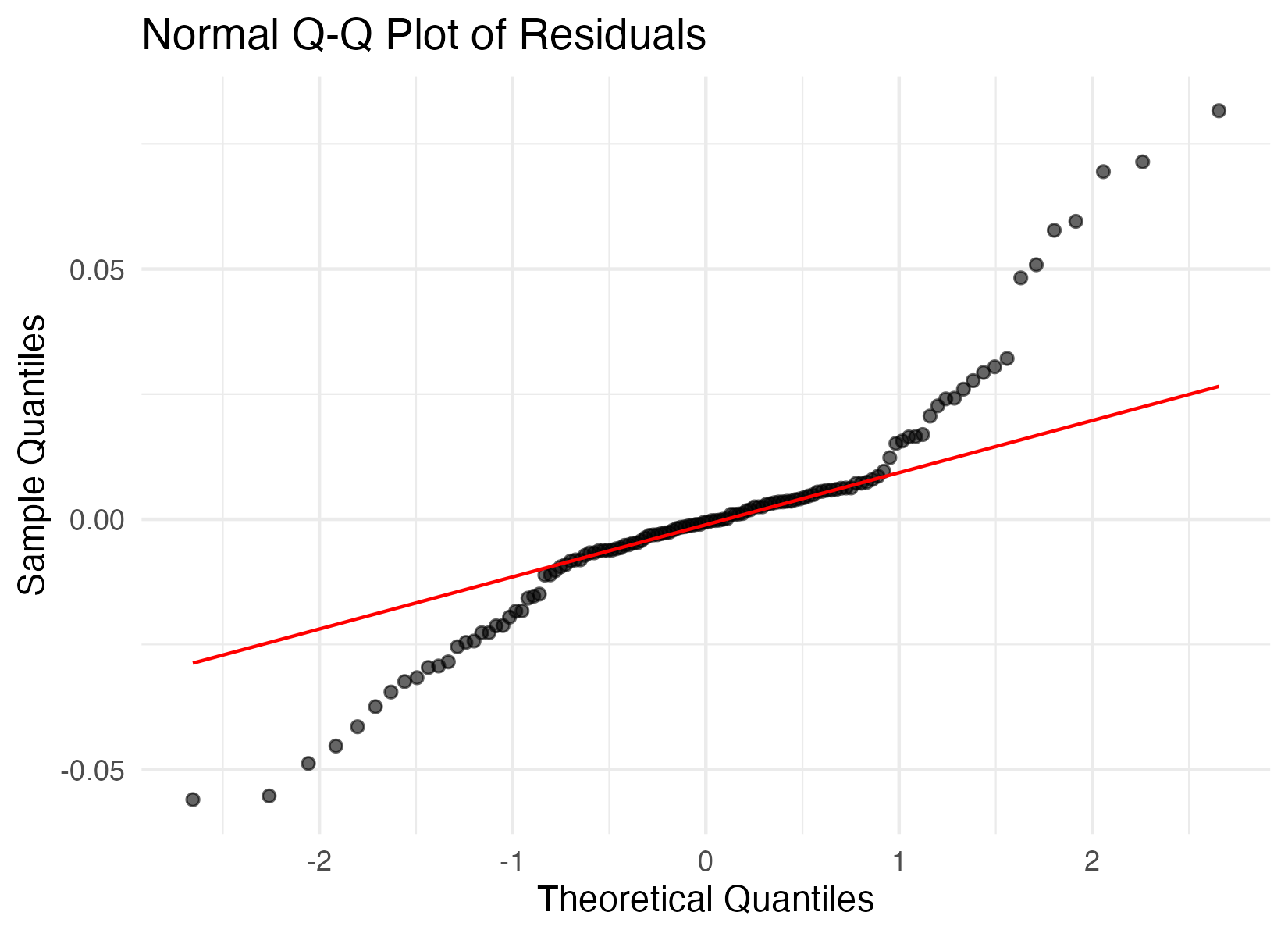


## Within-group change (baseline to follow-up)

| contrast | group | estimate | SE | df | lower.CL | upper.CL | t.ratio | p.value | effect_size |
| --- | --- | --- | --- | --- | --- | --- | --- | --- | --- |
| followup - baseline | C | 0.001 | 0.006 | 115 | -0.011 | 0.012 | 0.104 | 0.918 | 0.001 |
| followup - baseline | S | -0.007 | 0.006 | 115 | -0.018 | 0.004 | -1.218 | 0.226 | -0.014 |

## Between-group difference in change (interaction)

| timepoint_revpairwise | group_revpairwise | estimate | SE | df | lower.CL | upper.CL | t.ratio | p.value | effect_size |
| --- | --- | --- | --- | --- | --- | --- | --- | --- | --- |
| followup - baseline | S - C | -0.007 | 0.008 | 115 | -0.023 | 0.009 | -0.915 | 0.362 | -0.016 |

## Adjusted Means Over Time (with 95% CI)


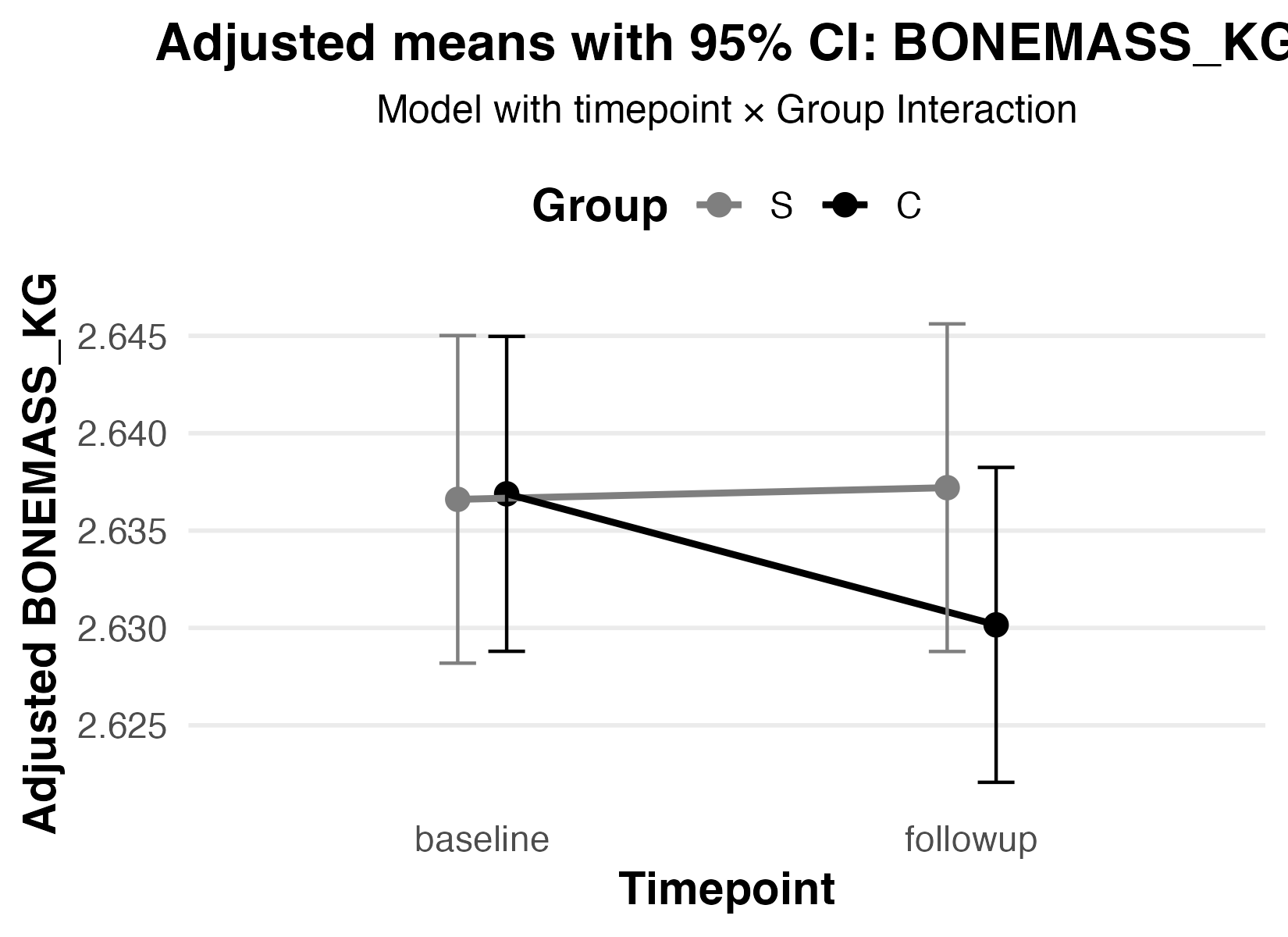


# Outcome: fatmass_kg

## Number of Participants Included: 63

## Distribution of DV at Baseline


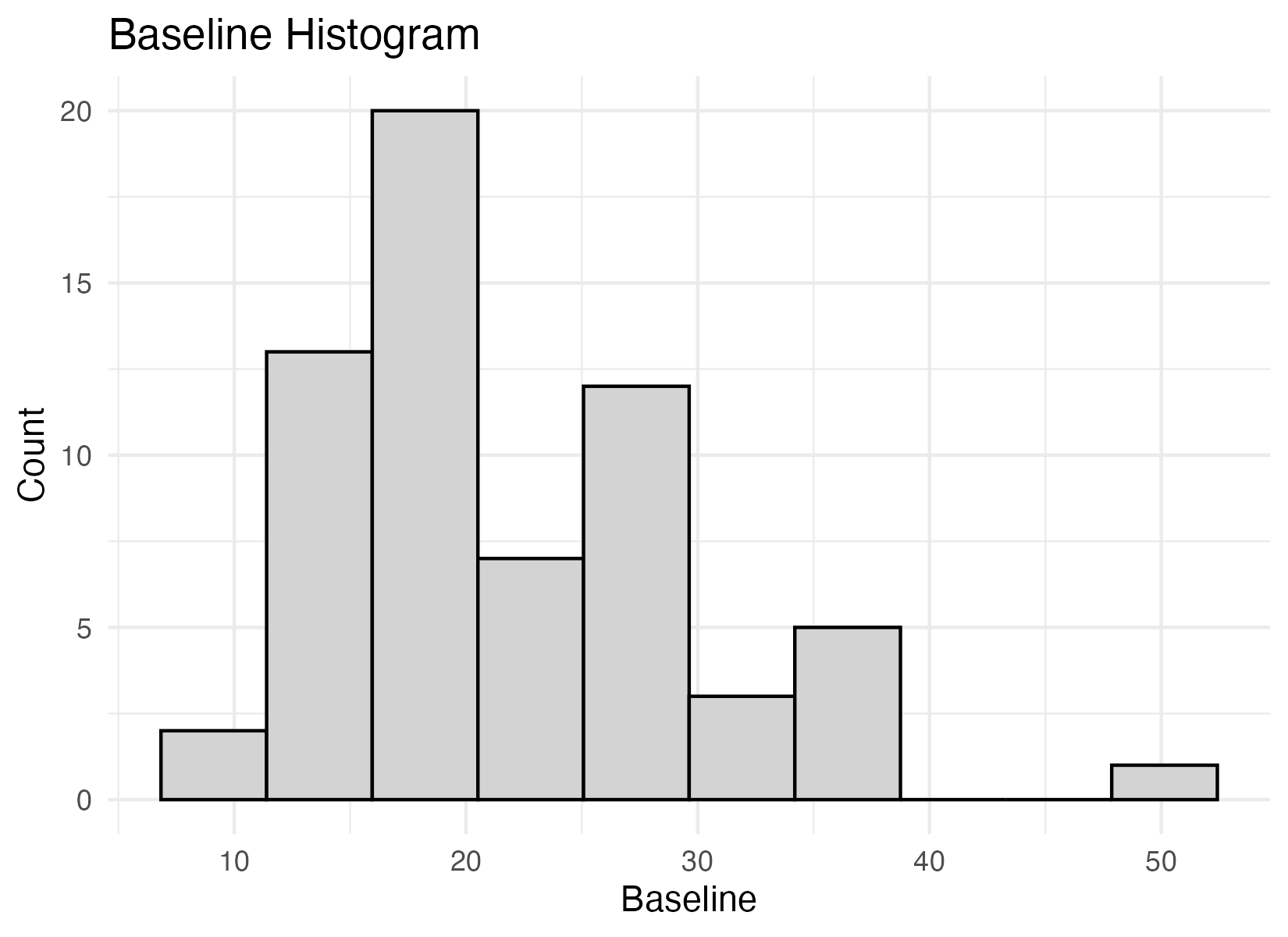


## Fitted vs Residuals


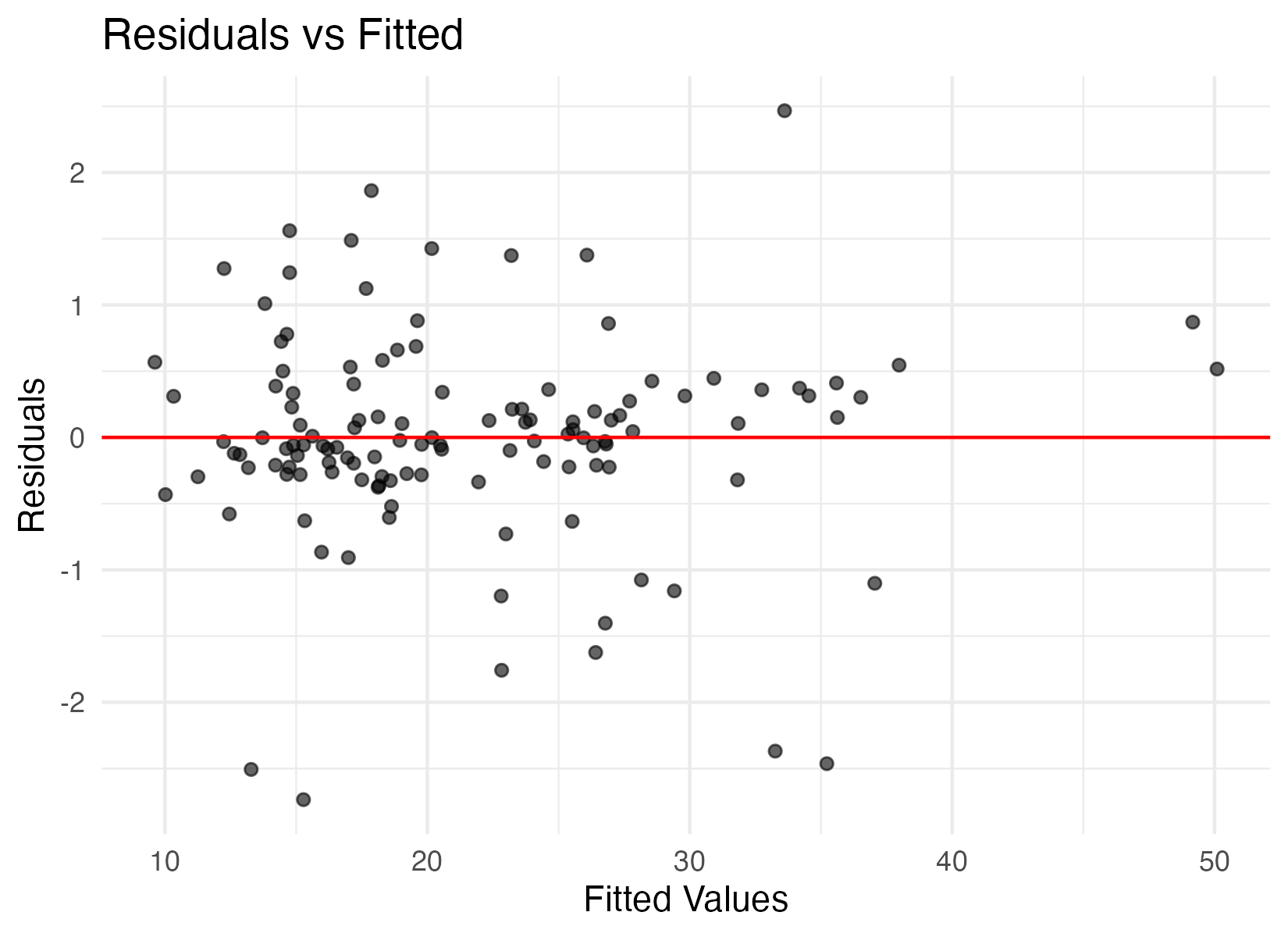


## QQ Plot


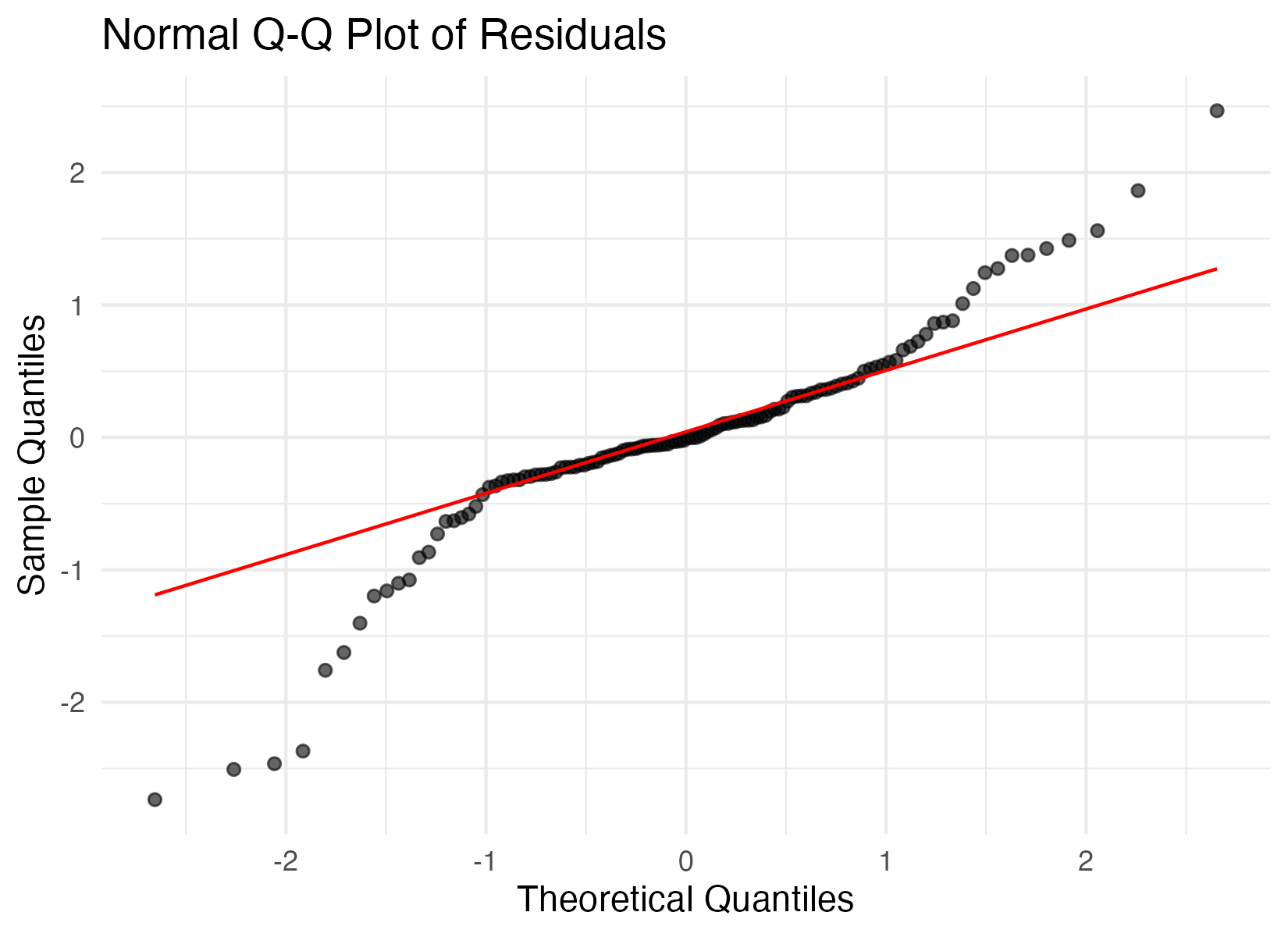


## Within-group change (baseline to follow-up)

| contrast | group | estimate | SE | df | lower.CL | upper.CL | t.ratio | p.value | effect_size |
| --- | --- | --- | --- | --- | --- | --- | --- | --- | --- |
| followup - baseline | C | -0.403 | 0.203 | 115 | -0.805 | 0.000 | -1.981 | 0.05 | -0.052 |
| followup - baseline | S | -0.927 | 0.194 | 115 | -1.310 | -0.543 | -4.782 | <0.001 | -0.121 |

## Between-group difference in change (interaction)

| timepoint_revpairwise | group_revpairwise | estimate | SE | df | lower.CL | upper.CL | t.ratio | p.value | effect_size |
| --- | --- | --- | --- | --- | --- | --- | --- | --- | --- |
| followup - baseline | S - C | -0.524 | 0.281 | 115 | -1.08 | 0.032 | -1.866 | 0.065 | -0.068 |

## Adjusted Means Over Time (with 95% CI)


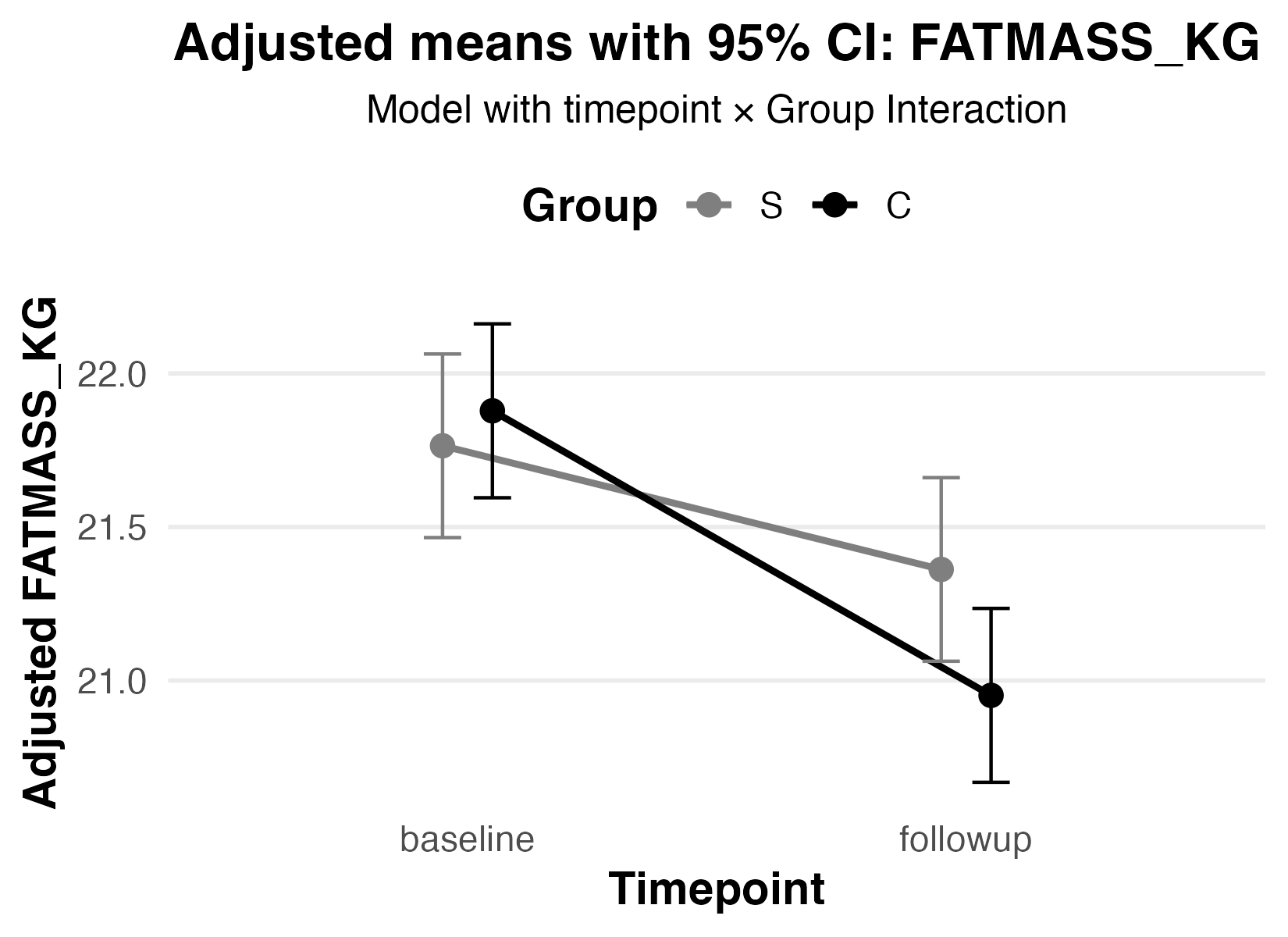


# Outcome: leanmass_kg

## Number of Participants Included: 63

## Distribution of DV at Baseline


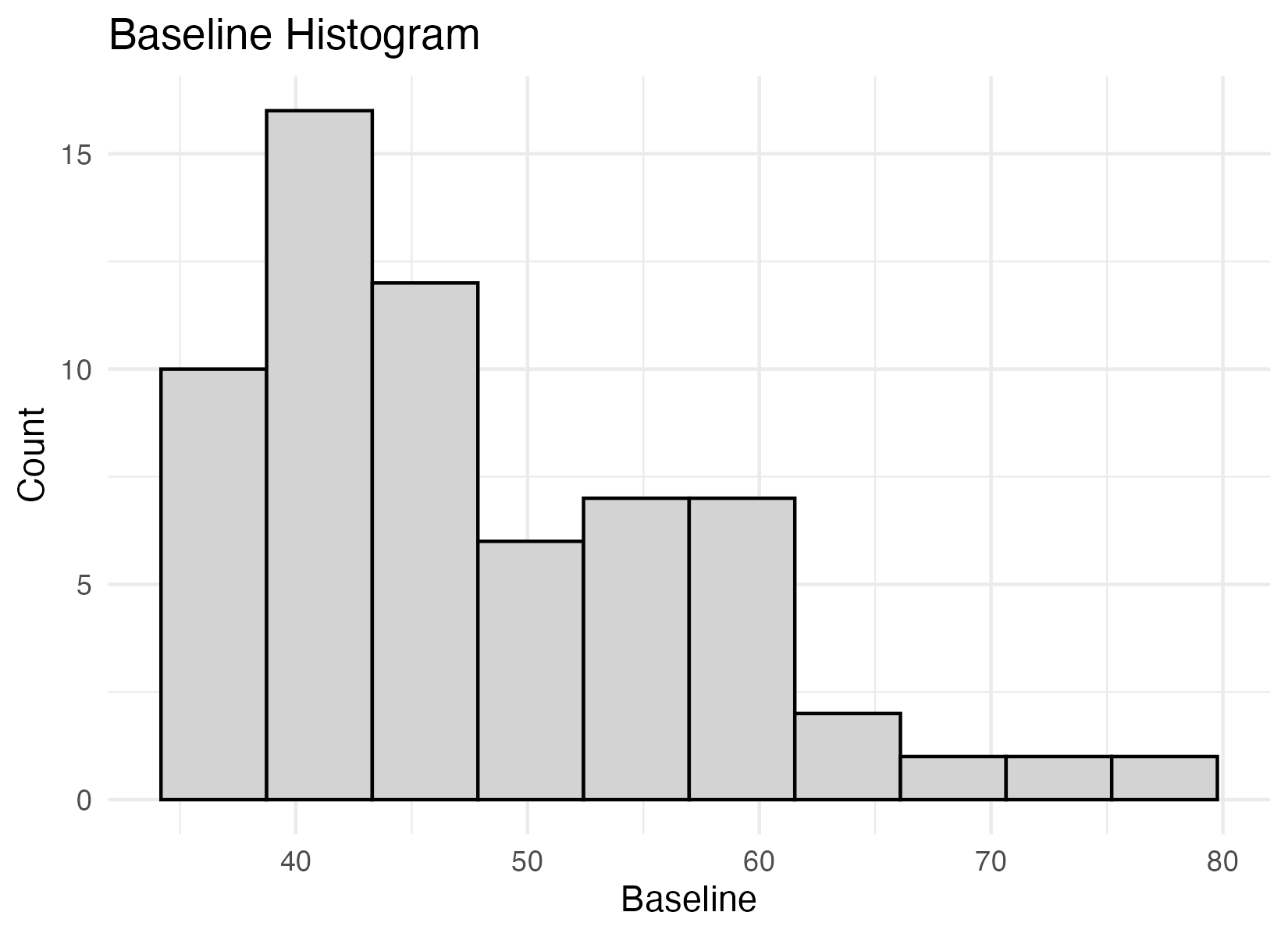


## Fitted vs Residuals


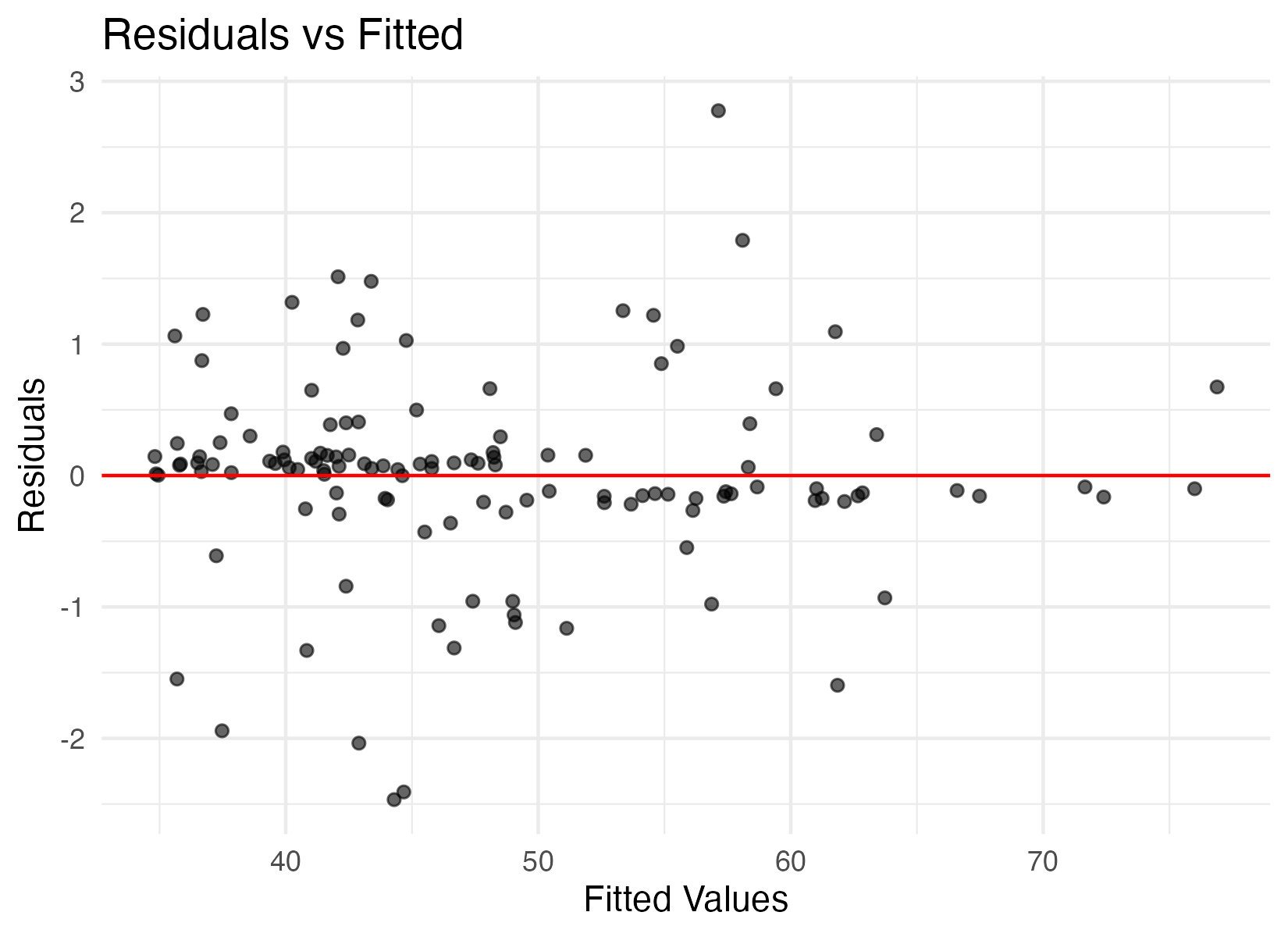


## QQ Plot


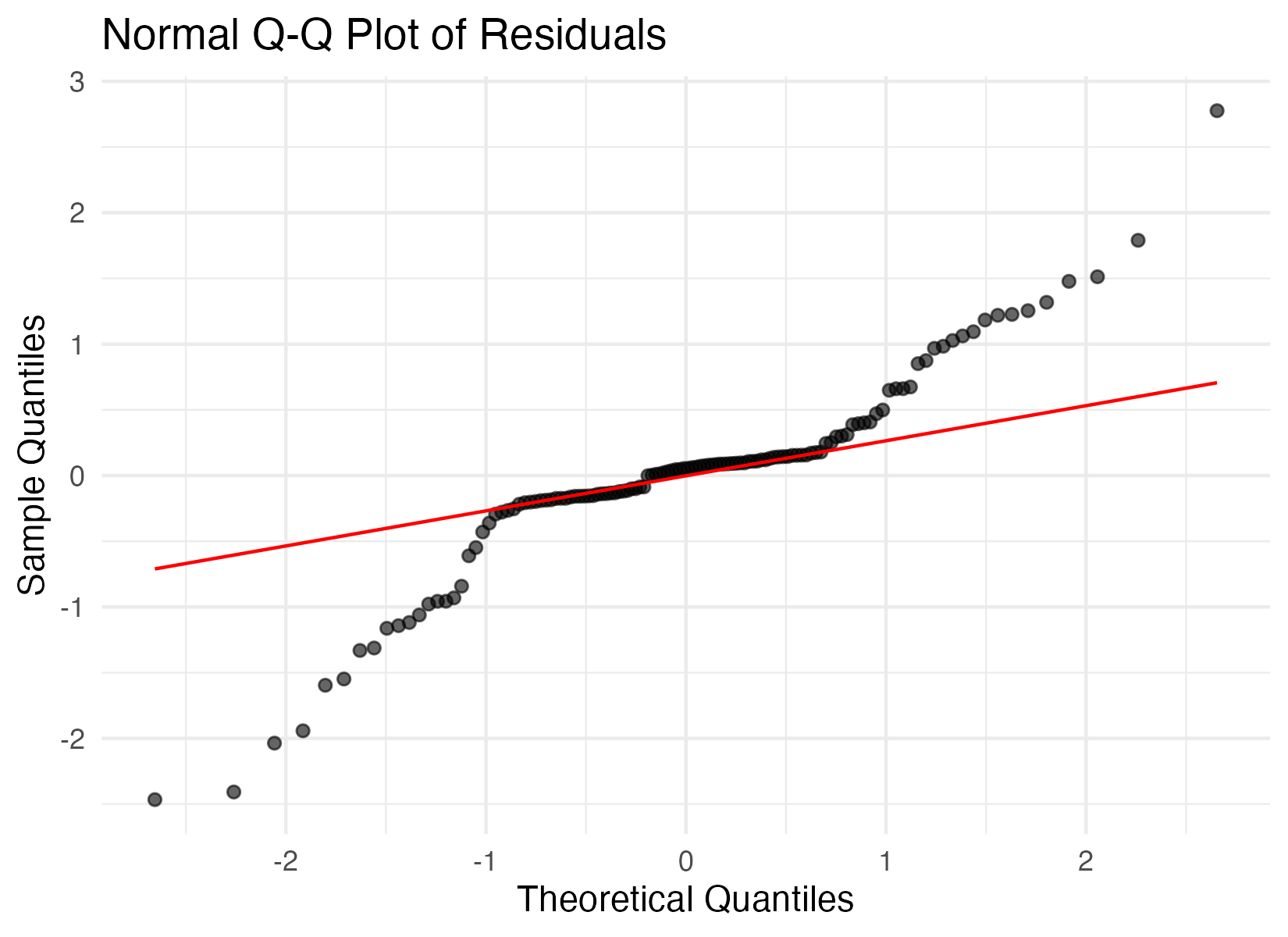


## Within-group change (baseline to follow-up)

| contrast | group | estimate | SE | df | lower.CL | upper.CL | t.ratio | p.value | effect_size |
| --- | --- | --- | --- | --- | --- | --- | --- | --- | --- |
| followup - baseline | C | 0.887 | 0.197 | 115 | 0.498 | 1.277 | 4.510 | <0.001 | 0.093 |
| followup - baseline | S | 0.743 | 0.188 | 115 | 0.371 | 1.114 | 3.958 | <0.001 | 0.078 |

## Between-group difference in change (interaction)

| timepoint_revpairwise | group_revpairwise | estimate | SE | df | lower.CL | upper.CL | t.ratio | p.value | effect_size |
| --- | --- | --- | --- | --- | --- | --- | --- | --- | --- |
| followup - baseline | S - C | -0.145 | 0.272 | 115 | -0.683 | 0.394 | -0.532 | 0.595 | -0.015 |

## Adjusted Means Over Time (with 95% CI)


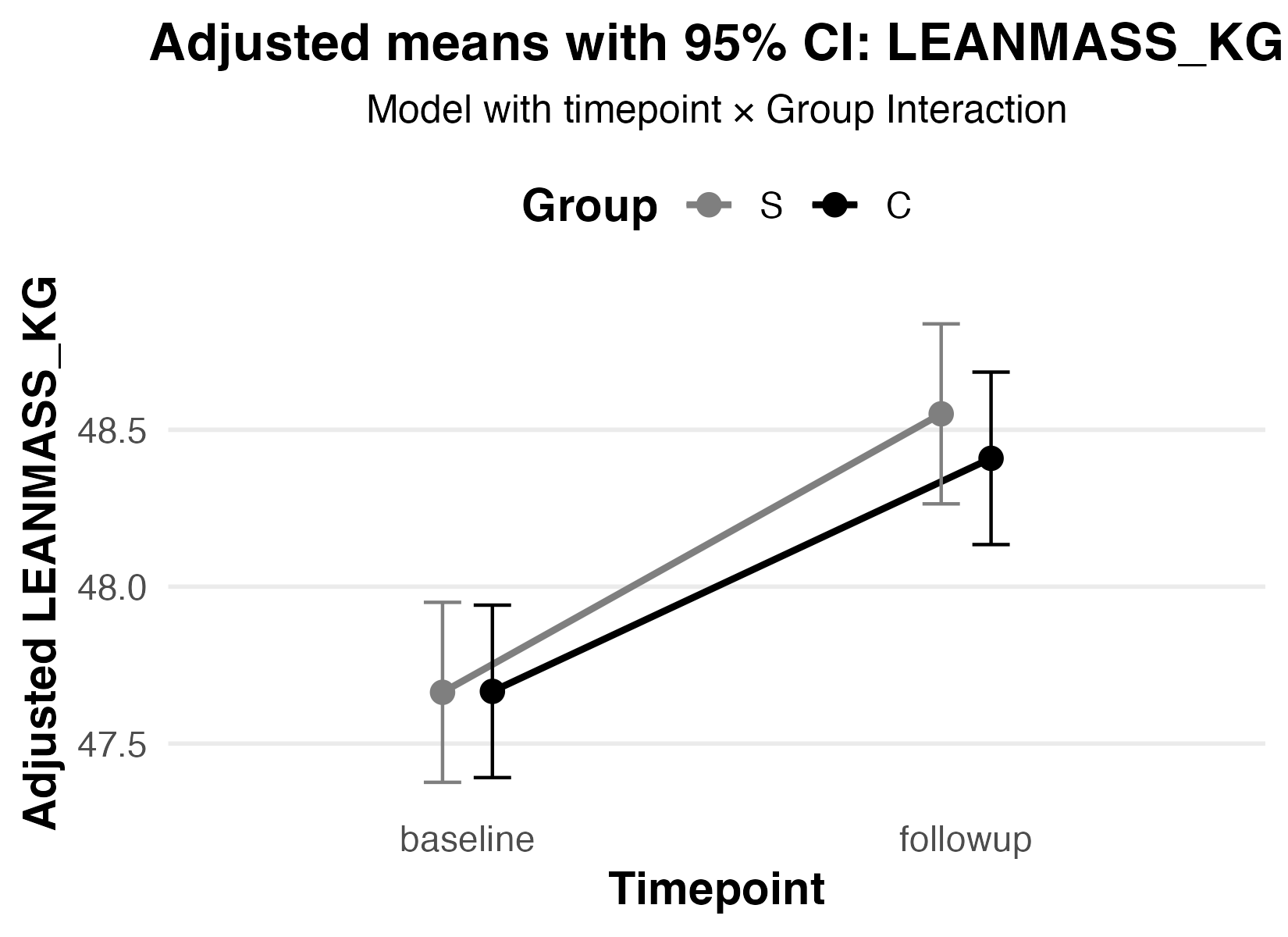


# Outcome: fatmass_percent

## Number of Participants Included: 63

## Distribution of DV at Baseline


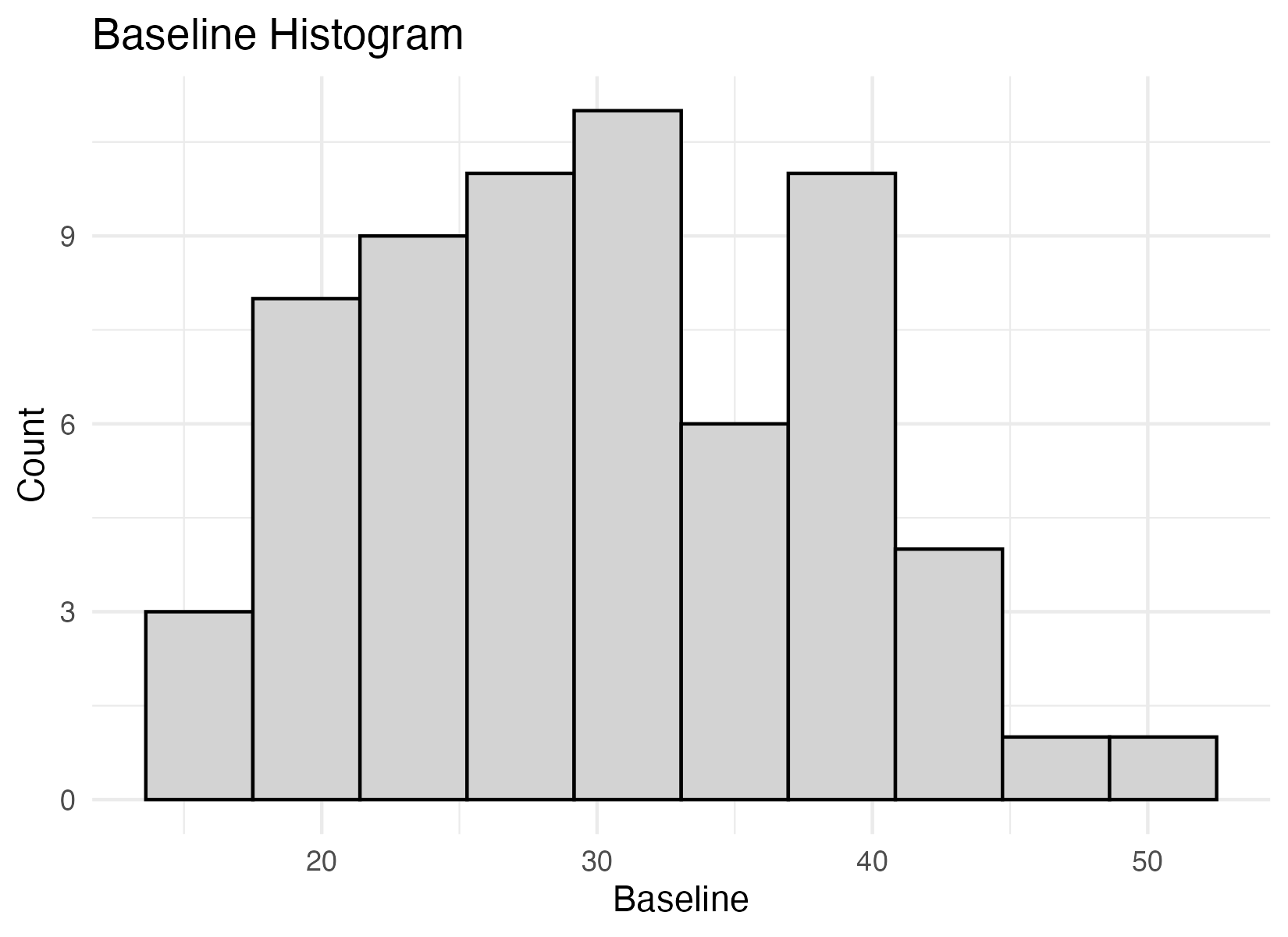


## Fitted vs Residuals


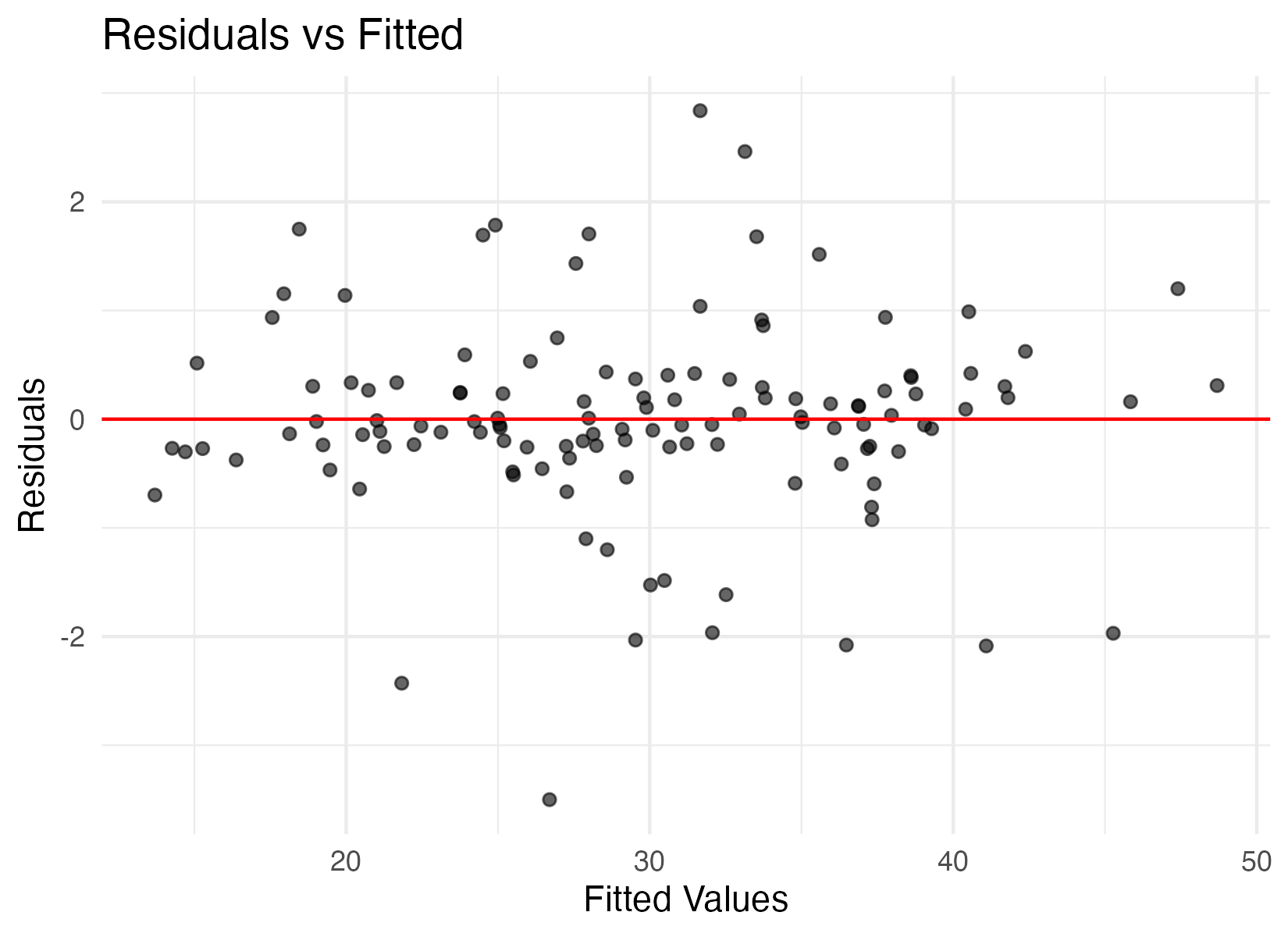


## QQ Plot


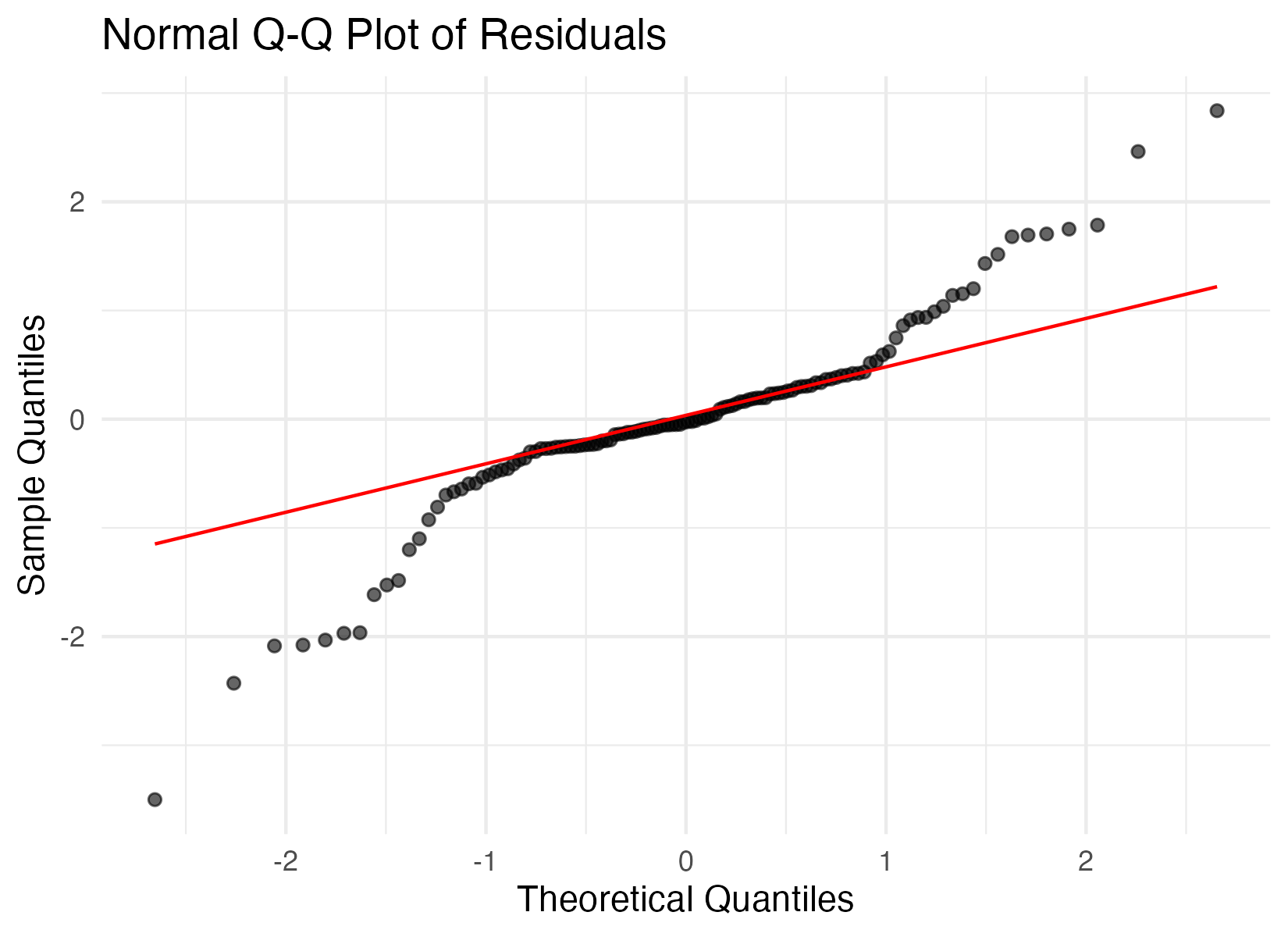


## Within-group change (baseline to follow-up)

| contrast | group | estimate | SE | df | lower.CL | upper.CL | t.ratio | p.value | effect_size |
| --- | --- | --- | --- | --- | --- | --- | --- | --- | --- |
| followup - baseline | C | -0.570 | 0.232 | 115 | -1.03 | -0.110 | -2.453 | 0.016 | -0.074 |
| followup - baseline | S | -1.291 | 0.222 | 115 | -1.73 | -0.852 | -5.825 | <0.001 | -0.167 |

## Between-group difference in change (interaction)

| timepoint_revpairwise | group_revpairwise | estimate | SE | df | lower.CL | upper.CL | t.ratio | p.value | effect_size |
| --- | --- | --- | --- | --- | --- | --- | --- | --- | --- |
| followup - baseline | S - C | -0.721 | 0.321 | 115 | -1.357 | -0.085 | -2.245 | 0.027 | -0.093 |

## Adjusted Means Over Time (with 95% CI)


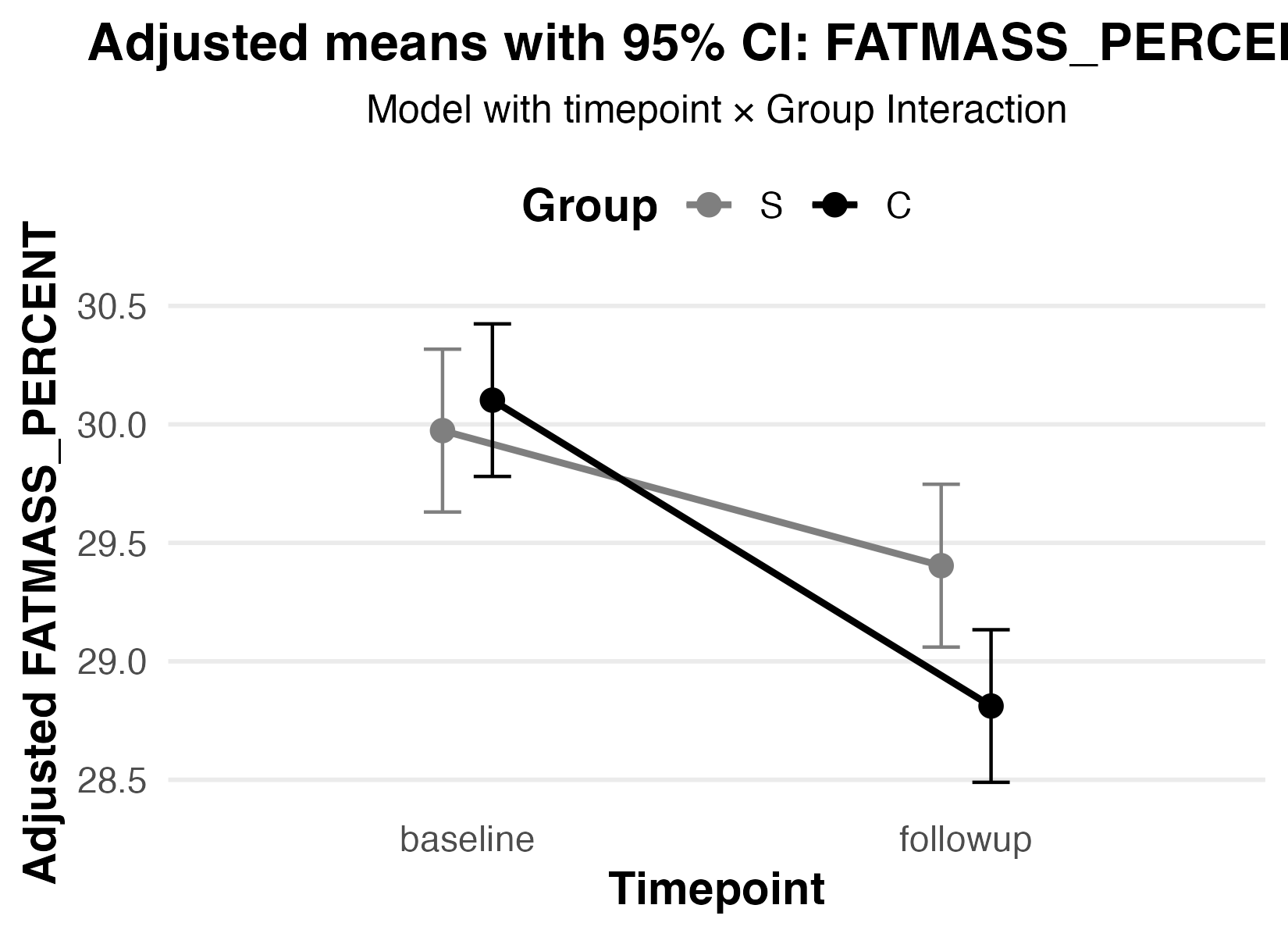


# Outcome: leanmass_percent

## Number of Participants Included: 63

## Distribution of DV at Baseline


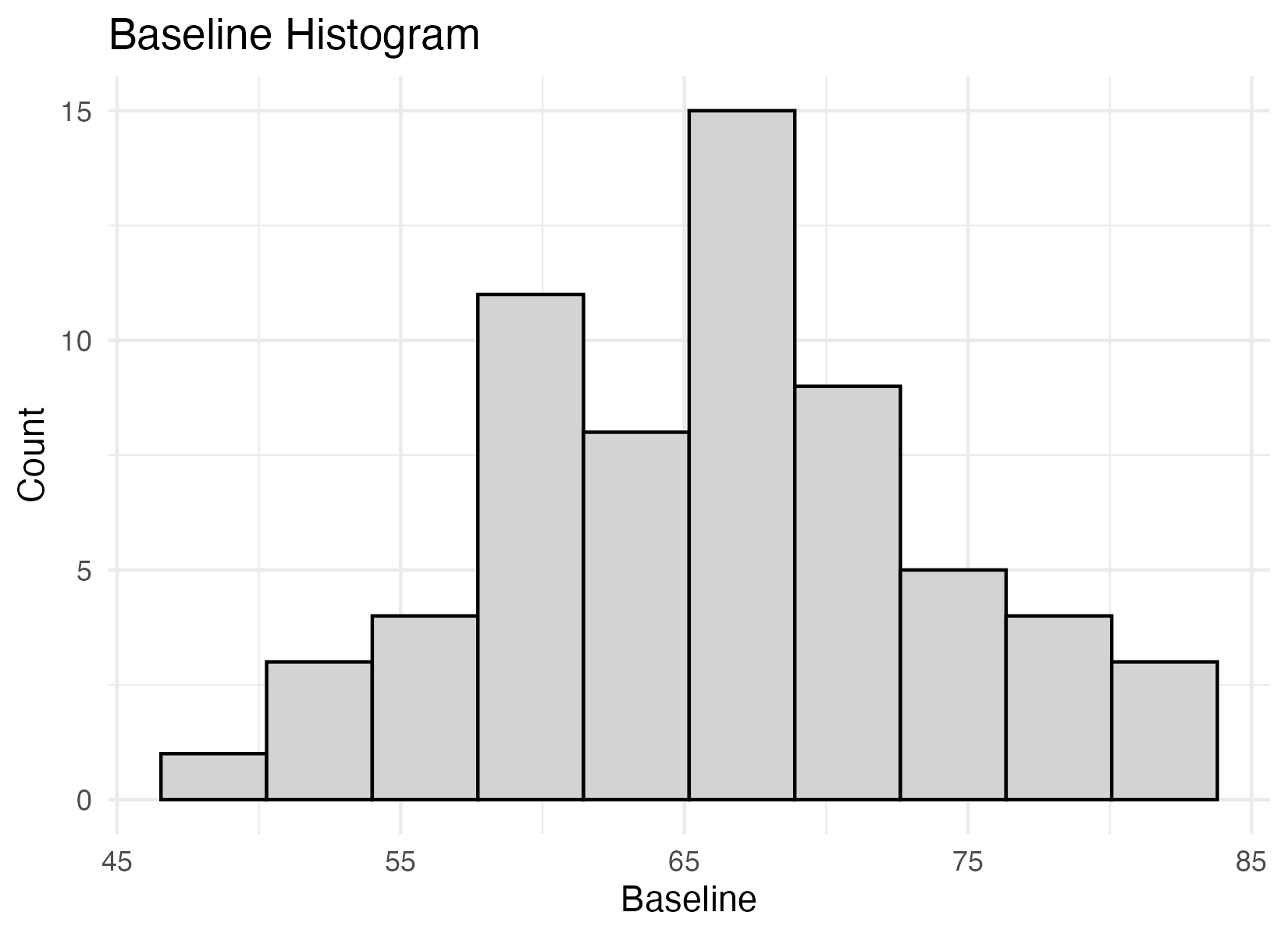


## Fitted vs Residuals


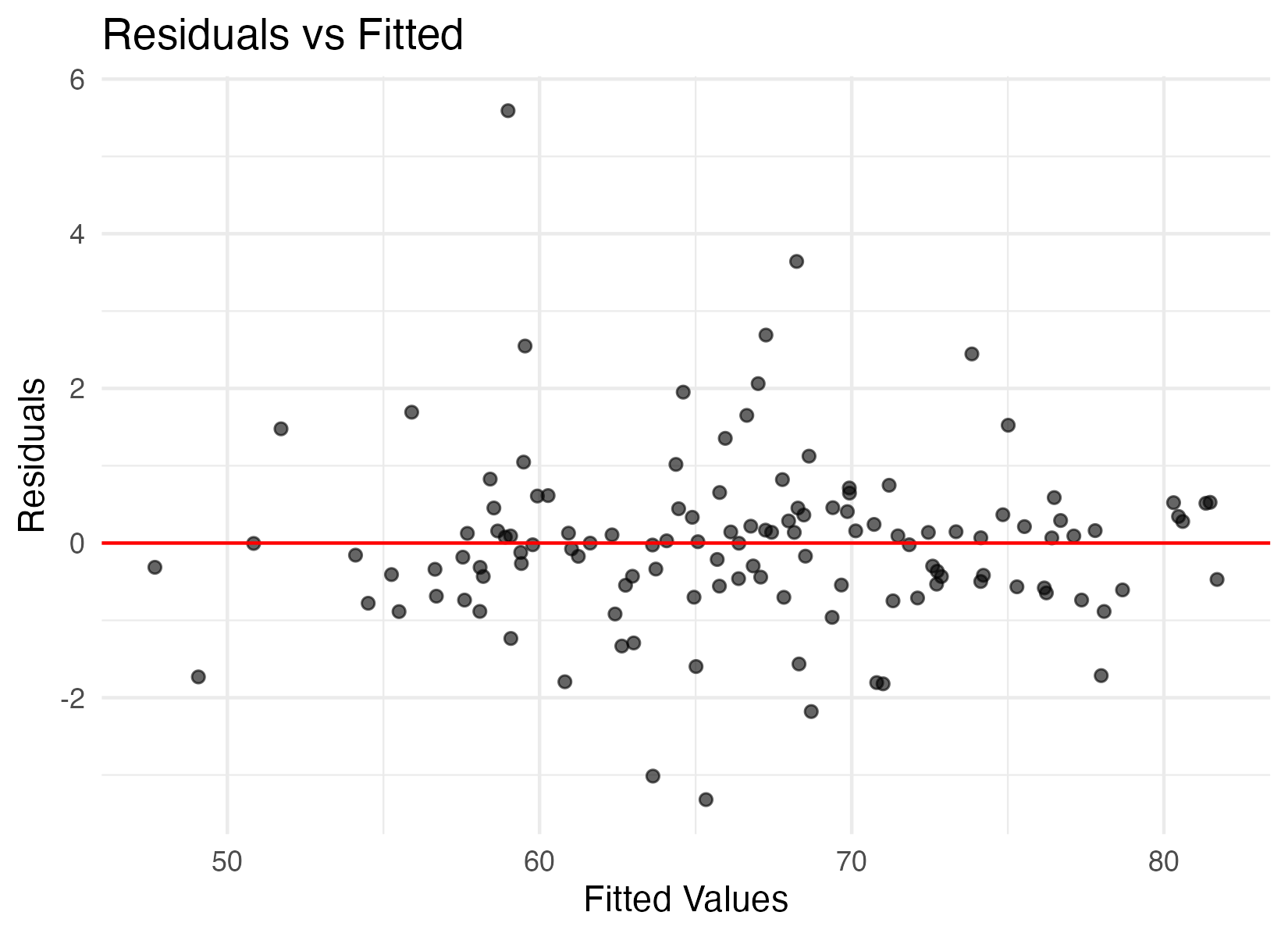


## QQ Plot


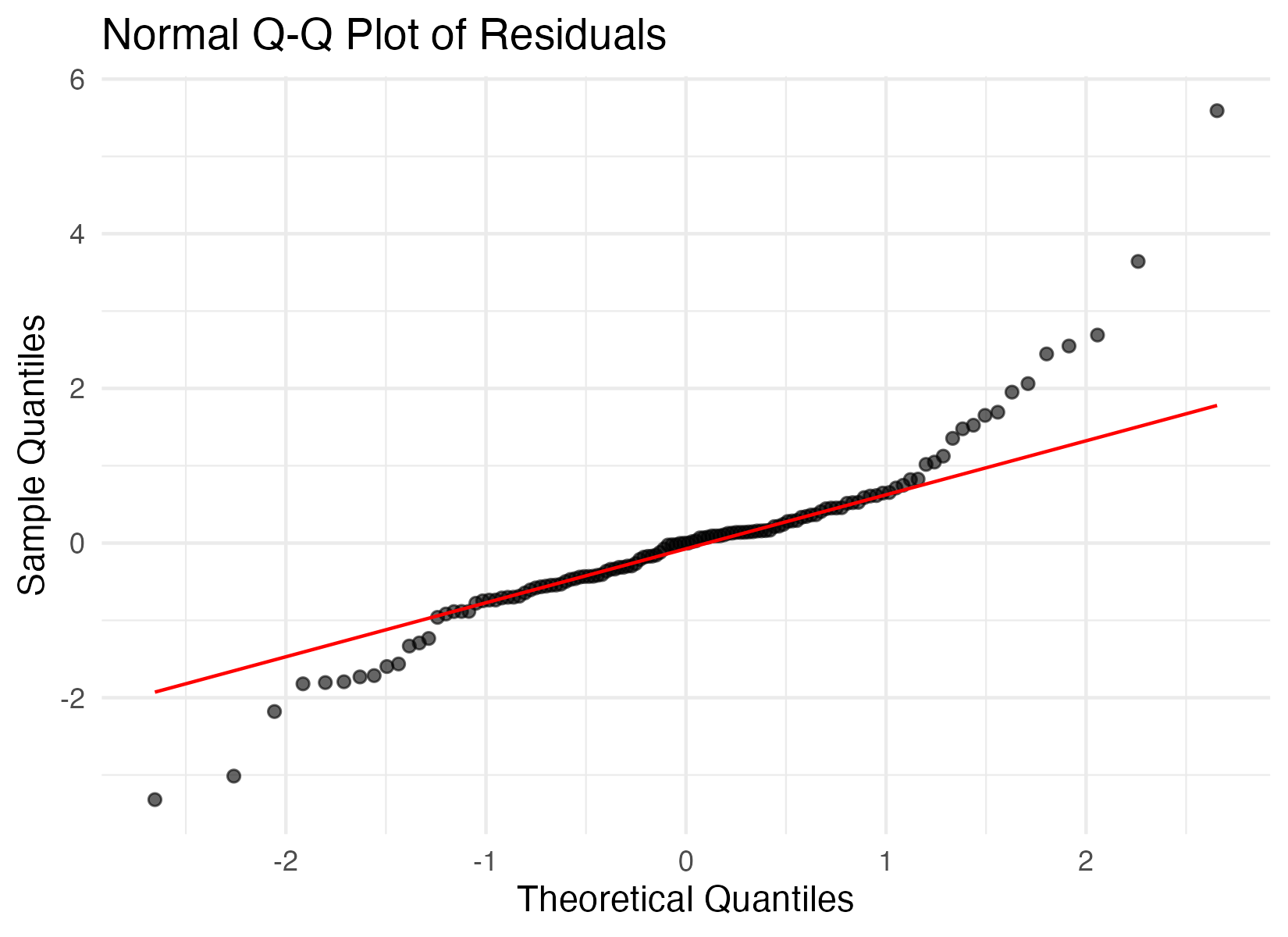


## Within-group change (baseline to follow-up)

| contrast | group | estimate | SE | df | lower.CL | upper.CL | t.ratio | p.value | effect_size |
| --- | --- | --- | --- | --- | --- | --- | --- | --- | --- |
| followup - baseline | C | 0.877 | 0.290 | 115 | 0.302 | 1.452 | 3.021 | 0.003 | 0.116 |
| followup - baseline | S | 1.393 | 0.277 | 115 | 0.845 | 1.941 | 5.035 | <0.001 | 0.184 |

## Between-group difference in change (interaction)

| timepoint_revpairwise | group_revpairwise | estimate | SE | df | lower.CL | upper.CL | t.ratio | p.value | effect_size |
| --- | --- | --- | --- | --- | --- | --- | --- | --- | --- |
| followup - baseline | S - C | 0.517 | 0.401 | 115 | -0.278 | 1.311 | 1.288 | 0.2 | 0.068 |

## Adjusted Means Over Time (with 95% CI)


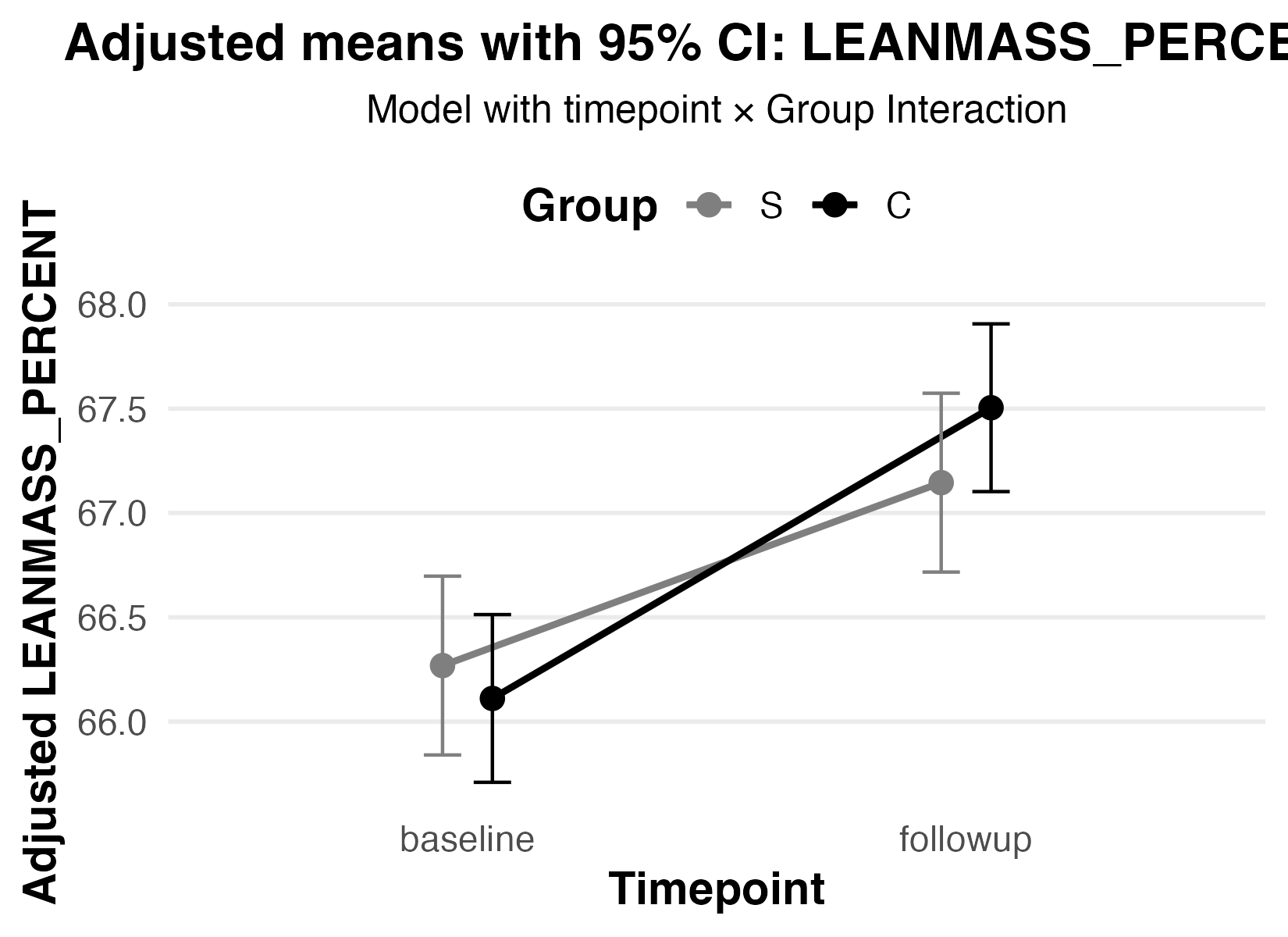


# Outcome: vo2peak_ml_kg_min

## Number of Participants Included: 63

## Distribution of DV at Baseline


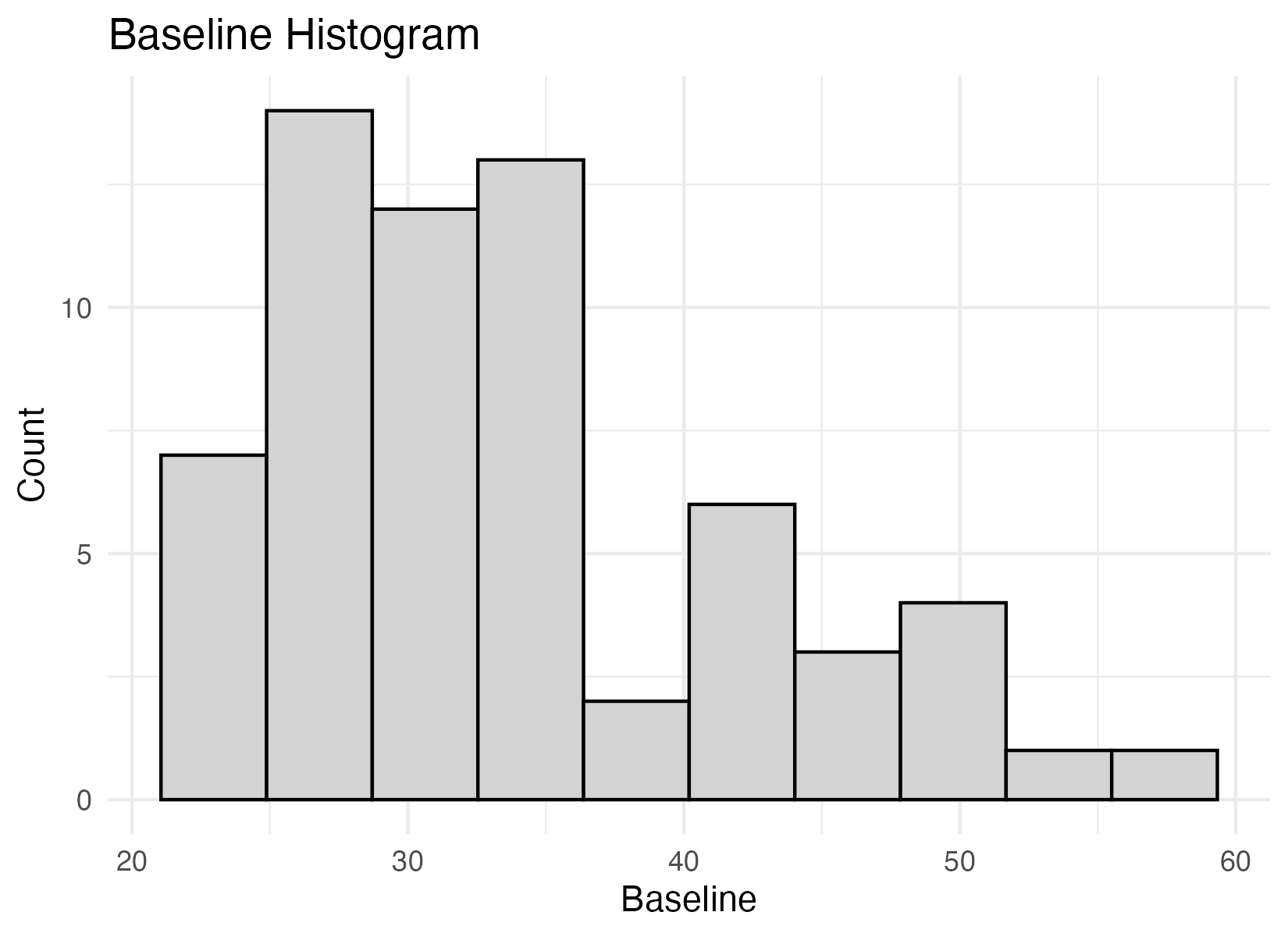


## Fitted vs Residuals


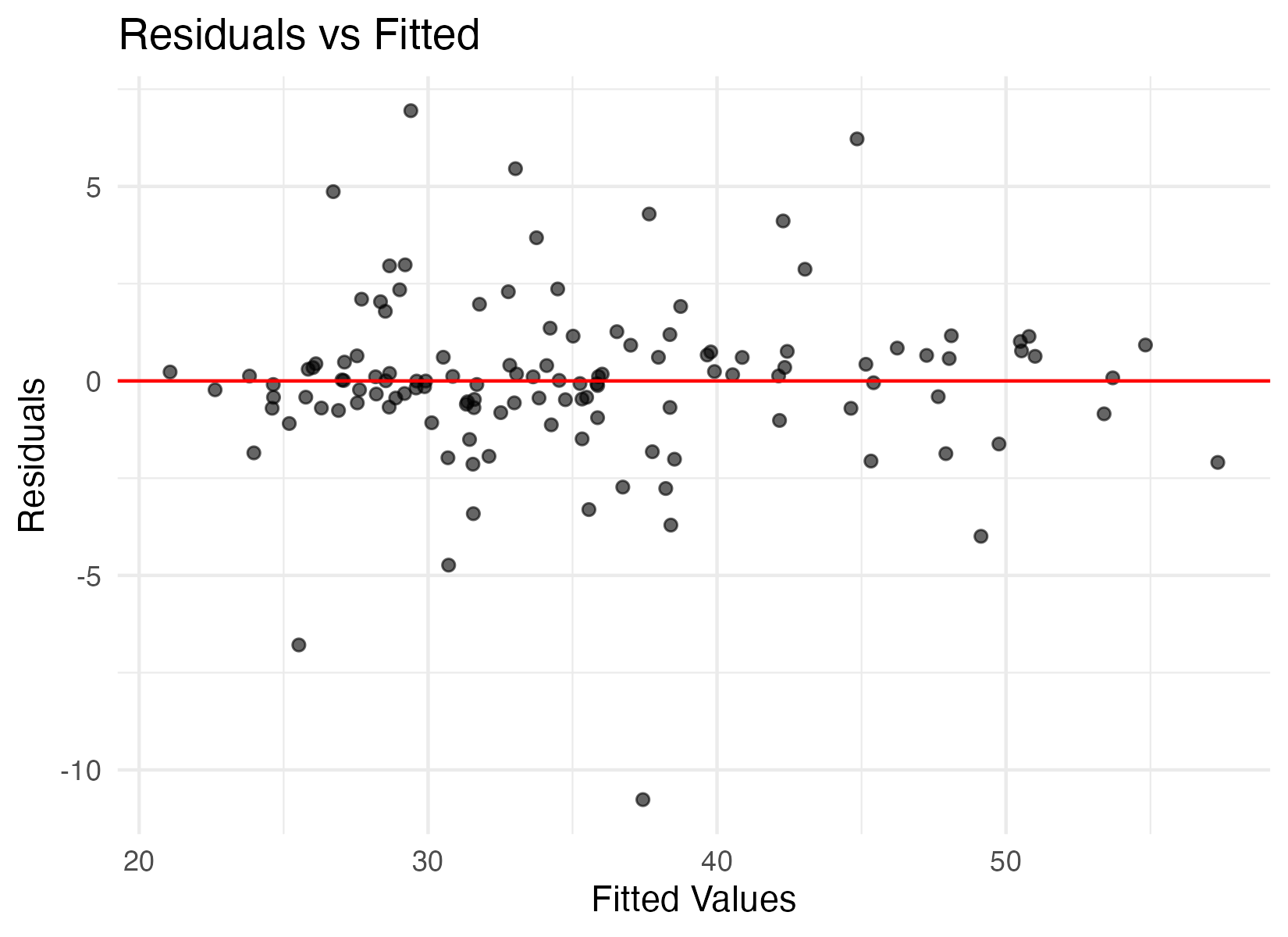


## QQ Plot


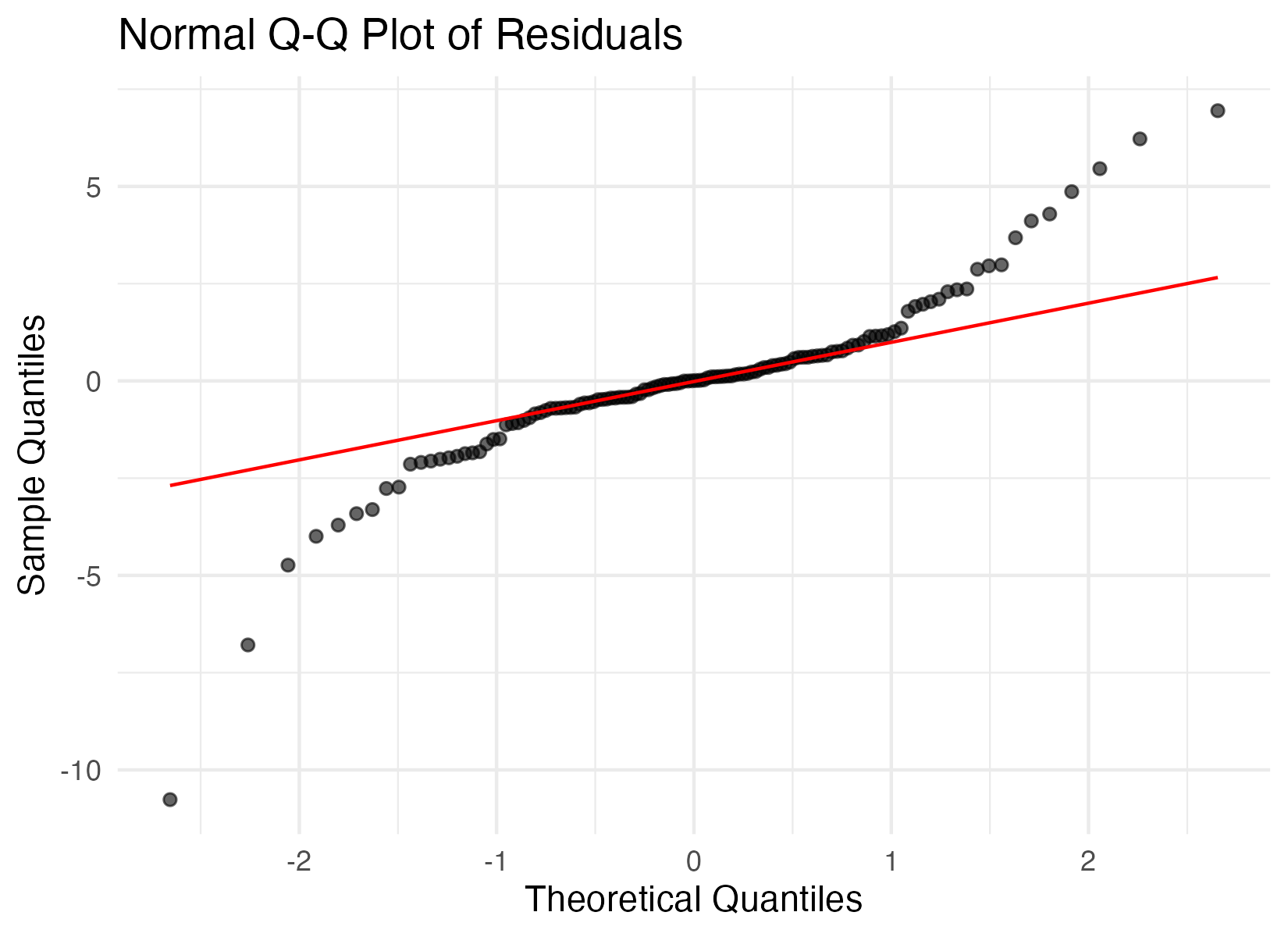


## Within-group change (baseline to follow-up)

| contrast | group | estimate | SE | df | lower.CL | upper.CL | t.ratio | p.value | effect_size |
| --- | --- | --- | --- | --- | --- | --- | --- | --- | --- |
| followup - baseline | C | 2.503 | 0.553 | 115 | 1.408 | 3.598 | 4.529 | <0.001 | 0.303 |
| followup - baseline | S | 2.899 | 0.527 | 115 | 1.855 | 3.943 | 5.500 | <0.001 | 0.351 |

## Between-group difference in change (interaction)

| timepoint_revpairwise | group_revpairwise | estimate | SE | df | lower.CL | upper.CL | t.ratio | p.value | effect_size |
| --- | --- | --- | --- | --- | --- | --- | --- | --- | --- |
| followup - baseline | S - C | 0.396 | 0.764 | 115 | -1.117 | 1.908 | 0.518 | 0.605 | 0.048 |

## Adjusted Means Over Time (with 95% CI)


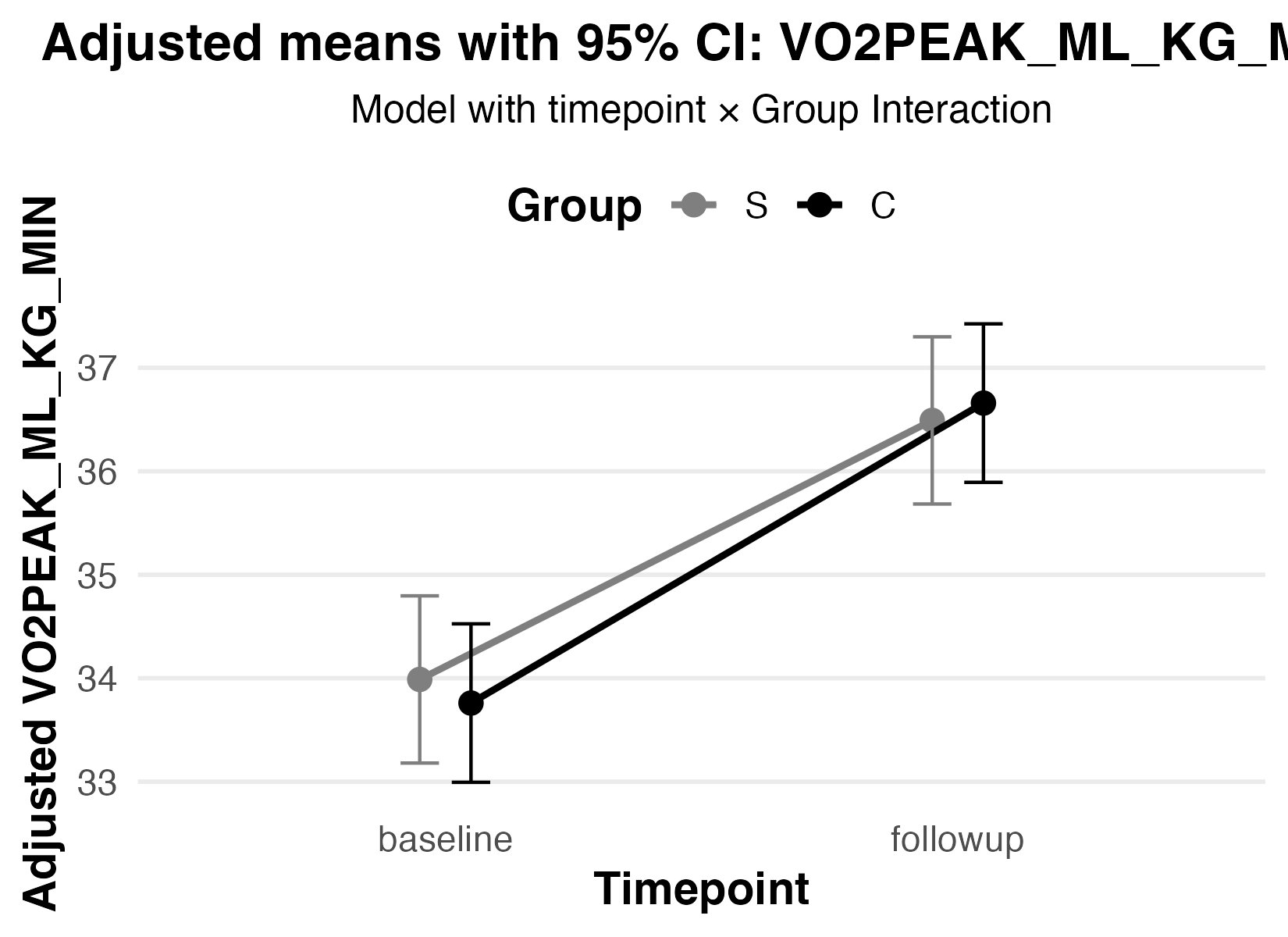


# Outcome: vo2_l_min

## Number of Participants Included: 63

## Distribution of DV at Baseline


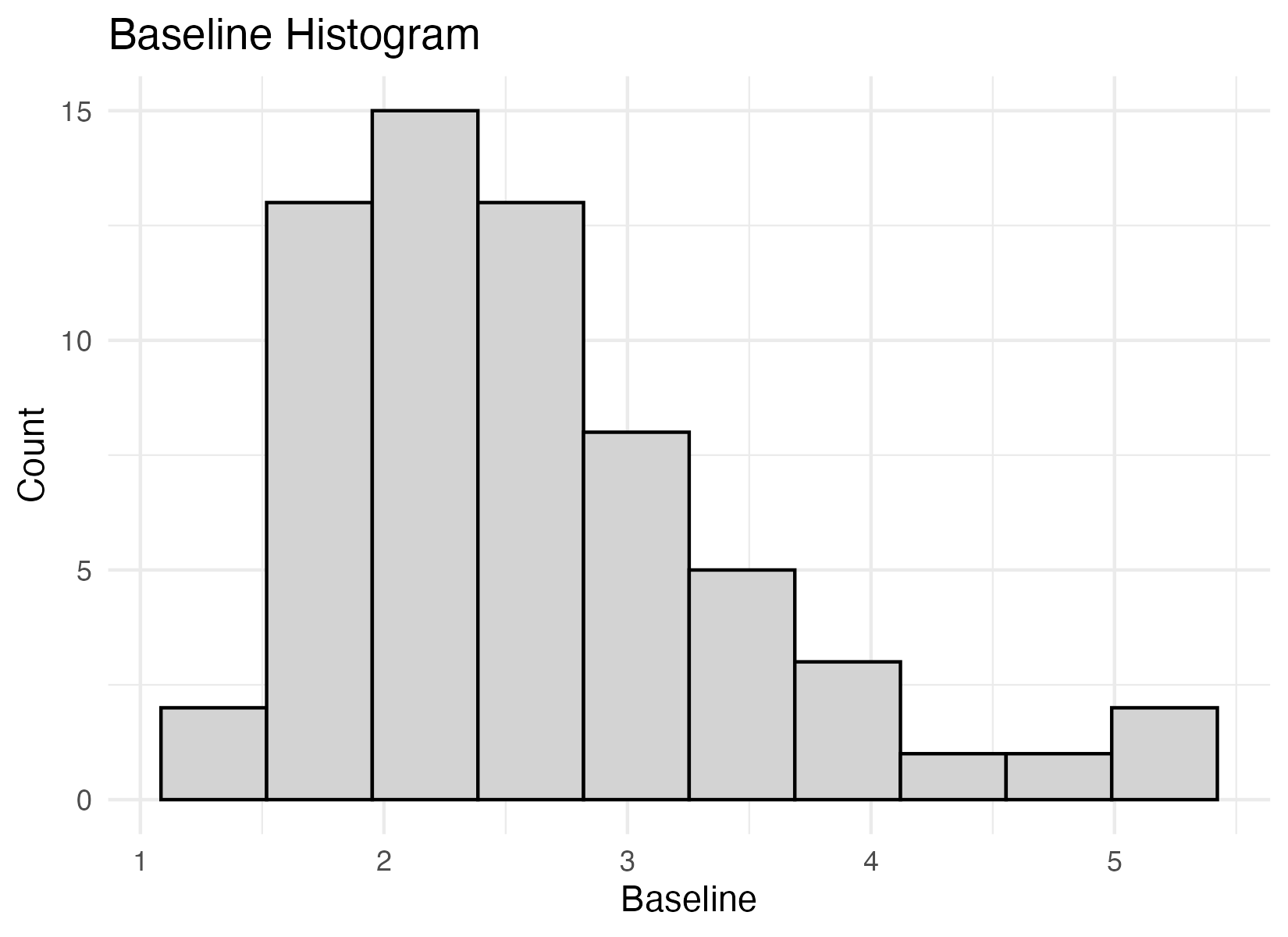


## Fitted vs Residuals


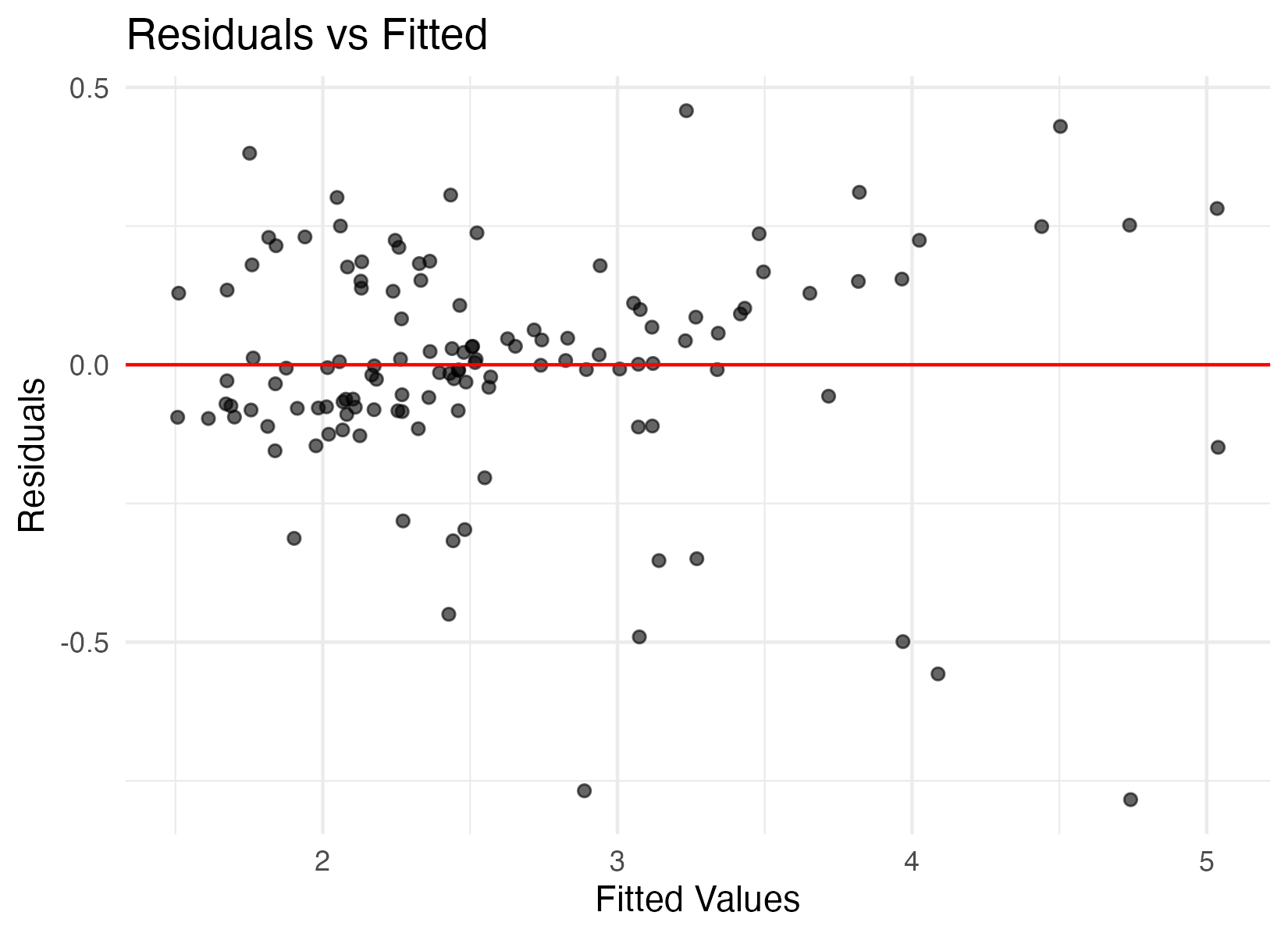


## QQ Plot


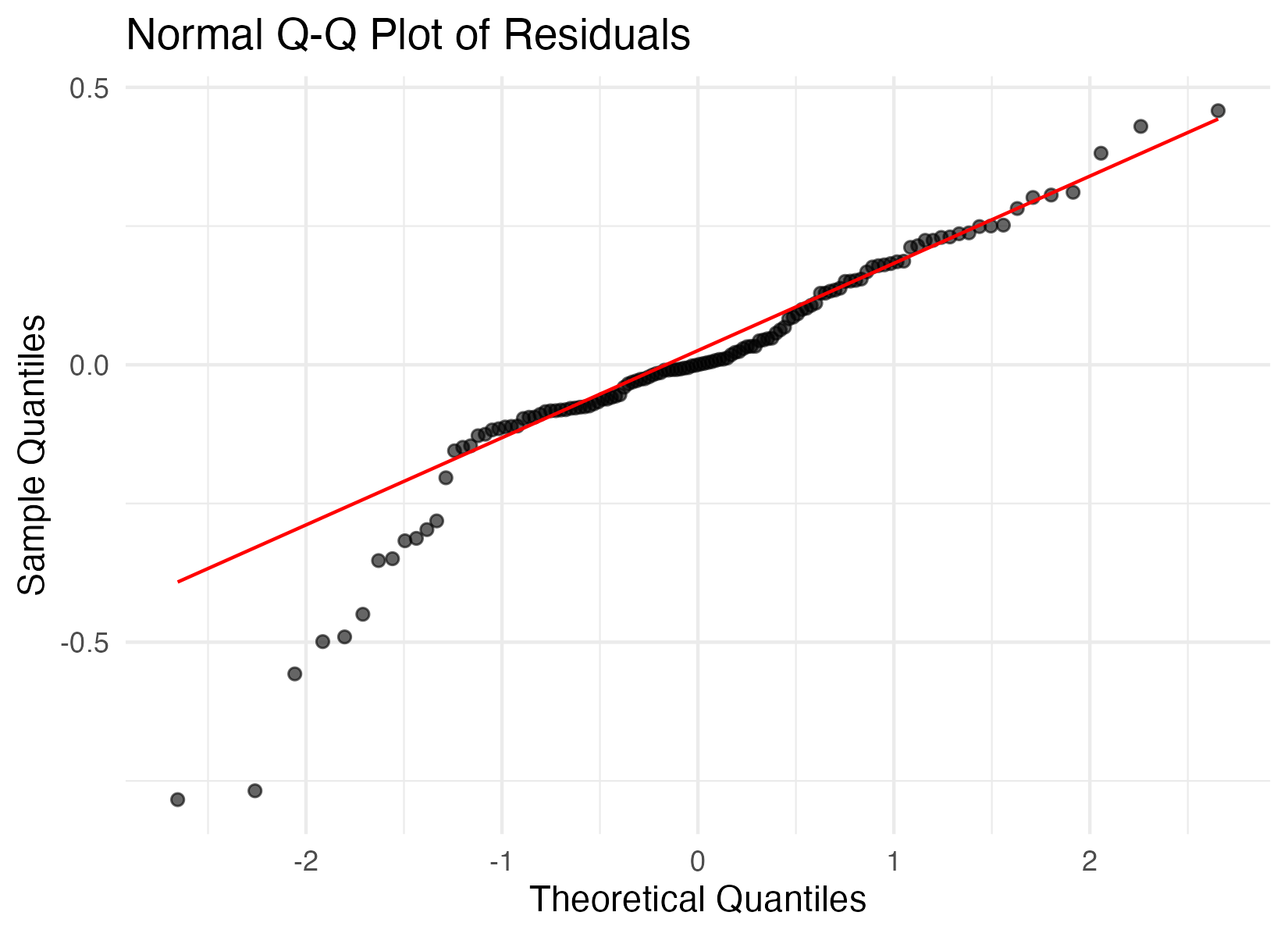


## Within-group change (baseline to follow-up)

| contrast | group | estimate | SE | df | lower.CL | upper.CL | t.ratio | p.value | effect_size |
| --- | --- | --- | --- | --- | --- | --- | --- | --- | --- |
| followup - baseline | C | 0.003 | 0.052 | 115 | -0.100 | 0.107 | 0.065 | 0.948 | 0.004 |
| followup - baseline | S | 0.064 | 0.050 | 115 | -0.035 | 0.162 | 1.276 | 0.205 | 0.080 |

## Between-group difference in change (interaction)

| timepoint_revpairwise | group_revpairwise | estimate | SE | df | lower.CL | upper.CL | t.ratio | p.value | effect_size |
| --- | --- | --- | --- | --- | --- | --- | --- | --- | --- |
| followup - baseline | S - C | 0.06 | 0.072 | 115 | -0.083 | 0.203 | 0.833 | 0.406 | 0.076 |

## Adjusted Means Over Time (with 95% CI)


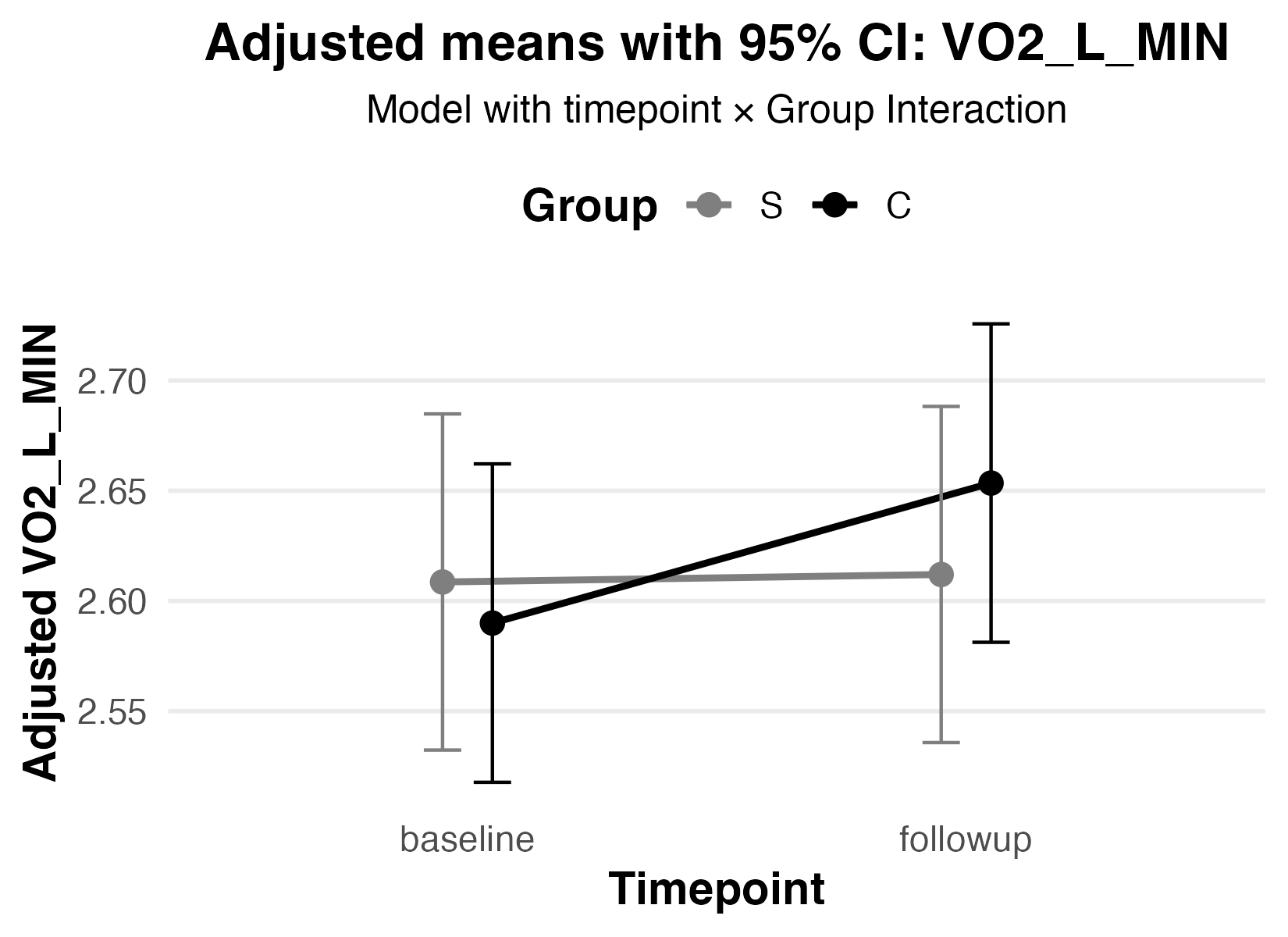


# Outcome: workmax

## Number of Participants Included: 63

## Distribution of DV at Baseline


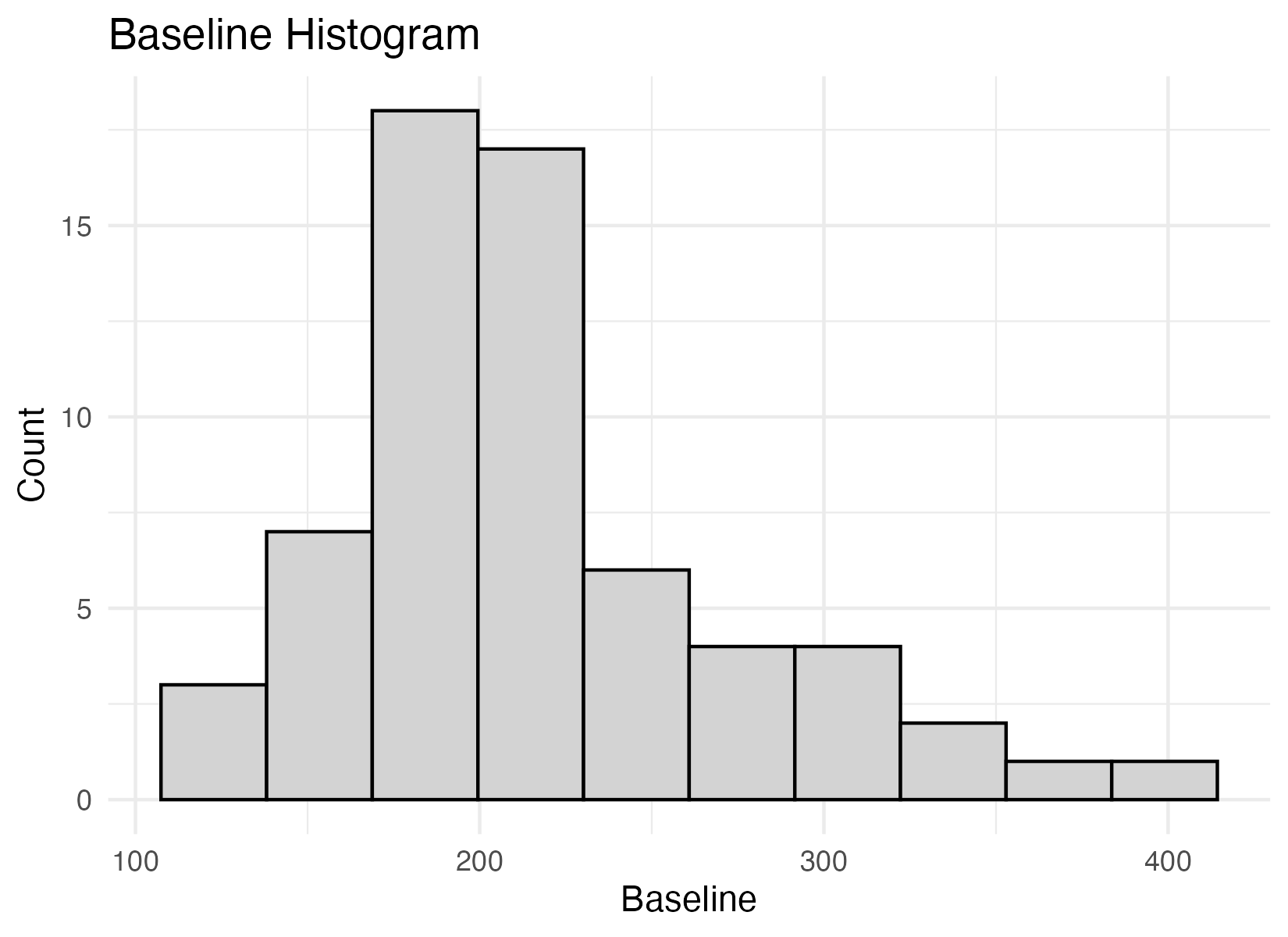


## Fitted vs Residuals


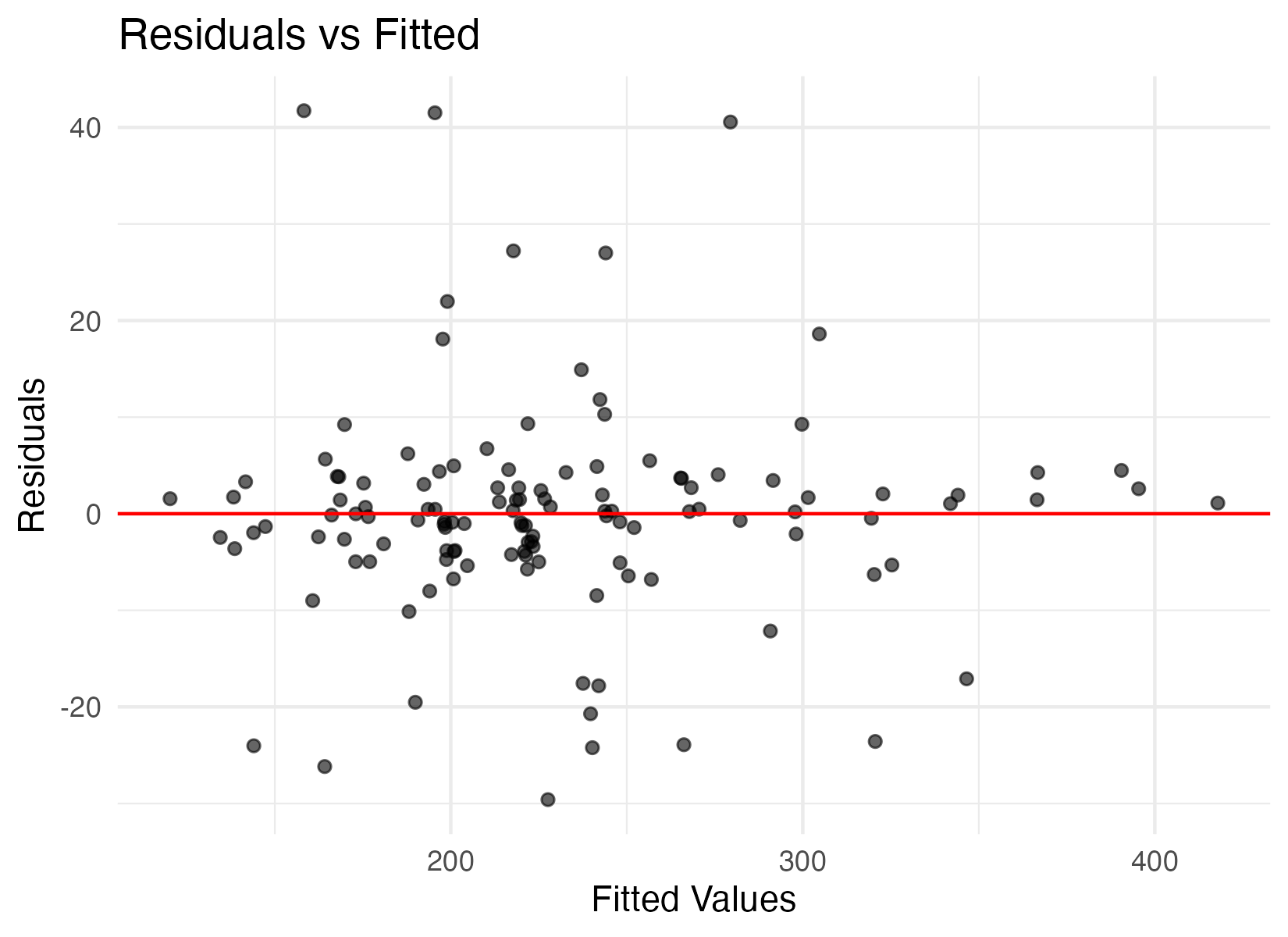


## QQ Plot


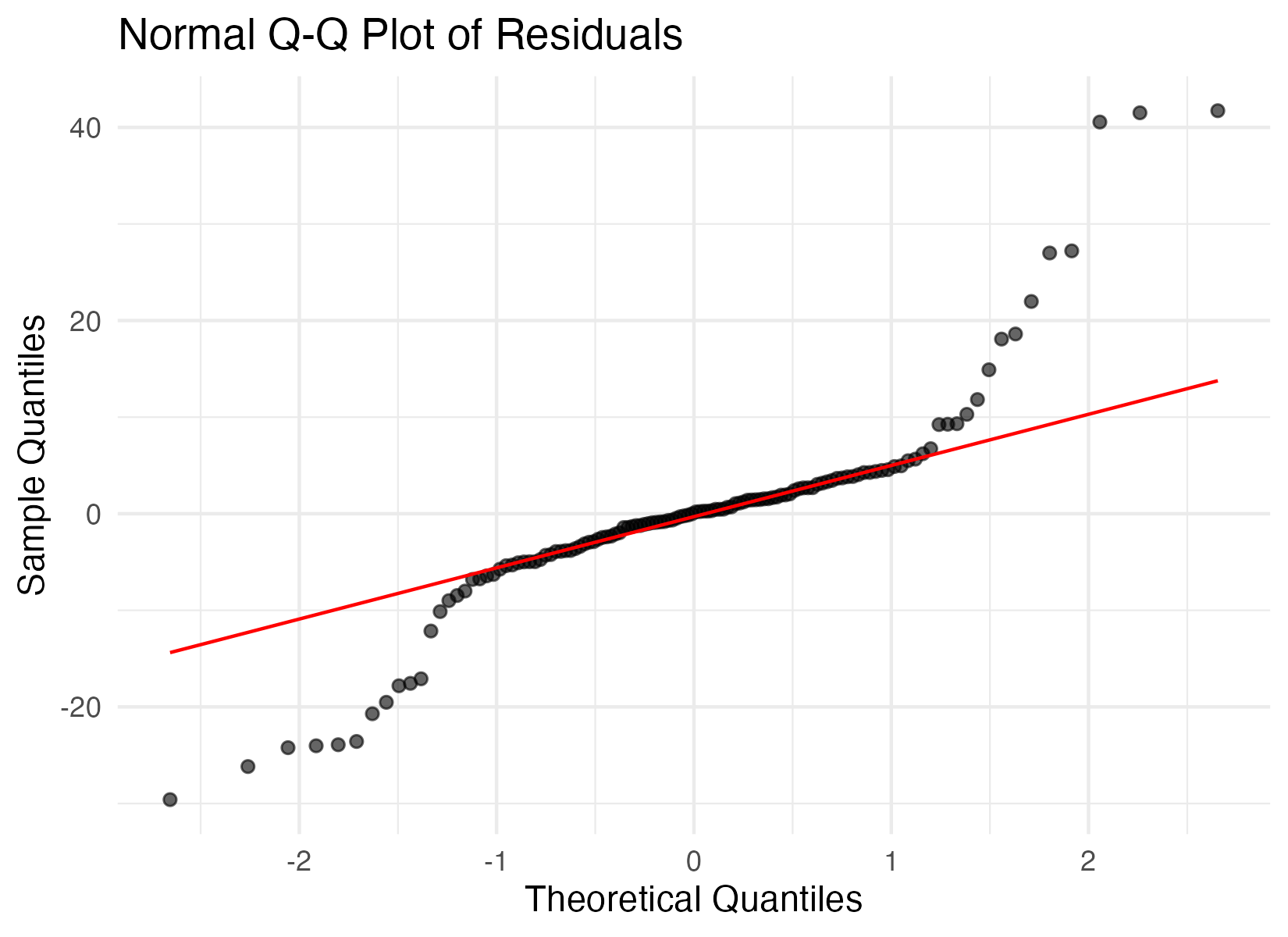


## Within-group change (baseline to follow-up)

| contrast | group | estimate | SE | df | lower.CL | upper.CL | t.ratio | p.value | effect_size |
| --- | --- | --- | --- | --- | --- | --- | --- | --- | --- |
| followup - baseline | C | 22.480 | 2.870 | 115 | 16.796 | 28.164 | 7.834 | <0.001 | 0.379 |
| followup - baseline | S | 23.781 | 2.736 | 115 | 18.361 | 29.200 | 8.691 | <0.001 | 0.401 |

## Between-group difference in change (interaction)

| timepoint_revpairwise | group_revpairwise | estimate | SE | df | lower.CL | upper.CL | t.ratio | p.value | effect_size |
| --- | --- | --- | --- | --- | --- | --- | --- | --- | --- |
| followup - baseline | S - C | 1.301 | 3.965 | 115 | -6.553 | 9.154 | 0.328 | 0.743 | 0.022 |

## Adjusted Means Over Time (with 95% CI)


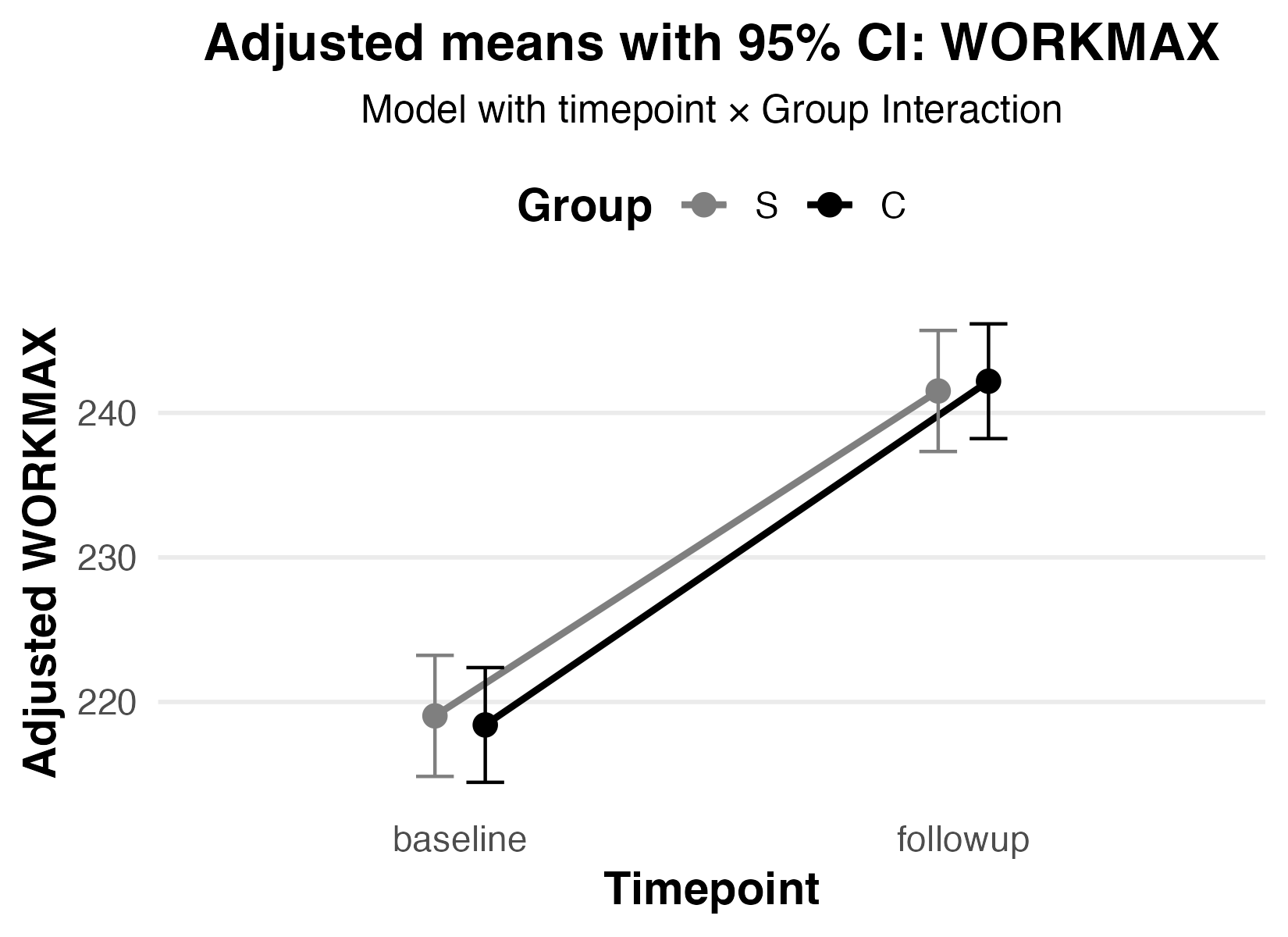


# Outcome: maxhr

## Number of Participants Included: 63

## Distribution of DV at Baseline


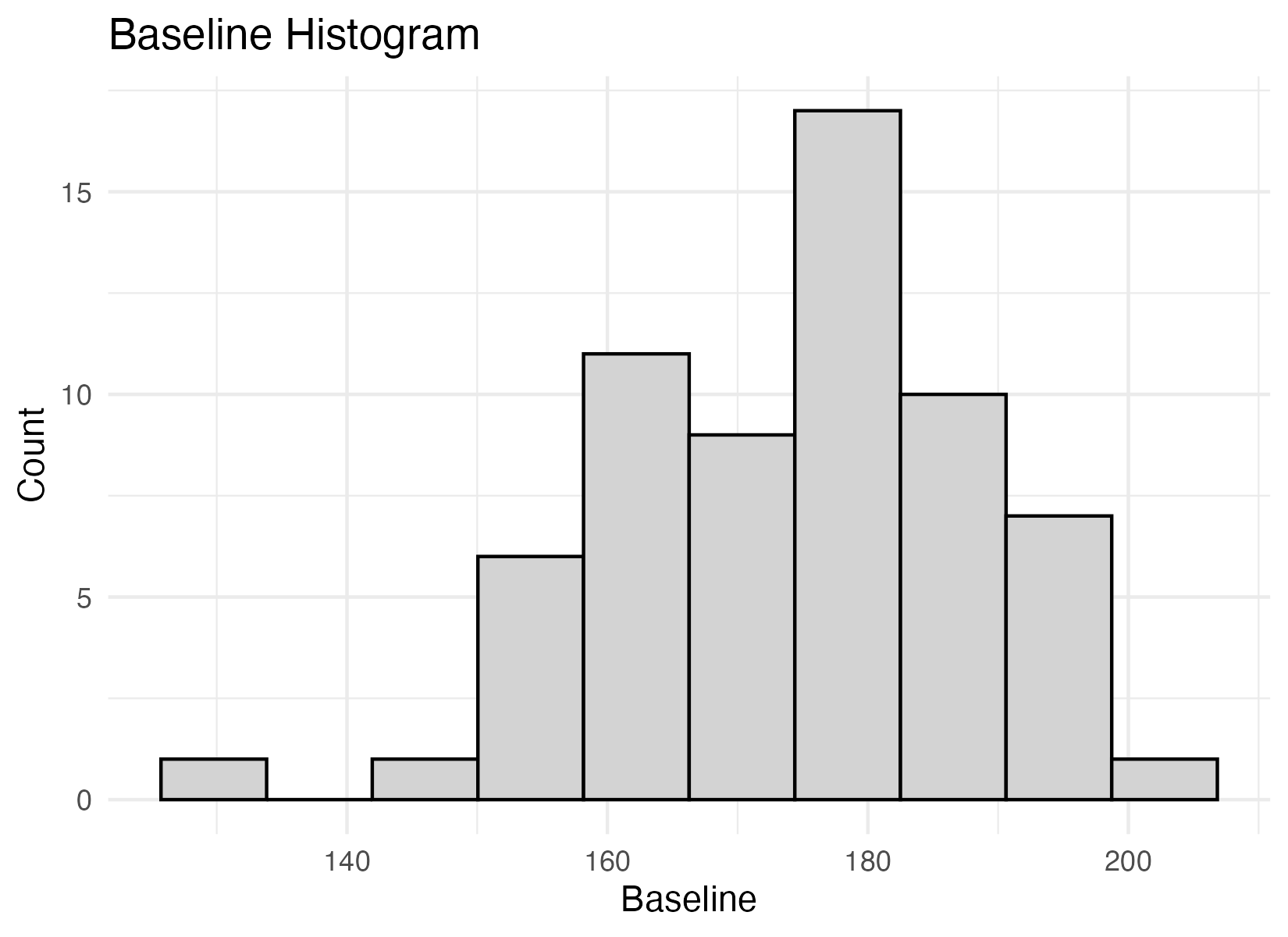


## Fitted vs Residuals


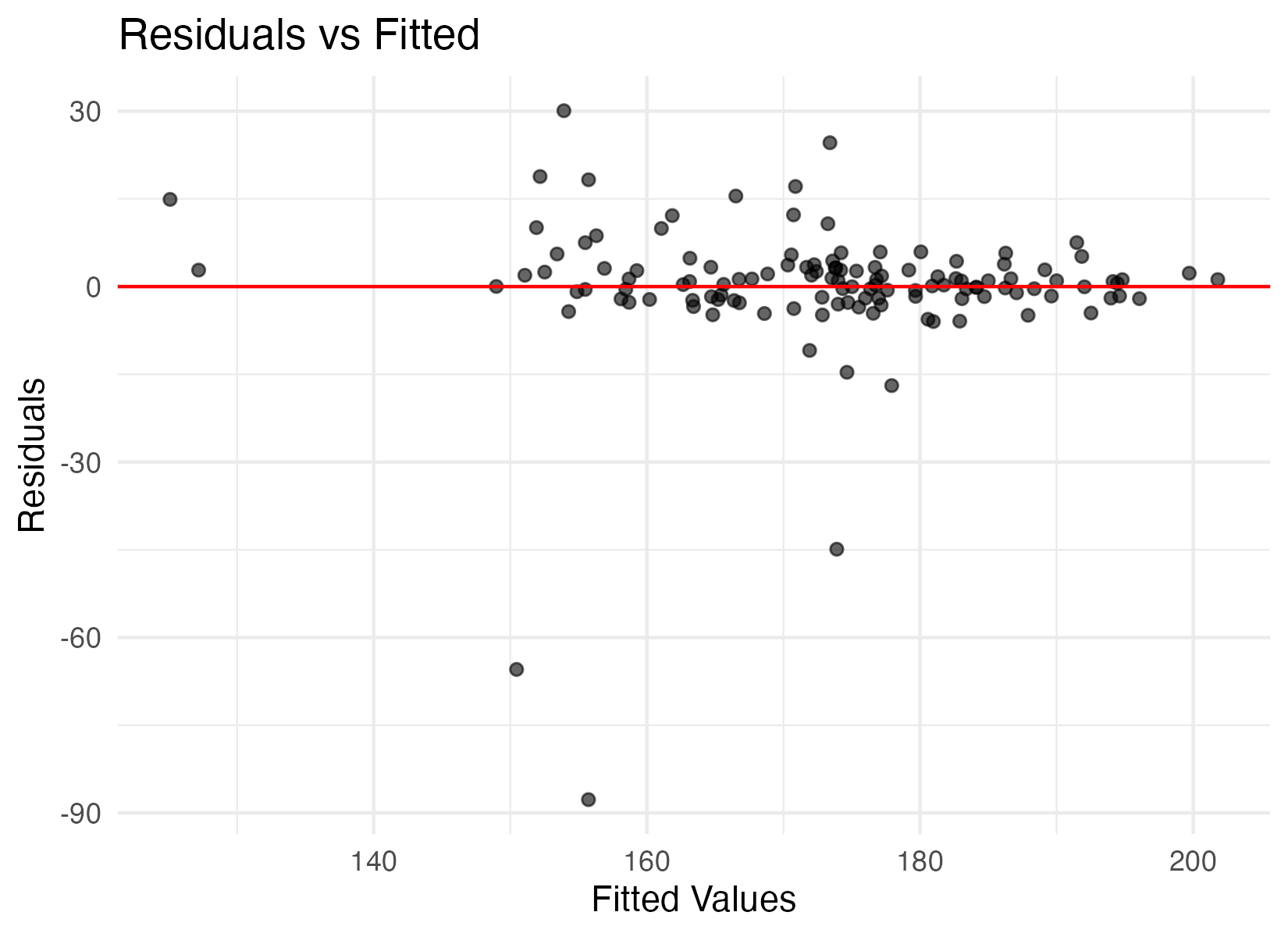


## QQ Plot


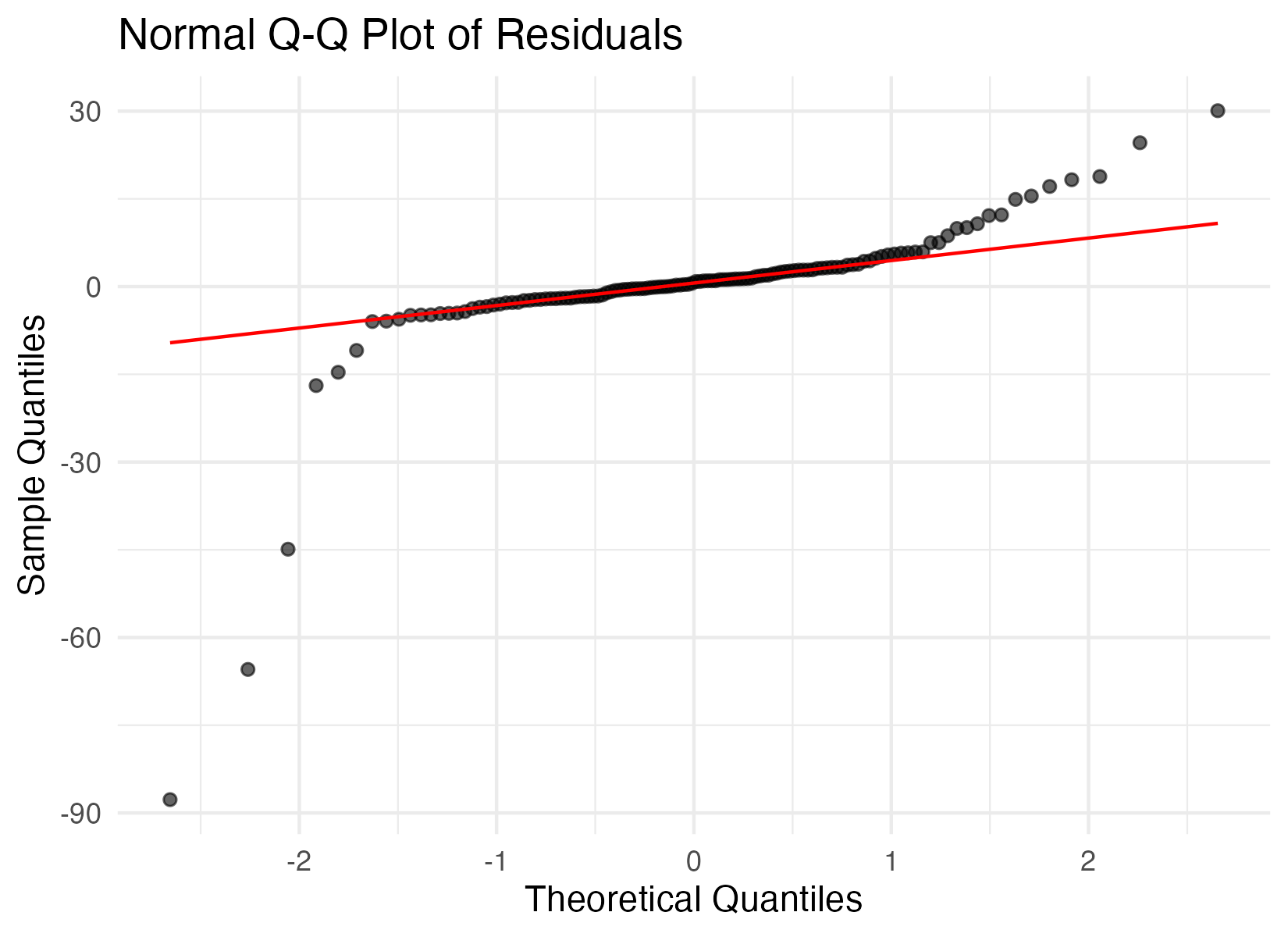


## Within-group change (baseline to follow-up)

| contrast | group | estimate | SE | df | lower.CL | upper.CL | t.ratio | p.value | effect_size |
| --- | --- | --- | --- | --- | --- | --- | --- | --- | --- |
| followup - baseline | C | -2.967 | 3.193 | 115 | -9.291 | 3.358 | -0.929 | 0.355 | -0.162 |
| followup - baseline | S | -2.091 | 3.044 | 115 | -8.121 | 3.939 | -0.687 | 0.494 | -0.114 |

## Between-group difference in change (interaction)

| timepoint_revpairwise | group_revpairwise | estimate | SE | df | lower.CL | upper.CL | t.ratio | p.value | effect_size |
| --- | --- | --- | --- | --- | --- | --- | --- | --- | --- |
| followup - baseline | S - C | 0.876 | 4.412 | 115 | -7.863 | 9.614 | 0.199 | 0.843 | 0.048 |

## Adjusted Means Over Time (with 95% CI)


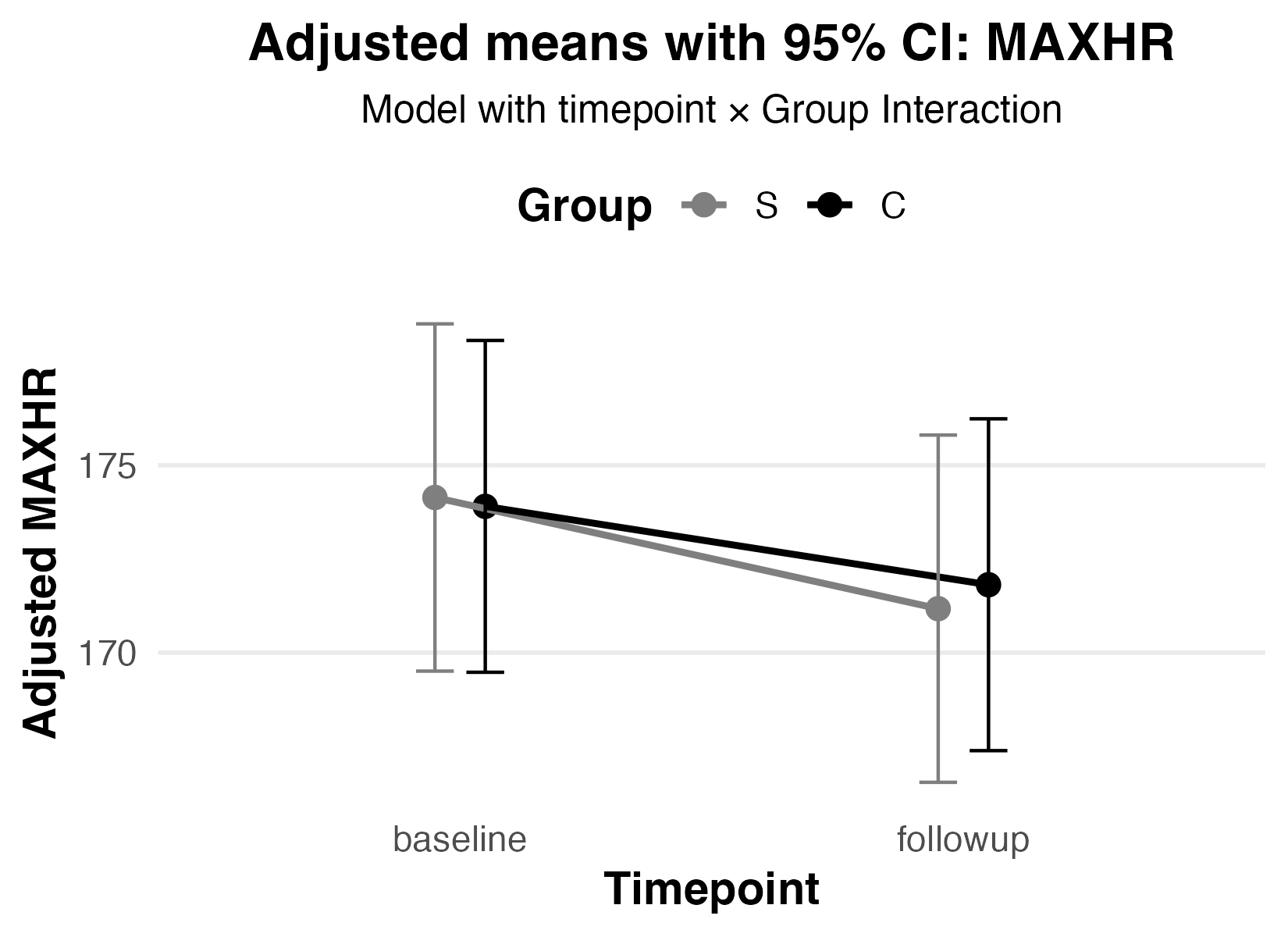

Supplement: Supplementary file 2 — Supporting Information S2 [file EJSC-26-e70199-s001.docx]
